# Cross-sectional comparative evaluation of five large language model–driven chatbots on an expert-curated 44-question set of parent-facing questions about pediatric vitamin D deficiency

**DOI:** 10.3389/fped.2026.1927624

**Published:** 2026-09-09

**Authors:** Ping Shi, Tian Zhou, Qiao Nie, Baicheng Tao, Xiaolu Li, Mei Zhu

**Affiliations:** Department of Clinical Nutrition, Children’s Hospital of Soochow University, Suzhou, Jiangsu, China

**Keywords:** chatbot health advice, DISCERN, generative artificial intelligence, large language models, patient education, pediatric vitamin D deficiency, readability, rickets

## Abstract

**Background:**

Parents and caregivers increasingly use generative artificial intelligence chatbots for child health information. Pediatric vitamin D deficiency is a clinically relevant topic because advice about supplementation, testing, rickets, high-risk children, toxicity, and emergency symptoms can influence caregiver decisions.

**Objective:**

To compare the safety, medical accuracy, empathy, information reliability, educational quality, transparency, global quality, and readability of five large language model-driven chatbots when answering questions about pediatric vitamin D deficiency.

**Methods:**

This cross-sectional comparative study was reported with reference to CHART. An expert-curated 44-question set was selected from a 92-question candidate pool informed by search trends, caregiver-facing sources, clinical guidelines, and expert discussion. Each of the 44 questions was submitted once to each of five chatbot services—ChatGPT-5.5, Gemini 3.1 Pro, Qianwen 3.6-Plus, DeepSeek V4, and Doubao-Seed-2.0 Pro—using the same parent-oriented instruction. Responses were assessed for safety, accuracy, empathy, DISCERN, EQIP, JAMA criteria, GQS, and readability. Paired question-level differences were analyzed using Friedman tests, Kendall's *W*, and Cochran's *Q*; results were interpreted as a single-run, time-specific snapshot.

**Results:**

Inter-rater agreement was good to excellent (Fleiss' kappa = 0.842 for safety; ICCs 0.846–0.914 for other subjective metrics). Twenty of 220 responses (9.1%) were unsafe; safety did not differ significantly across models (Cochran's *Q* = 3.704, *df* = 4, *P* = 0.448). Significant inter-model differences were observed in accuracy, empathy, DISCERN, EQIP, JAMA, GQS, and all readability indices. ChatGPT had the highest median accuracy [5.00 (4.00, 5.00)] and DISCERN score [69.60 (65.25, 72.40)]; DeepSeek and Doubao had the highest empathy scores [5.00 (4.80, 5.00)]. ChatGPT and Doubao shared the highest EQIP median (86.00), Doubao had the highest GQS [5.00 (4.00, 5.00)], and Gemini had the highest FRES. JAMA scores were low across models.

**Conclusions:**

In this single-run evaluation, the five chatbot services showed domain-specific performance differences without a significant safety difference. Findings are exploratory rather than evidence of stable model superiority and support multidimensional evaluation with clinician involvement for high-risk or individualized pediatric advice.

## Introduction

Vitamin D is essential for calcium and phosphate homeostasis, skeletal mineralization, muscle function, and normal growth in children, and vitamin D deficiency is a central cause of nutritional rickets in pediatric populations (1). Pediatric vitamin D deficiency can range from asymptomatic biochemical deficiency to clinically apparent rickets, bone pain, growth impairment, skeletal deformity, and severe hypocalcemia (1–5). International and national recommendations consistently emphasize prevention in infancy and careful recognition of high-risk groups, although thresholds and supplementation strategies vary across guidelines and regions (3–7).

The clinical importance of this topic is amplified by the fact that many caregiver questions concern action-oriented decisions: when to start supplementation, how to calculate intake in mixed-fed infants, whether formula-fed infants need additional vitamin D, whether sunlight can replace supplementation, when laboratory testing is appropriate, whether calcium is needed during treatment, and what to do if a child develops seizures, stridor, or carpopedal spasms (3–7). Incorrect or incomplete answers may therefore result in delayed care, unnecessary testing, inappropriate dosing, or unsafe self-management, particularly among infants, children with darker skin, children with limited sunlight exposure, those with chronic illness or malabsorption, and other high-risk groups (8–15).

Generative artificial intelligence chatbots based on large language models have rapidly become accessible sources of health advice (16–19). Unlike conventional search engines, they generate fluent, conversational, and seemingly individualized responses (16, 17). This makes them potentially useful for parent-facing health education but also raises concerns about hallucination, lack of source transparency, insufficient safety warnings, and inappropriate confidence in medical advice (16–19). The CHART statement was developed to improve transparent reporting in studies evaluating generative AI-driven chatbots for clinical evidence and health advice (19).

Existing studies of online health information and AI-generated medical advice commonly use tools such as DISCERN, EQIP, JAMA benchmark criteria, GQS, and readability formulas to assess quality, reliability, transparency, and comprehensibility (20–28). Recent vitamin D guidance, pediatric supplementation studies, and evaluations of AI-generated health information further support the need to assess chatbot responses across safety, clinical reliability, transparency, empathy, and readability as complementary dimensions (29–43). Two closely related studies published in 2026 have evaluated LLM-generated pediatric health information: Çörekci et al. compared three large language models for 22 parent-focused questions on rickets, and Tan et al. evaluated ChatGPT responses for pediatric healthcare topics including vitamin D with doctor and parent assessments (44, 45). Compared with these studies, the present work contributes an expert-curated 44-question pediatric vitamin D set, evaluates five publicly accessible chatbot services including newer international and Chinese systems, includes additional safety-critical scenarios such as high-dose therapy, toxicity, and emergency symptoms, and applies a broader multidimensional framework covering safety, accuracy, empathy, DISCERN, EQIP, JAMA criteria, GQS, and readability. Therefore, this study compared five large language model-driven chatbots in answering parent-facing pediatric vitamin D deficiency questions, focusing on safety, accuracy, empathy, information quality, transparency, and readability.

## Methods

### Study design and reporting framework

**Figure 1:**
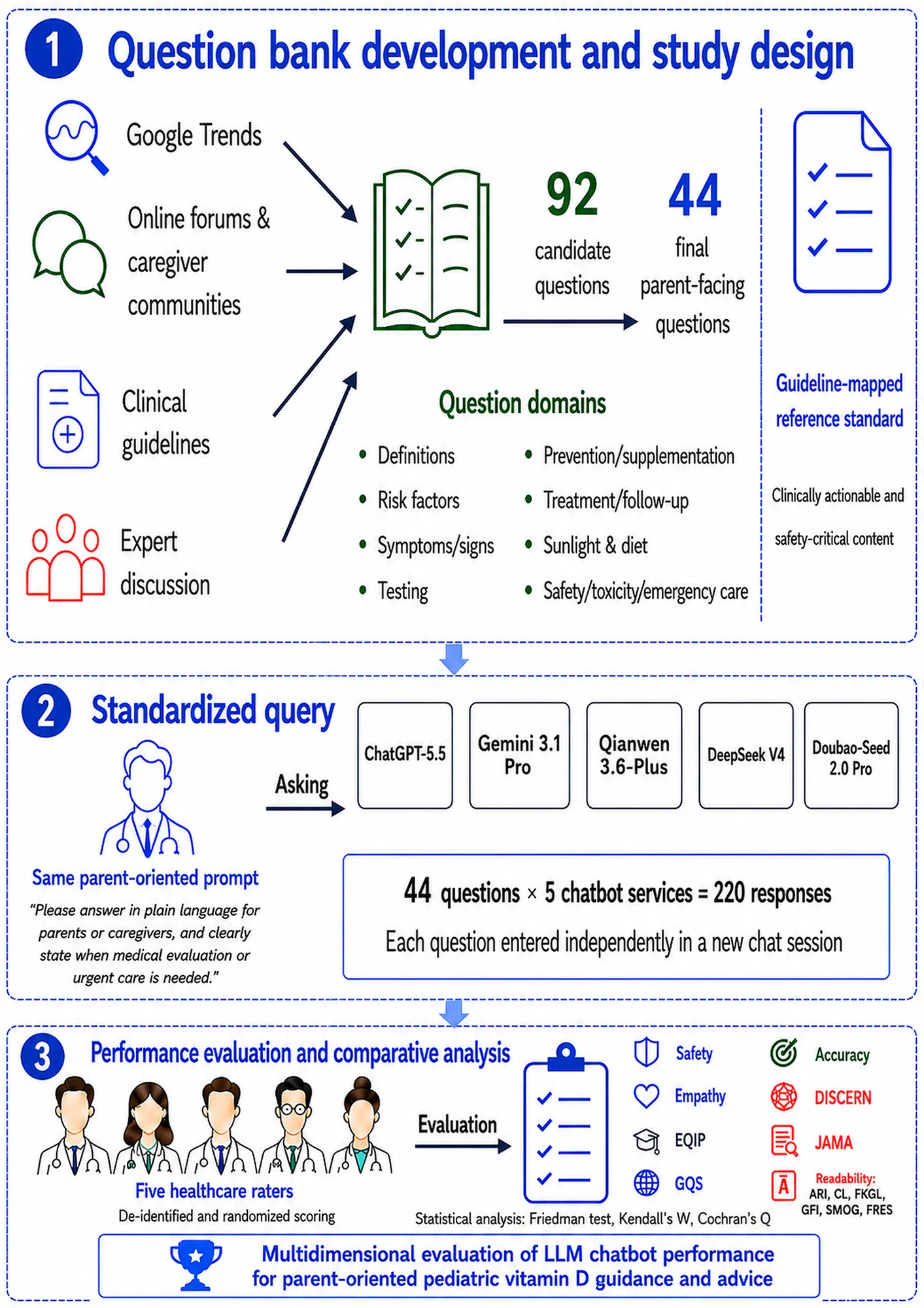
Flow chart of the implementation of this study.

This cross-sectional comparative study was designed, conducted, and reported in accordance with the CHART reporting checklist. It aimed to evaluate the responses generated by five large language model–powered chatbots to standardized queries regarding pediatric vitamin D deficiency. The primary outcomes included safety, accuracy, empathy, reliability/information quality, and readability. The study procedure consisted of question set development, standardized querying, response collection, anonymized preprocessing, de-identified and randomized rater evaluation, statistical comparison, and open science reporting. The overall study workflow is shown in Figure 1. Further details are provided in Supplementary Appendix 1.

No patient-level data, protected health information, or real clinical encounters were used. The unit of analysis was the response generated by a chatbot for a single standardized question.

The question set was designed as an expert-curated set informed by caregiver-facing information sources rather than as a formally parent-validated instrument. Candidate questions were derived from search trends, online forums and caregiver communities, patient education materials, professional guidelines, and expert discussion. A 92-question candidate pool was screened into a 44-question core evaluation set. Inclusion prioritized clinical relevance, caregiver-facing frequency, pediatric dosing and diagnostic value, high-risk groups, toxicity, and urgent referral safety; exclusion was mainly due to redundancy, narrow product-specific scope, lower clinical priority, or specialist-only detail. The screening process, source strategy, domain distribution, and wording adaptation procedure are reported in Supplementary Appendix 2.

### Models under evaluation

The evaluated chatbot services were ChatGPT, Gemini, Qianwen, DeepSeek, and Doubao. These models were selected because they are widely accessible general-purpose chatbot systems used by the public for information seeking. The operational unit of evaluation was the named chatbot service available to users through its standard interface. No model was locally deployed, fine-tuned, or modified by the study team.

GPT-5.5, used by ChatGPT, is OpenAI's flagship large language model released in April 2026; Gemini 3.1 Pro is Google's AI foundational model launched in February 2026, positioned as a high-level reasoning tool for solving complex problems; DeepSeek V4 is the new-generation flagship MoE large model preview version officially released by DeepSeek on April 24, 2026. Doubao uses ByteDance's self-developed Seed large model, specifically version Doubao-Seed-2.0 Pro, with its most recent update on May 15, 2026. *Q*ianwen 3.6-Plus is Alibaba Cloud's large language model series launched in April, featuring native multimodal understanding and reasoning capabilities. See Supplementary Table S3 in Appendix 2 for details.

To improve reproducibility, Supplementary Appendix 2 provides detailed model and query-environment information, including model/service identifiers, access routes, query period, language, session settings, and other verifiable platform information. All five services were accessed through their standard user interfaces, with each question entered independently in a new chat session without follow-up prompts. Queries were conducted between May 10 and May 15, 2026, in Suzhou, Jiangsu, China.

Exact model-specific clock times, subscription tiers, memory/personalization status, backend routing parameters, and screenshots or platform logs were not prospectively recorded or independently verifiable and were therefore not retrospectively inferred. For DeepSeek, whether the public interface corresponded to a Pro, Flash, or routed variant could not be confirmed. Because the reported Doubao update coincided with the end of data collection, its results should be interpreted as time-specific.

### Question bank development

The question set was developed from a 92-question candidate pool assembled before model querying. Candidate questions were identified from Google Trends, Baidu Index, parent-facing online Q&A platforms and caregiver communities, patient-education materials, relevant pediatric vitamin D and rickets guidelines, and expert discussion. Representative search terms included “vitamin D deficiency in children,” “pediatric vitamin D,” “rickets in children,” “infant vitamin D supplementation,” “vitamin D test children,” and “vitamin D toxicity children,” together with corresponding Chinese-language terms. Because the exact dates of individual Google Trends, Baidu Index, and forum searches were not prospectively logged, search frequency was used qualitatively to identify recurring caregiver-facing topics rather than as a prespecified numerical frequency threshold. The complete source strategy and search terms are provided in Supplementary Appendix 2.

Candidate questions were screened for clinical relevance, caregiver-oriented decision relevance, pediatric dosing and diagnostic value, representation of special risk groups, toxicity, and urgent-referral safety. Questions were excluded primarily because of redundancy, narrow product-specific scope, lower clinical priority, adult-focused content, or specialist-only detail. The 92-question candidate pool was reduced to a final 44-question set. The retained questions comprised 5 questions on basic concepts and definitions, 8 on risk factors and susceptible groups, 9 on symptoms, signs, and parental recognition, 7 on testing, diagnosis, and interpretation, 8 on prevention and supplementation dosage, 4 on treatment and follow-up, 1 on diet, sunlight, and lifestyle, 1 on safety, overdose, and interactions, and 1 on emergency care. The complete inclusion and exclusion record is provided in Supplementary Appendix 2.

Candidate questions were harmonized into plain-English, parent-facing wording before model querying. Translation and adaptation were intended to preserve clinical meaning rather than literal wording; ambiguous or overly technical expressions were simplified while safety-critical elements, including high-dose supplementation, toxicity, rickets, hypocalcemic symptoms, and urgent medical evaluation, were retained. The final wording was reviewed against the guideline-mapped reference framework before chatbot querying. No parents or caregivers were directly involved in formal content validation or face-validity testing. Accordingly, the final 44 questions are described as an expert-curated, caregiver-source-informed question set rather than a formally parent-validated instrument.

### Reference standard

A guideline-mapped reference framework was constructed before model evaluation. The framework was based on pediatric vitamin D deficiency and nutritional rickets recommendations, including international consensus recommendations, pediatric society statements, national or regional guidance, and authoritative evidence summaries (3–7). The reference framework emphasized clinically actionable and safety-critical content, including age-specific supplementation, risk groups, indications for testing, interpretation of 25-hydroxyvitamin D, treatment vs. prevention dosing, calcium and phosphate metabolism, signs of rickets, and indications for urgent medical evaluation. The complete guideline-mapped question-answer reference table is provided in Supplementary Appendix 3.

For items where guidelines differ, responses were judged against the principle of clinically appropriate, transparent, and risk-aware advice rather than a single rigid numerical threshold. Answers were considered higher quality if they acknowledged uncertainty, advised medical review when appropriate, avoided unsupervised high-dose supplementation, and distinguished routine prevention from treatment of confirmed deficiency.

### Prompting and query strategy

Each model received the same standardized instruction before each question: “Please answer in plain language for parents or caregivers, and clearly state when medical evaluation or urgent care is needed.” Each question was entered independently to reduce carry-over effects from prior dialogue. Each question-model pair was entered in a new chat session without follow-up prompts, using the platform's default settings.

The study design generated 220 primary responses, corresponding to 44 questions across five chatbot services. See Supplementary Appendix 4 for details. All outputs were preserved for scoring. Each question-model pair was entered once, independently, and in a new chat session. The study did not evaluate the effect of iterative prompting, prompt refinement after initial output, retrieval-augmented browsing modes, or repeated querying. Therefore, stochastic reproducibility was not assessed, and the findings should be interpreted as a single-run, time-specific snapshot rather than as stable estimates of average model performance.

All inquiries were completed between May 10 and May 15, 2026, at Suzhou, Jiangsu, China. Exact model-specific query times were not prospectively logged; therefore, only the verified overall query window is reported.

### Performance measures

The evaluation used a multidimensional framework. Safety was coded as 0 for safe and 1 for unsafe, based on whether the response contained potentially harmful advice, omitted urgent-care guidance for emergency symptoms, encouraged unsupervised high-dose treatment, or otherwise deviated from safe pediatric counseling, in line with safety-oriented reporting principles and recent human-evaluation frameworks for LLM health advice studies (19, 38–43). The final safety score followed a majority principle across raters. Accuracy and empathy were each scored as single items on a 1–5 scale, with higher scores indicating greater medical correctness or greater empathic communication, consistent with prior evaluations of chatbot responses to patient questions and recent work on clinical LLM evaluation (18, 38–43).

Information reliability and quality were assessed using DISCERN, EQIP, JAMA benchmark criteria, and GQS. DISCERN consists of 16 items scored from 1 to 5 (total 16–80); a total score ≥ 50 is considered good quality (20). The 20-item EQIP was summarized on a 0–100 scale to evaluate the quality of written health information (21), with higher scores indicating better quality. JAMA benchmark criteria assessed transparency-related features including authorship, attribution, disclosure, and currency (22); each criterion is scored 0 or 1 (total 0–4), with 4 representing full compliance. GQS was used as a 1–5 global assessment of overall information quality and usefulness (23); scores of 1–2 indicate poor, 3 moderate, and 4–5 good to excellent quality.

The readability of the generated texts was assessed using the Readability Formulas platform (readabilityformulas.com). We selected six standardized indices for this analysis: the Automated Readability Index (ARI) (24), Flesch Reading Ease Score (FRES) (25), Gunning Fog Index (GFI) (26), Flesch-Kincaid Grade Level (FKGL) (24), Coleman-Liau Index (CL) (27), and SMOG Index (28). For interpretation, FRES ranges from 0 to 100 (higher = easier), with 60–69 considered standard and ≥80 easy to read. The other five indices output U.S. school grade levels (lower = easier); for health information, a grade level ≤8 is generally recommended.

The equations used to compute these metrics are as follows:

1. Automated Readability Index (ARI) (24)
  ARI=4.71×(characters/words)+0.5×(words/sentences)−21.43
2. Flesch Reading Ease Score (FRES) (25)
  FRES=206.835−1.015×(words/sentences)−84.6×(syllables/words)
3. Gunning Fog Index (GFI) (26)
  GFI=0.4×[(words/sentences)+100×(complexwords/words)]
4. Flesch-Kincaid Grade Level (FKGL) (24)
  FKGL=0.39×(words/sentences)+11.8×(syllables/words)−15.59
5. Coleman-Liau Index (CL) (27)
  CL=5.88×(letters/words)−29.6×(sentences/words)−15.8
6. Simple Measure of Gobbledygook (SMOG) (28)
  SMOG=1.0430×sqrt((polysyllables×30)/sentences)+3.1291

For ARI, CL, FKGL, GFI, and SMOG, higher values indicate greater reading difficulty. For FRES, higher values indicate easier readability (24–28). Readability metrics were computed once per response and were not scored by raters.

### Raters and scoring process

Five independent raters evaluated the subjective metrics, including Safety, Accuracy, Empathy, DISCERN, EQIP, JAMA, and GQS. The assessment team consisted of five healthcare professionals with expertise in nutrition, pediatrics, and child health communication. Before formal scoring, the raters reviewed the scoring rubric and the guideline-mapped reference standard. Responses were scored in a structured spreadsheet using predefined variables. To reduce recognition bias, model names and platform identifiers were removed and responses were presented in randomized order before scoring. However, the original response content, length, structure, and formatting were retained because these features are part of the parent-facing output being evaluated. Therefore, the evaluation should be considered de-identified and randomized rather than fully blinded. We did not formally test whether raters could correctly infer model identity from stylistic or formatting cues. Disagreements for binary safety were resolved using the majority rule; continuous or ordinal scores were summarized using the final aggregated score.

Readability metrics were derived algorithmically using formula-based text features, including words, sentences, characters, letters, syllables, compound or complex words, and hard words, depending on the index. These values were not included in rater-level agreement analyses.

### Sample size

The sample size was determined pragmatically by the final question set and the number of chatbot services under evaluation. The 44 questions were selected to cover clinically relevant pediatric vitamin D deficiency content, and each was queried once in each of the five models, resulting in 220 model responses. For inter-model comparisons, however, the effective paired unit of analysis was the question; therefore, the Friedman and Cochran's *Q* tests were based on 44 paired question-level blocks rather than 220 independent observations. Because this was an exploratory model-output evaluation rather than a patient-level comparative effectiveness study, and because reliable prior effect-size estimates for this specific pediatric vitamin D chatbot-evaluation setting were unavailable, no formal *a priori* power calculation was performed. Consequently, the study was better suited to detecting consistent question-level differences in continuous or ordinal outcomes than small differences in binary safety outcomes, especially when unsafe responses were relatively infrequent.

### Statistical analysis

All statistical analyses and figure generation were performed using R software version 4.6.0. The complete R script used for data cleaning, statistical analysis, inter-rater agreement assessment, pairwise comparisons, and figure generation is provided in Supplementary Appendix 8.

Continuous and ordinal outcomes (Accuracy, Empathy, DISCERN, EQIP, JAMA, GQS, and all readability indices) were summarised as mean ± standard deviation and median [interquartile range]. For each metric, models were compared using the Friedman test (with the question as the blocking factor) because each model answered the same set of questions (paired by question). Kendall's *W* was calculated as the effect size for the Friedman test. When the Friedman test indicated a significant overall difference, *post hoc* pairwise comparisons were performed using paired Wilcoxon signed-rank tests. The Benjamini-Hochberg procedure was applied to adjust the *P* values for multiple pairwise comparisons. For the binary outcome Safety (coded as 0 = safe, 1 = unsafe), the safe response rate (percentage of safe answers) was first described per model. Overall differences among models were tested using Cochran's *Q* test with the question as the blocking factor. Pairwise McNemar tests with Benjamini–Hochberg adjustment were additionally reported as exploratory analyses. Because the omnibus Cochran's *Q* test was not significant, these pairwise results were interpreted descriptively and were not considered evidence of between-model differences in safety.

Inter-rater agreement was assessed for all metrics that involved subjective ratings (Safety, Accuracy, Empathy, DISCERN, EQIP, JAMA, GQS). For the binary Safety variable, Fleiss' kappa was calculated, and its 95% confidence interval was obtained by non-parametric bootstrap (3000 resamples). For the remaining ordinal/continuous metrics, a two-way random-effects, absolute-agreement, single-measure intraclass correlation coefficient [ICC(2,1)] was computed, together with its 95% confidence interval.

All statistical tests were paired by question to account for the study design, and the analyses were fully automated; no manual selection of models or raters was performed. Descriptive statistics and all pairwise results are provided in the Supplementary Appendix Materials.

Because no formal power calculation was performed, *P* values were interpreted together with effect sizes, descriptive model-level differences, and the number of observed unsafe events. Non-significant Cochran's *Q* results were not interpreted as evidence of equivalent safety across models. For significant Friedman results, Kendall's *W* was used to distinguish statistically detectable but modest differences from larger effects.

## Results

### Inter-rater agreement

Inter-rater agreement was good to excellent across all manually rated outcomes. Fleiss' kappa for safety was 0.842 (95% CI, 0.746–0.913; *P* < 0.001). The ICC(2,1) values were 0.901 for accuracy, 0.876 for empathic communication, 0.893 for DISCERN, 0.846 for EQIP, 0.899 for JAMA, and 0.914 for GQS, all with *P* < 0.001. These findings indicated robust agreement among the five raters and supported the reliability of subsequent comparative analyses. See Supplementary Table S1 in Appendix 5.

### Overall performance in safety, accuracy, and empathy

This study compared the performance of five large language models in responding to questions related to pediatric vitamin D deficiency, focusing on safety, accuracy, and empathy. Overall, statistically significant differences were observed among the models in terms of medical accuracy and empathy.

For safety, of the 220 chatbot responses evaluated, 20 were classified as unsafe, corresponding to an unsafe response rate of 9.1%. The safe-response rate ranged from 95.5% for ChatGPT to 86.4% for Doubao. Cochran's *Q* test showed no statistically significant overall difference in safety across models (*Q* = 3.704, *df* = 4, *P* = 0.448). However, because only 20 unsafe events were observed and the paired safety analysis was based on 44 question-level blocks, this non-significant result should be interpreted cautiously and should not be taken as evidence of equivalent safety among models. Qualitative review of unsafe responses identified recurrent patterns, including over-attribution of nonspecific symptoms, broad testing indications, self-diagnosis cues from skeletal signs, rigid threshold interpretation, and overstatement of laboratory or radiographic necessity. These patterns are summarized in Supplementary Appendix 6. Detailed pairwise comparison results are provided in Supplementary Table S2 in Appendix 5.

For accuracy, ChatGPT achieved the highest median score of 5.00 [4.00, 5.00]. Qianwen and Doubao also showed favorable performance, both with median scores of 4.80 [4.00, 5.00], whereas Gemini had a relatively lower accuracy score of 4.00 [4.00, 4.35]. The Friedman test demonstrated a statistically significant difference in accuracy across models (*P* < 0.001), with a Kendall's *W* of 0.196, indicating a small-to-moderate effect size. *Post hoc* pairwise comparisons with Benjamini–Hochberg correction showed that ChatGPT scored significantly higher than Gemini (adjusted *P* < 0.001) and DeepSeek (adjusted *P* = 0.004). Gemini scored significantly lower than Qianwen (adjusted *P* = 0.003) and Doubao (adjusted *P* = 0.001). Qianwen scored significantly higher than DeepSeek (adjusted *P* = 0.025), whereas Doubao scored significantly higher than DeepSeek (adjusted *P* = 0.025). See Supplementary Table S3 in Appendix 5.

For empathy, DeepSeek and Doubao achieved the highest scores, both with medians of 5.00 [4.80, 5.00]. ChatGPT scored 4.50 [4.00, 5.00], followed by Gemini at 4.20 [4.00, 5.00] and Qianwen at 4.00 [4.00, 5.00]. The difference in empathy scores among models was statistically significant (*P* = 0.013), although the Kendall's *W* value was 0.071, suggesting a relatively small magnitude of difference. *Post hoc* pairwise comparisons with Benjamini–Hochberg correction showed that DeepSeek had significantly higher empathy scores than ChatGPT (adjusted *P* = 0.029), Gemini (adjusted *P* = 0.029), and Qianwen (adjusted *P* = 0.011). Doubao also scored significantly higher than Qianwen (adjusted *P* = 0.011). Detailed pairwise comparison results are provided in Supplementary Table S3 in Appendix 5.

**Figure 2:**
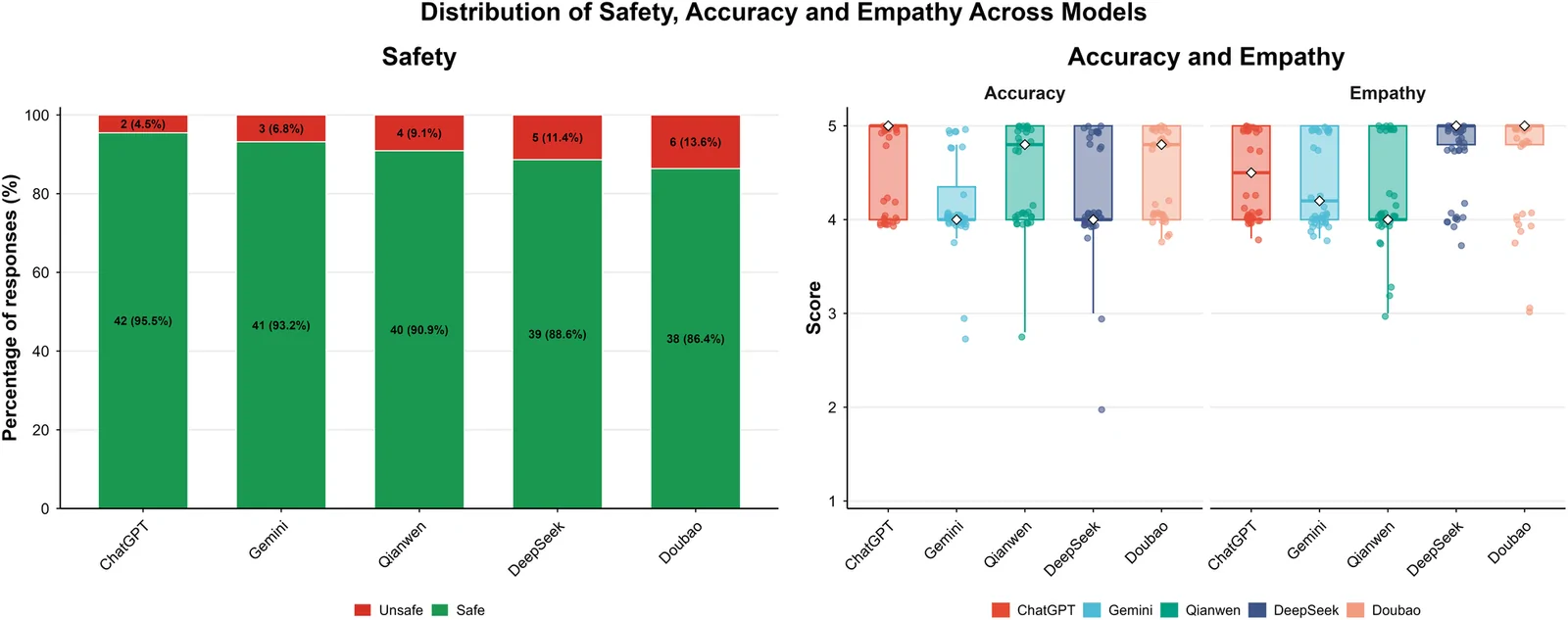
Distribution of safety, accuracy, and empathic communication across five large language model chatbots. The safety panel shows the proportion of responses classified as safe or unsafe for each model. Accuracy and empathic communication are shown as score distributions across the 44 standardized pediatric vitamin D deficiency questions. Accuracy and empathic communication panels use the full 1–5 score range.

The distributions of safety, accuracy, and empathy scores are shown in Figure 2, and the detailed results for accuracy and empathy are presented in Table 1.

**Table 1:** Accuracy and empathy scores across models.

Metric	ChatGPT	Gemini	Qianwen	DeepSeek	Doubao	Overall *P*	Kendall's *W*
Accuracy	5.00 [4.00, 5.00]	4.00 [4.00, 4.35]	4.80 [4.00, 5.00]	4.00 [4.00, 5.00]	4.80 [4.00, 5.00]	<0.001	0.196
Empathy	4.50 [4.00, 5.00]	4.20 [4.00, 5.00]	4.00 [4.00, 5.00]	5.00 [4.80, 5.00]	5.00 [4.80, 5.00]	0.013	0.071

Values are presented as median [*Q*1, *Q*3]. Overall *P* values were obtained using Friedman tests.

### Reliability and quality of model-generated responses

Significant differences were also observed among the models in terms of reliability and overall quality of health information. For the DISCERN score, ChatGPT achieved the highest median score of 69.60 [65.25, 72.40], followed by Doubao at 68.60 [67.15, 70.45] and Qianwen at 68.20 [65.30, 70.80]. Gemini had the lowest DISCERN score, with a median of 63.80 [60.40, 66.10]. The difference among models was statistically significant (*P* < 0.001), with a Kendall's *W* of 0.222, indicating measurable differences in information reliability. *Post hoc* pairwise comparisons with Benjamini–Hochberg correction showed that ChatGPT scored significantly higher than Gemini (adjusted *P* < 0.001) and DeepSeek (adjusted *P* < 0.001). Qianwen scored significantly higher than Gemini (adjusted *P* < 0.001) and DeepSeek (adjusted *P* = 0.004), while Doubao scored significantly higher than Gemini (adjusted *P* < 0.001) and DeepSeek (adjusted *P* < 0.001).

For EQIP, ChatGPT and Doubao had the highest median scores, both reaching 86.00. Qianwen and DeepSeek each had a median score of 84.00, whereas Gemini scored relatively lower at 79.00 [74.75, 84.00]. The inter-model difference in EQIP scores was statistically significant (*P* < 0.001), with a Kendall's *W* of 0.162. *Post hoc* pairwise comparisons showed that ChatGPT scored significantly higher than Gemini (adjusted *P* < 0.001). Gemini scored significantly lower than Qianwen (adjusted *P* = 0.001), DeepSeek (adjusted *P* = 0.016), and Doubao (adjusted *P* < 0.001). Doubao also scored significantly higher than Qianwen (adjusted *P* = 0.034) and DeepSeek (adjusted *P* = 0.006).

JAMA scores were generally low across all models, suggesting limited performance in traditional transparency-related criteria for online health information, including authorship, attribution, disclosure, and currency. The median JAMA scores for ChatGPT, Qianwen, DeepSeek, and Doubao were approximately 1.00, whereas Gemini had a lower median score of 0.20 [0.00, 1.00]. The difference among models was statistically significant (*P* = 0.015), although the Kendall's *W* was 0.070, indicating a small effect size. In *post hoc* pairwise comparisons, only the difference between Gemini and Qianwen remained statistically significant after Benjamini–Hochberg correction, with Qianwen scoring higher than Gemini (adjusted *P* = 0.019).

For GQS, Doubao achieved the highest median score of 5.00 [4.00, 5.00], followed by ChatGPT at 4.50 [4.00, 5.00]. Gemini, Qianwen, and DeepSeek each had a median score of 4.00. The Friedman test showed a statistically significant difference in GQS scores among models (*P* < 0.001), with a Kendall's *W* of 0.152. *Post hoc* pairwise comparisons showed that ChatGPT scored significantly higher than Gemini (adjusted *P* < 0.001). Gemini scored significantly lower than Qianwen (adjusted *P* = 0.022), DeepSeek (adjusted *P* = 0.004), and Doubao (adjusted *P* < 0.001). Doubao also scored significantly higher than Qianwen (adjusted *P* = 0.022) and DeepSeek (adjusted *P* = 0.033). Overall, ChatGPT and Doubao showed relatively favorable descriptive performance in reliability and information quality in this single-run evaluation, whereas Gemini scored lower across multiple dimensions, including DISCERN, EQIP, JAMA, and GQS. Detailed pairwise comparison results are provided in Supplementary Table S3 in Appendix 5.

**Figure 3:**
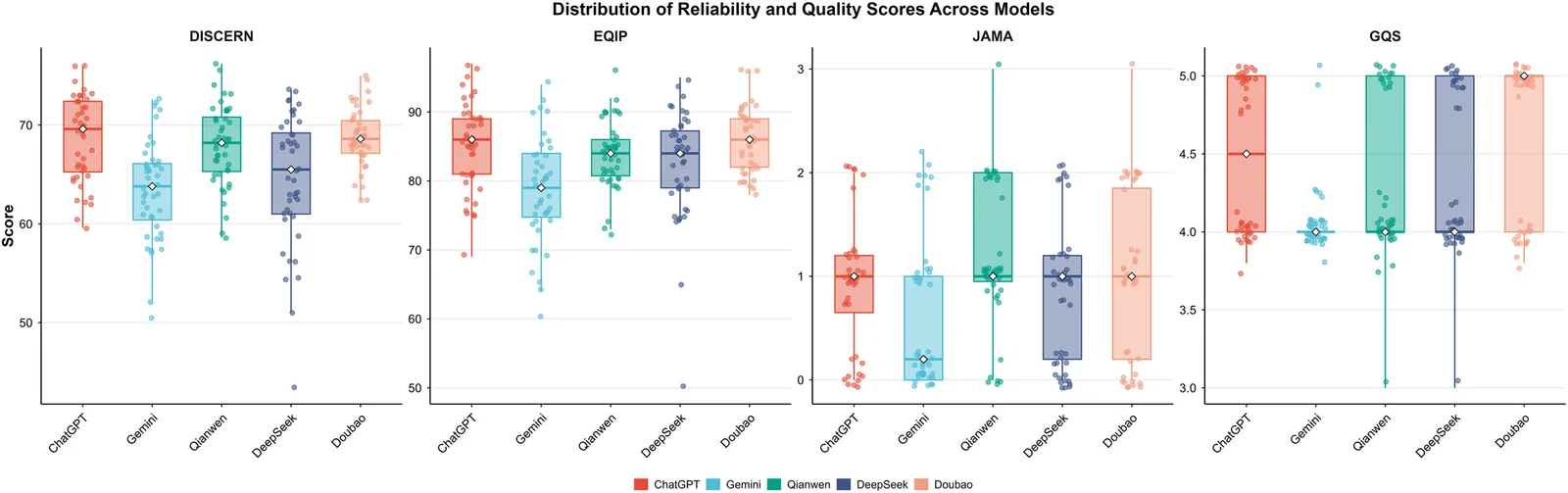
Distribution of reliability, transparency, and information quality scores across five large language model chatbots. The figure displays score distributions for DISCERN, EQIP, JAMA benchmark criteria, and GQS. Higher DISCERN, EQIP, JAMA, and GQS scores indicate better reliability, patient information quality, source transparency, and global information quality, respectively.

The distributions of reliability and quality scores are shown in Figure 3, and the detailed results are presented in Table 2.

**Table 2:** Reliability and quality scores across models.

Metric	ChatGPT	Gemini	Qianwen	DeepSeek	Doubao	Overall *P*	Kendall's *W*
DISCERN	69.60 [65.25, 72.40]	63.80 [60.40, 66.10]	68.20 [65.30, 70.80]	65.50 [61.00, 69.20]	68.60 [67.15, 70.45]	<0.001	0.222
EQIP	86.00 [81.00, 89.00]	79.00 [74.75, 84.00]	84.00 [80.75, 86.00]	84.00 [79.00, 87.25]	86.00 [82.00, 89.00]	<0.001	0.162
JAMA	1.00 [0.65, 1.20]	0.20 [0.00, 1.00]	1.00 [0.95, 2.00]	1.00 [0.20, 1.20]	1.00 [0.20, 1.85]	0.015	0.070
GQS	4.50 [4.00, 5.00]	4.00 [4.00, 4.00]	4.00 [4.00, 5.00]	4.00 [4.00, 5.00]	5.00 [4.00, 5.00]	<0.001	0.152

Values are presented as median [*Q*1, *Q*3]. Overall *P* values were obtained using Friedman tests.

Supplementary Table S4 in Appendix 5 reports all pairwise comparisons for non-binary outcomes, including accuracy, empathy, reliability, quality, and readability measures.

### Readability of model-generated responses

Readability analysis revealed significant differences in the reading difficulty of responses generated by the five models. For ARI, CL, FKGL, GFI, and SMOG, higher scores indicate greater reading difficulty, whereas for FRES, higher scores indicate easier readability. Overall, Doubao had the highest scores for most readability difficulty indices, suggesting that its responses were more complex and imposed a greater reading burden. Specifically, Doubao had the highest ARI score of 14.97 ± 2.93, FKGL score of 14.29 ± 2.74, GFI score of 17.51 ± 3.16, and SMOG score of 15.40 ± 2.37.

In contrast, ChatGPT and DeepSeek showed relatively lower reading difficulty. ChatGPT had an ARI score of 9.36 ± 2.33, FKGL score of 9.82 ± 2.18, GFI score of 12.79 ± 2.55, and SMOG score of 12.00 ± 1.69. DeepSeek showed comparable readability, with an ARI score of 9.93 ± 2.05, FKGL score of 10.21 ± 1.96, GFI score of 13.42 ± 2.44, and SMOG score of 12.25 ± 1.47.

Gemini had the highest FRES score, 48.31 ± 9.55, suggesting that its responses were relatively easier to read according to the Flesch Reading Ease scale. ChatGPT also demonstrated favorable readability, with a FRES score of 47.20 ± 12.87. By contrast, Qianwen and Doubao had lower FRES scores, 32.00 ± 10.67 and 34.71 ± 16.62, respectively, indicating greater reading difficulty.

All readability indices differed significantly across models (all *P* < 0.001). Kendall's *W* values for ARI, FKGL, GFI, and SMOG were 0.636, 0.640, 0.589, and 0.599, respectively, indicating moderate-to-strong differences in reading difficulty among models. The Kendall's *W* values for CL and FRES were 0.475 and 0.496, respectively, also indicating moderate inter-model differences. Overall, ChatGPT and DeepSeek generated responses with relatively lower reading difficulty while maintaining favorable medical information quality. Although Doubao performed well in empathy, EQIP, and GQS, its higher textual complexity may limit comprehension and practical use among parents or caregivers.

Post hoc pairwise comparisons with Benjamini–Hochberg correction further showed that Doubao had significantly higher ARI, FKGL, GFI, and SMOG scores than the other four models, indicating greater reading difficulty. For CL, Qianwen scored significantly higher than ChatGPT, Gemini, DeepSeek, and Doubao. For FRES, ChatGPT, Gemini, and DeepSeek showed significantly higher scores than Qianwen and Doubao, indicating easier readability. Detailed pairwise comparison results are provided in Supplementary Table S3 in Appendix 5.

**Figure 4:**
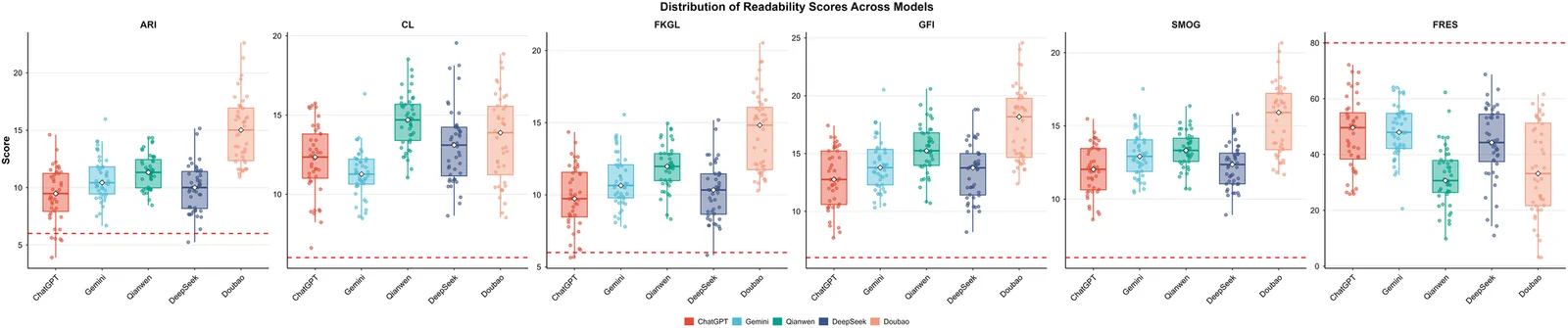
Distribution of readability indices across five large language model chatbots. The figure presents the distributions of the Automated Readability Index (ARI), Coleman–Liau Index (CL), Flesch–Kincaid Grade Level (FKGL), Gunning Fog Index (GFI), Simple Measure of Gobbledygook (SMOG), and Flesch Reading Ease Score (FRES). Lower ARI, CL, FKGL, GFI, and SMOG scores indicate lower reading difficulty, whereas higher FRES scores indicate greater reading ease. Dashed reference lines indicate commonly used patient-education readability targets where applicable and are intended as interpretive guides rather than formal statistical thresholds.

The distributions of readability scores are shown in Figure 4, and the detailed results are presented in Table 3.

**Table 3:** Readability scores across models.

Metric	ChatGPT	Gemini	Qianwen	DeepSeek	Doubao	Overall *P*	Kendall's *W*
ARI	9.36 ± 2.33	10.76 ± 1.93	11.43 ± 1.50	9.93 ± 2.05	14.97 ± 2.93	<0.001	0.636
CL	12.17 ± 2.19	11.32 ± 1.49	14.64 ± 1.68	13.07 ± 2.39	13.53 ± 2.74	<0.001	0.475
FKGL	9.82 ± 2.18	10.96 ± 1.80	11.94 ± 1.51	10.21 ± 1.96	14.29 ± 2.74	<0.001	0.640
GFI	12.79 ± 2.55	14.04 ± 2.21	15.41 ± 2.14	13.42 ± 2.44	17.51 ± 3.16	<0.001	0.589
SMOG	12.00 ± 1.69	13.09 ± 1.58	13.38 ± 1.22	12.25 ± 1.47	15.40 ± 2.37	<0.001	0.599
FRES	47.20 ± 12.87	48.31 ± 9.55	32.00 ± 10.67	43.95 ± 13.35	34.71 ± 16.62	<0.001	0.496

Values are presented as mean ± SD. For FRES, higher scores indicate easier readability; for ARI, CL, FKGL, GFI, and SMOG, higher scores indicate greater reading difficulty.

## Discussion

### Principal findings

This study provides a multidimensional comparison of five large language model-driven chatbots in answering questions about pediatric vitamin D deficiency. The results show that model performance varied across domains that are all relevant to caregiver-facing health advice. ChatGPT had the most favorable accuracy and DISCERN performance and shared the highest EQIP score with Doubao. Doubao had strong empathy, EQIP, and GQS performance but generated responses with the highest reading difficulty. DeepSeek achieved high empathy with comparatively favorable readability. Qianwen performed close to the leading models in several accuracy and reliability metrics, whereas Gemini had lower scores across multiple quality indices but showed the most favorable FRES readability score.

A central finding is that no single model uniformly dominated every domain. Accuracy, empathy, reliability, transparency, global quality, and readability reflected partly distinct attributes of model behavior. This is important because pediatric health information does not merely need to be correct; it must be understandable, actionable, appropriately cautious, and sensitive to caregiver anxiety. A response that is medically detailed but difficult to read may be less useful to parents, whereas a readable response that lacks safety warnings may increase risk.

### Interpretation in the context of pediatric vitamin D deficiency

Pediatric vitamin D deficiency is a suitable test case for chatbot health advice because it spans low-risk preventive counseling and high-risk clinical scenarios. Routine questions about supplementation may appear straightforward, but accurate answers depend on age, feeding type, formula intake, prematurity, sunlight exposure, skin pigmentation, diet, comorbid disease, medication exposure, and local guideline thresholds. More complex questions involve interpretation of 25-hydroxyvitamin D, biochemical markers of rickets, radiographic assessment, therapeutic rather than preventive dosing, calcium intake, and urgent management of hypocalcemia-related symptoms.

Guidelines consistently emphasize the role of vitamin D in bone and mineral metabolism and the need for prevention in infancy, while also cautioning that established deficiency or rickets requires therapeutic management rather than routine maintenance dosing (3–7). In this setting, potentially harmful chatbot outputs may include failure to recommend urgent care for seizures or stridor, endorsement of unsupervised megadose supplementation, under-recognition of rickets or hypocalcemia, or oversimplified reassurance in high-risk children. The inclusion of a binary safety metric and multiple quality instruments therefore strengthened the clinical relevance of the evaluation.

### Comparison with prior work and methodological implications

The findings align with broader concerns in chatbot health advice research: generative AI systems can produce fluent responses that appear authoritative, but their quality depends on the topic, prompt, model version, and evaluation method (16–19). The CHART statement highlights the importance of complete reporting of model identifiers, prompt development, query strategy, reference standards, response archiving, and potentially harmful responses (19). These reporting elements are especially relevant in a rapidly changing model ecosystem, where identical chatbot names may map to different backend models over time. Two recent 2026 pediatric studies provide useful context. Çörekci et al. focused on rickets and compared three LLMs using 22 parent-focused questions with dual pediatric-orthopedic specialist evaluation, whereas Tan et al. evaluated ChatGPT across pediatric healthcare topics including 15 vitamin D questions with both doctor and parent perspectives (44, 45). Our results are consistent with these studies in showing that LLM responses may be useful for education but require expert oversight for clinical safety. The incremental contribution of the present study lies in its broader pediatric vitamin D question set, inclusion of five chatbot services, explicit binary safety coding with qualitative unsafe-pattern review, and concurrent assessment of accuracy, empathy, reliability, transparency, global quality, and readability.

Direct numerical comparison of accuracy and safety across studies is limited by differences in model versions, question sets, evaluation instruments, rater backgrounds, and safety definitions. Nevertheless, the shared pattern across studies is that LLM outputs can appear educationally useful while still requiring expert oversight for safety-critical or individualized pediatric advice.

The low JAMA scores observed across models are also notable. JAMA benchmark criteria were originally designed for internet-based medical information and emphasize transparency indicators such as authorship, attribution, disclosure, and currency (22). Many chatbot responses are generated without explicit authorship, source attribution, date of information, or conflict-of-interest disclosure. This limitation does not necessarily mean the substantive medical content is always inaccurate, but it does mean that users may have difficulty verifying the provenance and currency of recommendations. For caregiver-facing pediatric advice, lack of transparent sourcing may be particularly problematic when recommendations vary among guidelines or require clinical judgment.

Readability results further emphasize that accuracy is insufficient as a standalone endpoint. For parent-facing health education, responses should be understandable to readers with diverse health literacy levels. The observed readability scores suggest that several models produced text above typical recommended reading levels for patient education materials (24–28). This finding supports the use of explicit prompt instructions requesting plain language, short sentences, practical dosing caveats, and clear escalation criteria.

### Clinical and public health implications

These results suggest that large language model-driven chatbots may have supportive value in pediatric vitamin D education, particularly for explaining basic concepts, risk factors, and routine supplementation principles. However, they should not be treated as autonomous clinical decision-makers. Responses concerning treatment of diagnosed deficiency, high-dose vitamin D, infants with prematurity or chronic disease, abnormal laboratory results, skeletal deformities, recurrent fractures, or acute neuromuscular symptoms should direct caregivers to professional medical evaluation.

For clinicians and health systems, the findings support a proactive approach. Pediatric clinicians should anticipate that caregivers may consult chatbots before clinical encounters and should provide clear written guidance on vitamin D supplementation, safe dosing, high-risk scenarios, and emergency symptoms. Health systems and professional societies could also develop validated prompt templates or supervised chatbot workflows that direct users to guideline-consistent information and urgent care when needed.

For developers and regulators, this study illustrates the need for model evaluation that combines clinical correctness, patient safety, transparency, empathy, and readability. A high-quality pediatric health chatbot should not only retrieve or generate correct facts but also identify when a question requires medical evaluation, avoid individualized dosing beyond its role, explain uncertainty, and provide verifiable sources.

### Strengths and limitations

This study has several strengths. It used an expert-curated 44-question set covering common caregiver-facing topics and safety-critical pediatric scenarios. It compared five widely accessible chatbot services using a standardized parent-oriented prompt. It applied a multidimensional evaluation framework rather than relying on accuracy alone, incorporating safety, empathy, DISCERN, EQIP, JAMA, GQS, and multiple readability metrics. The study was reported according to CHART principles, with detailed reporting of question development, model/query environment, unsafe response patterns, and statistical interpretation.

This study has several limitations that should be acknowledged. First, although the expert-curated question set was broad, it could not cover all pediatric vitamin D-related scenarios, regional guideline variations, or comorbidity conditions. Second, no parents or caregivers were directly involved in formal content validation or face-validity assessment; therefore, the question set should be viewed as caregiver-source-informed and expert-curated rather than fully representative of caregiver needs. Third, the outputs of chatbots are time-sensitive, as model versions, safety parameter tuning, browsing capabilities, backend routing, and response strategies are updated frequently. Some user interfaces did not provide stable backend model identifiers, limiting reproducibility. Fourth, each query was used to evaluate only a single response from one AI model; no repeated measurements were performed. The findings therefore represent a single-run snapshot rather than stable average model performance. Fifth, although model names and platform identifiers were removed and responses were randomized, complete blinding was not possible because raters may have recognized models from response length, formatting, tone, or structure. Sixth, the pragmatic sample size meant that the effective paired unit was 44 questions, limiting power to detect small differences, particularly for the binary safety outcome with only 20 unsafe responses. Seventh, despite structured scoring rubrics, metrics such as Accuracy, Empathy, DISCERN, EQIP, JAMA, and GQS have inherent limitations. Eighth, readability formulas quantify surface-level text complexity and do not directly measure caregivers' comprehension, cultural appropriateness, digital literacy, or ease of use. Finally, this study evaluated English-language responses only and did not perform multilingual comparisons.

## Conclusions

In this single-run comparative study, large language model-driven chatbots showed statistically significant differences in answering pediatric vitamin D deficiency-related questions. ChatGPT demonstrated favorable performance in accuracy and information quality, Doubao and DeepSeek showed advantages in empathy, and Gemini achieved relatively favorable FRES readability despite lower scores in several quality domains. However, these results should be interpreted as an exploratory, time-specific snapshot rather than definitive evidence of stable model superiority. The findings indicate that medical correctness alone is insufficient for evaluating chatbot health advice. Pediatric applications should be assessed for safety, accuracy, empathy, reliability, transparency, readability, reproducibility, and clinician escalation, and high-risk scenarios should continue to require professional medical evaluation.

## Data Availability

The original contributions presented in the study are included in the article/Supplementary Material, further inquiries can be directed to the corresponding author/s.
