# Supplementary material for: Cross-sectional comparative evaluation of five large language model–driven chatbots on an expert-curated 44-question set of parent-facing questions about pediatric vitamin D deficiency
Source: Front Pediatr. 2026 Sep 9;14:1927624. doi: 10.3389/fped.2026.1927624 (PMC13597792; doi:10.3389/fped.2026.1927624)
Supplement: Supplementary file 1 [file Supplementaryfile1.docx]

Table S1. Question inclusion and exclusion record

Performance of Large Language Models in Answering Questions About Pediatric Vitamin D Deficiency

**Screening summary:** A 92-question candidate pool was screened for a 44-question core evaluation set. Inclusion prioritized clinical relevance, caregiver frequency, pediatric dosing and diagnostic value, special risk groups, toxicity, and urgent referral safety. Exclusion was mainly due to redundancy, narrow product-specific scope, lower clinical priority, or specialist-only detail.

| **Candidate ID** | **Domain** | **Candidate Question** | **Decision** | **Final ID** | **Reason for Inclusion or Exclusion** |
| --- | --- | --- | --- | --- | --- |
| 1 | Basic concepts and definitions | What is pediatric vitamin D deficiency? | Include | 1 | Included: foundational concept for answer accuracy. |
| 2 | Basic concepts and definitions | What is the main role of vitamin D in children's growth and development? | Include | 2 | Included: foundational concept for answer accuracy. |
| 3 | Basic concepts and definitions | Are vitamin D deficiency and rickets the same condition? | Include | 3 | Included: foundational concept for answer accuracy. |
| 4 | Basic concepts and definitions | What is the difference between vitamin D insufficiency, deficiency, and severe deficiency? | Exclude | - | Excluded: concept overlaps with the retained item on 25-hydroxyvitamin D interpretation and deficiency thresholds. |
| 5 | Basic concepts and definitions | What does 25-hydroxyvitamin D mean, and why is it commonly used to assess vitamin D status? | Include | 4 | Included: foundational concept for answer accuracy. |
| 6 | Basic concepts and definitions | How are vitamin D, calcium, phosphorus, and children's bone development related? | Include | 5 | Included: foundational concept for answer accuracy. |
| 7 | Basic concepts and definitions | Does vitamin D deficiency in children affect only the bones, or can it also affect muscles or immunity? | Exclude | - | Excluded: lower priority for the 44-item core set; broader non-skeletal effects are less central to deficiency management. |
| 8 | Basic concepts and definitions | Is pediatric vitamin D deficiency a common problem or a rare disease? | Exclude | - | Excluded: background epidemiology item; less useful for testing clinical answer quality than diagnosis and management items. |
| 9 | Risk factors and susceptible groups | Which children are more likely to develop vitamin D deficiency? | Include | 6 | Included: high-priority pediatric risk factor. |
| 10 | Risk factors and susceptible groups | Why are exclusively breastfed infants more likely to develop vitamin D deficiency? | Include | 7 | Included: high-priority pediatric risk factor. |
| 11 | Risk factors and susceptible groups | Can formula-fed infants still develop vitamin D deficiency? | Include | 8 | Included: high-priority pediatric risk factor. |
| 12 | Risk factors and susceptible groups | Why do preterm or low-birth-weight infants require special attention to vitamin D? | Include | 9 | Included: high-priority pediatric risk factor. |
| 13 | Risk factors and susceptible groups | Why are children with darker skin at higher risk of vitamin D deficiency? | Include | 10 | Included: high-priority pediatric risk factor. |
| 14 | Risk factors and susceptible groups | Can children who rarely go outdoors or have little outdoor activity develop vitamin D deficiency? | Exclude | - | Excluded: overlaps with retained questions on dark skin, winter/northern regions, and sunlight exposure. |

| **Candidate ID** | **Domain** | **Candidate Question** | **Decision** | **Final ID** | **Reason for Inclusion or Exclusion** |
| --- | --- | --- | --- | --- | --- |
| 15 | Risk factors and susceptible groups | Do northern regions, winter, or heavy air pollution increase the risk of vitamin D deficiency in children? | Include | 11 | Included: high-priority pediatric risk factor. |
| 16 | Risk factors and susceptible groups | Why may children with obesity be more likely to have vitamin D insufficiency? | Exclude | - | Excluded: obesity is represented indirectly within high-risk groups; removed to preserve balance across domains. |
| 17 | Risk factors and susceptible groups | Why are children with liver disease, kidney disease, or intestinal malabsorption more likely to develop vitamin D deficiency? | Include | 12 | Included: high-priority pediatric risk factor. |
| 18 | Risk factors and susceptible groups | Should children taking long-term antiepileptic drugs, glucocorticoids, or similar medications be monitored for vitamin D problems? | Include | 13 | Included: high-priority pediatric risk factor. |
| 19 | Symptoms, signs, and parental recognition | What are the early signs of vitamin D deficiency in children? | Include | 14 | Included: common caregiver concern or key clinical sign. |
| 20 | Symptoms, signs, and parental recognition | Do poor sleep, excessive sweating, or hair loss on the back of the head mean that a baby has vitamin D deficiency? | Include | 15 | Included: common caregiver concern or key clinical sign. |
| 21 | Symptoms, signs, and parental recognition | Do delayed tooth eruption or delayed closure of the fontanelle suggest vitamin D deficiency? | Include | 16 | Included: common caregiver concern or key clinical sign. |
| 22 | Symptoms, signs, and parental recognition | Are bowed legs or knock knees signs of vitamin D deficiency or rickets? | Include | 17 | Included: common caregiver concern or key clinical sign. |
| 23 | Symptoms, signs, and parental recognition | Are flared ribs or pigeon chest related to vitamin D deficiency? | Include | 18 | Included: common caregiver concern or key clinical sign. |
| 24 | Symptoms, signs, and parental recognition | Could frequent leg pain, muscle weakness, or fatigue in a child be related to vitamin D deficiency? | Include | 19 | Included: common caregiver concern or key clinical sign. |
| 25 | Symptoms, signs, and parental recognition | Can vitamin D deficiency affect a child's height growth? | Include | 20 | Included: common caregiver concern or key clinical sign. |
| 26 | Symptoms, signs, and parental recognition | Can irritability or delayed motor development in a baby be related to vitamin D deficiency? | Exclude | - | Excluded: overlaps with early signs and developmental/bone manifestations covered by retained symptom questions. |
| 27 | Symptoms, signs, and parental recognition | Should vitamin D deficiency be investigated in a child with recurrent fractures? | Include | 21 | Included: common caregiver concern or key clinical sign. |
| 28 | Symptoms, signs, and parental recognition | What signs suggest that a child may have rickets rather than simple vitamin D deficiency? | Include | 22 | Included: common caregiver concern or key clinical sign. |

| **Candidate ID** | **Domain** | **Candidate Question** | **Decision** | **Final ID** | **Reason for Inclusion or Exclusion** |
| --- | --- | --- | --- | --- | --- |
| 29 | Testing, diagnosis, and interpretation | Which children need a 25-hydroxyvitamin D test? | Include | 23 | Included: core diagnostic or monitoring item. |
| 30 | Testing, diagnosis, and interpretation | Should healthy children be routinely screened for vitamin D levels? | Include | 24 | Included: core diagnostic or monitoring item. |
| 31 | Testing, diagnosis, and interpretation | Is measuring 25-hydroxyvitamin D alone enough to assess vitamin D status? | Exclude | - | Excluded: overlaps with retained questions on who needs testing and which accompanying markers should be checked. |
| 32 | Testing, diagnosis, and interpretation | What 25-hydroxyvitamin D level is considered deficient, insufficient, or normal? | Include | 25 | Included: core diagnostic or monitoring item. |
| 33 | Testing, diagnosis, and interpretation | How do you convert vitamin D test results between ng/mL and nmol/L? | Exclude | - | Excluded: useful but too technical for the final parent-facing core set. |
| 34 | Testing, diagnosis, and interpretation | When vitamin D is low, should calcium, phosphorus, alkaline phosphatase, or parathyroid hormone also be checked? | Include | 26 | Included: core diagnostic or monitoring item. |
| 35 | Testing, diagnosis, and interpretation | Why might an X-ray be needed when rickets is suspected? | Include | 27 | Included: core diagnostic or monitoring item. |
| 36 | Testing, diagnosis, and interpretation | How should parents interpret a low vitamin D blood test result if the child has no symptoms? | Include | 28 | Included: core diagnostic or monitoring item. |
| 37 | Testing, diagnosis, and interpretation | Does a normal test result mean that a child does not need vitamin D supplementation? | Exclude | - | Excluded: overlaps with retained prevention and supplementation questions, especially routine supplementation by age and feeding pattern. |
| 38 | Testing, diagnosis, and interpretation | After treatment for vitamin D deficiency, when should vitamin D or related laboratory markers be rechecked? | Include | 29 | Included: core diagnostic or monitoring item. |
| 39 | Prevention and supplementation dosage | When should newborns start vitamin D supplementation? | Include | 30 | Included: core supplementation and dosing item. |
| 40 | Prevention and supplementation dosage | How much vitamin D does an infant aged 0-12 months need each day? | Include | 31 | Included: core supplementation and dosing item. |
| 41 | Prevention and supplementation dosage | How much vitamin D does a child older than 1 year need each day? | Include | 32 | Included: core supplementation and dosing item. |
| 42 | Prevention and supplementation dosage | Do adolescents still need vitamin D supplementation? | Exclude | - | Excluded: adolescent needs are partly covered by the retained question for children older than 1 year. |

| **Candidate ID** | **Domain** | **Candidate Question** | **Decision** | **Final ID** | **Reason for Inclusion or Exclusion** |
| --- | --- | --- | --- | --- | --- |
| 43 | Prevention and supplementation dosage | Until what age should children continue vitamin D supplementation? | Include | 33 | Included: core supplementation and dosing item. |
| 44 | Prevention and supplementation dosage | How should an exclusively breastfed infant receive vitamin D supplementation? | Include | 34 | Included: core supplementation and dosing item. |
| 45 | Prevention and supplementation dosage | How should vitamin D intake be calculated for a mixed-fed infant? | Include | 35 | Included: core supplementation and dosing item. |
| 46 | Prevention and supplementation dosage | Does an infant who drinks enough formula still need extra vitamin D supplementation? | Include | 36 | Included: core supplementation and dosing item. |
| 47 | Prevention and supplementation dosage | If a child misses vitamin D for one day or several days, should the missed doses be made up? | Exclude | - | Excluded: practical but lower risk than dose, deficiency treatment, toxicity, and urgent symptoms. |
| 48 | Prevention and supplementation dosage | How should parents choose among vitamin D drops, capsules, chewable tablets, or gummies? | Exclude | - | Excluded: product-format selection is less central than dose, timing of initiation, and safety. |
| 49 | Prevention and supplementation dosage | What is the difference between vitamin D2 and vitamin D3, and which is more suitable for children? | Include | 37 | Included: core supplementation and dosing item. |
| 50 | Prevention and supplementation dosage | Should vitamin D be taken in the morning or evening, and before or after meals? | Exclude | - | Excluded: administration timing is lower priority for model safety assessment. |
| 51 | Treatment and follow-up | If vitamin D deficiency has already been diagnosed, is a preventive dose still enough? | Include | 38 | Included: high-priority treatment or follow-up item. |
| 52 | Treatment and follow-up | How does management differ for mild, moderate, and severe vitamin D deficiency? | Exclude | - | Excluded: overlaps with the retained question on whether preventive dosing is sufficient after diagnosis and the retained high-dose treatment question. |
| 53 | Treatment and follow-up | How long does treatment for pediatric vitamin D deficiency usually take? | Exclude | - | Excluded: overlaps with retained follow-up and treatment-response questions. |
| 54 | Treatment and follow-up | Is high-dose vitamin D prescribed by a doctor safe for children? | Exclude | - | Excluded: overlaps with the retained high-dose and one-time mega-dose treatment-safety questions. |
| 55 | Treatment and follow-up | Can a one-time large dose of vitamin D be used to correct deficiency quickly? | Include | 39 | Included: high-priority treatment or follow-up item. |
| 56 | Treatment and follow-up | Is calcium supplementation needed when treating vitamin D deficiency? | Include | 40 | Included: high-priority treatment or follow-up item. |

| **Candidate ID** | **Domain** | **Candidate Question** | **Decision** | **Final ID** | **Reason for Inclusion or Exclusion** |
| --- | --- | --- | --- | --- | --- |
| 57 | Treatment and follow-up | How soon do symptoms improve after vitamin D treatment? | Exclude | - | Excluded: lower priority than non-response, calcium co-treatment, and urgent safety issues. |
| 58 | Treatment and follow-up | Why might a child's vitamin D level remain low after supplementation? | Include | 41 | Included: high-priority treatment or follow-up item. |
| 59 | Treatment and follow-up | Can bone deformities caused by rickets recover? | Exclude | - | Excluded: overlaps with retained questions on rickets signs and musculoskeletal deformities. |
| 60 | Treatment and follow-up | After vitamin D deficiency treatment is completed, is a maintenance dose still needed? | Exclude | - | Excluded: overlaps with retained questions on continuing supplementation by age and treatment follow-up. |
| 61 | Diet, sunlight, and lifestyle | Can sunlight exposure replace vitamin D supplementation? | Include | 42 | Included: key lifestyle question relevant to supplementation decisions. |
| 62 | Diet, sunlight, and lifestyle | Can infants obtain vitamin D through sunlight exposure, and can babies younger than 6 months be exposed to direct sunlight? | Exclude | - | Excluded: overlaps with the retained question on whether sunlight can replace supplementation. |
| 63 | Diet, sunlight, and lifestyle | How long should children spend in the sun each day to get enough vitamin D? | Exclude | - | Excluded: highly variable by latitude, season, skin exposure, and skin type; covered by retained sunlight question. |
| 64 | Diet, sunlight, and lifestyle | Do sunscreen and clothing coverage affect vitamin D production in the skin? | Exclude | - | Excluded: overlaps with retained sunlight and risk-factor questions. |
| 65 | Diet, sunlight, and lifestyle | Which foods are rich in vitamin D? | Exclude | - | Excluded: diet is useful but lower priority than supplementation dose and clinical safety for this 44-item instrument. |
| 66 | Diet, sunlight, and lifestyle | Can egg yolks, milk, and fish meet a child's daily vitamin D requirement? | Exclude | - | Excluded: overlaps with retained sunlight/supplementation adequacy questions and is lower priority than treatment safety. |
| 67 | Diet, sunlight, and lifestyle | How can vegetarian children obtain enough vitamin D? | Exclude | - | Excluded: special dietary scenario removed to preserve balance across more clinically consequential domains. |
| 68 | Diet, sunlight, and lifestyle | How can a child who does not like milk maintain adequate calcium and vitamin D intake? | Exclude | - | Excluded: calcium and vitamin D intake is partly covered by retained calcium co-treatment and supplementation questions. |
| 69 | Safety, overdose, and interactions | Can too much vitamin D cause toxicity? | Include | 43 | Included: high-priority toxicity and safety item. |
| 70 | Safety, overdose, and interactions | What are the symptoms of vitamin D overdose in children? | Exclude | - | Excluded: overlaps with retained general toxicity question. |

| **Candidate ID** | **Domain** | **Candidate Question** | **Decision** | **Final ID** | **Reason for Inclusion or Exclusion** |
| --- | --- | --- | --- | --- | --- |
| 71 | Safety, overdose, and interactions | What should parents do if a child accidentally takes too many drops of vitamin D? | Exclude | - | Excluded: practical overdose handling item, but the final set keeps one broader toxicity item and one urgent emergency item. |
| 72 | Safety, overdose, and interactions | Can taking multiple supplements at the same time lead to duplicate vitamin D intake? | Exclude | - | Excluded: duplicate intake is captured within the retained toxicity and high-dose-safety items. |
| 73 | Safety, overdose, and interactions | Can cod liver oil and vitamin D drops be taken together? | Exclude | - | Excluded: product-specific interaction issue; lower priority for the core instrument. |
| 74 | Safety, overdose, and interactions | Can taking vitamin D and calcium together increase the risk of kidney stones? | Exclude | - | Excluded: narrower safety question; calcium co-treatment and vitamin D toxicity are retained. |
| 75 | Safety, overdose, and interactions | Must children take vitamin K2 together with vitamin D? | Exclude | - | Excluded: common misconception but less central than dose, deficiency, treatment, and urgent symptoms. |
| 76 | Safety, overdose, and interactions | Is it safe for children to take more than the recommended amount of vitamin D every day for a long time? | Exclude | - | Excluded: overlaps with retained toxicity and high-dose treatment-safety questions. |
| 77 | Special pediatric scenarios | How does vitamin D supplementation for preterm infants differ from that for term infants? | Exclude | - | Excluded: overlaps with the retained preterm/low-birth-weight risk question. |
| 78 | Special pediatric scenarios | Do low-birth-weight infants or twins need a higher dose of vitamin D? | Exclude | - | Excluded: overlaps with the retained preterm/low-birth-weight risk question. |
| 79 | Special pediatric scenarios | How can children with cow's milk protein allergy or lactose intolerance maintain calcium and vitamin D intake? | Exclude | - | Excluded: special dietary scenario removed to preserve balance across diagnosis, treatment, and safety domains. |
| 80 | Special pediatric scenarios | Should the vitamin D dose be adjusted for children with obesity? | Exclude | - | Excluded: obesity-specific management removed; obesity risk is not a primary focus of the 44-item parent-facing core set. |
| 81 | Special pediatric scenarios | Can children with chronic kidney disease supplement vitamin D in the same way as healthy children? | Exclude | - | Excluded: chronic kidney disease is a specialist scenario and partly covered by retained chronic disease risk question. |
| 82 | Special pediatric scenarios | How should children with liver or biliary disease, including cholestasis, receive vitamin D? | Exclude | - | Excluded: hepatobiliary disease is a specialist scenario and partly covered by retained chronic disease risk question. |
| 83 | Special pediatric scenarios | Why are children with celiac disease, inflammatory bowel disease, or cystic fibrosis more likely to develop vitamin D deficiency? | Exclude | - | Excluded: malabsorption risk is covered by the retained chronic disease and malabsorption question. |
| 84 | Special pediatric scenarios | How should vitamin D and bone health be monitored in children with epilepsy who take long-term medication? | Exclude | - | Excluded: long-term antiepileptic medication risk is covered by the retained medication-related risk question. |

| **Candidate ID** | **Domain** | **Candidate Question** | **Decision** | **Final ID** | **Reason for Inclusion or Exclusion** |
| --- | --- | --- | --- | --- | --- |
| 85 | Common misconceptions and information appraisal | What is the difference between calcium deficiency and vitamin D deficiency? | Exclude | - | Excluded: overlaps with retained vitamin D/calcium/phosphorus relationship and calcium co-treatment questions. |
| 86 | Common misconceptions and information appraisal | Does slow growth always mean that a child has vitamin D deficiency? | Exclude | - | Excluded: overlaps with the retained question on height growth and vitamin D deficiency. |
| 87 | Common misconceptions and information appraisal | Is more vitamin D always better? | Exclude | - | Excluded: overlaps with retained toxicity and high-dose-safety questions. |
| 88 | Common misconceptions and information appraisal | If children spend more time outdoors in summer, can vitamin D supplementation be stopped? | Exclude | - | Excluded: overlaps with retained sunlight and supplementation-by-age questions. |
| 89 | Common misconceptions and information appraisal | Are imported vitamin D products with higher concentrations necessarily more effective? | Exclude | - | Excluded: product-specific misconception; lower priority for the 44-item scientific evaluation set. |
| 90 | When to seek medical care and emergency handling | If a child has seizures, stridor, or carpopedal spasms, could this be related to vitamin D deficiency or hypocalcemia, and what should parents do? | Include | 44 | Included: urgent referral and emergency safety item. |
| 91 | When to seek medical care and emergency handling | If a child has obvious bowed legs, knock knees, or recurrent bone pain, should the family see a pediatrician, orthopedist, or pediatric endocrinologist? | Exclude | - | Excluded: overlaps with retained questions on bowed legs/knock knees and rickets signs. |
| 92 | When to seek medical care and emergency handling | What information should parents prepare before consulting a doctor about pediatric vitamin D deficiency? | Exclude | - | Excluded: useful consultation-preparation item but less useful for testing biomedical accuracy and safety. |

**Note:** This record documents instrument construction rather than clinical recommendations. Final answer scoring should be conducted against predefined reference standards and independent expert review.

Table S2. Clean 44-question set

Pediatric Vitamin D Deficiency LLM Evaluation Study

**Suggested standardized prompt before each question:** Please answer in plain language for parents or caregivers, and clearly state when medical evaluation or urgent care is needed.

| **Item No.** | **Domain** | **Question** |
| --- | --- | --- |
| 1 | Basic concepts and definitions | What is pediatric vitamin D deficiency? |
| 2 | Basic concepts and definitions | What is the main role of vitamin D in children's growth and development? |
| 3 | Basic concepts and definitions | Are vitamin D deficiency and rickets the same condition? |
| 4 | Basic concepts and definitions | What does 25-hydroxyvitamin D mean? |
| 5 | Basic concepts and definitions | How are vitamin D, calcium, phosphorus, and children's bone development related? |
| 6 | Risk factors and susceptible groups | Which children are more likely to develop vitamin D deficiency? |
| 7 | Risk factors and susceptible groups | Why are exclusively breastfed infants more likely to develop vitamin D deficiency? |
| 8 | Risk factors and susceptible groups | Can formula-fed infants still develop vitamin D deficiency? |
| 9 | Risk factors and susceptible groups | Why do preterm or low-birth-weight infants require special attention to vitamin D? |
| 10 | Risk factors and susceptible groups | Why are children with darker skin at higher risk of vitamin D deficiency? |
| 11 | Risk factors and susceptible groups | Do northern regions, winter, or heavy air pollution increase the risk of vitamin D deficiency in children? |
| 12 | Risk factors and susceptible groups | Why are children with liver disease, kidney disease, or intestinal malabsorption more likely to develop vitamin D deficiency? |
| 13 | Risk factors and susceptible groups | Should children taking long-term antiepileptic drugs, glucocorticoids, or similar medications be monitored for vitamin D problems? |
| 14 | Symptoms, signs, and parental recognition | What are the early signs of vitamin D deficiency in children? |
| 15 | Symptoms, signs, and parental recognition | Do poor sleep, excessive sweating, or hair loss on the back of the head mean that a baby has vitamin D deficiency? |
| 16 | Symptoms, signs, and parental recognition | Do delayed tooth eruption or delayed closure of the fontanelle suggest vitamin D deficiency? |
| 17 | Symptoms, signs, and parental recognition | Are bowed legs or knock knees signs of vitamin D deficiency or rickets? |
| 18 | Symptoms, signs, and parental recognition | Are flared ribs or pigeon chest related to vitamin D deficiency? |
| 19 | Symptoms, signs, and parental recognition | Could frequent leg pain, muscle weakness, or fatigue in a child be related to vitamin D deficiency? |
| 20 | Symptoms, signs, and parental recognition | Can vitamin D deficiency affect a child's height growth? |
| 21 | Symptoms, signs, and parental recognition | Should vitamin D deficiency be investigated in a child with recurrent fractures? |
| 22 | Symptoms, signs, and parental recognition | What signs suggest that a child may have rickets rather than simple vitamin D deficiency? |

| **Item No.** | **Domain** | **Question** |
| --- | --- | --- |
| 23 | Testing, diagnosis, and interpretation | Which children need a 25-hydroxyvitamin D test? |
| 24 | Testing, diagnosis, and interpretation | Should healthy children be routinely screened for vitamin D levels? |
| 25 | Testing, diagnosis, and interpretation | What 25-hydroxyvitamin D level is considered deficient, insufficient, or normal? |
| 26 | Testing, diagnosis, and interpretation | When vitamin D is low, should calcium, phosphorus, alkaline phosphatase, or parathyroid hormone also be checked? |
| 27 | Testing, diagnosis, and interpretation | Why might an X-ray be needed when rickets is suspected? |
| 28 | Testing, diagnosis, and interpretation | How should parents interpret a low vitamin D blood test result if the child has no symptoms? |
| 29 | Testing, diagnosis, and interpretation | After treatment for vitamin D deficiency, when should vitamin D or related laboratory markers be rechecked? |
| 30 | Prevention and supplementation dosage | When should newborns start vitamin D supplementation? |
| 31 | Prevention and supplementation dosage | How much vitamin D does an infant aged 0-12 months need each day? |
| 32 | Prevention and supplementation dosage | How much vitamin D does a child older than 1 year need each day? |
| 33 | Prevention and supplementation dosage | Until what age should children continue vitamin D supplementation? |
| 34 | Prevention and supplementation dosage | How should an exclusively breastfed infant receive vitamin D supplementation? |
| 35 | Prevention and supplementation dosage | How should vitamin D intake be calculated for a mixed-fed infant? |
| 36 | Prevention and supplementation dosage | Does an infant who drinks enough formula still need extra vitamin D supplementation? |
| 37 | Prevention and supplementation dosage | What is the difference between vitamin D2 and vitamin D3? |
| 38 | Treatment and follow-up | If vitamin D deficiency has already been diagnosed, is a preventive dose still enough? |
| 39 | Treatment and follow-up | Can a one-time large dose of vitamin D be used to correct deficiency quickly? |
| 40 | Treatment and follow-up | Is calcium supplementation needed when treating vitamin D deficiency? |
| 41 | Treatment and follow-up | Why might a child's vitamin D level remain low after supplementation? |
| 42 | Diet, sunlight, and lifestyle | Can sunlight exposure replace vitamin D supplementation? |
| 43 | Safety, overdose, and interactions | Can too much vitamin D cause toxicity? |
| 44 | When to seek medical care and emergency handling | If a child has seizures, stridor, or carpopedal spasms, could this be related to vitamin D deficiency or hypocalcemia, and what should parents do? |

**Table S3. Characteristics of the large language model-driven chatbot services evaluated in this study**

| **Chatbot service** | **Provider** | **Model/version evaluated** | **Release/update information** | **Source availability** | **Study implementation** |
| --- | --- | --- | --- | --- | --- |
| ChatGPT | OpenAI | GPT-5.5 | Released in April 2026 according to OpenAI release documentation. | Proprietary / closed-source; model weights and training data were not accessed. | Accessed through the standard ChatGPT user interface. Default settings were used. Each of the 44 questions was entered in a separate new chat session during May 10–15, 2026 in Suzhou, Jiangsu, China. |
| Gemini | Google | Gemini 3.1 Pro | Launched in February 2026 according to Google release documentation. | Proprietary / closed-source; model weights and training data were not accessed. | Accessed through the standard Gemini user interface. Default settings were used. Each of the 44 questions was entered in a separate new chat session during May 10–15, 2026 in Suzhou, Jiangsu, China. |
| Qianwen / Qwen | Alibaba Cloud | Qianwen3.6-Plus | Released in April 2026 as part of Alibaba’s Qwen large language model series. | Proprietary service as accessed in this study; model weights and training data were not accessed. | Accessed through the standard Qianwen/Qwen user interface. Default settings were used. Each of the 44 questions was entered in a separate new chat session during May 10–15, 2026 in Suzhou, Jiangsu, China. |
| DeepSeek | DeepSeek | DeepSeek V4 | DeepSeek-V4 Preview was released on April 24, 2026 according to DeepSeek documentation. | Open-weight model family according to documentation; this study evaluated the public chatbot service, not a local deployment. | Accessed through the standard DeepSeek user interface. Default settings were used. Each of the 44 questions was entered in a separate new chat session during May 10–15, 2026 in Suzhou, Jiangsu, China. |
| Doubao | ByteDance | Doubao-Seed-2.0 Pro | The study record indicated an update to Doubao-Seed-2.0 Pro on May 15, 2026; because this coincided with the end of the data-collection window, the results should be interpreted as time-specific. | Proprietary service as accessed in this study; model weights and training data were not accessed. | Accessed through the standard Doubao user interface. Default settings were used. Each of the 44 questions was entered in a separate new chat session during May 10–15, 2026 in Suzhou, Jiangsu, China. |
|  |  |  |  |  |  |
|  | | | | | |

The 44 retained questions should be interpreted as an expert-curated, caregiver-source-informed question set rather than as a formally parent-validated instrument, because no parents or caregivers were directly involved in face-validity testing.

Source review, candidate question identification, and question screening were completed before model querying, which took place from May 10 to May 15, 2026. Exact dates for individual Google Trends, Baidu Index, and forum searches were not prospectively logged; therefore, search frequency was used qualitatively to inform the candidate pool rather than as a strict numerical threshold.

Table S4. Source strategy for question development

| **Source category** | **Platforms/sources considered** | **Search terms or source logic** | **Screening and selection rule** |
| --- | --- | --- | --- |
| Search trends | Google Trends and Baidu Index | vitamin D deficiency in children; pediatric vitamin D; rickets in children; infant vitamin D supplementation; vitamin D test children; vitamin D toxicity children; 维生素D缺乏 儿童; 佝偻病; 婴儿 维生素D补充; 儿童 维生素D检测 | Terms were used to identify parent-facing topics with recurring search interest. Frequency data were used qualitatively to inform the candidate pool rather than as a strict numerical threshold. |
| Online forums and caregiver communities | Parent-facing online Q&A platforms, health forums, and caregiver communities | Questions were screened for parent-oriented wording, practical decision relevance, and recurrence across caregiver-facing sources. | Product-specific, highly redundant, adult-focused, or specialist-only questions were excluded. |
| Clinical guidelines and professional materials | Pediatric vitamin D, rickets, infant feeding, testing, supplementation, toxicity, and high-risk-group guidance | Guideline sections concerning prevention, diagnosis, treatment, monitoring, toxicity, emergency symptoms, and special risk groups were mapped to candidate questions. | Questions were retained when they tested clinically actionable or safety-critical content that could influence caregiver decisions. |
| Expert discussion | Pediatrics, child health care, and nutrition specialists | Experts reviewed the candidate pool for clinical relevance, safety implications, and balance across domains. | Inclusion prioritized diagnostic value, dosing relevance, urgent referral safety, high-risk groups, and avoidance of redundant questions. |

Table S5. Distribution of retained questions by domain

| **Domain** | **Retained questions, n** |
| --- | --- |
| Basic concepts and definitions | 5 |
| Risk factors and susceptible groups | 8 |
| Symptoms, signs, and parental recognition | 9 |
| Testing, diagnosis, and interpretation | 7 |
| Prevention and supplementation dosage | 8 |
| Treatment and follow-up | 4 |
| Diet, sunlight, and lifestyle | 1 |
| Safety, overdose, and interactions | 1 |
| When to seek medical care and emergency handling | 1 |
| Total | 44 |

**English wording and adaptation procedure**

Candidate questions were harmonized into plain English parent-facing wording before model querying. Translation and adaptation aimed to preserve clinical intent rather than literal phrasing. Clinically ambiguous or overly technical wording was simplified, while safety-critical elements such as high-dose supplementation, toxicity, rickets, hypocalcemic symptoms, and urgent medical evaluation were retained. The final wording was reviewed against the guideline-mapped reference framework before chatbot querying.

Table S6. Model and query-environment reporting

| **Chatbot service** | **Displayed model/service identifier** | Access route, subscription tier, and settings | **Query period and environment** | **Limitations recorded** |
| --- | --- | --- | --- | --- |
| ChatGPT | ChatGPT-5.5 as displayed/reported by the study team | Standard user interface; default settings; new chat session for each question; no follow-up prompts; browsing or retrieval-augmented mode not intentionally used. Subscription tier was not independently verified. | May 10-15, 2026; Suzhou, Jiangsu, China; English prompts. | Automatic routing, hidden system prompts, temperature, and backend routing could not be independently verified. Screenshots or platform logs were not available in the submitted study record for independent verification. |
| Gemini | Gemini 3.1 Pro as displayed/reported by the study team | Standard user interface; default settings; new chat session for each question; no follow-up prompts. Subscription tier was not independently verified. | May 10-15, 2026; Suzhou, Jiangsu, China; English prompts. | Undocumented platform settings and backend routing were not accessible to the authors. Screenshots or platform logs were not available in the submitted study record for independent verification. |
| Qianwen | Qianwen 3.6-Plus as displayed/reported by the study team | Standard user interface; default settings; new chat session for each question; no follow-up prompts. Subscription tier was not independently verified. | May 10-15, 2026; Suzhou, Jiangsu, China; English prompts. | Internal model parameters and routing mechanisms were not accessible. Screenshots or platform logs were not available in the submitted study record for independent verification. |
| DeepSeek | DeepSeek V4 as displayed/reported by the study team | Standard user interface; default settings; new chat session for each question; no follow-up prompts. Subscription tier was not independently verified. | May 10-15, 2026; Suzhou, Jiangsu, China; English prompts. | The available source record did not independently confirm whether the interface corresponded to a Pro, Flash, or routed variant; this uncertainty is acknowledged. Screenshots or platform logs were not available in the submitted study record for independent verification. |
| Doubao | Doubao-Seed-2.0 Pro as displayed/reported by the study team | Standard user interface; default settings; new chat session for each question; no follow-up prompts. Subscription tier was not independently verified. | May 10-15, 2026; Suzhou, Jiangsu, China; English prompts. | Because the reported update coincided with the end of the data-collection window, the results should be treated as time-specific. Screenshots or platform logs were not available in the submitted study record for independent verification. |

**Note:** Exact model-specific clock times were not prospectively logged; only the overall query window of May 10–15, 2026 was retained. Memory and personalization status were not prospectively recorded and could not be independently verified. These unavailable settings were not retrospectively inferred.
