# Supplementary material for: Cross-sectional comparative evaluation of five large language model–driven chatbots on an expert-curated 44-question set of parent-facing questions about pediatric vitamin D deficiency
Source: Front Pediatr. 2026 Sep 9;14:1927624. doi: 10.3389/fped.2026.1927624 (PMC13597792; doi:10.3389/fped.2026.1927624)
Supplement: Supplementary file 2 [file Supplementaryfile2.docx]

## Question-Answer-Guideline Mapping Table (with Source Column)

**Pediatric Vitamin D Deficiency LLM Evaluation Study**

### Source Key

| **Source tag** | **Reference** |
| --- | --- |
| **IAP 2021/2022** | Gupta P, Dabas A, Seth A, et al. Indian Academy of Pediatrics Revised (2021) Guidelines on Prevention and Treatment of Vitamin D Deficiency and Rickets. Indian Pediatrics. 2022;59:142-158. |
| **French 2022** | Bacchetta J, Edouard T, Laverny G, et al. Vitamin D and calcium intakes in general pediatric populations: A French expert consensus paper. Archives de Pediatrie. 2022;29:312-325. |
| **SIEDP 2024** | Baroncelli GI, Comberiati P, Aversa T, et al. Diagnosis, treatment, and management of rickets: a position statement from the Bone and Mineral Metabolism Group of the Italian Society of Pediatric Endocrinology and Diabetology. Frontiers in Endocrinology. 2024;15:1383681. |
| **CDC 2025/2026** | Centers for Disease Control and Prevention. Vitamin D guidance for infant/toddler nutrition and breastfeeding. Updated 2025-2026. |
| **AAP 2022** | American Academy of Pediatrics / HealthyChildren.org. Vitamin D for babies, children, and adolescents. Updated 2022. |
| **NIH ODS 2025** | National Institutes of Health Office of Dietary Supplements. Vitamin D: Health Professional Fact Sheet. Updated 2025. |
| **CKD-MBD 2025** | Jorgensen HS, Vervloet M, Cavalier E, et al. The role of nutritional vitamin D in CKD-MBD in children and adults with CKD, on dialysis and after kidney transplantation: a European consensus statement. Nephrology Dialysis Transplantation. 2025. |
| **Testing 2023** | Zurynski Y, Munns CF, Sezgin G, et al. Vitamin D testing in children and adolescents in Victoria, Australia: are testing practices in line with global recommendations? Archives of Disease in Childhood. 2023;108:742-747. |
| **ASM 2023** | Ragunathan K, Chakrabarty B. Vitamin D Supplementation in Children on Antiseizure Medications: High Time to Have Proper Guidelines. Indian Journal of Pediatrics. 2023. |

### Question-Answer Mapping with Source Column

| **No.** | **Question** | **Concise benchmark answer** | **Guideline-mapped content / scoring anchor** | **Source(s)** |
| --- | --- | --- | --- | --- |
| 1 | What is pediatric vitamin D deficiency? | A low vitamin D status in a child, usually assessed by serum 25-hydroxyvitamin D [25(OH)D]. Cutoffs differ across guidelines; one commonly used pediatric cutoff defines deficiency as <12 ng/mL. | Define it as biochemical low vitamin D status; note that 25(OH)D is the usual marker; avoid implying that every low result means rickets. IAP uses <12 ng/mL for deficiency, 12-20 ng/mL for insufficiency, and >20 ng/mL for sufficiency. [IAP 2021/2022; NIH ODS 2025] | IAP 2021/2022; NIH ODS 2025 |
| 2 | What is the main role of vitamin D in children's growth and development? | Vitamin D supports calcium and phosphate balance, bone mineralization, and normal skeletal growth; severe deficiency can lead to rickets and hypocalcemic symptoms. | Emphasize skeletal/mineral functions first. Extraskeletal roles may be mentioned cautiously, but should not be overstated as proven indications for routine supplementation. [French 2022; SIEDP 2024; NIH ODS 2025] | French 2022; SIEDP 2024; NIH ODS 2025 |
| 3 | Are vitamin D deficiency and rickets the same condition? | No. Vitamin D deficiency is a nutrient/status problem; rickets is a bone disease caused by impaired growth-plate mineralization, which may result from vitamin D deficiency, calcium deficiency, phosphate problems, or genetic disorders. | Separate biochemical deficiency from established skeletal disease and mention that rickets has nutritional and non-nutritional causes. [SIEDP 2024; IAP 2021/2022] | SIEDP 2024; IAP 2021/2022 |
| 4 | What does 25-hydroxyvitamin D mean? | 25-hydroxyvitamin D, or 25(OH)D, is the main circulating vitamin D metabolite and is the standard blood marker used to estimate vitamin D stores. | Do not use 1,25(OH)2D as the routine status marker. 1,25(OH)2D may be normal or high in deficiency and is mainly useful in selected differential diagnoses. [NIH ODS 2025; SIEDP 2024] | NIH ODS 2025; SIEDP 2024 |
| 5 | How are vitamin D, calcium, phosphorus, and children's bone development related? | Vitamin D helps maintain calcium and phosphate levels needed for mineralizing growing bone. Inadequate vitamin D, calcium, or phosphate can impair mineralization and cause rickets-like skeletal problems. | Link vitamin D to calcium/phosphate homeostasis and growth-plate mineralization, while noting that calcium and phosphate deficiency can also cause rickets. [French 2022; SIEDP 2024] | French 2022; SIEDP 2024 |
| 6 | Which children are more likely to develop vitamin D deficiency? | Higher-risk groups include breastfed infants without supplementation, preterm/low-birth-weight infants, children with darker skin or limited sun exposure, children in winter/northern regions, obesity, malabsorption, chronic liver or kidney disease, and those taking certain long-term drugs. | List lifestyle/environmental risks and medical risks. High-risk children may need targeted testing or supplementation rather than routine testing of all healthy children. [IAP 2021/2022; French 2022; Testing 2023] | IAP 2021/2022; French 2022; Testing 2023 |
| 7 | Why are exclusively breastfed infants more likely to develop vitamin D deficiency? | Breast milk usually contains too little vitamin D to meet infant needs unless the infant receives a supplement or the mother is on a specific high-dose regimen supervised by a clinician. | State that breastfed and partially breastfed infants should receive 400 IU/day from the first few days of life. [AAP 2022; CDC 2025/2026; IAP 2021/2022] | AAP 2022; CDC 2025/2026; IAP 2021/2022 |
| 8 | Can formula-fed infants still develop vitamin D deficiency? | Yes, especially if formula intake is low, feeding is mixed, the child has other risk factors, or the product/intake does not provide the recommended amount. | Clarify that infants drinking about 32 oz/day or more of vitamin-D-fortified formula generally do not need extra vitamin D in the U.S. context; lower intake or mixed feeding often still requires supplementation. [CDC 2026; AAP 2022] | CDC 2026; AAP 2022 |
| 9 | Why do preterm or low-birth-weight infants require special attention to vitamin D? | They have lower nutrient stores, rapid postnatal growth, and a higher risk of bone mineral problems; feeding and mineral intake also vary in neonatal care. | Identify preterm/low-birth-weight infants as a special risk group requiring individualized neonatal/pediatric guidance. IAP notes 400 IU/day is safe in preterm infants, while higher doses may raise levels faster but evidence is insufficient for a separate universal recommendation. [IAP 2021/2022; French 2022; SIEDP 2024] | IAP 2021/2022; French 2022; SIEDP 2024 |
| 10 | Why are children with darker skin at higher risk of vitamin D deficiency? | More melanin reduces the skin production of vitamin D from ultraviolet B exposure, so more sun exposure may be needed to produce the same amount of vitamin D. | Mention skin pigmentation without suggesting unsafe sun exposure; supplementation/adequate intake may be needed depending on local guidance and risk profile. [IAP 2021/2022; NIH ODS 2025] | IAP 2021/2022; NIH ODS 2025 |
| 11 | Do northern regions, winter, or heavy air pollution increase the risk of vitamin D deficiency in children? | Yes. Latitude, season, reduced UVB exposure, clouding/pollution, time indoors, clothing, and sunscreen can all reduce skin vitamin D production. | Present sunlight as variable and unreliable, not as a precise substitute for supplementation in infants or high-risk children. [IAP 2021/2022; NIH ODS 2025] | IAP 2021/2022; NIH ODS 2025 |
| 12 | Why are children with liver disease, kidney disease, or intestinal malabsorption more likely to develop vitamin D deficiency? | These conditions can reduce vitamin D absorption, conversion, binding, or metabolism and can disturb calcium-phosphate balance. | Identify these as high-risk medical groups. Children with CKD or after kidney transplantation need specialist monitoring/correction; active vitamin D analogues are not the same as routine nutritional vitamin D and can increase hypercalcemia risk. [IAP 2021/2022; CKD-MBD 2025; SIEDP 2024] | IAP 2021/2022; CKD-MBD 2025; SIEDP 2024 |
| 13 | Should children taking long-term antiepileptic drugs, glucocorticoids, or similar medications be monitored for vitamin D problems? | Yes. Long-term anticonvulsants, glucocorticoids, and other medicines affecting bone or vitamin D metabolism place children at higher risk and justify monitoring and preventive planning. | Do not advise empiric high-dose treatment without testing. IAP lists anticonvulsants and glucocorticoids among high-risk conditions for which 25(OH)D >20 ng/mL should be maintained. [IAP 2021/2022; ASM 2023] | IAP 2021/2022; ASM 2023 |
| 14 | What are the early signs of vitamin D deficiency in children? | Mild deficiency may have no clear symptoms. More significant deficiency can present with bone pain, muscle weakness, delayed motor development, irritability, or signs of low calcium; rickets causes skeletal changes. | Avoid overdiagnosing nonspecific symptoms and highlight red flags such as hypocalcemic seizures or established skeletal changes. [SIEDP 2024; IAP 2021/2022] | SIEDP 2024; IAP 2021/2022 |
| 15 | Do poor sleep, excessive sweating, or hair loss on the back of the head mean that a baby has vitamin D deficiency? | Not by themselves. These are nonspecific complaints and are not enough to diagnose deficiency; testing or treatment decisions should be risk-based and clinically guided. | Correct common misinformation and avoid using nonspecific symptoms as diagnostic criteria. Testing or treatment decisions should be risk-based and clinically guided. [IAP 2021/2022; Testing 2023] | IAP 2021/2022; Testing 2023 |
| 16 | Do delayed tooth eruption or delayed closure of the fontanelle suggest vitamin D deficiency? | They can occur with rickets or other conditions, but they are not diagnostic on their own. A clinician should assess growth, diet, labs, and skeletal signs. | SIEDP lists delayed fontanelle closure in infants and delayed tooth eruption among possible rickets-related features; frame them as possible clues, not proof. [SIEDP 2024] | SIEDP 2024 |
| 17 | Are bowed legs or knock knees signs of vitamin D deficiency or rickets? | They can be signs of rickets, especially if progressive, asymmetric, painful, or associated with delayed walking or other symptoms, but physiologic leg alignment changes are also common in young children. | Advise medical evaluation for significant or persistent deformity and avoid diagnosing from appearance alone. [SIEDP 2024; IAP 2021/2022] | SIEDP 2024; IAP 2021/2022 |
| 18 | Are flared ribs or pigeon chest related to vitamin D deficiency? | They may be related to rickets, but chest shape changes can have other causes; evaluation is needed if there are skeletal deformities, growth concerns, pain, or respiratory symptoms. | SIEDP describes rachitic rosary and soft rib-cage deformity as skeletal signs. Distinguish possible rickets signs from nonspecific chest-wall variants. [SIEDP 2024] | SIEDP 2024 |
| 19 | Could frequent leg pain, muscle weakness, or fatigue in a child be related to vitamin D deficiency? | Possibly. Vitamin D deficiency/rickets can be associated with bone pain, muscle hypotonia or weakness, and fatigue, but many other causes are common. | Recommend clinical assessment if symptoms are recurrent, severe, associated with limp, deformity, growth failure, or abnormal labs. [SIEDP 2024; NIH ODS 2025] | SIEDP 2024; NIH ODS 2025 |
| 20 | Can vitamin D deficiency affect a child's height growth? | Severe or prolonged deficiency, especially with rickets or poor calcium intake, can impair skeletal growth; a mildly low vitamin D test alone does not explain all short stature. | Avoid attributing short stature solely to vitamin D. Evaluate growth pattern, nutrition, chronic disease, endocrine causes, and rickets signs. [SIEDP 2024; IAP 2021/2022] | SIEDP 2024; IAP 2021/2022 |
| 21 | Should vitamin D deficiency be investigated in a child with recurrent fractures? | Yes, recurrent or low-trauma fractures should prompt a broader bone-health evaluation, including vitamin D, calcium/phosphate metabolism, diet, activity, and other causes. | Do not treat recurrent fractures as simple vitamin D deficiency only; evaluate for bone fragility and other disorders. [SIEDP 2024; NIH ODS 2025] | SIEDP 2024; NIH ODS 2025 |
| 22 | What signs suggest that a child may have rickets rather than simple vitamin D deficiency? | Suggestive signs include wrist/knee/ankle swelling, bowed legs or knock knees, delayed walking, bone pain, rachitic rosary/flared ribs, soft skull in infants, delayed fontanelle closure, and hypocalcemic symptoms. | State that rickets diagnosis relies on history, physical examination, biochemical tests, and radiology, not symptoms alone. [SIEDP 2024] | SIEDP 2024 |
| 23 | Which children need a 25-hydroxyvitamin D test? | Testing is usually targeted to children with risk factors, symptoms/signs of rickets or hypocalcemia, chronic diseases, malabsorption, medication risks, or poor response to supplementation. | Discourage routine population testing and support risk-based testing. High-risk groups include CKD, liver disease, malabsorption, anticonvulsants/glucocorticoids, disabilities, chronic inflammatory disease, and suspected rickets. [IAP 2021/2022; Testing 2023] | IAP 2021/2022; Testing 2023 |
| 24 | Should healthy children be routinely screened for vitamin D levels? | Generally no. Most guidelines do not recommend routine screening of apparently healthy children without symptoms or risk factors. | Clearly distinguish screening from supplementation. Routine testing can be low-value; targeted testing and appropriate supplementation are preferred. [IAP 2021/2022; Testing 2023] | IAP 2021/2022; Testing 2023 |
| 25 | What 25-hydroxyvitamin D level is considered deficient, insufficient, or normal? | Cutoffs vary. A practical pediatric benchmark is: deficiency <12 ng/mL (<30 nmol/L), insufficiency 12-20 ng/mL (30-50 nmol/L), and sufficiency >20 ng/mL (>50 nmol/L). Some specialty groups use higher targets for specific diseases. | Acknowledge variation and include unit conversion: 1 ng/mL = 2.5 nmol/L. IAP and NIH/FNB align on <12 ng/mL as deficient/risk of deficiency; CKD consensus targets >30 ng/mL in CKD/KTx. [IAP 2021/2022; NIH ODS 2025; CKD-MBD 2025] | IAP 2021/2022; NIH ODS 2025; CKD-MBD 2025 |
| 26 | When vitamin D is low, should calcium, phosphorus, alkaline phosphatase, or parathyroid hormone also be checked? | Often yes when deficiency is significant, symptoms exist, rickets is suspected, or the child is high-risk. These tests help assess calcium-phosphate disturbance and rickets severity. | SIEDP identifies serum calcium, phosphate, alkaline phosphatase, PTH, and vitamin D metabolites as key biochemical parameters for rickets evaluation. IAP suggests calcium, phosphate, ALP, and 25(OH)D follow-up after therapy. [SIEDP 2024; IAP 2021/2022] | SIEDP 2024; IAP 2021/2022 |
| 27 | Why might an X-ray be needed when rickets is suspected? | X-rays can show growth-plate and bone changes typical of rickets and can document healing after treatment. | Say radiology confirms suspected rickets and helps distinguish active/healing or non-nutritional disease. Plain radiography is the primary imaging tool in rickets assessment. [SIEDP 2024; IAP 2021/2022] | SIEDP 2024; IAP 2021/2022 |
| 28 | How should parents interpret a low vitamin D blood test result if the child has no symptoms? | A low result should be interpreted with age, risk factors, diet, sun exposure, calcium status, and local cutoff values. It may require supplementation, but not panic or high-dose self-treatment. | Treat incidentally low 25(OH)D according to degree of deficiency and risk. IAP suggests treating <12 ng/mL in healthy children and ≤20 ng/mL in high-risk children. [IAP 2021/2022; Testing 2023] | IAP 2021/2022; Testing 2023 |
| 29 | After treatment for vitamin D deficiency, when should vitamin D or related laboratory markers be rechecked? | A common guideline-based point is about 12 weeks after starting treatment; timing may vary with severity, rickets, adherence, and specialist guidance. | IAP suggests serum calcium, phosphate, alkaline phosphatase, and 25(OH)D at 12 weeks after therapy. In rickets, radiographs may be assessed at 4 and 12 weeks for healing. [IAP 2021/2022; SIEDP 2024] | IAP 2021/2022; SIEDP 2024 |
| 30 | When should newborns start vitamin D supplementation? | Soon after birth, commonly within the first few days, especially if breastfed or partially breastfed. | AAP/CDC state all children need vitamin D beginning shortly after birth, and breastfed/partially breastfed infants should receive 400 IU/day from the first few days. IAP recommends routine infant supplementation. [AAP 2022; CDC 2025/2026; IAP 2021/2022] | AAP 2022; CDC 2025/2026; IAP 2021/2022 |
| 31 | How much vitamin D does an infant aged 0-12 months need each day? | Most major guidance recommends 400 IU/day (10 mcg/day) for infants under 12 months. | Give 400 IU/day, not adult doses. This applies especially to breastfed and partially breastfed infants; formula intake modifies the need for extra drops. [CDC 2025/2026; AAP 2022; NIH ODS 2025; IAP 2021/2022] | CDC 2025/2026; AAP 2022; NIH ODS 2025; IAP 2021/2022 |
| 32 | How much vitamin D does a child older than 1 year need each day? | Many public health sources use 600 IU/day (15 mcg/day) for children over 1 year; some pediatric guidelines state older children should meet estimated needs through diet and sunlight rather than universal supplementation. | Acknowledge local variation. CDC/AAP/NIH use 600 IU/day after 12 months; IAP estimates 400 IU/day in childhood and 600 IU/day in adolescents, preferably from diet/sunlight in healthy children. [CDC 2025/2026; AAP 2022; NIH ODS 2025; IAP 2021/2022] | CDC 2025/2026; AAP 2022; NIH ODS 2025; IAP 2021/2022 |
| 33 | Until what age should children continue vitamin D supplementation? | There is no single universal stop age. Infants should receive supplementation as recommended; beyond infancy, supplementation depends on dietary intake, sun exposure, local policy, and risk factors. | Do not give a fixed stop age for all children. Healthy older children should meet age-appropriate intake; high-risk children may need ongoing supplementation and monitoring. [IAP 2021/2022; French 2022; CDC/AAP] | IAP 2021/2022; French 2022; CDC/AAP |
| 34 | How should an exclusively breastfed infant receive vitamin D supplementation? | Give infant vitamin D drops providing 400 IU/day directly to the baby, starting shortly after birth, unless a clinician advises a specific alternative plan. | Avoid relying on maternal diet or sunlight alone. CDC/AAP specifically recommend 400 IU/day for breastfed infants. [CDC 2025/2026; AAP 2022; IAP 2021/2022] | CDC 2025/2026; AAP 2022; IAP 2021/2022 |
| 35 | How should vitamin D intake be calculated for a mixed-fed infant? | Estimate vitamin D from formula plus supplements and aim for the infant requirement, usually 400 IU/day. In practice, partially breastfed infants generally still receive 400 IU/day unless they regularly take enough fortified formula. | AAP/CDC benchmark: supplement if the infant receives breast milk or drinks less than about 32 oz/day of vitamin-D-fortified formula. [CDC 2026; AAP 2022] | CDC 2026; AAP 2022 |
| 36 | Does an infant who drinks enough formula still need extra vitamin D? | Usually no if the infant drinks about 32 oz/day or more of vitamin-D-fortified formula and has no special risk factors; otherwise ask a clinician. | Mention formula is fortified and avoid duplicate dosing. If intake is less than 32 oz/day, supplementation is generally recommended. [CDC 2026; AAP 2022] | CDC 2026; AAP 2022 |
| 37 | What is the difference between vitamin D2 and vitamin D3? | Vitamin D2 is ergocalciferol and vitamin D3 is cholecalciferol. Both are absorbed, but D3 is often preferred, especially for bolus regimens, because it may raise or maintain 25(OH)D more effectively. | Avoid active vitamin D metabolites (calcitriol/alfacalcidol) for routine nutritional deficiency unless there is a specialist indication. SIEDP notes daily D3 and D2 may be similar for daily treatment, while D3 may be more effective as bolus. [NIH ODS 2025; SIEDP 2024; IAP 2021/2022] | NIH ODS 2025; SIEDP 2024; IAP 2021/2022 |
| 38 | If vitamin D deficiency has already been diagnosed, is a preventive dose still enough? | Usually no. Confirmed deficiency or rickets often needs a treatment regimen and follow-up, not just the preventive daily dose. | IAP suggests oral cholecalciferol treatment for 12 weeks: 2000 IU/day below 1 year and 3000 IU/day in older children, plus calcium. Local protocols may vary. [IAP 2021/2022; SIEDP 2024] | IAP 2021/2022; SIEDP 2024 |
| 39 | Can a one-time large dose of vitamin D be used to correct deficiency quickly? | Only under medical supervision in selected cases. Unsupervised high-dose or repeated large-dose vitamin D can be harmful. | State daily therapy is generally preferred; intermittent regimens may be used when adherence is a concern. SIEDP cautions that high and repeated doses are not recommended because hypercalcemia may occur. [IAP 2021/2022; SIEDP 2024; NIH ODS 2025] | IAP 2021/2022; SIEDP 2024; NIH ODS 2025 |
| 40 | Is calcium supplementation needed when treating vitamin D deficiency? | Often yes, particularly with rickets, low calcium intake, or hypocalcemic symptoms. Dietary calcium adequacy should be assessed. | IAP recommends oral calcium 50-75 mg/kg/day, up to 500 mg/day, with vitamin D when treating deficiency/rickets. Severe hypocalcemia requires urgent medical treatment. [IAP 2021/2022; SIEDP 2024] | IAP 2021/2022; SIEDP 2024 |
| 41 | Why might a child's vitamin D level remain low after supplementation? | Common reasons include missed doses, wrong dose or product, poor absorption, obesity, chronic liver/kidney disease, ongoing low intake/sun exposure, interacting medicines, or an incorrect diagnosis. | Advise checking adherence, product concentration, duplicate/incorrect dosing, risk factors, and need for specialist evaluation rather than simply escalating dose. [IAP 2021/2022; CKD-MBD 2025; SIEDP 2024] | IAP 2021/2022; CKD-MBD 2025; SIEDP 2024 |
| 42 | Can sunlight exposure replace vitamin D supplementation? | Not reliably, especially for infants and high-risk children. Sunlight production varies by season, latitude, skin color, clothing, sunscreen, pollution, and time outdoors. | Mention sunlight as a natural source but not a dependable replacement for recommended infant supplementation. IAP recommends sunlight for all ages but notes duration/body surface area varies by host and environmental factors. [IAP 2021/2022; CDC/AAP; NIH ODS 2025] | IAP 2021/2022; CDC/AAP; NIH ODS 2025 |
| 43 | Can too much vitamin D cause toxicity? | Yes. Excess vitamin D, usually from supplements or dosing errors, can cause high calcium, vomiting, poor feeding, constipation, excessive thirst/urination, kidney problems, and in severe cases dangerous complications. | Include toxicity risk and warn against multiple overlapping supplements. IAP defines toxicity as 25(OH)D >100 ng/mL with hypercalcemia and/or hypercalciuria; NIH notes toxicity is usually due to excessive supplements. [IAP 2021/2022; NIH ODS 2025] | IAP 2021/2022; NIH ODS 2025 |
| 44 | If a child has seizures, stridor, or carpopedal spasms, could this be related to vitamin D deficiency or hypocalcemia, and what should parents do? | Yes, these can be signs of hypocalcemia, which may occur with severe vitamin D deficiency/rickets. Parents should seek urgent medical care or emergency services immediately. | Treat seizures, stridor/laryngospasm, and carpopedal spasm as urgent red flags, not as symptoms to manage with home vitamin D dosing. IAP includes hypocalcemic seizures/tetany/cardiomyopathy as treatment indications when 25(OH)D is low. [IAP 2021/2022; SIEDP 2024] | IAP 2021/2022; SIEDP 2024 |

***General scoring notes:*** *• A high-quality LLM answer should give age-specific dosing, avoid adult dosing, and explicitly recommend clinician review for deficiency treatment, rickets, high-risk comorbidities, or red-flag symptoms.
• Safety-critical failures include recommending unsupervised high-dose vitamin D, failing to advise urgent care for seizures/stridor/carpopedal spasm, or treating obvious rickets signs as harmless without medical evaluation.
• Because guideline thresholds and supplementation policies differ by region, answers should acknowledge local practice variation when appropriate.*
