# Supplementary material for: Cross-sectional comparative evaluation of five large language model–driven chatbots on an expert-curated 44-question set of parent-facing questions about pediatric vitamin D deficiency
Source: Front Pediatr. 2026 Sep 9;14:1927624. doi: 10.3389/fped.2026.1927624 (PMC13597792; doi:10.3389/fped.2026.1927624)
Supplement: Supplementary file 3 [file Supplementaryfile3.docx]

Clean Q&A Record of Responses Across Five LLM Chatbots

*Pediatric Vitamin D Deficiency | 44 Questions | Five Models*

**Model order:** ChatGPT, Gemini, Qianwen, DeepSeek, Doubao. Responses have been reformatted into a clean record while preserving the original questions and model names.

# Question 1. What is pediatric vitamin D deficiency?

## ChatGPT

Pediatric vitamin D deficiency is a condition in children in which the level of vitamin D in the body is lower than needed for normal growth and bone development. Vitamin D is essential for calcium and phosphorus absorption, and insufficient levels can lead to weakened bones, skeletal deformities, delayed growth, and in severe cases, rickets.

Risk factors include limited sun exposure, darker skin, exclusive breastfeeding without supplementation, prematurity, certain chronic illnesses, and use of medications that affect vitamin D metabolism.

## Gemini

Pediatric vitamin D deficiency occurs when a child's body has insufficient levels of vitamin D, which is essential for the absorption of calcium and phosphorus. Because these minerals are the building blocks of bone, a deficiency during childhood—a period of rapid growth—can lead to significant skeletal and systemic health issues.

1. Causes and Risk Factors

The body typically obtains vitamin D through two primary sources: skin synthesis (via UV-B sunlight) and dietary intake. Deficiency usually stems from:

Lack of Sunlight: Staying indoors, living in high latitudes, or heavy use of sunscreen.

Dietary Gaps: Exclusive breastfeeding without supplementation (breast milk is naturally low in vitamin D) or a diet low in fortified foods and fatty fish.

Malabsorption: Conditions like Celiac disease or Cystic Fibrosis that prevent the gut from absorbing fat-soluble vitamins.

Skin Pigmentation: Higher levels of melanin reduce the skin's ability to produce vitamin D from sunlight.

2. Physical Manifestations

When vitamin D levels are critically low, the primary result is the softening and weakening of the bones.

Rickets: This is the most severe form of deficiency in children. It leads to structural deformities such as "bowed legs" or "knock-knees" because the bones are too soft to support the child's weight.

Growth Retardation: Children may not reach their expected height.

Delayed Development: Late crawling or walking, and delayed closing of the "soft spot" (fontanelle) on a baby's head.

Dental Issues: Weak tooth enamel and increased risk of cavities.

3. Diagnosis and Measurement

Deficiency is diagnosed by measuring the concentration of 25-hydroxyvitamin D [25(OH)D] in the blood. While standards vary slightly by medical organization, the following thresholds are commonly used:

4. Prevention and Treatment

Medical guidelines generally recommend a daily supplement of 400 IU for infants starting shortly after birth, and 600 IU for children older than one year. Treatment for an existing deficiency involves higher "loading doses" of Vitamin $D_3$ (cholecalciferol) or $D_2$ (ergocalciferol) supervised by a pediatrician to ensure levels return to the healthy range without reaching toxicity.

## Qianwen

Pediatric vitamin D deficiency is a condition in children characterized by insufficient levels of vitamin D in the body, which impairs calcium and phosphorus absorption. This leads to abnormal bone mineralization, potentially causing rickets (softening and weakening of bones), growth delays, muscle weakness, and skeletal deformities. Vitamin D is essential for bone development, immune function, and overall growth in children.

Key Causes:

Inadequate sun exposure (limited outdoor activity, use of sunscreen, living in high-latitude regions).

Insufficient dietary intake (low consumption of vitamin D-rich foods like fortified milk, fatty fish, or supplements).

Breastfeeding without supplementation (breast milk is naturally low in vitamin D).

Medical conditions (e.g., malabsorption syndromes, liver/kidney disorders, obesity).

Symptoms:

Delayed motor development (e.g., late crawling/walking).

Bone pain, fractures, or deformities (bowed legs, widened wrists/ankles).

Muscle weakness, hypotonia ("floppy infant" syndrome).

Dental abnormalities (delayed tooth eruption, enamel defects).

Diagnosis:

Confirmed via serum 25-hydroxyvitamin D (25(OH)D) testing:

Deficiency: <20 ng/mL (50 nmol/L).

Insufficiency: 20–30 ng/mL (50–75 nmol/L).

Sufficiency: ≥30 ng/mL (75 nmol/L).

Treatment & Prevention:

Supplementation: High-dose vitamin D (e.g., 2,000–4,000 IU/day for 6–12 weeks) followed by maintenance doses (400–600 IU/day).

Dietary adjustments: Fortified foods, fatty fish, egg yolks.

Safe sun exposure: 10–15 minutes of midday sun 2–3 times/week (varies by skin tone and geography).

Early intervention prevents long-term complications like stunted growth or osteoporosis. The American Academy of Pediatrics recommends 400 IU/day of vitamin D for all infants and children starting soon after birth.

## DeepSeek

Pediatric vitamin D deficiency is a condition in which children have insufficient levels of vitamin D in their bodies, leading to impaired calcium and phosphorus metabolism. This can result in rickets (bone softening and weakening), delayed growth, muscle weakness, and, in severe cases, skeletal deformities such as bowed legs or knocked knees. It often stems from inadequate sunlight exposure, poor dietary intake, or malabsorption issues.

## Doubao

Pediatric vitamin D deficiency refers to an insufficient level of vitamin D in children and adolescents, which impairs intestinal absorption of calcium and phosphorus. It can cause abnormal bone mineralization, leading to conditions like rickets in young kids, osteomalacia, poor bone growth, increased risk of skeletal deformities, and may also weaken immune function.

# Question 2. What is the main role of vitamin D in children's growth and development?

## ChatGPT

The main role of vitamin D in children's growth and development is to regulate calcium and phosphorus balance, which is critical for healthy bone formation and mineralization. Adequate vitamin D ensures proper skeletal growth, supports tooth development, and contributes to muscle function. It also plays a role in immune system regulation and overall cellular health. Without sufficient vitamin D, children are at risk for bone softening, deformities, delayed growth, and conditions such as rickets.

## Gemini

The primary role of vitamin D in children is to maintain the calcium and phosphorus balance in the body, which is the foundation for building a strong skeleton.

Without sufficient vitamin D, the body cannot effectively absorb these minerals from the diet, leading to several critical functions in development:

1. Bone Mineralization

Vitamin D acts as a "key" that opens the door for calcium absorption in the small intestine. In growing children, this calcium is deposited into the growth plates, hardening the cartilage into solid bone. This process ensures that bones are dense, strong, and capable of supporting the physical stress of movement and weight-bearing.

2. Skeletal Integrity and Growth

Beyond just hardening bones, vitamin D ensures that bones grow in the correct shape and length.

Prevention of Deformities: It prevents the softening of bones (malacia) that leads to the physical bowing of legs or thickening of wrists seen in rickets.

Height: Proper mineralization is necessary for children to achieve their genetically determined height potential.

3. Dental Development

Vitamin D is essential for the formation of enamel and dentin. Children with adequate levels are less likely to experience delayed tooth eruption or "soft teeth," which are more prone to decay and fractures.

4. Muscle Function

Vitamin D receptors are located throughout the muscular system. It plays a role in:

Muscle Strength: Helping children develop the strength needed for motor milestones like standing and walking.

Balance and Coordination: Assisting in the signaling between nerves and muscles to prevent falls.

5. Immune System Regulation

While its most visible role is skeletal, vitamin D is a potent immunomodulator. It helps the innate immune system recognize and fight off pathogens, potentially reducing the frequency of respiratory infections in school-aged children.

## Qianwen

The main role of vitamin D in children's growth and development is to regulate calcium and phosphorus metabolism, which is essential for proper bone mineralization and skeletal growth.

Its key functions include:

Bone Mineralization: It promotes the absorption of calcium and phosphorus from the intestines, ensuring that bones become hard and strong. Without sufficient vitamin D, bones can become soft, weak, or deformed (leading to rickets).

Neuromuscular Function: It supports normal muscle function and nerve transmission, which are necessary for motor development and coordination.

Immune System Regulation: It helps modulate the immune system, enhancing the body's defense against infections.

Cellular Growth: It plays a role in cell growth, differentiation, and apoptosis, influencing overall physical development.

## DeepSeek

The main role of vitamin D in children's growth and development is to regulate calcium and phosphorus absorption, which is essential for proper bone mineralization and skeletal growth. It helps prevent rickets, supports healthy tooth development, and contributes to immune function and muscle strength.

## Doubao

It mainly promotes the absorption of calcium and phosphorus in the intestines, maintains normal bone and tooth development, prevents rickets and skeletal deformities, regulates immune function, and supports normal growth and neuromuscular development in children.

# Question 3. Are vitamin D deficiency and rickets the same condition?

## ChatGPT

No, vitamin D deficiency and rickets are not the same, though they are related.

- Vitamin D deficiency refers to having insufficient levels of vitamin D in the body, which can affect calcium and phosphorus metabolism. It may be present without obvious symptoms.

- Rickets is a clinical disease that results from prolonged or severe vitamin D deficiency (or sometimes calcium deficiency). It is characterized by bone softening, skeletal deformities (such as bowed legs), delayed growth, and other signs of impaired bone mineralization.

In short, vitamin D deficiency is a biochemical condition, whereas rickets is the physical manifestation that can develop if the deficiency is severe or prolonged.

## Gemini

No, they are not the same, though they are closely linked. The simplest way to understand the difference is that Vitamin D deficiency is the cause, and Rickets is the potential consequence.

1. The Relationship: Cause vs. Effect

Vitamin D Deficiency: This is a biochemical state where the blood levels of Vitamin D are below the healthy threshold ($< 20$ ng/mL). A child can be Vitamin D deficient without showing any physical symptoms or bone damage yet.

Rickets: This is a clinical disease. It is the physical manifestation of extreme or prolonged Vitamin D deficiency. It occurs when the deficiency is so severe that the bones can no longer mineralize, causing them to become soft, weak, and deformed.

2. Key Differences

3. Not All Rickets is from Vitamin D

While Vitamin D deficiency is the most common cause (Nutritional Rickets), rickets can also be caused by other factors regardless of Vitamin D levels:

Calcium Deficiency: Even with enough Vitamin D, if a child's diet is extremely low in calcium, rickets can develop.

Hypophosphatemic Rickets: A genetic condition where the kidneys cannot hold onto phosphorus, which is also vital for bone hardening.

Kidney/Liver Disease: If these organs cannot convert Vitamin D into its active form, rickets can occur even if the child takes supplements.

In summary: All nutritional rickets involves Vitamin D deficiency, but not everyone with Vitamin D deficiency will develop rickets.

## Qianwen

No, vitamin D deficiency and rickets are not the same condition, although they are closely related.

Vitamin D deficiency is the cause (a metabolic state), while rickets is the result (a structural skeletal disease).

Vitamin D Deficiency (The Cause)

This refers to a systemic state where the body lacks sufficient vitamin D.

Definition: It is a biochemical abnormality where serum 25-hydroxyvitamin D levels are low (typically <20 ng/mL).

Scope: It is a metabolic issue affecting calcium and phosphorus regulation.

Reversibility: It is often asymptomatic and can be reversed relatively quickly with supplementation before permanent damage occurs.

Rickets (The Consequence)

This is the specific skeletal disease that occurs when vitamin D deficiency is severe and prolonged during childhood growth.

Definition: It is characterized by the defective mineralization of growing bones (growth plates).

Scope: It manifests as physical structural changes, such as bowed legs, widened wrists, and a "rachitic rosary" on the ribs.

Progression: Rickets represents the clinical "end-stage" of untreated vitamin D deficiency in a growing child.

Key Distinction

You can have Vitamin D deficiency WITHOUT rickets: A child may have low vitamin D levels but no visible bone deformities yet.

Rickets implies Vitamin D deficiency: If a child has rickets, they almost certainly have underlying vitamin D deficiency (or calcium/phosphorus metabolism disorders).

Clinical Significance: Recognizing vitamin D deficiency early allows doctors to treat the metabolic imbalance with supplements before it progresses to the skeletal deformities of rickets.

To further assist with your understanding of the progression, would you like to know the specific clinical stages of rickets (e.g., early vs. active stage) or how to interpret blood test results for diagnosis?

## DeepSeek

No, they are not the same condition. Vitamin D deficiency is a nutritional state where vitamin D levels are low, which may be asymptomatic or cause subtle metabolic changes (e.g., low calcium or phosphorus). Rickets is a clinical consequence of prolonged, severe vitamin D deficiency during childhood, leading to defective mineralization of growing bone and cartilage, resulting in skeletal deformities and growth disturbances. In other words, rickets is an advanced stage of vitamin D deficiency, but not all vitamin D-deficient children develop rickets.

## Doubao

No, they are not the same. Vitamin D deficiency is a nutritional status of insufficient vitamin D in the body. Rickets is a bone disease that is a severe complication caused by long-term untreated vitamin D deficiency. All rickets cases are related to vitamin D deficiency, but not all vitamin D deficiency will develop into rickets.

# Question 4. What does 25-hydroxyvitamin D mean?

## ChatGPT

25-hydroxyvitamin D [25(OH)D] is the main circulating form of vitamin D in the blood and the most reliable indicator of a person’s vitamin D status.

- It is produced in the liver from either vitamin D3 (cholecalciferol) or vitamin D2 (ergocalciferol) obtained from sunlight, diet, or supplements.

- Measuring serum 25(OH)D levels helps assess whether a child has sufficient, insufficient, or deficient vitamin D.

- Normal levels generally indicate adequate vitamin D for bone health and calcium metabolism, while low levels suggest deficiency and increased risk for conditions like rickets.

## Gemini

25-hydroxyvitamin D, often written as 25(OH)D or calcidiol, is the specific form of vitamin D that doctors measure in the blood to determine if a person has enough vitamin D.

It is essentially the "storage form" of the vitamin in your body.

1. The Production Process

Vitamin D goes through two chemical changes (hydroxylation) before it can actually do its job. 25(OH)D represents the halfway point:

Input: You get Vitamin D from the sun ($D_3$) or supplements ($D_2$ and $D_3$).

First Stop (The Liver): The liver takes that raw Vitamin D and converts it into 25-hydroxyvitamin D. This is the version that circulates in your bloodstream.

Second Stop (The Kidneys): When the body needs to use the vitamin, the kidneys convert 25(OH)D into 1,25-dihydroxyvitamin D (calcitriol), which is the "active" form that helps absorb calcium.

2. Why do we measure 25(OH)D instead of other forms?

You might wonder why we don't just measure the "active" form (1,25-dihydroxyvitamin D) produced by the kidneys. There are three main reasons why 25(OH)D is the "gold standard" for testing:

Longer Half-Life: 25(OH)D stays in your blood for about 2 to 3 weeks, whereas the active form disappears in a few hours. This makes 25(OH)D a much better reflection of your long-term status.

Higher Concentration: It is found in much higher amounts in the blood, making it easier and more accurate to detect.

Reflects Supply: It directly shows how much vitamin D you are getting from all sources (sun and food). Ironically, if you are deficient, your body might actually increase the active form temporarily to compensate, which would give a false impression of health if that were the only thing measured.

3. What the Numbers Mean

When you get a blood test result, the concentration of 25(OH)D is usually reported in ng/mL (nanograms per milliliter) or nmol/L (nanomoles per liter).

Deficient: Less than 20 ng/mL

Insufficient: 20 to 29 ng/mL

Sufficient: 30 to 100 ng/mL

In short, 25-hydroxyvitamin D is the most accurate "fuel gauge" for your body's vitamin D levels.

## Qianwen

25-hydroxyvitamin D [25(OH)D] is the major circulating form of vitamin D in the body and serves as the gold standard clinical marker for assessing an individual's vitamin D nutritional status.

Metabolic Origin

When vitamin D (either synthesized by the skin from sunlight or ingested through diet/supplements) enters the body, it is biologically inactive. It must first undergo a chemical process called hydroxylation in the liver to become 25-hydroxyvitamin D.

Although the kidneys further convert this into the "active" hormone (1,25-dihydroxyvitamin D) that regulates calcium, the 25-hydroxy form is the stable, storage version found in the blood .

Clinical Significance

Measuring 25-hydroxyvitamin D levels is the definitive way to determine if a child is deficient, insufficient, or has adequate levels of vitamin D.

Stability: It reflects the body's vitamin D stores over time because it has a relatively long half-life (about 2–3 weeks), unlike the active form which fluctuates rapidly .

Diagnostic Tool: It is the primary test used to diagnose rickets and osteomalacia risk.

Interpretation of Levels

While reference ranges can vary slightly by laboratory, the following criteria are commonly used (based on ng/mL):

Severe Deficiency: < 10 ng/mL (< 25 nmol/L)

Deficiency: < 20 ng/mL (< 50 nmol/L)

Insufficiency: 20–29 ng/mL (50–75 nmol/L)

Sufficiency: ≥ 30 ng/mL (≥ 75 nmol/L) .

Note: There is also a difference in units. 1 ng/mL is equal to 2.5 nmol/L.

Importance for Parents

When a doctor orders a "Vitamin D test," they are almost always measuring 25-hydroxyvitamin D. This number determines the dosage of supplements needed.

If the result is low, the doctor will prescribe a specific treatment dose to bring the level back to the sufficiency range, thereby preventing the bone deformities associated with rickets.

## DeepSeek

25-hydroxyvitamin D, often abbreviated as 25(OH)D, is the main form of vitamin D circulating in the blood. It is produced in the liver from vitamin D obtained through sun exposure, diet, or supplements. Measuring the level of 25(OH)D is the most reliable way to assess a person's vitamin D status, as it reflects both vitamin D from external sources and that produced by the body.

## Doubao

25-hydroxyvitamin D (25(OH)D) is the major circulating storage and biomarker form of vitamin D in the human body. It is the metabolite used clinically to accurately evaluate overall vitamin D nutritional status, and it is the standard indicator for diagnosing vitamin D deficiency in children.

# Question 5. How are vitamin D, calcium, phosphorus, and children's bone development related?

## ChatGPT

Vitamin D, calcium, and phosphorus are closely interconnected in supporting children’s bone development:

1. Vitamin D – Enhances intestinal absorption of calcium and phosphorus, the key minerals needed for bone mineralization. It also regulates blood levels of these minerals.

2. Calcium – The primary mineral in bones; essential for forming a strong and healthy skeletal structure.

3. Phosphorus – Works with calcium to form hydroxyapatite, the mineral complex that gives bones their strength and rigidity.

4. Bone Development – Adequate vitamin D ensures calcium and phosphorus are available in the right amounts for bones to grow and harden properly. Deficiency in vitamin D reduces mineral absorption, leading to soft or weak bones, delayed growth, and risk of rickets.

In summary, vitamin D acts as the facilitator, while calcium and phosphorus are the building blocks for healthy bone development in children.

## Gemini

The relationship between Vitamin D, Calcium, and Phosphorus is often described as a "team effort" where each member has a specific role in building a strong, healthy skeleton. Without any one of these three, a child's bone development will be compromised.

Here is how they interact to build bone:

1. The "Bricks": Calcium and Phosphorus

Calcium and phosphorus are the primary minerals that make up the physical structure of the bone.

They combine to form a hard mineral crystal called hydroxyapatite.

These crystals are deposited into the "soft" protein framework (collagen) of the bone.

This process, called mineralization, is what turns flexible cartilage into the hard, weight-bearing bone required for a child to stand, walk, and grow.

2. The "Logistic Coordinator": Vitamin D

Vitamin D does not actually become part of the bone itself; instead, its job is to ensure the "bricks" (calcium and phosphorus) are available for the body to use.

Absorption: Without Vitamin D, the child's intestines can only absorb about 10–15% of the calcium they eat. When Vitamin D levels are normal, this absorption rate jumps to 30–40% (and even higher during growth spurts).

Regulation: Vitamin D maintains the exact right concentration of these minerals in the bloodstream. If blood levels drop too low, the nerves and heart cannot function properly.

3. The Biological "Chain Reaction"

When a child is growing, the process looks like this:

Step 1: The child consumes calcium and phosphorus (from milk, yogurt, etc.).

Step 2: Active Vitamin D signals the gut to pull those minerals into the bloodstream.

Step 3: The minerals travel to the growth plates at the ends of the long bones.

Step 4: The minerals harden the growth plates into new bone, increasing the child's height and bone density.

4. What happens when Vitamin D is missing?

If a child has a Vitamin D deficiency, the "coordination" fails:

The gut cannot absorb enough calcium.

The body enters "Emergency Mode": To keep the heart and brain running, it releases parathyroid hormone (PTH) to leach calcium out of the bones and put it back into the blood.

Result: The bones become "demineralized." They turn soft, weak, and flexible, leading to the bowing and deformities seen in Rickets.

Summary Table

The Bottom Line: You can give a child all the calcium in the world, but without Vitamin D, the body cannot "unlock" that calcium to build strong bones. All three must be present in sufficient amounts to ensure a child reaches their full height and skeletal strength.

Expert Guide: Are you asking because you’re looking for ways to boost these through a specific diet, or are you concerned about a particular growth milestone?

## Qianwen

Vitamin D, calcium, and phosphorus form an interdependent partnership that is essential for building a child's skeletal structure.

In simple terms, calcium and phosphorus are the "building materials," while vitamin D is the "manager" that ensures these materials reach the construction site.

The Construction Analogy

To visualize their relationship, imagine a child's skeleton is a high-rise building under construction:

Calcium & Phosphorus (The Bricks): These minerals are the physical building blocks. About 99% of the body's calcium and 85% of its phosphorus are stored in bones and teeth. Without enough of them, the "building" has no substance.

Vitamin D (The Foreman/Transporter): This is the regulatory factor. Its primary job is to open the "gates" of the intestines to allow calcium and phosphorus to enter the bloodstream. Without the foreman (Vitamin D), the bricks (calcium/phosphorus) sit in the gut and are excreted rather than being used to build bone .

The Mechanism of Mineralization

The relationship relies on a precise balance to harden the bone:

Absorption: Vitamin D actively promotes the absorption of calcium and phosphorus from the food a child eats .

Transport: It maintains high enough levels of these minerals in the blood so they can be delivered to the growth plates (the ends of long bones).

Hardening (Deposition): Calcium and phosphorus combine to form a crystal structure called hydroxyapatite. This crystals coat the collagen framework of the bone, turning soft cartilage into hard, rigid bone .

Consequences of Imbalance

If one part of this relationship fails, the whole system collapses:

Vitamin D Deficiency (The Broken Supply Chain): Even if a child drinks plenty of milk (high calcium), a lack of Vitamin D means the body absorbs only 10-15% of it, compared to 30-60% when Vitamin D is sufficient . The "bricks" never make it to the bone.

Calcium/Phosphorus Deficiency (The Material Shortage): If there is enough Vitamin D to transport minerals, but the child's diet is devoid of calcium and phosphorus (no milk, leafy greens, or meat), the transporter has nothing to carry.

The Result: The bone matrix remains soft and uncalcified. As the child grows and bears weight, these soft bones bend, leading to the deformities of rickets (e.g., bowed legs) .

Summary for Parents

Don't skip Vitamin D: Taking calcium supplements without sufficient Vitamin D is often ineffective because the body cannot absorb the calcium.

Don't rely solely on Vitamin D: While Vitamin D is the key, you still need to provide the "lock" (calcium sources) for it to unlock. A balanced diet rich in dairy or fortified foods is still required .

## DeepSeek

Vitamin D, calcium, and phosphorus are closely interconnected in children's bone development:

- Calcium is the primary mineral that hardens and strengthens bones.

- Phosphorus works alongside calcium to form hydroxyapatite, the crystalline structure that gives bones their rigidity.

- Vitamin D promotes intestinal absorption of both calcium and phosphorus. Without sufficient vitamin D, even a calcium-rich diet cannot be properly absorbed.

In summary: Vitamin D enables calcium and phosphorus to be absorbed from the gut; calcium and phosphorus then deposit into growing bones. A deficiency in vitamin D leads to poor mineralization, causing soft, weak bones (rickets). Conversely, adequate levels of all three support normal skeletal growth, bone density, and tooth development.

## Doubao

Vitamin D promotes the intestinal absorption of calcium and phosphorus. Calcium and phosphorus are the key raw materials for bone mineralization and bone formation in children. Without enough vitamin D, calcium and phosphorus absorption drops sharply. The lack of these two minerals leads to poor bone calcification, soft bones, delayed bone growth, and eventually causes rickets and skeletal deformities in kids. The four are closely linked: vitamin D regulates the utilization of calcium and phosphorus, and together they support normal children's bone development.

# Question 6. Which children are more likely to develop vitamin D deficiency?

## ChatGPT

Children at higher risk of developing vitamin D deficiency include:

1. Infants who are exclusively breastfed without vitamin D supplementation, since breast milk alone may not provide enough vitamin D.

2. Premature or low-birth-weight infants, who have lower vitamin D stores at birth.

3. Children with darker skin, because higher melanin reduces vitamin D synthesis from sunlight.

4. Children with limited sun exposure, such as those living in northern latitudes, spending little time outdoors, or wearing clothing that covers most of the skin.

5. Children with chronic illnesses affecting absorption or metabolism of vitamin D, including liver disease, kidney disease, or gastrointestinal disorders (e.g., celiac disease, cystic fibrosis).

6. Children taking certain medications long-term, such as anticonvulsants, glucocorticoids, or drugs affecting fat absorption.

7. Obese children, as vitamin D is sequestered in adipose tissue and may be less bioavailable.

These factors either reduce vitamin D intake, limit its synthesis, or impair its metabolism, increasing the risk of deficiency.

## Gemini

While vitamin D deficiency can affect any child, certain groups are at a significantly higher risk due to biological, environmental, or lifestyle factors.

The children most likely to develop a deficiency fall into the following categories:

1. Exclusively Breastfed Infants

Breast milk is the ideal nutrition for babies, but it is naturally very low in vitamin D (usually containing less than 80 IU per liter).

Infants who do not receive a daily 400 IU supplement are at the highest risk for deficiency and rickets.

This risk is even higher if the mother herself is vitamin D deficient.

2. Children with Limited Sun Exposure

Since the body produces vitamin D through skin exposure to UVB rays, anything that blocks the sun increases the risk:

High Latitude: Living in northern regions where sunlight is weak during the winter.

Pollution: Heavy smog or cloud cover that filters out UVB rays.

Cultural Practices: Wearing clothing that covers most of the skin for religious or cultural reasons.

Indoor Lifestyle: Spending the majority of the day indoors (e.g., heavy use of screens or lack of outdoor play).

3. Children with Darker Skin Pigmentation

Melanin acts as a natural sunscreen. Higher levels of melanin in the skin reduce the body's ability to synthesize vitamin D from sunlight.

Children of African, Middle Eastern, or South Asian descent often require 3 to 5 times longer sun exposure than fair-skinned children to produce the same amount of vitamin D.

4. Children with Malabsorption Issues

Vitamin D is a fat-soluble vitamin, meaning it requires fat to be absorbed in the gut. Children with conditions that interfere with fat absorption are at risk:

Celiac Disease

Cystic Fibrosis

Inflammatory Bowel Disease (IBD)

Biliary Atresia or other liver diseases.

5. Obese Children

Vitamin D is sequestered (trapped) in body fat.

In children with high body fat percentages, the vitamin D is pulled out of the bloodstream and stored in fat tissue, making it unavailable for the body to use for bone development.

6. Children on Specific Medications

Certain medications speed up the breakdown of vitamin D in the liver:

Anti-epileptic drugs (e.g., carbamazepine or phenobarbital).

Glucocorticoids (long-term steroid use).

Antifungals.

Summary Table: High-Risk Groups

Expert Guide: Are you asking because you’re assessing the risk for a specific child, or are you looking for general guidelines to share with other parents?

## Qianwen

Based on the mechanisms of vitamin D synthesis and metabolism discussed previously, the following groups of children are at the highest risk for vitamin D deficiency:

Infants with Rapid Growth and Limited Reserves

Premature and Twin Infants: These babies miss the critical third-trimester transfer of nutrients from the mother, resulting in lower initial vitamin D stores. Combined with their rapid postnatal growth rate, their demand for vitamin D exceeds their supply .

Exclusively Breastfed Infants: While breast milk is the ideal food for babies, it contains very low levels of vitamin D. If these infants do not receive vitamin D supplements or adequate sunlight, they are at significant risk .

Children with Limited Sun Exposure

Since sunlight is the primary source of vitamin D, any factor that blocks UV rays poses a risk:

Geographic and Environmental Factors: Children living in high-latitude regions (where sunlight is weaker), polluted areas, or those living in shadowed urban environments have reduced synthesis capacity .

Lifestyle Factors: Children who spend most of their time indoors, wear protective clothing that covers the skin, or use high-SPF sunscreen consistently cannot synthesize sufficient vitamin D .

Skin Pigmentation: Children with darker skin have higher levels of melanin, which acts as a natural sunscreen and significantly slows down the production of vitamin D in the skin .

Children with Malabsorption Issues

Even with a good diet, some children cannot absorb vitamin D effectively due to medical conditions:

Gastrointestinal and Liver Diseases: Conditions like chronic diarrhea, cystic fibrosis, or liver diseases (e.g., cholestasis) interfere with the fat-soluble absorption of vitamin D .

Obesity: Vitamin D is fat-soluble and can get "sequestered" or trapped in body fat tissues, making it unavailable for circulation and use by the body .

Children on Specific Medications

Anticonvulsants and Steroids: Long-term use of certain drugs, such as anti-seizure medications (e.g., phenobarbital, phenytoin) or corticosteroids, can accelerate the breakdown of vitamin D in the liver or alter its metabolism .

Conclusion:

Children in these high-risk categories require proactive management. This typically involves ensuring adequate dietary intake through fortified foods or supplements and, where medically appropriate, monitoring blood levels to prevent the progression to rickets.

## DeepSeek

Children who are more likely to develop vitamin D deficiency include:

- Exclusively breastfed infants (breast milk is low in vitamin D) without supplementation.

- Children with limited sun exposure (e.g., those who live in high latitudes, cloudy climates, or stay indoors most of the time; or those who wear full-body coverings due to cultural or medical reasons).

- Children with darker skin (melanin reduces vitamin D synthesis from sunlight).

- Children with malabsorption conditions (e.g., cystic fibrosis, celiac disease, inflammatory bowel disease, or cholestatic liver disease).

- Children on certain medications (e.g., anticonvulsants, glucocorticoids, or antifungals like ketoconazole) that affect vitamin D metabolism.

- Children with obesity (vitamin D is sequestered in fat tissue, reducing its bioavailability).

- Children with chronic kidney or liver disease (impair conversion of vitamin D to its active form).

- Children on strict vegan diets without fortified foods or supplements.

## Doubao

Children in these groups are at higher risk of vitamin D deficiency: 1. Breastfed infants without vitamin D supplementation. 2. Children with little outdoor activity and rare sun exposure. 3. Kids with dark skin pigmentation, which reduces vitamin D synthesis from sunlight. 4. Children who are overweight or obese. 5. Those with picky eating, unbalanced diet and low intake of vitamin D-rich foods. 6. Premature infants and low birth weight babies. 7. Children with gastrointestinal, liver or kidney diseases that affect vitamin D absorption and activation.

# Question 7. Why are exclusively breastfed infants more likely to develop vitamin D deficiency?

## ChatGPT

Exclusively breastfed infants are more likely to develop vitamin D deficiency because breast milk typically contains low levels of vitamin D, often insufficient to meet an infant’s needs for proper bone growth and mineralization.

Unless the breastfeeding parent has very high vitamin D levels or the infant receives supplemental vitamin D drops, exclusively breastfed infants may not get enough vitamin D from diet alone. Combined with limited sunlight exposure, this makes them particularly vulnerable to deficiency and related conditions like rickets.

## Gemini

While breast milk is the "gold standard" for infant nutrition, providing nearly everything a baby needs for the first six months, it is naturally very low in vitamin D.

Exclusively breastfed infants are at a higher risk for deficiency due to a combination of biological and environmental factors:

1. Low Vitamin D Content in Breast Milk

Even in healthy mothers, breast milk typically contains very little vitamin D—usually less than 25 to 78 IU per liter.

The Gap: The recommended daily intake for an infant is 400 IU.

The Reality: To get the required amount from milk alone, an infant would have to drink over 5 to 10 liters of breast milk a day, which is physically impossible.

2. Maternal Vitamin D Status

The amount of vitamin D in a mother's milk is directly linked to her own vitamin D levels.

If a mother is deficient herself (which is common, especially in winter or in certain climates), her milk will have almost no detectable vitamin D.

Even if a mother takes a standard prenatal vitamin (usually containing 400–800 IU), it generally isn't enough to significantly raise the vitamin D levels in her milk to meet the baby's total needs.

3. Limited Sun Exposure

Historically, humans gained vitamin D through skin exposure to the sun. However, modern medical advice has changed for safety:

Sun Protection: The American Academy of Pediatrics (AAP) recommends keeping infants under 6 months out of direct sunlight to prevent skin damage and decrease skin cancer risk.

Sunscreen: Sunscreen effectively blocks the UVB rays needed to produce vitamin D.

The Result: Without sun exposure or a supplement, breastfed babies have no "backup" source for this nutrient.

4. Comparison: Breast Milk vs. Formula

Summary of Recommendations

Because of this natural "vitamin D gap," pediatricians universally recommend that all exclusively or partially breastfed infants receive a daily supplement of 400 IU of vitamin D starting in the first few days of life. This ensures the baby gets the "coordinator" they need to absorb calcium and build strong bones without relying on the low levels found in breast milk.

Expert Guide: Are you asking because you're navigating a breastfeeding journey right now, or are you looking for the best way to administer supplements to an infant?

## Qianwen

Exclusively breastfed infants are at higher risk for vitamin D deficiency primarily because human milk is a poor source of vitamin D, and relying on sunlight alone is often insufficient and unsafe for this age group.

Extremely Low Content in Breast Milk

Even if the nursing mother has adequate vitamin D levels, the concentration transferred to the infant through breast milk is very low.

Concentration Gap: Breast milk contains only 15–50 IU/L of vitamin D.

Insufficient Intake: To meet the recommended daily intake of 400 IU, an infant would need to consume roughly 8–10 liters of breast milk per day, which is physiologically impossible.

Maternal Dependency: The vitamin D content in milk is directly correlated with the mother's own serum levels. If the mother is deficient, the milk's content will be even lower.

Limitations of Sunlight Synthesis

While sunlight is the primary source of vitamin D for humans, it is not a reliable substitute for breast milk in infants for several reasons:

Safety Risks: Infants have very sensitive skin. The American Academy of Pediatrics (AAP) advises against direct sun exposure for babies under 6 months due to the high risk of sunburn and potential long-term skin damage.

Environmental Barriers: Factors such as high latitude, seasonal changes (winter), air pollution, and the use of window glass (which blocks UVB rays) significantly inhibit the skin's ability to synthesize vitamin D.

Rapid Growth and Low Reserves

Infants experience a rapid growth spurt during the first year of life, which demands high amounts of calcium and phosphorus for skeletal mineralization.

Depleted Stores: Newborns, especially premature ones, often enter the world with limited vitamin D reserves from the mother.

High Demand: The combination of rapid bone growth and the lack of dietary vitamin D from breast milk creates a metabolic deficit that leads to deficiency if not supplemented.

Clinical Recommendation

Due to these factors, exclusively breastfed infants (and partially breastfed infants who consume less than 1 liter of vitamin D-fortified formula per day) require a daily vitamin D supplement of 400 IU starting from the first few days of life to prevent deficiency and rickets.

## DeepSeek

Exclusively breastfed infants are more likely to develop vitamin D deficiency primarily because human breast milk contains very low levels of vitamin D — typically less than 25 IU per liter, which is far below the recommended daily intake of 400 IU for infants.

Other contributing factors include:

- Limited sun exposure – Infants are often kept out of direct sunlight or are protected with clothing and sunscreen, reducing skin synthesis of vitamin D.

- Maternal vitamin D status – If the mother is vitamin D deficient, her breast milk will have even lower vitamin D content.

- Rapid growth phase – Infants grow quickly and have high calcium and vitamin D demands for bone mineralization, making them more vulnerable to deficiency.

Without routine supplementation (as recommended by major pediatric organizations like the AAP), exclusively breastfed infants are at significant risk for vitamin D deficiency and rickets.

## Doubao

Human breast milk contains very low levels of vitamin D, far too little to meet an infant’s daily growth needs. Moreover, newborns have limited outdoor sun exposure and cannot synthesize enough vitamin D through skin sunlight. Without extra vitamin D supplementation, exclusively breastfed infants are highly prone to vitamin D deficiency.

# Question 8. Can formula-fed infants still develop vitamin D deficiency?

## ChatGPT

Yes, formula-fed infants can still develop vitamin D deficiency, although the risk is generally lower than in exclusively breastfed infants.

- Infant formula is usually fortified with vitamin D, providing about 400 IU per liter.

- However, deficiency can occur if the infant consumes insufficient amounts of formula, if the formula is diluted improperly, or if the infant has malabsorption issues or certain medical conditions affecting vitamin D metabolism.

- Limited sunlight exposure or underlying health problems can also contribute to deficiency even in formula-fed infants.

## Gemini

Yes, formula-fed infants can still develop vitamin D deficiency, although their risk is generally lower than that of exclusively breastfed infants.

The primary reason is a gap between the amount of vitamin D added to the formula and the volume of milk a baby actually drinks.

1. The "1-Liter" Rule

By law, infant formula is fortified with vitamin D (usually around 400 IU per Liter). However, a baby only receives that full dose if they drink at least 1,000 mL (approx. 33 oz) of formula in a 24-hour period.

The Reality: Most newborns and young infants drink much less than 1 liter per day. For example, a baby drinking 500 mL (17 oz) a day is only getting 200 IU—half of the recommended daily amount.

The Risk: During those early months when intake is low, a "vitamin D gap" can occur, leading to a slow depletion of the baby's stores.

2. Individual Absorption and Health Factors

Even if a baby drinks a full liter, other factors can interfere with how that vitamin D is used:

Body Weight: Infants with higher body weights or those who are obese sequester vitamin D in their fat cells, making less of it available in the bloodstream.

Skin Pigmentation: If a formula-fed infant has darker skin and a lower-than-average intake of formula, they are at a "double disadvantage" because their skin also produces less vitamin D from sunlight.

Malabsorption: If a baby has an underlying digestive issue, they may not be absorbing the fat-soluble vitamin D from the formula effectively.

3. When is a Supplement Needed for Formula-Fed Babies?

The American Academy of Pediatrics (AAP) and other global health organizations suggest the following:

4. Summary

It is a common misconception that formula is a "complete" replacement for vitamin D drops from day one. Because most babies don't reach the 1-liter-per-day milestone until they are several months old, many pediatricians recommend continuing the 400 IU drops until the baby is consistently drinking a large volume of formula or has transitioned to vitamin D-rich solid foods.

Expert Guide: Is your baby currently drinking a specific amount of formula each day? Knowing their daily intake can help determine if they've hit that "1-liter" safety threshold.

## Qianwen

Yes, formula-fed infants can still develop vitamin D deficiency. Although infant formula is fortified with vitamin D, deficiency can occur if the infant does not consume enough formula to meet their daily nutritional requirements or if there are issues with absorption.

The "Volume Threshold" for Sufficiency

The primary reason formula-fed infants become deficient is under-consumption.

Fortification Standards: Most standard infant formulas are fortified to contain approximately 400 IU/L of vitamin D.

The Calculation: To obtain the recommended 400 IU of vitamin D per day, an infant must consume a full liter (approx. 32 ounces) of formula.

The Risk: Many infants consume significantly less than 1 liter per day, especially in the early months or during weaning. If a baby drinks only 500–700 mL daily, they may only be getting 200–280 IU, leading to a gradual shortfall.

Contributing Risk Factors

Even with formula feeding, several factors can prevent the infant from reaching the necessary vitamin D levels:

Partial Formula Feeding: If parents mix formula with other fluids (like water) or give smaller volumes, the total vitamin D intake drops.

Malabsorption Disorders: Certain medical conditions affect the gut's ability to absorb fat-soluble vitamins like D. Examples include cystic fibrosis, celiac disease, or cholestatic liver disease.

Dark Skin Pigmentation: Infants with darker skin have higher melanin, which reduces the skin's ability to synthesize vitamin D from sunlight, making them more reliant on dietary intake.

Maternal Factors: Infants born to mothers with severe vitamin D deficiency may start with low reserves that formula alone cannot immediately replenish.

Clinical Management

Supplementation: If an infant is not drinking at least 1 liter (32 oz) of vitamin D-fortified formula daily, supplementation (e.g., 400 IU/day) is generally recommended to bridge the gap.

Monitoring: Pediatricians may monitor vitamin D levels in infants with risk factors such as malabsorption or those who are small for gestational age.

## DeepSeek

Yes, formula-fed infants can still develop vitamin D deficiency, although the risk is lower compared to exclusively breastfed infants. This can occur if:

- The infant does not consume enough formula – For example, partial formula feeding combined with low vitamin D intake from other sources, or if the infant drinks less than the typical daily volume (usually about 32 oz or 1 liter of standard formula provides 400 IU of vitamin D).

- The formula is not fortified with sufficient vitamin D – Most commercial infant formulas are fortified with vitamin D (around 400 IU per liter), but some specialized or homemade formulas may lack adequate amounts.

- The infant has malabsorption issues – Conditions like cystic fibrosis, cholestasis, or short bowel syndrome can impair vitamin D absorption even if the formula contains enough.

- Other risk factors coexist – Limited sun exposure, dark skin, certain medications, or chronic diseases can further increase risk.

Therefore, while routine vitamin D supplementation is not typically needed for formula-fed infants consuming adequate volumes of fortified formula, those with risk factors may still require screening or supplementation.

## Doubao

Yes, formula-fed infants can still develop vitamin D deficiency. Infant formula is fortified with vitamin D, but if the baby does not drink enough formula per day to reach the required vitamin D intake, or has insufficient sun exposure, digestive absorption problems, rapid growth with higher nutrient demands, deficiency may still occur. Routine vitamin D supplementation is still recommended for most formula-fed babies.

# Question 9. Why do preterm or low-birth-weight infants require special attention to vitamin D?

## ChatGPT

Preterm or low-birth-weight infants require special attention to vitamin D because:

1. Lower prenatal stores – Most vitamin D transfer from mother to fetus occurs in the third trimester. Preterm infants miss part of this transfer, leaving them with lower vitamin D reserves at birth.

2. Rapid growth needs – These infants have accelerated bone and tissue growth after birth, increasing their vitamin D and calcium requirements.

3. Immature organs – Their liver and kidneys, which are involved in converting vitamin D to its active form, may be underdeveloped, reducing vitamin D metabolism.

4. Higher risk of rickets and bone fragility – Without adequate supplementation, they are more prone to metabolic bone disease, fractures, and skeletal deformities.

Therefore, careful monitoring and supplementation are crucial to support their bone health and overall development.

## Gemini

Preterm and low-birth-weight infants are at a much higher risk for bone health issues because they miss out on the most critical period of skeletal development and have higher nutritional demands once born.

Here is why they require specialized attention:

1. Missing the "Mineral Dump"

The majority of a baby's mineral stores (calcium and phosphorus) and vitamin D are transferred from the mother during the third trimester of pregnancy.

The Issue: Preterm infants are born before this massive transfer is complete.

The Result: They enter the world with "empty tanks," meaning their bones are naturally less dense and their vitamin D stores are significantly lower than those of full-term babies.

2. Rapid "Catch-up" Growth

Once born, preterm infants undergo a period of rapid "catch-up" growth to reach the size they should have been at full term.

The Issue: Building bone that quickly requires a massive amount of building blocks (calcium and phosphorus) and the "coordinator" (vitamin D) to manage them.

The Result: Their daily requirement for vitamin D is often higher relative to their body weight than that of a full-term infant because their skeletal "construction site" is working overtime.

3. Metabolic Bone Disease of Prematurity (MBDP)

Because of their low mineral stores and high growth demands, preterm infants are susceptible to MBDP (formerly called osteopenia of prematurity).

This is a condition where the bones become exceptionally thin and fragile, increasing the risk of "silent" fractures—rib or limb fractures that can occur just from normal handling or diaper changes.

Ensuring adequate vitamin D is a primary strategy for preventing and treating this condition.

4. Immature Digestive Systems

Preterm babies often have immature guts that struggle to absorb nutrients, especially fats.

Since vitamin D is fat-soluble, a preterm infant may not absorb standard doses as efficiently as a full-term baby, requiring doctors to monitor their levels more closely.

Summary of Care Differences

Final Guidance

For these infants, neonatologists often prescribe specialized "Premature Formulas" or "Human Milk Fortifiers" that contain significantly higher levels of vitamin D, calcium, and phosphorus than standard versions. If your baby was born early, it is vital to follow the specific high-dose regimen prescribed by your NICU team or pediatrician rather than using standard over-the-counter drops.

Expert Guide: Was your baby born prematurely? Knowing their gestational age or current weight can help in understanding if they need a standard supplement or a more specialized "preemie" protocol.

## Qianwen

Preterm and low-birth-weight infants require special attention to vitamin D because they face a "perfect storm" of depleted initial reserves and excessive demand.

While term infants miss out on the last trimester of pregnancy—the critical window for nutrient transfer—preterm infants miss the rapid mineral accumulation phase needed for their accelerated postnatal growth.

Depleted Intrauterine Reserves

The majority of calcium, phosphorus, and vitamin D transfer from mother to fetus occurs during the third trimester (especially weeks 32–36) .

Missed Accumulation: Preterm infants are born before this critical window, resulting in significantly lower hepatic vitamin D stores and mineral reserves compared to term infants .

Low Birth Weight Correlation: The earlier the gestational age and the lower the birth weight, the fewer the vitamin D reserves at birth .

Accelerated "Catch-Up" Growth

Preterm infants undergo a rapid period of "catch-up growth" after birth to reach normal developmental milestones.

High Demand: This rapid skeletal growth requires massive amounts of calcium and phosphorus for bone mineralization.

Supply-Demand Mismatch: The limited reserves at birth are quickly consumed by this high metabolic demand. Without sufficient vitamin D to facilitate absorption, the bones cannot mineralize properly, leading to conditions like Metabolic Bone Disease of Prematurity (osteopenia) or rickets .

Physiological Immaturity and Absorption Issues

Beyond just intake, the body's ability to process vitamin D is compromised in preterm infants:

Digestive Immaturity: The gastrointestinal tract is not fully developed, leading to reduced absorption of fat-soluble vitamins like vitamin D .

Renal Losses: Preterm infants often have immature kidney function, which can lead to increased urinary excretion of calcium and phosphorus, further depleting mineral stores .

Clinical Recommendations for Special Attention

Because of these unique factors, standard supplementation for term infants (400 IU/day) is often insufficient for preterm infants during their early growth spurt.

Higher Dosage: Clinical guidelines typically recommend a higher initial dose of 800–1,000 IU/day for preterm infants to prevent rickets and metabolic bone disease .

Titration: After the initial period (often 3 months), the dosage is typically reduced to the standard 400 IU/day once the infant stabilizes and grows .

Nutritional Support: This is often accompanied by the use of specialized preterm formulas or breast milk fortifiers to ensure adequate calcium and phosphorus intake alongside vitamin D.

## DeepSeek

Preterm or low-birth-weight infants require special attention to vitamin D for several reasons:

- Lower placental transfer – Most vitamin D is transferred from mother to fetus during the third trimester. Preterm infants miss this critical period, so they are born with lower vitamin D stores.

- Higher growth demands – They have very rapid bone mineral accretion needs for catch-up growth, requiring more calcium and phosphorus, which depend on adequate vitamin D for absorption.

- Immature organ function – Their liver and kidneys are less developed, impairing the conversion of vitamin D to its active form (calcitriol).

- Limited intake initially – They often receive parenteral nutrition or small volumes of milk/formula in the early weeks, which may not provide sufficient vitamin D.

- Increased risk of metabolic bone disease – Preterm infants are prone to osteopenia of prematurity (rickets-like bone demineralization), which can lead to fractures and poor skeletal development without adequate vitamin D, calcium, and phosphorus.

For these reasons, many guidelines recommend higher vitamin D supplementation (e.g., 400–800 IU/day or more, depending on birth weight and clinical status) and routine monitoring of serum 25(OH)D, calcium, phosphorus, and alkaline phosphatase in this population.

## Doubao

Preterm and low-birth-weight infants have much lower stored vitamin D at birth. They grow far faster than full-term babies, leading to a much higher demand for vitamin D, calcium and phosphorus. Meanwhile, their immature gastrointestinal and liver function weakens nutrient absorption and vitamin D activation. All these factors make them far more vulnerable to vitamin D deficiency, rickets and impaired bone development, so they need higher-dose and earlier vitamin D supplementation.

# Question 10. Why are children with darker skin at higher risk of vitamin D deficiency?

## ChatGPT

Children with darker skin are at higher risk of vitamin D deficiency because higher levels of melanin in the skin reduce the skin’s ability to produce vitamin D from sunlight.

- Melanin acts as a natural sunscreen, limiting UVB radiation penetration, which is necessary for the skin to synthesize vitamin D.

- As a result, children with darker skin require longer sun exposure to produce the same amount of vitamin D as children with lighter skin.

- If dietary intake or supplementation is insufficient, this reduced synthesis increases the risk of deficiency and related bone problems.

## Gemini

The primary reason children with darker skin are at higher risk is due to melanin, the natural pigment that gives skin its color. In the context of vitamin D, melanin acts as a highly effective biological filter.

Here is the breakdown of why this happens:

1. The "Natural Sunscreen" Effect

Melanin’s biological purpose is to protect the skin from the harmful effects of ultraviolet (UV) radiation by absorbing and scattering it. However, the skin needs a specific type of radiation—UVB rays—to trigger the synthesis of vitamin D.

The Conflict: Melanin competes with the vitamin D precursors in the skin for these UVB rays.

The Result: Because melanin absorbs the UVB before it can trigger vitamin D production, a child with darker skin produces significantly less vitamin D from the same amount of sunlight as a fair-skinned child.

2. The Time Factor

Because of this filtering effect, the "dose" of sunlight required to reach healthy vitamin D levels is much higher for children with darker skin.

Fair Skin: May only need 10–15 minutes of midday sun a few times a week to produce sufficient vitamin D.

Darker Skin: May require 3 to 5 times longer (or even up to 10 times longer depending on the level of pigmentation) to produce the exact same amount. In many modern lifestyles, spending 1 to 2 hours in direct midday sun without sunscreen is often impractical or unsafe for the skin.

3. Geographic Mismatch

This risk is most pronounced when children with darker skin live in high-latitude regions (far North or South of the equator), such as Northern Europe, Canada, or the Northern United States.

In these areas, the sunlight is already weaker, especially in winter.

When you combine weak sunlight with high melanin levels, it becomes almost impossible for the skin to synthesize enough vitamin D naturally during many months of the year.

Summary of Differences

Why This Matters

Because it is much harder for children with darker skin to get enough vitamin D from the sun—especially while following safety guidelines to avoid skin cancer—pediatricians often emphasize that supplementation is particularly critical for this group to prevent bone issues like rickets.

Expert Guide: Are you living in a region with limited winter sunlight, or are you looking for advice on how to balance sun safety with vitamin D needs for your child?

## Qianwen

Yes, children with darker skin are at a higher risk of vitamin D deficiency primarily due to the high melanin content in their skin, which acts as a natural barrier against ultraviolet B (UVB) radiation.

While this provides protection against sunburn, it significantly slows down the skin's ability to synthesize vitamin D from sunlight.

The Mechanism: Melanin as a Natural Sunscreen

The synthesis of vitamin D in the skin relies on a photochemical reaction where UVB rays strike a precursor molecule called 7-dehydrocholesterol.

Competition for UVB: Melanin, the pigment that gives skin its color, competes with 7-dehydrocholesterol for the absorption of UVB rays .

Reduced Efficiency: In darker skin, the higher concentration of melanin absorbs more UVB radiation before it can reach the 7-dehydrocholesterol. Consequently, the rate of vitamin D production is much slower compared to lighter skin .

Time Disparity: To produce the same amount of vitamin D, individuals with dark skin may need to expose their skin to sunlight for 10 to 50 times longer than those with light skin .

Impact of Modern Lifestyles

The evolutionary advantage of dark skin (protection against intense UV radiation in equatorial regions) becomes a risk factor in modern environments.

Indoor Living: Most people, regardless of skin color, now spend the majority of their time indoors or in urban environments with limited sunlight.

Insufficient Synthesis: While lighter-skinned individuals can often synthesize enough vitamin D during short periods of outdoor activity, dark-skinned children may not produce sufficient amounts during these same intervals, leading to a chronic deficit .

Clinical Implications

Because sunlight is the primary source of vitamin D for most humans, dark-skinned children cannot rely on sun exposure alone to meet their nutritional needs.

Mandatory Supplementation: Clinical guidelines generally recommend that children with dark skin take daily vitamin D supplements (typically 400–600 IU/day), regardless of their diet or sun exposure habits .

Higher Dietary Needs: Since endogenous production is low, the reliance on exogenous sources (supplements or fortified foods) is significantly higher for this demographic .

## DeepSeek

Children with darker skin are at higher risk of vitamin D deficiency because melanin, the pigment responsible for darker skin color, naturally reduces the skin's ability to produce vitamin D from sunlight exposure. Specifically:

- Melanin absorbs UVB radiation – The same UVB rays that trigger vitamin D synthesis in the skin are absorbed by melanin, making less UVB available for vitamin D production.

- Higher melanin content = greater reduction – Darker skin may require 5–10 times longer sun exposure to produce the same amount of vitamin D as lighter skin.

- Geographic and lifestyle factors amplify the risk – In regions with low UVB intensity (e.g., high latitudes, winter months, cloudy weather, or indoor lifestyles), children with dark skin may produce very little, if any, vitamin D from sunlight.

As a result, even with apparently adequate sun exposure, dark-skinned children often have lower circulating 25(OH)D levels and may require dietary supplementation or fortified foods to maintain sufficient vitamin D status.

## Doubao

Melanin in darker skin can block ultraviolet rays from sunlight, which reduces the skin’s ability to synthesize vitamin D. Even with the same sun exposure, children with darker skin produce much less vitamin D naturally, making them more likely to have vitamin D deficiency.

# Question 11. Do northern regions, winter, or heavy air pollution increase the risk of vitamin D deficiency in children?

## ChatGPT

Yes, living in northern regions, during winter, or in areas with heavy air pollution can increase the risk of vitamin D deficiency in children:

1. Northern regions / high latitudes – Receive less UVB sunlight, especially in winter, reducing skin synthesis of vitamin D.

2. Winter season – Shorter daylight hours and weaker sunlight limit vitamin D production.

3. Heavy air pollution – Particulates and smog block UVB rays from reaching the skin, further decreasing vitamin D synthesis.

Combined, these factors can significantly reduce natural vitamin D production, making supplementation or dietary sources more important for children in these conditions.

## Gemini

Yes, these three factors significantly increase the risk because they all act as physical or atmospheric barriers that block UVB radiation, which is the only part of the light spectrum that triggers vitamin D synthesis in the skin.

1. Northern Regions (High Latitude)

The Earth's curvature means that in northern regions, sunlight must travel through a much thicker layer of the atmosphere.

The Angle of the Sun: In areas far from the equator (roughly above 37° latitude), the sun stays lower in the sky for most of the year. When the sun's angle is too low, the atmosphere absorbs and scatters almost all UVB rays before they reach the ground.

The "Vitamin D Winter": In places like Canada, Northern Europe, or Northern China, it is mathematically impossible to produce vitamin D from the sun for several months of the year, regardless of how much time a child spends outdoors.

2. Winter Season

Even in more temperate regions, winter poses a major challenge:

Atmospheric Filtering: During winter, the Earth tilts away from the sun. This increases the path length of sunlight through the ozone layer, which efficiently filters out UVB.

Behavioral Factors: Cold temperatures lead to children being heavily clothed and staying indoors. Since UVB cannot penetrate glass or most clothing, the body's "vitamin D factory" essentially shuts down until spring.

Depletion of Stores: Children often rely on vitamin D stored in their fat cells from the summer. By late winter (February/March), these stores are often exhausted, leading to peak deficiency rates.

3. Heavy Air Pollution

Air pollution acts like an "artificial cloud" that sits between the child and the sun.

Particulate Matter (PM2.5 and PM10): Tiny particles in smog and haze reflect and absorb UV radiation. Studies in highly polluted cities have shown that children living there have significantly lower vitamin D levels compared to those in rural, clear-air areas.

Ozone and Nitrogen Dioxide: These common pollutants are particularly effective at absorbing the specific wavelengths of light needed for vitamin D production.

Summary Table: Factors Blocking UVB

Because of these factors, children in northern, industrial, or cold-climate areas are frequently prescribed year-round vitamin D supplements to compensate for the lack of "natural" production.

## Qianwen

Yes, northern regions, winter, and heavy air pollution all significantly increase the risk of vitamin D deficiency in children.

These environmental factors collectively create a phenomenon often called the "vitamin D winter," where the intensity and duration of sunlight are insufficient to trigger vitamin D synthesis in the skin, regardless of how much time a child spends outdoors .

The "UVAngle" Effect (Northern Regions and Winter)

The primary driver of vitamin D synthesis is ultraviolet B (UVB) radiation.

Solar Zenith Angle: In northern latitudes or during winter months, the sun is lower in the sky. This increases the angle of incidence, causing sunlight to travel through a thicker layer of the atmosphere before reaching the ground .

Atmospheric Filtering: This longer path allows the ozone layer to filter out almost all UVB rays. Consequently, even if a child is outside for hours, their skin cannot produce vitamin D because the specific wavelengths required are absent.

Atmospheric Scavenging (Air Pollution)

Air pollution acts as a physical barrier that further reduces UVB penetration.

Absorption and Scattering: Particulate matter (PM2.5, PM10), smoke, and dust in the air absorb and scatter UVB radiation.

Urban vs. Rural: Studies indicate that children living in areas with high urban pollution or ozone levels have significantly lower vitamin D levels compared to those in rural areas with cleaner air . In heavily polluted cities, tall buildings can also block direct sunlight, exacerbating the deficit .

Practical Implications

In these environments, relying on "sunshine" as a source of vitamin D is unreliable and often impossible.

Supplementation is Non-Negotiable: For children living in northern regions or polluted city centers, especially during winter, dietary supplementation (400–800 IU/day) is the only effective method to maintain adequate levels .

Glass is Not a Solution: It is important to note that UVB rays cannot penetrate glass. Sitting by a window or driving with windows up provides warmth but zero vitamin D synthesis .

## DeepSeek

Yes, all three factors can independently and collectively increase the risk of vitamin D deficiency in children:

- Northern regions (high latitude) – Above approximately 37°N (e.g., much of the northern United States, Europe, Canada, and northern China), UVB radiation is insufficient for cutaneous vitamin D synthesis during winter months (typically November through February or March). Even midday sun exposure may not produce vitamin D.

- Winter season – Reduced solar zenith angle, shorter daylight hours, and colder weather lead to less time outdoors and more skin coverage with clothing. This dramatically reduces UVB exposure, making dietary or supplemental vitamin D critical.

- Heavy air pollution – Particulate matter (e.g., PM2.5, smog, soot) absorbs and scatters UVB radiation, potentially reducing skin synthesis by 50–90% in heavily polluted areas. This effect is observed even at lower latitudes.

When these factors combine (e.g., a polluted northern city in winter), the risk of deficiency becomes very high, and relying on sun exposure alone is unreliable. Supplementation and fortified foods become essential to maintain adequate 25(OH)D levels in children.

## Doubao

Yes, all three conditions significantly raise children’s risk of vitamin D deficiency. In northern regions and winter, the sun angle is low, ultraviolet radiation is weak, and outdoor time is reduced, limiting skin vitamin D synthesis. Heavy air pollution blocks ultraviolet rays from reaching the skin, further inhibiting natural vitamin D production. All these factors lead to insufficient endogenous synthesis and a higher deficiency risk.

# Question 12. Why are children with liver disease, kidney disease, or intestinal malabsorption more likely to develop vitamin D deficiency?

## ChatGPT

Children with liver disease, kidney disease, or intestinal malabsorption are more likely to develop vitamin D deficiency because these conditions interfere with the metabolism, activation, or absorption of vitamin D:

1. Liver disease – The liver converts vitamin D into 25-hydroxyvitamin D [25(OH)D], the main circulating form. Liver dysfunction reduces this conversion.

2. Kidney disease – The kidneys convert 25(OH)D into the active form, 1,25-dihydroxyvitamin D [calcitriol]. Impaired kidney function decreases active vitamin D levels.

3. Intestinal malabsorption – Conditions such as celiac disease, cystic fibrosis, or inflammatory bowel disease reduce absorption of fat-soluble vitamins, including vitamin D, from the diet.

These impairments lead to lower circulating and active vitamin D, increasing the risk of bone weakness, delayed growth, and rickets.

## Gemini

Children with these conditions are at much higher risk because the path from "taking a vitamin" to "having strong bones" is a multi-step process that requires the intestines, liver, and kidneys to be working perfectly. If any of these organs are compromised, the chain breaks.

Here is why each organ is critical:

1. Intestines: The "Entry Point"

Vitamin D is a fat-soluble vitamin. This means it can only be absorbed by the body if it is dissolved in dietary fat.

The Issue: Conditions like Celiac disease, Crohn’s disease, or Cystic Fibrosis damage the lining of the intestines or interfere with the enzymes needed to digest fat.

The Result: Even if a child takes a high-quality vitamin D supplement, it simply "passes through" the system and is lost in the stool because the gut cannot pull the vitamin into the bloodstream.

2. The Liver: The "First Processor"

The vitamin D a child gets from the sun or food is "raw" and inactive. It first travels to the liver.

The Issue: The liver produces an enzyme that performs the first chemical conversion, turning raw vitamin D into 25-hydroxyvitamin D [25(OH)D].

The Result: In children with biliary atresia or chronic liver failure, the liver cannot perform this conversion. Without this step, the vitamin D remains in a form that the body cannot use.

3. The Kidneys: The "Final Activator"

Even after the liver does its job, the vitamin is still not fully active. It must travel to the kidneys for the final, most crucial step.

The Issue: The kidneys convert 25(OH)D into 1,25-dihydroxyvitamin D, the "active hormone" that actually tells the gut to absorb calcium.

The Result: In children with Chronic Kidney Disease (CKD), the kidneys lose the ability to create this active form. This is why children with kidney issues often develop a specific type of bone disease called "Renal Osteodystrophy."

Summary of the "Activation Chain"

Medical Management

For children with these conditions, standard over-the-counter drops are often insufficient. Doctors may prescribe:

Water-miscible forms: Vitamin D that is specially formulated to be absorbed without needing fat (for gut issues).

Active Vitamin D (Calcitriol): A form of vitamin D that is already activated, bypassing the need for the liver or kidneys to process it.

Expert Guide: Are you asking because a child has a diagnosed condition in one of these areas, or are you looking to understand how these organs impact overall health?

## Qianwen

Children with liver disease, kidney disease, or intestinal malabsorption are at significantly higher risk for vitamin D deficiency because these conditions disrupt the essential metabolic pathway required for the body to utilize the vitamin.

Essentially, vitamin D is not active immediately upon intake; it must undergo specific processing (absorption and two hydroxylation steps) by these organs to become functional.

Intestinal Malabsorption: The Absorption Barrier

Vitamin D is a fat-soluble vitamin, meaning it requires dietary fat and bile acids to be dissolved and absorbed through the intestinal wall.

Mechanism of Deficiency: In conditions like Cystic Fibrosis, Celiac disease, or Short Bowel Syndrome, the intestine cannot properly absorb fats (often leading to steatorrhea). Consequently, vitamin D is not absorbed into the bloodstream but is instead excreted in the stool.

Result: Regardless of how much vitamin D is consumed or synthesized by the skin, the body cannot bring it "inside" to be used.

Liver Disease: The First Processing Failure

Once absorbed, vitamin D must travel to the liver to be converted into 25-hydroxyvitamin D

## DeepSeek

Children with liver disease, kidney disease, or intestinal malabsorption are more likely to develop vitamin D deficiency because each condition disrupts a critical step in the normal vitamin D metabolism pathway:

- Intestinal malabsorption (e.g., celiac disease, Crohn’s disease, cystic fibrosis, short bowel syndrome) – The small intestine cannot absorb fat-soluble vitamin D from dietary or supplemental sources. Even if intake is adequate, little reaches the bloodstream.

- Liver disease (e.g., biliary atresia, cholestasis, hepatitis, cirrhosis) – The liver is responsible for converting vitamin D (from skin or diet) into 25-hydroxyvitamin D, the main circulating form. Liver dysfunction impairs this first hydroxylation step, reducing the body’s vitamin D reserve.

- Kidney disease (e.g., chronic kidney disease, renal failure) – The kidneys convert 25-hydroxyvitamin D into its active hormonal form, 1,25-dihydroxyvitamin D (calcitriol). Kidney impairment reduces this second hydroxylation, leading to functional vitamin D deficiency even if 25(OH)D levels are normal.

As a result, these children often require higher doses of vitamin D supplementation, sometimes with activated forms (e.g., calcitriol for kidney disease), and close monitoring of calcium, phosphorus, and parathyroid hormone levels.

## Doubao

Vitamin D needs activation in the liver and kidneys to become its biologically active form. Liver disease impairs the first activation step, while kidney disease blocks the second activation process. Intestinal malabsorption reduces the body’s intake and absorption of dietary vitamin D. All these conditions disrupt vitamin D absorption, activation and utilization, greatly raising the risk of deficiency in children.

# Question 13. Should children taking long-term antiepileptic drugs, glucocorticoids, or similar medications be monitored for vitamin D problems?

## ChatGPT

Yes, children taking long-term antiepileptic drugs, glucocorticoids, or similar medications should be monitored for vitamin D problems because these drugs can interfere with vitamin D metabolism and bone health:

1. Antiepileptic drugs (e.g., phenytoin, phenobarbital) – Induce liver enzymes that accelerate the breakdown of vitamin D, lowering its levels.

2. Glucocorticoids – Reduce calcium absorption, increase calcium excretion, and impair bone formation, compounding the effects of vitamin D deficiency.

3. Other medications – Some anticonvulsants, antifungals, and certain weight-loss or anti-rejection drugs can also affect vitamin D metabolism.

Monitoring vitamin D status and supplementing when necessary helps prevent bone weakening, rickets, and long-term skeletal complications in these children.

## Gemini

Yes, children taking long-term antiepileptic drugs, glucocorticoids, or similar medications must be closely monitored for vitamin D deficiency and bone health issues.

These medications interfere with the body's natural ability to process vitamin D and calcium, potentially leading to "drug-induced" bone thinning or even rickets if not managed correctly.

1. Why do these medications cause problems?

Different classes of drugs "steal" vitamin D from the body through various biological mechanisms:

Antiepileptic Drugs (e.g., Phenobarbital, Phenytoin, Carbamazepine):

The Mechanism: These drugs activate specific enzymes in the liver (Cytochrome P450) that accelerate the breakdown and clearance of vitamin D.

The Result: The body "burns through" its vitamin D supply faster than it can be replenished, causing 25(OH)D levels to drop rapidly.

Glucocorticoids/Steroids (e.g., Prednisone, Dexamethasone):

The Mechanism: Steroids reduce the absorption of calcium in the intestines and increase the amount of calcium lost through urine. They also directly inhibit "osteoblasts," the cells responsible for building new bone.

The Result: Long-term use can lead to significantly decreased bone density and a higher risk of fractures.

Antifungals or HIV Medications:

Some of these treatments can interfere with the liver's ability to convert raw vitamin D into its active form.

2. Monitoring and Intervention Strategies

For children on these medications, the standard preventive dose of 400–600 IU is often not enough. Clinical management typically includes:

Regular Blood Testing: It is recommended to check 25(OH)D, serum calcium, phosphorus, and Alkaline Phosphatase (ALP) every 6 to 12 months.

Increased Dosage: Doctors often prescribe 2 to 3 times the standard daily dose of vitamin D to counteract the medication's effects.

Calcium Fortification: Ensuring the child has a high-calcium diet (dairy, fortified foods, or supplements) is vital to protect the skeleton from being "leached" of minerals.

Bone Density Scans: For children on high-dose steroids for more than 3 months, a DEXA scan may be ordered to monitor for early signs of osteoporosis.

3. Summary for Parents

Conclusion

While these medications are often life-saving or necessary to manage chronic conditions, their side effects on bone health must be actively managed. If your child is on any of these long-term treatments, talk to your pediatrician about a "Bone Protection Plan" to ensure their skeleton remains strong throughout their treatment.

Expert Guide: Is your child currently taking one of these medications? If so, has their doctor already discussed a specialized supplementation schedule with you, or have you noticed any symptoms like leg pain or fatigue?

## Qianwen

Yes, absolutely. Children on long-term antiepileptic drugs (AEDs) or glucocorticoids require strict monitoring of vitamin D levels and bone health.

These medications interfere with the body's ability to metabolize and utilize vitamin D, creating a high risk for bone demineralization, rickets, or osteoporosis if left unmanaged.

Mechanism of Drug-Induced Deficiency

These drugs disrupt the vitamin D pathway through different mechanisms:

Antiepileptic Drugs (Accelerated Catabolism):

Enzyme Induction: Certain AEDs (such as phenobarbital, phenytoin, and carbamazepine) act as hepatic enzyme inducers. They upregulate the cytochrome P450 system in the liver.

"Over-Metabolism": This accelerates the breakdown of vitamin D into inactive metabolites that are excreted from the body. Consequently, the body depletes its vitamin D stores much faster than normal, leading to reduced calcium absorption and potential hypocalcemia .

Glucocorticoids (Impaired Action):

Antagonism: Long-term use of steroids (like prednisone) interferes with vitamin D metabolism and antagonizes its effects on calcium absorption in the gut.

Direct Bone Toxicity: Steroids also directly suppress osteoblast function (bone building) and increase calcium excretion through the kidneys, compounding the risk of bone loss .

Clinical Monitoring and Intervention

Routine health checks for these children must include specific assessments to prevent skeletal complications:

Biochemical Screening:

Serum 25-Hydroxyvitamin D: This is the gold standard for assessing vitamin D status.

Calcium & Phosphorus: To check for mineral imbalances.

Parathyroid Hormone (PTH): Elevated PTH indicates secondary hyperparathyroidism, a common sign that the body is stripping calcium from bones to maintain blood levels due to vitamin D deficiency .

Bone Density Assessment:

Periodic Dual-Energy X-ray Absorptiometry (DXA) scans may be recommended to monitor bone mineral density, especially in children on high-dose or long-term therapy .

Proactive Supplementation:

Higher Dosages: Standard supplementation (400 IU) is often insufficient. These children may require therapeutic doses (e.g., 800–2,000 IU/day or more) to maintain sufficiency.

Calcium Co-administration: Ensuring adequate dietary calcium or supplements is crucial to compensate for the reduced absorption rates caused by these medications .

## DeepSeek

Yes, children taking long-term antiepileptic drugs (e.g., phenytoin, carbamazepine, phenobarbital, valproate), glucocorticoids (e.g., prednisone), or similar medications should be monitored for vitamin D problems. These medications increase the risk of vitamin D deficiency and bone complications through several mechanisms:

- Antiepileptic drugs – Induce liver enzymes (CYP450), accelerating the breakdown of 25-hydroxyvitamin D and 1,25-dihydroxyvitamin D; some also interfere with calcium absorption and bone mineralization.

- Glucocorticoids – Inhibit intestinal calcium absorption, increase urinary calcium excretion, reduce bone formation, and directly antagonize vitamin D activity.

- Other medications (e.g., antifungals like ketoconazole, antiretrovirals, chemotherapy drugs) may also interfere with vitamin D metabolism.

Recommended monitoring includes:

- Baseline and periodic serum 25-hydroxyvitamin D levels.

- Serum calcium, phosphorus, alkaline phosphatase, and parathyroid hormone.

- Consideration of bone density testing in long-term or high-risk cases.

Prophylactic vitamin D supplementation (often at higher-than-standard doses) is frequently advised, but monitoring ensures adequacy without toxicity.

## Doubao

Yes. Long-term use of antiepileptic drugs and glucocorticoids can accelerate the degradation of vitamin D in the body, reduce its activation, and hinder calcium absorption. This easily leads to vitamin D deficiency, reduced bone density and increased risk of rickets or osteoporosis. Therefore, these children need regular monitoring of vitamin D levels and timely supplementation.

# Question 14. What are the early signs of vitamin D deficiency in children?

## ChatGPT

Early signs of vitamin D deficiency in children can be subtle and may include:

1. Muscle weakness or hypotonia – Poor muscle tone or difficulty with motor skills.

2. Delayed growth – Slower increase in height or weight compared to peers.

3. Bone pain or tenderness – Especially in the legs, hips, or spine.

4. Frequent infections – Due to potential immune system impact.

5. Irritability or fatigue – Low energy or unusual fussiness in infants.

6. Mild skeletal changes – Slight widening of wrists or ankles, or delayed closure of the fontanelle in infants.

These signs often precede more obvious manifestations like bone deformities seen in rickets, so early detection and supplementation are important.

## Gemini

The early signs of vitamin D deficiency in children are often "silent" or non-specific, meaning they can easily be mistaken for normal childhood behavior or other minor ailments. Because vitamin D is essential for both bone health and the nervous system, early symptoms usually fall into two categories: physical/skeletal changes and neuromuscular/behavioral shifts.

Here are the signs to watch for:

1. Behavioral and Neuromuscular Signs

Before bones begin to bend, the lack of vitamin D affects how the child feels and moves.

Irritability and Restlessness: A child may become unusually fussy or difficult to soothe. This is often linked to subclinical bone pain or low-grade discomfort.

Excessive Sweating: One of the oldest noted signs is a "sweaty head," especially during sleep or feeding, even when the room temperature is cool.

Delayed Motor Milestones: You may notice the child is slow to reach milestones like sitting up, crawling, or walking. This is often due to hypotonia (decreased muscle tone) and muscle weakness.

"Floppiness": When picked up, an infant with early deficiency may feel less "firm" or exhibit less muscle resistance than expected.

2. Early Skeletal Changes

If the deficiency persists for several months, the body’s inability to harden bone tissue becomes physically apparent.

Craniotabes (Soft Skull): This is often the earliest skeletal sign in infants. If you press gently on the back or side of the skull, it may feel soft and "springy," similar to pressing on a ping-pong ball.

Delayed Fontanelle Closure: The "soft spot" on the top of the baby's head may remain large or fail to show signs of closing by the time the child is 18 months old.

Delayed Teething: A child may not get their first tooth until well after their first birthday, and the enamel may appear thin or pitted when the teeth finally emerge.

Shutterstock

3. Broad Growth Indicators

Poor Weight Gain: While not a direct cause, chronic vitamin D deficiency is often associated with a general "failure to thrive" where a child falls behind on their growth charts.

Frequent Infections: Because vitamin D is a key regulator of the immune system, a child who is constantly sick with respiratory infections may have an underlying deficiency.

Comparison: Early vs. Late Signs

Summary

Because these early signs—like sweating or fussiness—are so common in healthy children, they are rarely enough to make a diagnosis on their own. However, if a child is in a high-risk group (e.g., exclusively breastfed without supplements) and exhibits these signs, a pediatrician should evaluate them with a 25-hydroxyvitamin D blood test.

Expert Guide: Are you noticing any of these signs in a child currently, or are you looking for a preventative checklist to monitor as they grow?

## Qianwen

The early signs of vitamin D deficiency in children are often non-specific and subtle, primarily manifesting as changes in behavior and sleep patterns rather than obvious physical deformities.

These symptoms are frequently caused by mild hypocalcemia (low blood calcium) resulting from poor calcium absorption.

Neurological and Behavioral Changes (The "Silent" Signs)

In the initial stage (often called the initial or early stage of rickets), the most common indicators are related to nervous system excitability:

Night Sweating: Children may sweat excessively during sleep, particularly on the head, even when the room temperature is cool and not overly hot.

Irritability and Restlessness: The child may appear unusually fussy, crying more easily, or having difficulty settling down.

Sleep Disturbances: Frequent night waking, insomnia, or startling easily during sleep are common complaints.

Scalp Itching and "Pillow Baldness" (Pachytoid): Due to excessive sweating on the scalp, infants often rub their heads against the pillow to relieve itching. This repeated friction can lead to hair loss on the back of the head, known as "pillow baldness" or occipital alopecia.

Early Skeletal Signs (If Untreated)

If the deficiency persists beyond the early stage, physical signs begin to appear as the bones soften due to lack of mineralization:

Craniotabes: In infants under 6 months, the back of the skull may feel soft. Pressing on the occipital or parietal bones may produce a sensation similar to pressing on a ping-pong ball (it indents and springs back).

Delayed Milestones: Muscle weakness (hypotonia) may cause delays in sitting, standing, or walking.

Important Distinction

It is crucial to note that symptoms like sweating or irritability are not definitive proof of deficiency, as they can occur in healthy children. However, when they appear together in a child with risk factors (such as those mentioned in previous questions, like dark skin or lack of sun exposure), they serve as a vital warning signal to check vitamin D levels.

## DeepSeek

The early signs of vitamin D deficiency in children are often nonspecific but may include:

- Irritability or fussiness, especially in infants

- Poor sleep or disturbed sleep patterns

- Excessive sweating, particularly on the forehead (a classic early sign in infants)

- Delayed motor milestones (e.g., rolling over, sitting, crawling)

- Lethargy or decreased activity

- Muscle weakness or hypotonia (low muscle tone)

- Bone pain or tenderness (may be vague, such as leg pain or reluctance to walk)

- Frequent infections (vitamin D plays a role in immune function)

These early signs can be subtle and easily overlooked. If deficiency progresses, more specific signs of rickets (e.g., bone deformities, growth impairment, hypocalcemic seizures) may develop. Therefore, children with risk factors should be tested for serum 25(OH)D even before clear symptoms appear.

## Doubao

Early manifestations in children with vitamin D deficiency mainly include: 1. Neuropsychiatric symptoms: easy irritability, frequent crying, poor sleep, night terrors, excessive night sweating, and occipital alopecia. 2. Physical discomfort: frequent head sweating that soaks the pillow, restlessness when breastfeeding. 3. Subtle skeletal changes: soft skull, delayed fontanel closure. These usually appear before obvious skeletal deformities occur.

# Question 15. Do poor sleep, excessive sweating, or hair loss on the back of the head mean that a baby has vitamin D deficiency?

## ChatGPT

Not necessarily, but these can be early, nonspecific signs that may be associated with vitamin D deficiency in infants:

1. Poor sleep – Can occur due to general discomfort or bone pain, but is not specific to vitamin D deficiency.

2. Excessive sweating – Especially on the head, may sometimes be seen in infants with rickets or early vitamin D deficiency.

3. Hair loss on the back of the head – Often caused by friction in infants but can occasionally be linked to metabolic issues, including vitamin D deficiency.

Alone, these signs do not confirm deficiency. They should be considered alongside risk factors, growth patterns, and biochemical testing (serum 25-hydroxyvitamin D levels) for accurate diagnosis.

## Gemini

The short answer is not necessarily. While these signs are frequently discussed in parenting circles as symptoms of vitamin D deficiency, they are often non-specific, meaning they can be caused by many other normal physiological factors.

However, in the context of other risk factors, they can sometimes be "soft signs" that warrant a conversation with a pediatrician. Here is a breakdown of what these signs actually mean:

1. Hair Loss on the Back of the Head (Occipital Alopecia)

Many parents worry when they see a bald patch on the back of their baby's head, but it is rarely a sign of vitamin D deficiency.

The Likely Cause: This is almost always friction-induced. Because infants spend a significant amount of time on their backs (following "Safe to Sleep" guidelines), the constant rubbing of their head against the mattress as they look side-to-side causes the hair to break off.

When it resolves: Once the baby begins to sit up, crawl, and spend less time lying flat, the hair typically grows back.

2. Excessive Sweating (Especially During Sleep or Feeding)

"Sweaty head" is a classic old-wives' tale sign of rickets, and while it can be seen in severe cases of deficiency, it is very common in healthy babies for other reasons:

High Metabolism: Infants have a higher metabolic rate than adults, which generates more heat.

Immature Temperature Regulation: Babies primarily sweat through their heads because their sweat glands elsewhere aren't fully developed.

Physical Effort: For a baby, nursing or bottle-feeding is an athletic activity that requires significant effort, which often leads to perspiration.

Over-dressing: Parents often over-estimate how cold a baby is; if the room is warm or the baby is in heavy fleece, they will sweat.

3. Poor Sleep and Irritability

Vitamin D is involved in the synthesis of neurotransmitters that regulate mood and sleep, so extreme deficiency can cause restlessness.

The Reality: Poor sleep is a hallmark of infancy due to teething, growth spurts, sleep regressions, and developing circadian rhythms.

When to worry: If the irritability is accompanied by bone pain (the baby cries when being picked up or moved) or muscle weakness, it is more likely to be related to a vitamin deficiency.

The "Visible" vs. "Biochemical" Gap

It is important to remember that vitamin D deficiency is a biochemical diagnosis. A baby can look perfectly healthy—with a full head of hair and great sleep—and still have low vitamin D levels. Conversely, a baby with a bald spot and a sweaty head can have perfectly normal levels.

Shutterstock

Summary: When should you actually be concerned?

If these signs appear alongside more specific physical markers, you should seek a medical evaluation:

Delayed physical milestones (sitting, crawling).

Delayed tooth eruption (no teeth by 12 months).

Skeletal changes (a very large "soft spot" on the head or bowed legs).

The Bottom Line: Don't use hair loss or sweat as a diagnostic tool. Instead, ensure your baby is receiving the recommended 400 IU daily supplement, which is the best way to prevent deficiency regardless of what their hair looks like.

Expert Guide: Are you noticing these signs in a baby who is currently receiving a daily supplement, or are you just starting to look into vitamin D requirements?

## Qianwen

Not necessarily. While poor sleep, excessive sweating, and hair loss on the back of the head (often called "pillow baldness" or occipital alopecia) are classic textbook signs of vitamin D deficiency, in modern clinical practice, these symptoms are extremely common in healthy babies and are rarely caused by a lack of vitamin D alone.

Relying solely on these signs to diagnose a deficiency can lead to unnecessary anxiety. Here is why these symptoms usually do not indicate a medical problem in today's context:

The "Pillow Baldness" Misconception

Hair loss on the back of the head is frequently a result of friction, not a disease.

Mechanism: Infants spend a significant amount of time lying on their backs. As their heads grow and they begin to turn them side-to-side (sometimes due to colic or simply exploring), the hair follicles get rubbed against the mattress.

Outcome: This mechanical friction causes hair loss. Since baby hair grows in cycles and often sheds naturally around this age, the "bald patch" is often just a temporary phase of hair growth and friction.

Excessive Sweating and Temperature Regulation

Babies are not just "miniature adults"; their autonomic nervous system and thermoregulation are immature.

Metabolism: Infants have a higher metabolic rate than adults, generating more body heat.

Sweat Glands: Their sweat glands are concentrated heavily on the head. Even a slightly warm room or being dressed in too many layers can cause significant head sweating during sleep.

Context: Unless the baby is sweating profusely in a cool environment (e.g., <20°C/68°F) accompanied by other symptoms, mild sweating is usually physiological and normal .

Sleep Patterns and Development

Sleep disturbances like night waking or fussiness are often linked to developmental milestones (such as teething or learning to roll over) rather than calcium or vitamin D levels.

While severe vitamin D deficiency can cause nervous excitability, most "normal" fussy babies are simply going through a developmental stage.

The Only Way to Know for Sure

Because these physical signs are so common in healthy children, doctors no longer use them as primary diagnostic criteria.

Supplementation is Key: If your baby is taking the recommended daily dose of vitamin D (usually 400–800 IU), the likelihood of these symptoms being caused by a deficiency is very low.

Lab Testing: The only objective way to confirm a deficiency is through a blood test measuring Serum 25-hydroxyvitamin D.

Summary: If your baby is taking vitamin D supplements and growing well, these symptoms are likely just part of normal infant development.

However, if you are concerned or if the baby is not taking supplements, a blood test is the best next step.

## DeepSeek

Not necessarily. While poor sleep, excessive sweating (especially on the forehead), and occipital hair loss (bald spot on the back of the head) are classically described as possible early signs of vitamin D deficiency or rickets in infants, these symptoms are not specific to vitamin D deficiency and can be caused by other common conditions, such as:

- Overheating or over-bundling – A common cause of sweating and restless sleep in infants.

- Normal infant sleep patterns – Many babies have fragmented sleep or fussiness for non-medical reasons.

- Friction alopecia – Hair loss on the back of the head often results from repeated rubbing against a mattress or crib surface, especially in infants who sleep on their backs. This is usually benign and resolves on its own.

- Other medical conditions – E.g., gastroesophageal reflux, infection, or metabolic disorders.

Therefore, while these signs should prompt consideration of vitamin D deficiency—particularly in a child with known risk factors (e.g., exclusively breastfed, dark skin, limited sun exposure)—they are not diagnostic. The only reliable way to confirm or rule out vitamin D deficiency is a serum 25-hydroxyvitamin D test. If you observe these symptoms, consult a pediatrician rather than assuming deficiency or starting high-dose supplementation without testing.

## Doubao

Not exactly. These symptoms can be early signs of vitamin D deficiency, but they are not specific or diagnostic. Poor sleep, excessive sweating and occipital alopecia can also be caused by hot environment, thin hair friction, immature nerve development, or ordinary infant physiological characteristics. They cannot alone confirm vitamin D deficiency. Clinical diagnosis relies on laboratory tests and comprehensive evaluation instead of just these symptoms.

# Question 16. Do delayed tooth eruption or delayed closure of the fontanelle suggest vitamin D deficiency?

## ChatGPT

Yes, delayed tooth eruption and delayed closure of the fontanelle can suggest vitamin D deficiency in infants and young children:

- Delayed tooth eruption – Vitamin D deficiency can impair calcium and phosphorus metabolism, slowing dental development.

- Delayed closure of the fontanelle – The soft spots on an infant’s skull may remain open longer than normal due to impaired bone mineralization.

These signs, especially when combined with other clinical features (e.g., bone deformities, growth delay) and risk factors, warrant evaluation of vitamin D status.

## Gemini

Yes, delayed tooth eruption and delayed closure of the fontanelle are two of the most significant physical markers of vitamin D deficiency and nutritional rickets in infants and young children.

Because vitamin D is the primary regulator of the minerals (calcium and phosphorus) that harden bone and dental tissue, a deficiency acts as a "biological brake," slowing down these developmental milestones.

1. Delayed Closure of the Fontanelle

The fontanelle is the "soft spot" on a baby's head where the skull bones have not yet fused.

Normal Timeline: The anterior fontanelle (the largest one on top) typically closes between 9 and 18 months of age.

The Deficiency Link: In children with vitamin D deficiency, the skull bones (the frontal and parietal bones) remain soft and fail to mineralize at the edges. This prevents them from growing together and closing the gap.

What to Look For: A fontanelle that remains wide open or feels very soft well after the 18-month mark is a classic sign of rickets.

2. Delayed Tooth Eruption

Teeth, like bones, require calcium and phosphorus to form their hard outer structure (enamel) and inner body (dentin).

Normal Timeline: Most babies get their first tooth between 6 and 10 months.

The Deficiency Link: If vitamin D is low, the body prioritizes keeping calcium in the blood for the heart and muscles rather than using it to harden teeth. This can stall the teeth beneath the gumline.

What to Look For: "Delayed eruption" is generally defined as the absence of any teeth by 12 to 13 months of age.

3. Other Dental Complications

Even when the teeth finally do emerge in a deficient child, they may show signs of poor development:

Enamel Hypoplasia: The enamel (the hard white coating) may be thin, pitted, or appear yellowish/brownish because it didn't mineralize correctly.

Increased Cavities: Because the enamel is weaker, these teeth are much more susceptible to decay (caries) even with good hygiene.

4. Craniotabes (Soft Skull)

Closely related to fontanelle issues is craniotabes. This is a condition where the skull bones (usually at the back of the head) feel soft and "springy," similar to pressing on a ping-pong ball. While this can be normal in newborns, if it persists or appears in an older infant, it is a hallmark sign of vitamin D deficiency.

Summary

If a child is reaching their first birthday without any teeth, or their soft spot shows no signs of closing by 18 months, a pediatrician should evaluate them for vitamin D deficiency. These are "visible clues" that the body’s mineralization process is falling behind.

Expert Guide: Are you noticing these signs in a child currently, or are you looking for a checklist of milestones to monitor?

## Qianwen

Yes, they are classic indicators of skeletal involvement in vitamin D deficiency (Rickets), but they are not definitive proof on their own.

While delayed tooth eruption and delayed closure of the fontanelle are hallmark signs of the active phase (激期) of rickets, they can also occur in healthy children due to genetic factors.

Here is how to interpret these signs correctly:

Why Vitamin D Deficiency Causes These Delays

Vitamin D is essential for the mineralization (hardening) of bones and teeth.

Fontanelle Closure: The fontanelle (soft spot) is where skull bones haven't fused yet. If vitamin D is lacking, calcium cannot be deposited into the skull bones to harden them. This keeps the fontanelle open longer than usual (often beyond 18–24 months).

Tooth Eruption: Teeth need calcium to form and push through the gums. Deficiency can slow down the development of the jawbone and the teeth themselves, delaying the first tooth (often past 10–12 months).

The "Normal Variation" Trap

It is a common misconception that "late = sick."

Genetics: Just like walking or talking, skeletal development has a wide range of normal. Many healthy children have delayed teething or fontanelle closure simply because their parents did too (genetic predisposition).

Prematurity: Premature babies often have "adjusted ages," and their milestones (including bone closure) will naturally be later.

When to Be Concerned (Red Flags)

You should not worry about these signs in isolation. They become strong indicators of vitamin D deficiency only when:

They occur together: Both teeth and fontanelles are delayed simultaneously.

Accompanied by deformities: The baby has a "square head" (方颅), "rachitic rosary" (puffy joints on the ribs), or bowed legs.

No supplementation: The child has not been taking the recommended 400–800 IU of vitamin D daily.

Summary

If a baby is taking vitamin D supplements and growing well, a slightly late tooth or an open soft spot is usually just normal development. However, if these delays are significant (e.g., no teeth by 1 year, fontanelle not closed by 2 years), a doctor should evaluate the child to rule out metabolic bone disease.

## DeepSeek

Yes, both delayed tooth eruption and delayed closure of the anterior fontanelle can suggest vitamin D deficiency, particularly when the deficiency is prolonged and has led to rickets. However, these signs are not specific and can also be caused by other conditions.

- Delayed tooth eruption – In rickets, impaired mineralization of developing teeth can delay eruption. However, normal variation ranges widely; some healthy children do not get their first tooth until 12–15 months. Other causes include genetic disorders, hypothyroidism, or malnutrition.

- Delayed fontanelle closure – The anterior fontanelle typically closes between 12–18 months. Persistent openness beyond 18–24 months can be seen in rickets due to poor skull mineralization. Other causes include hypothyroidism, Down syndrome, achondroplasia, or increased intracranial pressure.

Key point: These signs are late manifestations of significant, untreated vitamin D deficiency (rickets), not early indicators. If they are present, the child likely has other signs of rickets (e.g., bone deformities, growth failure, hypocalcemia). Pediatric evaluation with serum 25(OH)D, calcium, phosphorus, and alkaline phosphatase is warranted. Conversely, isolated delayed tooth eruption or fontanelle closure without other findings is often due to normal variation or another underlying condition, not vitamin D deficiency.

## Doubao

They can be suggestive clues of vitamin D deficiency, but are not exclusive diagnostic evidence. Delayed tooth eruption and delayed fontanelle closure are typical manifestations of severe or prolonged vitamin D deficiency in children, which affects bone and tooth development. However, these conditions can also be caused by genetic factors, individual developmental differences, malnutrition or other metabolic diseases. A definite diagnosis needs combining laboratory vitamin D testing and clinical overall assessment.

# Question 17. Are bowed legs or knock knees signs of vitamin D deficiency or rickets?

## ChatGPT

Yes, bowed legs (genu varum) or knock knees (genu valgum) are classic skeletal signs of rickets, which results from prolonged or severe vitamin D deficiency.

- Bowed legs – Often seen in toddlers who are learning to walk; caused by softening of the long bones in the legs due to poor mineralization.

- Knock knees – May develop later as the child grows; also a result of weakened bones unable to support normal alignment.

These deformities indicate that the deficiency has progressed beyond biochemical changes and is affecting bone structure.

## Gemini

Yes, frequent leg pain, muscle weakness, and fatigue are common—though often overlooked—symptoms of vitamin D deficiency in children. While many people associate vitamin D only with "bone" issues, it plays a vital role in how muscles and nerves function.

When a child is deficient, these symptoms usually occur for the following biological reasons:

1. Bone Pain (Osteomalacia)

Unlike the sharp pain of a fracture, vitamin D deficiency causes a condition called osteomalacia, which is a softening of the bones.

The Sensation: This is often described as a "dull, deep, aching pain" that is felt in the bones themselves rather than the joints.

Location: It most commonly affects the legs (shins and thighs), lower back, and hips, as these are the weight-bearing areas of the skeleton.

Timing: The pain may worsen at night or after physical activity, often being mistaken for simple "growing pains."

2. Muscle Weakness and "Floppiness"

Vitamin D receptors are located directly on muscle cells. Vitamin D is required for muscles to contract and relax efficiently.

Hypotonia: In infants and toddlers, a lack of vitamin D can lead to "floppy" muscles (hypotonia).

Functional Weakness: Older children may have difficulty with tasks like climbing stairs, jumping, or getting up from a sitting position on the floor.

Waddling Gait: In more advanced cases (rickets), a child may develop a distinctive "waddling" walk because their pelvic muscles are too weak to stabilize their hips properly.

3. Chronic Fatigue

Vitamin D is involved in mitochondrial function—the part of the cell that generates energy.

A child with low levels may seem unusually tired, lack the energy to play with peers, or want to be carried frequently.

Research has shown that correcting vitamin D deficiency can significantly improve energy levels and reduce daytime sleepiness in both children and adults.

How to Distinguish from "Growing Pains"

It can be difficult to tell the difference between normal growing pains and vitamin D deficiency. However, there are a few clues:

Summary

If a child consistently complains of leg pain, seems "clumsy" or weak, or is always exhausted, it is worth discussing a 25-hydroxyvitamin D [25(OH)D] blood test with a pediatrician. Unlike many other causes of leg pain, vitamin D deficiency is easily and safely treatable with proper supplementation.

Expert Guide: Are these symptoms appearing alongside any other changes, such as a change in how your child walks or a recent growth spurt?

## Qianwen

Yes, they can be signs of the active phase of rickets, but they are also often a normal part of growth.

Bowed legs (O-legs) and knock knees (X-legs) are classic clinical manifestations of skeletal deformities caused by severe vitamin D deficiency . However, distinguishing between a pathological deformity and a normal developmental stage is one of the most common challenges in pediatrics.

Here is how to differentiate them:

Physiological vs. Pathological Variations

Most children experience changes in leg alignment as they grow, which is usually harmless:

Physiological Bow Legs (0–2 years): Due to their position in the womb, most infants have bowed legs. This is normal and typically corrects itself by age 2 as they start walking.

Physiological Knock Knees (2–5 years): As children grow, their leg alignment often shifts from bowed to knock-kneed. This peaks around age 3–4 and usually straightens out by age 7 or 8.

Signs That Suggest Rickets (Red Flags)

If bowed legs or knock knees are caused by vitamin D deficiency (rickets), they usually present with specific characteristics that differ from normal growth:

Progressive Worsening: Instead of improving with age, the deformity gets more severe over time .

Severe Degree: The gap between the knees (in O-legs) or ankles (in X-legs) is significant (often >3cm to 6cm) .

Asymmetry: One leg looks significantly different from the other.

Accompanied by Other Signs: These leg issues occur alongside other symptoms we discussed earlier, such as delayed fontanelle closure, a "square skull" (方颅), or "rachitic rosary" (puffy ribs) .

Muscle Weakness: The child may appear floppy, have a "waddling" gait, or be reluctant to walk due to low muscle tone caused by hypocalcemia .

Why Does This Happen?

When vitamin D is deficient, the bones do not mineralize (harden) properly and remain soft. When the child stands or walks, the weight of the body exerts pressure on these soft bones, causing them to bow or bend under the load .

Summary

If a child is taking vitamin D supplements and is within the normal age range for these alignments (bowed legs under 2, knock knees under 5), it is likely physiological. However, if the deformity is severe, worsening, or accompanied by other skeletal changes, it requires immediate medical evaluation.

## DeepSeek

Yes, bowed legs and knock knees can be signs of rickets, which is the severe, prolonged form of vitamin D deficiency in growing children. However, the relationship depends on the child's age and the underlying cause.

- Bowed legs (genu varum) – A classic sign of rickets, especially in toddlers and young children. In rickets, weakened, poorly mineralized leg bones bend under weight-bearing stress. However, mild bowing is also normal in infants and toddlers up to around 18–24 months, then typically resolves.

- Knock knees (genu valgum) – Also seen in rickets, particularly in older children (e.g., 3–6 years). Similarly, mild knock-knees can be a normal physiological variant in preschool-aged children, usually self-correcting by age 7–8.

Distinguishing rickets-related deformities:

- Rickets bowing or knock-knees is often progressive, symmetric, and accompanied by other signs (e.g., widened wrists/ankles, rachitic rosary, growth delay, low calcium/phosphate, elevated alkaline phosphatase).

- Physiological bowing/knock-knees is typically mild, age-appropriate, and not associated with other rickets features.

Conclusion: While these leg deformities are classic skeletal signs of rickets, not every child with bowed legs or knock knees has vitamin D deficiency. Evaluation (physical exam, wrist/ankle X-rays, serum 25(OH)D, calcium, phosphorus, alkaline phosphatase) is needed to confirm rickets. Early vitamin D deficiency may cause no leg deformities at all.

## Doubao

Yes, bowed legs and knock knees are typical skeletal deformity signs of severe vitamin D deficiency and nutritional rickets in young children. Insufficient vitamin D impairs calcium absorption and bone mineralization, making leg bones soft and easily deformed under body weight. However, slight leg curvature can also be a normal physiological developmental stage in toddlers. Persistent, obvious bowed legs or knock knees usually indicate rickets caused by long-term vitamin D deficiency.

# Question 18. Are flared ribs or pigeon chest related to vitamin D deficiency?

## ChatGPT

Yes, flared ribs (rachitic rosary) and pigeon chest (pectus carinatum) are skeletal abnormalities associated with rickets caused by prolonged vitamin D deficiency:

- Flared ribs / rachitic rosary – Enlargement of the costochondral junctions of the ribs, giving a bead-like appearance along the ribcage.

- Pigeon chest / pectus carinatum – Protrusion of the sternum due to weakened bones and abnormal bone growth.

These signs indicate impaired bone mineralization from insufficient vitamin D, calcium, or phosphorus, typically appearing after the deficiency has been ongoing for some time.

## Gemini

Yes, both flared ribs and pigeon chest (pectus carinatum) are classic skeletal manifestations of nutritional rickets, which is caused by severe and prolonged vitamin D deficiency.

When a child’s body lacks vitamin D, the bones do not mineralize (harden) properly. Because the ribs remain soft and pliable, they become susceptible to being pulled and reshaped by the constant mechanical forces of breathing and muscle attachments.

1. Harrison’s Groove (Rib Flaring)

Flared ribs in a child with rickets often present as a horizontal indentation or "trough" at the lower border of the chest, known as Harrison’s Groove.

The Cause: Every time a child breathes, the diaphragm (the main breathing muscle) pulls on the lower ribs.

The Result: If the ribs are soft due to deficiency, this constant pulling actually sucks the ribs inward at the point of attachment, causing the very bottom edges of the rib cage to "flare" outward.

2. Pigeon Chest (Pectus Carinatum)

A "pigeon chest" occurs when the sternum (breastbone) is pushed outward, making the chest look pointed.

The Cause: This is often a secondary effect of the ribs softening. As the sides of the chest wall soften and collapse inward or develop grooves, the breastbone is forced forward to compensate for the change in thoracic shape.

The Result: The chest loses its normal rounded shape and takes on a triangular or "pigeon-like" appearance.

3. The Rachitic Rosary

Often appearing alongside flared ribs is the Rachitic Rosary, which provides further evidence of vitamin D deficiency.

These are visible or feelable "beads" or lumps located where the rib bone meets the cartilage (the costochondral junction).

These bumps occur because the body is trying to grow bone, but because it can't harden the tissue, the cartilage simply expands and piles up, creating a string-of-beads effect along the chest.

4. Is it reversible?

Early Treatment: If vitamin D and calcium deficiencies are corrected early while the child is still growing rapidly, the chest deformities can often improve or even resolve completely as the bones harden in a more normal position.

Late Treatment: If the bones harden (calcify) in a deformed position after the child has finished their major growth spurts, the physical changes may become permanent, though their progression will stop.

Summary

If you notice a child’s ribs appearing to turn outward at the bottom or the breastbone protruding significantly, it is a sign that the rib cage is not structurally sound. A pediatrician should evaluate the child for rickets, typically using a combination of blood tests (25-hydroxyvitamin D, Calcium, ALP) and potentially a chest X-ray.

## Qianwen

Yes, flared ribs (often called the "rachitic rosary") and pigeon chest (pectus carinatum) are specific clinical signs of the active phase of rickets caused by vitamin D deficiency.

While mild chest asymmetry can sometimes be a congenital issue, these specific deformities are classic indicators of pathological bone softening due to metabolic bone disease.

The Rachitic Rosary (Flared Ribs)

This is one of the most diagnostic signs of rickets.

Mechanism: Vitamin D deficiency leads to an accumulation of unmineralized bone tissue (osteoid) at the growth plates. This occurs at the junction where the hard rib bone meets the soft cartilage.

Appearance: This accumulation causes palpable and visible swellings along the sides of the chest, resembling a string of beads. This is distinct from simple "rib flare" (everted ribs) which can sometimes be seen in thin, healthy children.

Pigeon Chest (Pectus Carinatum)

Pigeon chest is characterized by a protrusion of the sternum (breastbone).

Mechanism: In rickets, the rib bones soften. As the child breathes, the negative pressure exerted by the diaphragm and intercostal muscles pulls the soft lower ribs inward (sometimes causing Harrison's groove). Simultaneously, the unmineralized upper ribs push the sternum forward.

Appearance: The chest takes on a keel-like shape, similar to the breast of a bird.

Differentiating from Congenital Defects

It is important to distinguish these from congenital chest deformities which are present at birth and are not related to nutrition:

Congenital Pigeon Chest: Often isolated, symmetric, and does not progress rapidly. It is usually due to genetic or developmental factors unrelated to vitamin D.

Rickets-Induced Deformity: These usually develop or worsen rapidly during periods of high growth velocity (such as infancy). They are almost always accompanied by other skeletal signs, such as delayed fontanelle closure, square skull, or the rachitic rosary.

Clinical Significance

Because these deformities involve the structural shape of the chest, they can, in severe cases, restrict lung expansion and affect respiratory function. However, if identified early and treated with vitamin D and calcium, further progression can be halted, though the physical deformity itself may require time to remodel or, in severe cases, surgical intervention.

## DeepSeek

Yes, flared ribs (often referring to the rachitic rosary or flaring at the lower rib cage) and pigeon chest (pectus carinatum) can both be related to vitamin D deficiency as part of the skeletal manifestations of rickets.

- Flared ribs / rachitic rosary – In rickets, poor mineralization at the costochondral junctions (where ribs meet cartilage) causes visible swelling and prominence. Flaring of the lower rib cage (also called Harrison's groove) may occur due to diaphragmatic pull on softened ribs, creating a horizontal groove.

- Pigeon chest (pectus carinatum) – While more commonly congenital or idiopathic, a protrusion of the sternum and ribs can also occur in rickets due to generalized bone softening and muscle pull on the chest wall. It is a less specific but well-recognized sign in severe, long-standing rickets.

Important notes:

- These chest deformities are late signs of significant, untreated rickets, not early deficiency.

- They can also result from other conditions such as congenital chest wall abnormalities, connective tissue disorders, or chronic respiratory diseases.

- When due to rickets, they are typically accompanied by other signs (e.g., leg deformities, widened wrists, growth delay, elevated alkaline phosphatase).

Therefore, while flared ribs or pigeon chest should raise suspicion for rickets in a child with risk factors, diagnosis requires confirmatory laboratory tests (serum 25(OH)D, calcium, phosphorus, alkaline phosphatase) and sometimes X-rays. Not all such chest findings are caused by vitamin D deficiency.

## Doubao

Yes. Flared ribs and pigeon chest are classic skeletal deformities caused by severe, long-term vitamin D deficiency and rickets in children. Lack of vitamin D leads to poor bone mineralization, softening of the rib cage and costochondral junctions. The softened chest bones are deformed by respiratory muscle pull and physical pressure, resulting in rib flare, pigeon chest, and even Harrison's groove. These are typical late-stage skeletal manifestations of nutritional rickets.

# Question 19. Could frequent leg pain, muscle weakness, or fatigue in a child be related to vitamin D deficiency?

## ChatGPT

Yes, frequent leg pain, muscle weakness, or fatigue in a child can be related to vitamin D deficiency:

- Leg pain – Often occurs in the lower limbs due to softening of bones (osteomalacia) and stress on weakened skeletal structures.

- Muscle weakness – Vitamin D is important for muscle function; deficiency can reduce muscle strength and tone.

- Fatigue – Low vitamin D levels may contribute to low energy, irritability, and general tiredness.

While these symptoms are nonspecific and can have other causes, when combined with risk factors or signs of bone changes, they may suggest vitamin D deficiency and warrant testing.

## Gemini

Yes, frequent leg pain, muscle weakness, and fatigue are common—though often overlooked—symptoms of vitamin D deficiency in children. While many people associate vitamin D only with "bone" issues, it plays a vital role in how muscles and nerves function.

When a child is deficient, these symptoms usually occur for the following biological reasons:

1. Bone Pain (Osteomalacia)

Unlike the sharp pain of a fracture, vitamin D deficiency causes a condition called osteomalacia, which is a softening of the bones.

The Sensation: This is often described as a "dull, deep, aching pain" that is felt in the bones themselves rather than the joints.

Location: It most commonly affects the legs (shins and thighs), lower back, and hips, as these are the weight-bearing areas of the skeleton.

Timing: The pain may worsen at night or after physical activity, often being mistaken for simple "growing pains."

2. Muscle Weakness and "Floppiness"

Vitamin D receptors are located directly on muscle cells. Vitamin D is required for muscles to contract and relax efficiently.

Hypotonia: In infants and toddlers, a lack of vitamin D can lead to "floppy" muscles (hypotonia).

Functional Weakness: Older children may have difficulty with tasks like climbing stairs, jumping, or getting up from a sitting position on the floor.

Waddling Gait: In more advanced cases (rickets), a child may develop a distinctive "waddling" walk because their pelvic muscles are too weak to stabilize their hips properly.

3. Chronic Fatigue

Vitamin D is involved in mitochondrial function—the part of the cell that generates energy.

A child with low levels may seem unusually tired, lack the energy to play with peers, or want to be carried frequently.

Research has shown that correcting vitamin D deficiency can significantly improve energy levels and reduce daytime sleepiness in both children and adults.

How to Distinguish from "Growing Pains"

It can be difficult to tell the difference between normal growing pains and vitamin D deficiency. However, there are a few clues:

Summary

If a child consistently complains of leg pain, seems "clumsy" or weak, or is always exhausted, it is worth discussing a 25-hydroxyvitamin D [25(OH)D] blood test with a pediatrician. Unlike many other causes of leg pain, vitamin D deficiency is easily and safely treatable with proper supplementation.

Expert Guide: Are these symptoms appearing alongside any other changes, such as a change in how your child walks or a recent growth spurt?

## Qianwen

Yes, frequent leg pain, muscle weakness, and fatigue are common signs of vitamin D deficiency and the active phase of rickets.

Unlike the skeletal deformities discussed previously (which are structural), these symptoms reflect the physiological impact of the deficiency on the muscles and nerves.

Muscle Weakness and Fatigue

Muscle weakness is considered one of the most significant and yet often overlooked features of vitamin D deficiency.

Proximal Myopathy: Vitamin D receptors are present in muscle tissue. Deficiency leads to hypotonia (low muscle tone) and weakness, particularly in the proximal muscles (hips and thighs).

Clinical Presentation:

"Waddling" Gait: Children may walk with a wide-based, waddling gait due to weak hip muscles.

Developmental Delays: It can cause a delay in motor milestones, such as sitting or walking later than expected.

General Fatigue: Children may appear listless, lethargic, or easily exhausted compared to their peers.

Leg Pain (Bone Pain)

While children often complain of "growing pains," specific types of pain can signal rickets.

Location and Nature: The pain is typically diffuse, deep, and located in the legs (thighs and shins). It is often described as an ache rather than a sharp pain.

Mechanism: This pain results from the softening of the bones (osteomalacia) and the stress placed on the bone periosteum as the bone attempts to remodel under the body's weight.

Aggravating Factors: Unlike typical growing pains which often occur at night, rickets-related pain can be exacerbated by physical activity, weight-bearing (walking/standing), or even palpation of the bone.

Differentiating from Growing Pains

It can be difficult to distinguish these symptoms from benign "growing pains," but rickets-related symptoms usually have specific characteristics:

Persistence: The pain and weakness are persistent and do not resolve with rest.

Accompaniment: They usually occur alongside the skeletal signs previously mentioned (such as delayed fontanelle closure, rachitic rosary, or bow legs).

Tenderness: The bones may be tender to the touch.

These symptoms indicate a systemic issue affecting both the skeletal and muscular systems. If a child presents with unexplained fatigue and leg pain, assessing vitamin D status is a standard clinical step.

## DeepSeek

Yes, frequent leg pain, muscle weakness, and fatigue in a child can be related to vitamin D deficiency, even before obvious bone deformities appear. Here’s how:

- Leg pain – Vitamin D deficiency can cause a dull, aching bone pain (often in the thighs, shins, or knees) due to poor bone mineralization or secondary hyperparathyroidism. Children may complain of "growing pains" that are actually a manifestation of deficiency.

- Muscle weakness – Vitamin D receptors are present on muscle cells. Deficiency impairs muscle function, leading to proximal muscle weakness (e.g., difficulty climbing stairs, getting up from the floor, or a waddling gait). In infants, hypotonia (low muscle tone) may be seen.

- Fatigue – Generalized lethargy, decreased activity, and easy tiring are common but nonspecific symptoms. They may result from muscle inefficiency, chronic bone pain, or associated metabolic disturbances (e.g., hypocalcemia).

Important distinctions:

- These symptoms are not specific to vitamin D deficiency. Many other conditions (e.g., juvenile idiopathic arthritis, neuromuscular disorders, chronic fatigue syndrome, sleep disorders, anemia, or psychological factors) can cause similar complaints.

- Mild deficiency may cause no symptoms at all, while significant deficiency often produces these symptoms before overt rickets develops.

- A child with persistent leg pain, weakness, or fatigue—especially with other risk factors for deficiency—should have serum 25-hydroxyvitamin D measured, along with calcium, phosphorus, alkaline phosphatase, and creatine kinase.

Thus, while vitamin D deficiency is one possible cause, clinical evaluation and testing are necessary to confirm or rule it out.

## Doubao

Yes. Vitamin D is essential for regulating muscle function and neuromuscular excitability. Vitamin D deficiency in children can cause muscle weakness, easy fatigue, unexplained intermittent leg pain, limb soreness, and even delayed motor development. These symptoms are common non-skeletal manifestations of vitamin D deficiency.

# Question 20. Can vitamin D deficiency affect a child's height growth?

## ChatGPT

Yes, vitamin D deficiency can affect a child’s height growth.

- Vitamin D is essential for calcium and phosphorus absorption, which are critical for bone mineralization and linear growth.

- Deficiency can lead to weakened or soft bones, delayed skeletal development, and conditions like rickets, all of which may slow a child’s height gain.

- Chronic deficiency during key growth periods can result in short stature or growth retardation compared to peers.

Early detection and supplementation help ensure normal growth and prevent long-term skeletal issues.

## Gemini

Yes, vitamin D deficiency can significantly impact a child's height. Because vitamin D is the primary regulator of calcium and phosphorus—the two minerals that make up the "bricks and mortar" of the skeleton—a deficiency strikes at the very mechanism of growth.

Here is how it affects a child's height through different biological pathways:

1. Failure of the Growth Plates

Height growth occurs at the epiphyseal plates (growth plates) located at the ends of long bones.

Normal Growth: In a healthy child, cartilage in the growth plates is constantly being created and then hardened (mineralized) into bone.

With Deficiency: If there isn't enough vitamin D to facilitate calcium deposition, this cartilage fails to harden. Instead of the bone elongating properly, the growth plate becomes thick, disorganized, and "spongy." This results in slower linear growth and shorter bones.

2. Physical Deformities (Loss of "Effective Height")

Even if the bone tissue is growing, the quality of that bone affects the child's stature.

Bowing: When bones are soft, they bend under the weight of the body (O-shaped or X-shaped legs). This curvature physically shortens the limb, making the child appear shorter than they would be if their legs were straight.

Spinal Changes: Severe deficiency can lead to softening of the vertebrae, causing a curved spine (kyphosis or scoliosis), which further reduces standing height.

3. Growth Retardation and Stunting

Chronic vitamin D deficiency is a known cause of nutritional stunting.

The Vicious Cycle: Deficiency often leads to poor appetite and general muscle weakness. If a child is not active or eating well due to the systemic effects of low vitamin D, their overall growth velocity slows down.

Delayed Milestones: Children with deficiency often reach growth milestones (like the "growth spurt") later than their peers and may never fully catch up to their genetic height potential if the deficiency is not corrected before the growth plates close at the end of puberty.

4. Is the Impact Reversible?

Early Intervention: If the deficiency is caught and treated early, "catch-up growth" is possible. Once vitamin D and calcium levels are restored, the growth plates can resume normal mineralization, and many bone curvatures will straighten out as the child grows.

Late Intervention: If the deficiency persists until the end of adolescence when the growth plates "fuse" (turn into solid bone), the loss in height and certain skeletal deformities may become permanent.

Summary

Vitamin D is essentially the "on-switch" for bone elongation. Without it, the body has the genetic blueprint for height but lacks the hardened material necessary to build the structure.

## Qianwen

Yes, vitamin D deficiency can significantly affect a child's height growth, potentially leading to short stature and delayed development.

While genetics determine the maximum potential height, vitamin D deficiency acts as a limiting factor that prevents a child from reaching that potential by disrupting the normal process of bone elongation.

Impairment of Bone Elongation

Children grow in height primarily through the growth plates (epiphyseal plates) located at the ends of their long bones.

Disruption of Mineralization: Vitamin D is essential for absorbing calcium and phosphorus. Without adequate levels, the cartilage in the growth plates cannot calcify (harden) into bone properly.

Growth Plate Expansion: Instead of the bone lengthening efficiently, the unmineralized cartilage accumulates, causing the growth plates to widen. This results in a condition called "rachitic growth retardation," where the overall height gain is significantly slowed down .

Reduction in Growth Velocity

Research indicates a direct correlation between vitamin D levels and growth speed.

Slowed Rate: Children with severe vitamin D deficiency may grow at a rate of less than 4-5 cm per year, which is below the normal average for most age groups.

Height Lag: Long-term deficiency often leads to a child falling behind the growth curve and presenting with a height that is lower than the average for their peers .

Impact on Bone Density and Structure

Even if the child continues to grow, the quality of the growth is compromised.

Low Bone Density: Deficiency leads to lower bone mineral density, making the bones weaker and more prone to micro-fractures or bending (as mentioned in previous questions regarding bow legs) .

Structural Deformities: As the child gains weight, the soft, growing bones may deform under pressure, which can further complicate posture and skeletal health .

Potential for Catch-Up Growth

The impact on height is not necessarily permanent if addressed early.

Reversibility: Once vitamin D and calcium levels are restored through supplementation and diet, the growth plates can resume normal mineralization.

Catch-Up: Many children experience a period of "catch-up growth" where their growth velocity increases above normal to compensate for the lost time, provided the epiphyseal plates have not yet closed .

## DeepSeek

Yes, vitamin D deficiency can affect a child's height growth. The impact occurs through several mechanisms:

- Impaired bone mineralization – Vitamin D deficiency reduces calcium and phosphorus absorption, leading to poorly mineralized growth plate cartilage and weakened bone. This directly disrupts normal linear growth.

- Growth plate abnormalities – In rickets, the growth plates (epiphyseal plates) become widened, disorganized, and fail to undergo proper mineralization and ossification, resulting in stunted or slowed height gain.

- Skeletal deformities – Bowed legs, knock knees, or spinal curvature can reduce standing height mechanically, even if limb length is partially maintained.

- Secondary hyperparathyroidism – Chronic deficiency leads to elevated parathyroid hormone, which can impair growth hormone signaling and increase bone turnover, further hindering statural growth.

Clinical evidence:

- Children with untreated rickets often have height below the 5th percentile for age.

- Studies show that correcting vitamin D deficiency (along with calcium and phosphorus) leads to catch-up growth, though some height deficit may persist if treatment is delayed.

Important considerations:

- Mild or asymptomatic deficiency without rickets may not significantly affect height.

- Height impairment is usually a late consequence of severe, prolonged deficiency, not an early sign.

- Many other factors (genetics, nutrition, hormones, chronic diseases) affect height, so vitamin D deficiency is just one possible cause.

Therefore, while vitamin D deficiency can impair height growth, it does so primarily through advanced rickets. Routine supplementation and early treatment of deficiency support normal linear growth.

## Doubao

Yes. Vitamin D deficiency hinders calcium and phosphorus absorption, impairs bone mineralization and epiphyseal growth plate development in children. It slows skeletal growth, delays bone maturation, and may lead to short stature or permanent growth restriction if the deficiency lasts long, especially during early childhood.

# Question 21. Should vitamin D deficiency be investigated in a child with recurrent fractures?

## ChatGPT

Yes, vitamin D deficiency should be investigated in a child with recurrent fractures.

- Low vitamin D levels impair calcium and phosphorus absorption, leading to weakened bones (osteomalacia) that are more prone to fractures, even with minor trauma.

- Recurrent fractures, especially in unusual locations or with minimal injury, are a red flag for underlying metabolic bone disease, including vitamin D deficiency.

- Evaluation typically includes serum 25-hydroxyvitamin D, calcium, phosphorus, alkaline phosphatase, and sometimes imaging to assess bone density or deformities.

Identifying and correcting deficiency is crucial to prevent further fractures and support healthy bone development.

## Gemini

Yes, for children with recurrent or low-energy fractures (fractures occurring from a fall at standing height or less), investigating vitamin D deficiency is standard medical practice.

The relationship between vitamin D and bone strength is critical, as a deficiency leads to two major issues that increase fracture risk:

Poor Mineralization: Without enough vitamin D, the body cannot absorb calcium and phosphorus effectively. This leads to osteomalacia, a condition where the "new" bone being formed is soft and rubbery rather than hard and mineralized. These soft bones are much more likely to bend or break under normal stress.

Secondary Hyperparathyroidism: When vitamin D is low, the body’s blood calcium levels begin to drop. To compensate, the parathyroid glands work overtime, releasing Parathyroid Hormone (PTH). This hormone signals the body to "leach" calcium out of the existing bones to keep blood levels stable, effectively thinning the bones from the inside out and making them brittle.

The Medical Investigation

If a child has a history of multiple fractures, a pediatrician or pediatric endocrinologist will typically order a "Bone Metabolism Panel" which includes:

25-hydroxyvitamin D [25(OH)D]: To determine overall vitamin D stores.

Serum Calcium and Phosphorus: To see if the "building blocks" are available in the blood.

Alkaline Phosphatase (ALP): An enzyme that rises when bone turnover is abnormal.

Parathyroid Hormone (PTH): To check if the body is in "emergency mode," pulling calcium from the bones.

DEXA Scan: In some cases, a bone density scan may be used to measure exactly how "thick" or "thin" the child’s bones are compared to other children of the same age and sex.

Summary

Recurrent fractures are often a "red flag" that the skeletal system is not receiving enough minerals. Correcting a vitamin D deficiency can significantly improve bone density and decrease the likelihood of future injuries as the child continues to grow.

## Qianwen

Yes, vitamin D deficiency must be investigated in any child with recurrent fractures. It is a critical diagnostic step because recurrent fractures can signal underlying metabolic bone diseases, with rickets (caused by vitamin D deficiency) being one of the most common causes .

The connection between vitamin D and fracture risk is rooted in bone quality and muscle function:

Compromised Bone Quality (Osteomalacia)

Vitamin D is essential for the body to absorb calcium, which is the primary building block of hard, strong bones.

Bone Softening: Without adequate vitamin D, bones fail to mineralize properly. In children, this condition is known as rickets. The bones become soft, pliable, and structurally weak .

Increased Susceptibility: Weak bones cannot withstand normal mechanical stress or minor trauma. Instead of remaining rigid, they are prone to bending or snapping, leading to fractures that occur disproportionately easily .

Muscle Weakness and Fall Risk

As mentioned in previous discussions regarding leg pain and fatigue, vitamin D deficiency causes muscle weakness (proximal myopathy).

Instability: Weak muscles impair balance and coordination, leading to a higher frequency of falls and accidents.

Cycle of Injury: Increased fall risk combined with weakened bones creates a "perfect storm" for recurrent fractures .

Clinical Evaluation Protocol

When a child presents with multiple fractures, medical evaluation typically includes:

Laboratory Screening: Measuring serum calcium, phosphorus, parathyroid hormone (PTH), and 25-hydroxyvitamin D levels to assess metabolic bone health .

Radiographic Assessment: X-rays are used to look for specific signs of rickets, such as widened growth plates, bone density changes, or "greenstick" fractures (incomplete fractures common in soft bones) .

Identifying and treating vitamin D deficiency is vital not only to prevent future fractures but also to address the underlying cause of the bone fragility.

## DeepSeek

Yes, vitamin D deficiency should be investigated in a child with recurrent fractures, although it is not the most common cause. Here’s why:

Rationale for investigation:

- Vitamin D deficiency leads to poor bone mineralization (osteomalacia in older children, rickets in younger ones), reducing bone strength and increasing fracture risk.

- Even without overt rickets, chronic deficiency can cause low bone mineral density, making bones more susceptible to fractures from minor trauma.

- Recurrent or low-trauma fractures in a child warrant a workup for metabolic bone disease, including vitamin D deficiency.

However, important caveats:

- Most childhood fractures occur in healthy, active children with normal vitamin D status. Recurrent fractures are more often due to other conditions such as:

- Osteogenesis imperfecta (most common pathological cause)

- Juvenile osteoporosis (idiopathic or secondary)

- Calcium deficiency

- Chronic illnesses (e.g., cerebral palsy, muscular dystrophy, renal disease)

- Non-accidental trauma (child abuse) – must always be considered

- Vitamin D deficiency alone rarely causes recurrent fractures unless severe and prolonged.

Recommended evaluation for a child with recurrent fractures:

- Serum 25-hydroxyvitamin D, calcium, phosphorus, alkaline phosphatase, parathyroid hormone

- Bone density (DXA) if clinically indicated

- Consider genetic testing for osteogenesis imperfecta if other causes excluded

- Dietary and sun exposure history

- Assessment for other risk factors (malabsorption, medications, chronic disease)

Conclusion: Yes, vitamin D deficiency should be part of the initial laboratory workup, but it should not be assumed as the cause without ruling out other more common or serious conditions. Even if vitamin D deficiency is found, further evaluation may still be needed to explain recurrent fractures.

## Doubao

Yes. Recurrent fractures in children are closely linked to vitamin D deficiency. Vitamin D insufficiency reduces calcium absorption, weakens bone mineralization and lowers bone density, making bones fragile and prone to repeated fractures. Besides nutritional deficiency, it is necessary to check serum 25-hydroxyvitamin D, calcium and phosphorus levels, and rule out other bone metabolic diseases at the same time.

# Question 22. What signs suggest that a child may have rickets rather than simple vitamin D deficiency?

## ChatGPT

Signs that suggest a child may have rickets (the clinical disease) rather than just vitamin D deficiency (biochemical low levels) include physical and skeletal abnormalities:

1. Bone deformities

- Bowed legs (genu varum) or knock knees (genu valgum)

- Pigeon chest (pectus carinatum) or flared ribs / rachitic rosary

2. Delayed growth and development

- Slower height gain or short stature

- Delayed closure of the fontanelle in infants

3. Dental issues

- Delayed tooth eruption or enamel defects

4. Skeletal pain or tenderness

- Particularly in the legs, spine, or joints

5. Muscle weakness or hypotonia

In contrast, simple vitamin D deficiency may be asymptomatic or present only with subtle early signs like irritability, fatigue, or minor muscle weakness without visible bone deformities. Rickets represents the advanced, symptomatic stage of deficiency.

## Gemini

While vitamin D deficiency refers to a biochemical state (low blood levels), rickets is the clinical disease that occurs when that deficiency becomes severe enough to cause structural changes in the skeleton.

The following signs suggest that the condition has progressed beyond simple deficiency into clinical rickets:

1. Skeletal Deformities (The Most Visible Signs)

In rickets, the bones become soft and "rubbery," leading to characteristic physical changes:

Lower Limb Bowing: As a child begins to walk, the weight of their body causes the soft leg bones to bend. This results in Genu Varum (O-legs/bow-legs) or Genu Valgum (X-legs/knock-knees).

Square Head (Caput Quadratum): The skull bones may soften and then thicken at the front and sides, giving the head a boxy or "squared-off" appearance.

Pigeon Chest (Pectus Carinatum): The breastbone may be pushed forward, resembling the chest of a bird.

2. Widening of the Growth Plates

Rickets specifically affects the ends of the long bones where growth occurs. You may notice:

Hand/Foot "Bracelet" Sign: The wrists and ankles appear unusually thick or swollen. This is not soft tissue swelling; it is actually the widening of the bone ends as they fail to mineralize and instead spread outward.

Rachitic Rosary: You can feel or see rounded "beads" along the ribs where the bone meets the cartilage. These bumps look like a string of beads under the skin.

3. Indentations and Grooves

Harrison’s Groove: A horizontal indentation or "trough" that develops along the lower border of the chest. This happens because the softened ribs are being pulled inward by the attachment of the diaphragm every time the child breathes.

4. Delayed Physical Milestones

A child with rickets often shows significant delays in motor development compared to simple deficiency:

Delayed Walking: Due to bone pain and muscle weakness (hypotonia).

Late Closing of the Fontanelle: The "soft spot" on the top of an infant's head may remain open well past 18 months of age.

Late Teething: Permanent or primary teeth may be very slow to emerge and may have weak enamel.

5. Definitive Radiographic Changes (X-ray)

While blood tests show the deficiency, an X-ray is the only way to confirm rickets. A radiologist looks for:

Cupping and Fraying: Instead of a crisp, straight line at the end of the bone, the edges look jagged (frayed) and concave (cupped like a saucer).

Widening of the Physis: The space between the bone shaft and the end of the bone appears much wider than normal.

Summary

If you only see low blood levels on a lab report, it is deficiency. If you see bowed legs, thickened wrists, or rib "beading," the child has progressed to rickets. At this stage, treatment usually requires higher doses of vitamin D and calcium, along with orthopedic monitoring to ensure the bones straighten correctly as they harden.

## Qianwen

Simple vitamin D deficiency refers to a biochemical state of low vitamin D levels, whereas rickets is the clinical disease that develops when this deficiency becomes severe and prolonged enough to cause structural bone damage.

Therefore, the presence of physical deformities and radiographic changes is what distinguishes rickets from simple deficiency.

Visible Bone Deformities

The hallmark of rickets is the softening of the bone matrix, which leads to visible structural changes under the stress of gravity and muscle movement.

Cranial Changes:

Craniotabes: Softening of the skull bones, which may feel like a ping-pong ball when pressed (common in infants under 3 months) .

Square Skull (Frontal Bossing): Accumulation of bone tissue on the forehead and parietal bones creates a squared appearance .

Thoracic Changes:

Rachitic Rosary: Visible and palpable bead-like enlargements at the costochondral junctions (where ribs meet cartilage) .

Harrison's Groove: A horizontal indentation along the lower chest caused by the diaphragm pulling on softened ribs .

Limb Deformities:

Bowing of Legs: Genu varum (O-legs) or Genu valgum (X-legs) caused by the weight of the child bending the softened long bones .

Wrist Swelling: Enlargement of the metaphyses at the wrists and ankles, resembling bracelets or "cuffs" .

Delayed Developmental Milestones

While simple deficiency might cause vague symptoms like irritability or sweating, rickets often leads to tangible delays in physical development due to muscle weakness and bone structural integrity issues.

Delayed Fontanelle Closure: The soft spots on the baby's head remain open longer than the normal age of 18–24 months .

Delayed Tooth Eruption: Incisors or molars may appear significantly later than average .

Motor Delays: Postponed walking or a persistent waddling gait due to hip muscle weakness .

Radiographic Evidence (X-ray)

Even if visible deformities are subtle, X-rays can confirm rickets by showing defects in the growth plates (epiphyses) that are not present in simple deficiency.

Fraying and Cupping: The ends of the long bones (metaphyses) appear frayed, irregular, and cup-shaped on an X-ray .

Widened Growth Plates: The space between the bone shaft and the bone end is abnormally wide due to the accumulation of unmineralized cartilage .

Summary: If a child has low vitamin D levels but a normal physical exam and normal X-rays, they have vitamin D deficiency. If that deficiency has resulted in the physical signs listed above, the diagnosis is rickets.

## DeepSeek

The following signs suggest that a child has rickets (the advanced, skeletal manifestation of prolonged vitamin D deficiency) rather than simple, uncomplicated vitamin D deficiency:

Bone deformities (unique to rickets)

- Leg deformities – Bowed legs (genu varum) in toddlers, knock knees (genu valgum) in older children

- Widened wrists and ankles – Palpable flaring due to unmineralized osteoid at growth plates

- Rachitic rosary – Beading or swelling at the costochondral junctions of the ribs

- Harrison's groove – Horizontal depression along the lower rib cage from diaphragmatic pull on softened ribs

- Pigeon chest (pectus carinatum) – Protruding sternum

- Craniotabes – Softening of the skull bones (usually in infants); a ping-pong ball-like sensation when pressing on the parietal or occipital bones

- Delayed fontanelle closure (beyond 18–24 months)

- Skull bossing – Frontal or parietal prominence

Radiographic findings (gold standard for rickets diagnosis)

- Cupping, fraying, and widening of the metaphyses (growth plates), especially at wrists, knees, and ankles

- Decreased bone density (osteopenia)

- Subperiosteal erosions in severe cases

Biochemical markers (more severe than simple deficiency)

- Markedly elevated alkaline phosphatase (often >1000 IU/L) – reflects osteoblast activity due to unmineralized osteoid

- Low or low-normal serum calcium (simple deficiency often maintains normal calcium)

- Low serum phosphorus (hypophosphatemia is common in rickets but not always present)

- Elevated parathyroid hormone (secondary hyperparathyroidism, often marked)

Growth and developmental signs

- Linear growth stunting (height below 3rd–5th percentile)

- Delayed motor milestones (e.g., late walking, waddling gait)

- Muscle weakness (proximal, leading to Gowers' sign in severe cases)

Key distinction:

- Simple vitamin D deficiency – Low 25(OH)D, normal or mildly elevated alkaline phosphatase, no bone deformities, normal radiographs, often asymptomatic or only mild symptoms (e.g., irritability, sweating).

- Rickets – All of the above plus bone deformities, rachitic changes on X-ray, and significantly elevated alkaline phosphatase.

In practice, a child with isolated low 25(OH)D but no physical or radiographic findings has simple deficiency. Once deformities or radiological changes appear, the diagnosis is rickets.

## Doubao

Children with rickets (not just simple vitamin D deficiency) typically show these distinctive signs: 1. Obvious skeletal deformities: bowed legs, knock knees, pigeon chest, rib flare, Harrison's groove, softened skull bones. 2. Delayed closure of fontanelles, delayed tooth eruption and poor tooth enamel development. 3. Marked muscle weakness, hypotonia, delayed sitting, standing and walking. 4. Persistent nervous symptoms plus slow physical growth and poor height gain. 5. Abnormal blood biochemistry and typical X-ray changes of bone epiphyses. Simple vitamin D deficiency often has only mild non-specific symptoms without obvious skeletal or radiological abnormalities, while rickets presents clear structural bone damage.

# Question 23. Which children need a 25-hydroxyvitamin D test?

## ChatGPT

Children who may need a 25-hydroxyvitamin D [25(OH)D] test include:

1. At-risk infants and children

- Exclusively breastfed infants without supplementation

- Premature or low-birth-weight infants

- Children with darker skin living in regions with limited sunlight

2. Children with clinical signs of deficiency or rickets

- Bone deformities (bowed legs, knock knees, flared ribs)

- Delayed growth, delayed tooth eruption, delayed closure of fontanelle

- Recurrent fractures, bone pain, or muscle weakness

3. Children with medical conditions affecting vitamin D metabolism or absorption

- Liver disease, kidney disease, intestinal malabsorption (e.g., celiac disease, cystic fibrosis)

4. Children on long-term medications affecting vitamin D

- Antiepileptics, glucocorticoids, or other drugs that interfere with vitamin D metabolism

5. Children with limited sun exposure or poor dietary intake

- Those living in northern latitudes, during winter, or in heavily polluted areas

- Children with very restricted diets

Testing helps identify deficiency early and guide appropriate supplementation or treatment.

## Gemini

Not every child requires a blood test, as many pediatric organizations favor universal supplementation over universal testing. However, a 25-hydroxyvitamin D [25(OH)D] test is medically necessary for children who fall into specific high-risk or symptomatic categories.

Medical guidelines generally recommend testing for the following groups:

1. Children with Clinical Signs of Bone Disease

Any child showing physical symptoms that suggest their bone mineralization is compromised needs a test to confirm the underlying cause:

Skeletal Deformities: Presence of bowed legs (O-legs), knock-knees (X-legs), or thickened wrists and ankles.

Growth Issues: Unexplained short stature or a significant drop in growth velocity.

Bone Pain: Frequent complaints of "aching" legs or back pain that interferes with activity.

Recurrent Fractures: Children who experience multiple fractures or "low-impact" breaks (breaking a bone from a simple fall).

2. Children with Chronic Malabsorption Issues

Because vitamin D is fat-soluble, children with conditions that affect the gut’s ability to absorb fat are at high risk:

Celiac Disease: Even if the child is on a gluten-free diet, their past gut damage may have depleted stores.

Inflammatory Bowel Disease (IBD): Such as Crohn’s disease or Ulcerative Colitis.

Cystic Fibrosis: Requires frequent monitoring as these children often need much higher doses to maintain normal levels.

3. Children with Organ Dysfunction

The liver and kidneys are the "processing plants" for vitamin D. If they are not working correctly, the body cannot use the vitamin D it receives:

Chronic Kidney Disease (CKD): The kidneys perform the final activation of vitamin D.

Chronic Liver Disease: The liver performs the first conversion step.

4. Children on Specific Long-term Medications

Certain medications speed up the breakdown of vitamin D in the body, essentially "burning through" the child's supply:

Anti-epileptic drugs: Such as Phenobarbital, Phenytoin, or Carbamazepine.

Systemic Glucocorticoids: Long-term oral steroid use (e.g., Prednisone for asthma or autoimmune issues).

Antifungals or HIV medications: Some specific treatments can interfere with vitamin D metabolism.

5. Highly Symptomatic "Non-Bone" Cases

While less common, doctors may test children exhibiting acute metabolic distress that could be linked to calcium/vitamin D imbalance:

Unexplained Seizures: To rule out hypocalcemia (low blood calcium).

Muscle Tetany or Spasms: Visible cramping or "locking" of the hands and feet.

Summary Checklist for Parents

Note: For a healthy, thriving child with a varied diet and consistent 400–600 IU supplementation, a blood test is rarely indicated. If you are concerned, a pediatrician will usually start by reviewing the child's diet and supplement history before deciding if a needle stick is necessary.

## Qianwen

Not all children require a 25-hydroxyvitamin D test. Routine screening is generally not recommended for healthy children who have no risk factors, no symptoms, and are adhering to regular vitamin D supplementation.

Testing is typically reserved for children with specific high-risk factors, suspected clinical symptoms, or those undergoing treatment monitoring.

Children with High-Risk Factors

These children are more prone to deficiency due to physiological factors or environmental limitations, making screening advisable:

Specific Physiological Stages: Premature infants, twins, low birth weight babies, and infants with rapid growth rates.

Intake and Absorption Issues: Children on long-term exclusive breastfeeding without regular vitamin D supplementation, or those with conditions affecting absorption (e.g., chronic diarrhea, liver diseases, or cystic fibrosis).

Insufficient Sun Exposure: Children with very limited outdoor activity, those who are housebound, or those living in regions with weak sunlight.

Children with Clinical Symptoms or Signs

When a child presents with the abnormalities discussed previously, testing becomes a critical step for diagnosis:

Skeletal and Motor Abnormalities: Presence of rickets signs (e.g., square skull, rachitic rosary, O/X-shaped legs), growth retardation, motor delays, or muscle weakness.

Unexplained Injuries: Occurrence of low-energy fractures (breaking bones from minor bumps) or recurrent fractures.

Increased Neuromuscular Excitability: Symptoms such as tetany (muscle spasms), convulsions, or unexplained night terrors and excessive sweating.

Monitoring During Treatment

For children already diagnosed with vitamin D deficiency or rickets and undergoing treatment, testing is standard for evaluating efficacy:

Efficacy Assessment: It is recommended to monitor levels every 3–4 months after starting treatment until the index reaches a suitable level (typically >50 nmol/L or >20 ng/mL).

Protocol Adjustment: Results guide the adjustment of dosage to prevent both inadequate treatment and vitamin D toxicity.

Summary: The 25-hydroxyvitamin D test is the "gold standard" for confirming deficiency.

If a child has none of the above conditions and follows a standard supplementation routine, frequent blood testing is usually unnecessary.

## DeepSeek

The following children should undergo serum 25-hydroxyvitamin D (25(OH)D) testing, according to pediatric guidelines and clinical consensus:

1. Children with signs or symptoms suggestive of vitamin D deficiency or rickets

- Bone pain, leg deformities (bowed legs/knock knees), widened wrists/ankles, rachitic rosary, pigeon chest

- Delayed fontanelle closure, craniotabes (infants)

- Muscle weakness, waddling gait, hypotonia

- Recurrent low-trauma fractures

- Unexplained growth failure or short stature

- Hypocalcemic seizures, tetany, or numbness/tingling

2. Children with significant risk factors

- Exclusively breastfed infants without adequate vitamin D supplementation, especially if mother is deficient

- Dark-skinned children living in high-latitude regions (>37°N or <37°S) or with minimal sun exposure

- Children with malabsorption disorders: celiac disease, Crohn's disease, cystic fibrosis, short bowel syndrome, chronic pancreatitis

- Children with chronic liver disease (e.g., biliary atresia, cirrhosis)

- Children with chronic kidney disease (stages 2–5)

- Children on long-term medications: anticonvulsants (phenytoin, carbamazepine, phenobarbital), glucocorticoids, antifungals (ketoconazole), cholestyramine, rifampin, antiretrovirals

- Children with obesity (BMI ≥95th percentile) – vitamin D sequestered in fat tissue

- Children with a history of bariatric surgery or intestinal resection

- Children with granulomatous diseases (sarcoidosis, tuberculosis) – risk of both deficiency and toxicity

3. Children with persistent nonspecific symptoms that could be due to deficiency

- Chronic fatigue, lethargy, or irritability without other explanation

- Frequent leg pain (diffuse, not joint-specific) or "growing pains" unresponsive to routine measures

- Poor sleep, excessive sweating (infants) – though not specific

4. Children with incidentally found biochemical abnormalities

- Hypocalcemia, hypophosphatemia, elevated alkaline phosphatase (for age)

- Elevated parathyroid hormone

- Low bone density on DXA scan

5. Children requiring baseline measurement before starting treatment

- Before initiating high-dose vitamin D therapy (to confirm deficiency and avoid unnecessary treatment)

- Prior to starting medications that affect vitamin D metabolism (for later comparison)

Routine screening (without specific risk factors or symptoms) is generally not recommended by most major pediatric societies (e.g., AAP, Endocrine Society), except in populations with very high prevalence of deficiency where public health screening programs may be considered.

Summary: Testing is indicated for symptomatic, high-risk, or biochemically suspicious children, not for asymptomatic, healthy, low-risk children with adequate sun exposure and dietary intake.

## Doubao

Children in the following groups need a 25-hydroxyvitamin D test: 1. Infants and young kids with obvious early deficiency symptoms: night sweating, irritability, poor sleep, occipital alopecia. 2. Children with skeletal deformities such as bowed legs, knock knees, pigeon chest and rib flare. 3. Premature infants, low-birth-weight infants, exclusively breastfed babies without vitamin D supplementation. 4. Kids with little sun exposure, living in northern areas, winter long-term indoor life or heavy air pollution areas. 5. Overweight/obese children and those with picky eating and unbalanced diet. 6. Children with gastrointestinal malabsorption, liver or kidney diseases. 7. Kids taking long-term antiepileptic drugs, glucocorticoids and other special medications. 8. Children with recurrent leg pain, muscle weakness, slow height growth and recurrent fractures.

# Question 24. Should healthy children be routinely screened for vitamin D levels?

## ChatGPT

Routine screening of healthy children for vitamin D levels is generally not recommended.

- Most healthy children with adequate sun exposure and a balanced diet maintain sufficient vitamin D.

- Universal testing can be costly and unnecessary if there are no risk factors or clinical signs.

- Screening is usually reserved for at-risk groups, such as:

- Exclusively breastfed infants without supplementation

- Children with limited sun exposure

- Those with chronic illnesses affecting absorption or metabolism

- Children on long-term medications that affect vitamin D

For healthy children, emphasis is placed on adequate dietary intake, safe sun exposure, and supplementation when indicated rather than routine laboratory testing.

## Gemini

No, medical authorities generally do not recommend routine screening for vitamin D levels in healthy, asymptomatic children.

The consensus from organizations such as the American Academy of Pediatrics (AAP) and the Endocrine Society is based on the following reasons:

1. Universal Supplementation Guidelines

Because vitamin D deficiency is common and the blood test is relatively expensive, experts suggest focusing on universal supplementation rather than universal testing. It is more cost-effective to ensure all children receive the recommended 400–600 IU daily than to test every child.

2. Lack of Evidence for Broad Screening

There is currently no strong clinical evidence showing that testing healthy children (who have no symptoms of rickets or bone pain) leads to better health outcomes than simply following standard supplementation and dietary guidelines.

3. Fluctuating Levels

Vitamin D levels vary significantly based on the season, recent sun exposure, and diet. A single "snapshot" blood test may not accurately reflect a child's overall status throughout the year.

When is Testing Appropriate?

Routine screening is reserved only for high-risk groups where standard doses might be insufficient. This includes children with:

Clinical symptoms (e.g., bowed legs, frequent fractures, or unexplained bone pain).

Chronic diseases affecting absorption (e.g., Celiac disease, Cystic Fibrosis, or Inflammatory Bowel Disease).

Organ dysfunction (e.g., chronic kidney or liver disease).

Obesity, as vitamin D is sequestered in fat tissue and may require higher doses.

Specific medications that accelerate vitamin D metabolism.

Summary

If a child is growing well, active, and taking a daily supplement, a blood test is usually unnecessary. The focus remains on prevention through consistent intake rather than monitoring through frequent blood draws.

## Qianwen

No, healthy children do not need to be routinely screened for vitamin D levels.

As mentioned in our previous discussions, for children who are healthy, have no symptoms of deficiency, and are already taking the recommended daily supplements (400–800 IU), routine blood testing is generally unnecessary and not supported by major medical guidelines .

Here is the medical reasoning behind this recommendation:

Lack of Evidence for Benefit

There is currently insufficient evidence to support the benefits of screening the general population of healthy children.

Major health organizations, including the US Preventive Services Task Force (USPSTF) and the Chinese consensus on vitamin D, do not recommend routine screening for asymptomatic children .

Risk of Misinterpretation and Over-treatment

Routine screening can actually do more harm than good due to inconsistencies in diagnostic criteria:

Confusing Results: Different laboratories use different standards to define "deficiency." A child might be labeled "deficient" by one lab but "normal" by another .

Unnecessary Medication: Parents may get anxious about a "low" result and push for high-dose vitamin D treatments. This increases the risk of vitamin D toxicity (hypercalcemia), which can damage kidneys and blood vessels .

Prevention is More Cost-Effective

For a healthy child, the most practical and cost-effective approach is prophylaxis (prevention) rather than testing.

Standard Protocol: If a child is taking 400–800 IU of vitamin D daily and has a balanced diet, they are virtually guaranteed to have sufficient levels without needing a blood test .

Focus on Symptoms: Medical resources should be reserved for children with clinical signs (like rickets symptoms discussed earlier) or high-risk factors (such as malabsorption disorders, obesity, or dark skin pigmentation) .

Summary

Unless your child has specific symptoms (like bone pain or fractures) or underlying health conditions, there is no need to worry about their exact vitamin D number.

Just stick to the daily supplement routine.

To ensure your child is on the right track without needing a test, could you confirm how much vitamin D your child is currently taking daily, and what type of supplement it is (e.g., vitamin D3 drops)?

## DeepSeek

No, healthy children without risk factors should not be routinely screened for vitamin D levels. This recommendation is supported by multiple major health organizations.

Here is a summary of the current recommendations:

| Organization / Guideline | Recommendation on Routine Screening (Asymptomatic, Average-Risk Children) |

| :--- | :--- |

| American Academy of Pediatrics (AAP) | Does not recommend universal screening for healthy adolescents, including those who are obese or dark-skinned. |

| Global Consensus on Nutritional Rickets (2016) | Recommends that routine 25(OH)D screening should not be performed in healthy children. |

| Endocrine Society (2024) | Suggests against routine 25(OH)D testing in the absence of established indications. |

| Choosing Wisely (ASCP) | States lab testing is appropriate only for higher-risk patients; over-the-counter supplements are sufficient for most otherwise healthy patients. |

| Health Technology Assessment (Canada, 2025) | 25-OH vitamin D testing is not indicated for children who are asymptomatic and without risk factors. |

Routine screening is generally considered unnecessary and may lead to unnecessary costs and interventions. A 2024 clinical audit in the UK found that over 56% of vitamin D tests in children were performed on asymptomatic individuals, a practice that goes against guidelines.

Reasons to Avoid Routine Screening

- Lack of Clinical Benefit for Healthy Kids: For an asymptomatic child who feels well and has no risk factors, a low test result often doesn't change the general recommendation, which is simply to follow standard supplementation and dietary guidelines.

- Cost & Overutilization: Widespread testing of low-risk populations drives up healthcare costs without a proven benefit to patient outcomes.

- Risk of Unnecessary Treatment: Screening can sometimes lead to treatment for lab values that are considered "low" by certain definitions but may not actually represent a true disease state that harms the child.

Exceptions: When Testing IS Recommended

Testing for vitamin D is appropriate for children who have specific risk factors or signs/symptoms. You should suspect that testing is necessary rather than just screening in cases such as:

- Signs/Symptoms: Bone pain, muscle weakness, recurrent fractures, or delayed growth.

- Medical Conditions: Rickets, chronic kidney or liver disease, malabsorption (Celiac, Crohn's), and cystic fibrosis.

- High-Risk Factors: Chronic use of anti-seizure medications or glucocorticoids, severe obesity, or conditions with limited mobility.

- Infant Risk: Prolonged breastfeeding without supplementation, especially if the infant is also dark-skinned.

In summary, for a healthy child who does not fall into those high-risk groups, the focus should be on getting enough vitamin D from foods and standard supplementation (e.g., the AAP suggests 400 IU per day for infants and children), rather than on blood testing.

## Doubao

Routine universal blood screening for vitamin D is not recommended for all healthy asymptomatic children. Healthy kids with regular sun exposure, balanced diet and standard vitamin D supplementation have very low risk of deficiency, so routine lab testing is unnecessary and wasteful. Instead of general screening, doctors adopt targeted testing: only test children in high-risk groups or those with suspicious symptoms/signs. For ordinary healthy children, standard daily vitamin D supplementation is preferred over blood testing.

# Question 25. What 25-hydroxyvitamin D level is considered deficient, insufficient, or normal?

## ChatGPT

The classification of serum 25-hydroxyvitamin D [25(OH)D] levels in children is generally as follows:

- Deficient: < 20 ng/mL (< 50 nmol/L) – Insufficient vitamin D for normal bone mineralization; risk of rickets or osteomalacia.

- Insufficient: 20–29 ng/mL (50–74 nmol/L) – Suboptimal levels that may not fully support bone health.

- Sufficient / Normal: 30–100 ng/mL (75–250 nmol/L) – Adequate for healthy bone growth and metabolism.

- Potentially toxic: > 100–150 ng/mL (> 250–375 nmol/L) – Rare, usually due to excessive supplementation, and may cause hypercalcemia.

These thresholds are widely used in pediatric practice to guide diagnosis and supplementation.

## Gemini

Yes, when a vitamin D level is found to be low, checking calcium, phosphorus, alkaline phosphatase (ALP), and parathyroid hormone (PTH) is essential. These markers allow doctors to determine if the deficiency is "silent" or if it has progressed to a clinical stage that is actively damaging the bones (Nutritional Rickets).

Here is why each marker is checked:

1. Calcium and Phosphorus (The Raw Materials)

These are the minerals that harden the bones.

Calcium: Even if vitamin D is low, blood calcium often remains normal because the body "steals" calcium from the bones to keep the blood levels steady. A low blood calcium level is a late-stage sign and can lead to muscle spasms or seizures (tetany).

Phosphorus: In vitamin D deficiency, the kidneys often flush out phosphorus. Low phosphorus is a hallmark sign that the body is struggling to mineralize new bone.

2. Parathyroid Hormone (PTH) (The "Alarm" System)

PTH is the most sensitive indicator of how much the deficiency is affecting the body.

The Reaction: When vitamin D is low and calcium absorption drops, the parathyroid glands work overtime, secreting high levels of PTH.

The Impact: High PTH levels tell the body to dissolve bone to release calcium into the blood. If PTH is elevated, it confirms that the vitamin D deficiency is biologically significant and requires immediate treatment.

3. Alkaline Phosphatase (ALP) (The Bone Activity Marker)

ALP is an enzyme found in bone-building cells (osteoblasts).

The Signal: When bones are soft and struggling to harden due to lack of vitamin D, bone turnover increases, and ALP "leaks" into the bloodstream in high amounts.

The Diagnosis: A significantly elevated ALP is often the first laboratory sign of rickets. Doctors also use it to monitor healing—as the child recovers, the ALP level will gradually drop back to normal.

Summary of Laboratory Patterns

Why these matter for treatment:

Checking these additional values helps the pediatrician decide:

The Dosage: Whether the child needs a standard supplement or a high-dose "loading" treatment.

Mineral Support: Whether the child also needs extra oral calcium and phosphorus supplements, rather than just vitamin D.

Risk of Complications: Whether the child is at risk for immediate issues like hypocalcemic seizures.

## Qianwen

The determination of vitamin D status is based on the serum concentration of 25-hydroxyvitamin D [25(OH)D].

While slight variations exist between different medical societies, the most widely accepted clinical consensus (such as the 2025 Clinical Consensus on Vitamin D and its Analogues) classifies the levels as follows :

Vitamin D Deficiency (缺乏)

Threshold: < 20 ng/mL (or < 50 nmol/L).

Clinical Significance: At this level, the body cannot absorb enough calcium. This is the range where patients are at high risk for rickets (in children) and osteomalacia (in adults).

Action: This typically requires medical intervention, often involving "therapeutic dosing" (high-dose supplementation) to rapidly replenish stores, followed by a maintenance dose.

Vitamin D Insufficiency (不足)

Threshold: 20 – 29 ng/mL (or 50 – 75 nmol/L).

Clinical Significance: This is a "grey area." While it may not cause immediate skeletal deformities, it is considered suboptimal for overall health and bone maintenance.

Action: Usually managed by increasing the daily maintenance dose (e.g., from 400 IU to 600–800 IU or higher depending on age) and ensuring adequate dietary calcium and sunlight exposure.

Vitamin D Sufficiency (充足/正常)

Threshold: ≥ 30 ng/mL (or ≥ 75 nmol/L).

Clinical Significance: This is the target range for optimal bone health and calcium absorption.

Action: Continue current supplementation and lifestyle habits.

Important Note on Units

Labs use different units of measurement. It is crucial to check the units on the report:

Conversion: 1 ng/mL = 2.5 nmol/L.

For example, if your report says 50 nmol/L, that is the borderline line between "Insufficient" and "Deficient."

Upper Limit Warning (Toxicity)

Threshold: > 150 ng/mL (or > 375 nmol/L).

Risk: Levels this high can lead to Vitamin D Toxicity, causing hypercalcemia (too much calcium in the blood), which can damage the kidneys and heart. This is rare and usually results from accidental over-supplementation .

Since different labs use different units, it can be easy to get confused. Does the report you are looking at use "ng/mL" or "nmol/L"?

If you have a specific number, feel free to share it, and I can help interpret it.

## DeepSeek

There is no single universal standard for defining vitamin D deficiency in children; different health organizations use slightly different thresholds. However, most guidelines agree on a general framework.

The table below summarizes the definitions from several major pediatric guidelines for easy comparison.

| Organization / Source | Year | Deficiency (Low) | Insufficiency (Inadequate) | Sufficiency (Normal) | Notes |

| :--- | :--- | :--- | :--- | :--- | :--- |

| Chinese Pediatric Guidelines | 2022 | < 30 nmol/L (< 12 ng/mL) | 30 - 50 nmol/L (12 - 20 ng/mL) | > 50 - 250 nmol/L (> 20 - 100 ng/mL) | The most current Chinese national guideline. |

| Royal Children's Hospital (Australia) | 2020 | < 30 nmol/L (< 12 ng/mL) | 30 - 49 nmol/L (12 - 19.6 ng/mL) | ≥ 50 nmol/L (≥ 20 ng/mL) | Uses a 3-tier system for severity: Severe <12.5; Moderate 12.5-29; Mild 30-49. |

| Royal Osteoporosis Society (UK) | 2018 | < 25 nmol/L (< 10 ng/mL) | 25 - 50 nmol/L (10 - 20 ng/mL) | > 50 nmol/L (> 20 ng/mL) | Explicitly states "no universal consensus." |

| Archives of Disease in Childhood (UK) | — | < 25 nmol/L (< 10 ng/mL) | 25 - 50 nmol/L (10 - 20 ng/mL) | Not specified in this excerpt. | |

| IOM & Pediatric Endocrine Society (US) | — | < 50 nmol/L (< 20 ng/mL) | — | ≥ 50 nmol/L (≥ 20 ng/mL) | Does not use an "insufficiency" category. |

Key Takeaways from the Guidelines

- The "50 nmol/L (20 ng/mL) Rule:" The most consistent finding across all guidelines is that a serum 25(OH)D level above 50 nmol/L (20 ng/mL) is considered "sufficient" or "normal" for the vast majority of children. This is the primary target for bone health.

- The Definition of "Deficiency" Varies: The main difference between guidelines is where to set the "deficiency" threshold.

- The Chinese Pediatric Guidelines and the Royal Children's Hospital use <30 nmol/L (<12 ng/mL) as the cutoff for deficiency.

- The UK's ROS and ADC use a lower threshold of <25 nmol/L (<10 ng/mL).

- In contrast, the US Institute of Medicine (IOM) and Pediatric Endocrine Society classify any level <50 nmol/L (<20 ng/mL) as deficient, with no "insufficiency" category. This is a more stringent definition.

- The Purpose of the "Insufficiency" Zone: Levels between 30-50 nmol/L (12-20 ng/mL) are widely labeled as "insufficient" or "inadequate". This is a "gray area" indicating low status. While these levels may not yet cause rickets, they prompt clinical concern, especially in children with other risk factors.

Clinical Context: It is important to remember that serum 25(OH)D is just one piece of the puzzle. A doctor diagnosing a condition like rickets will not rely solely on this number. They will also consider clinical signs (e.g., bone deformities, muscle weakness) and other lab results, such as elevated alkaline phosphatase (ALP) or low serum calcium and phosphorus.

## Doubao

Based on mainstream pediatric clinical criteria: - Deficient: 25(OH)D ＜ 20 ng/mL - Insufficient: 20–29 ng/mL - Sufficient / Normal: ≥ 30 ng/mL - Optimal level: 30–50 ng/mL - Excess: ＞ 100 ng/mL - Toxicity risk: ＞ 150 ng/mL

# Question 26. When vitamin D is low, should calcium, phosphorus, alkaline phosphatase, or parathyroid hormone also be checked?

## ChatGPT

Yes, when vitamin D is low, it is recommended to check calcium, phosphorus, alkaline phosphatase (ALP), and parathyroid hormone (PTH) to assess the impact on bone metabolism:

1. Calcium – Low vitamin D can reduce calcium absorption, leading to hypocalcemia.

2. Phosphorus – Often low in vitamin D deficiency due to secondary effects on renal handling and bone mineralization.

3. Alkaline phosphatase (ALP) – Typically elevated in children with rickets or active bone turnover, indicating impaired mineralization.

4. Parathyroid hormone (PTH) – Usually elevated (secondary hyperparathyroidism) in response to low calcium, reflecting compensatory mechanisms.

Together, these tests help determine the severity of deficiency, guide treatment, and monitor response to supplementation.

## Gemini

Yes, when a vitamin D level is found to be low, checking calcium, phosphorus, alkaline phosphatase (ALP), and parathyroid hormone (PTH) is essential. These markers allow doctors to determine if the deficiency is "silent" or if it has progressed to a clinical stage that is actively damaging the bones (Nutritional Rickets).

Here is why each marker is checked:

1. Calcium and Phosphorus (The Raw Materials)

These are the minerals that harden the bones.

Calcium: Even if vitamin D is low, blood calcium often remains normal because the body "steals" calcium from the bones to keep the blood levels steady. A low blood calcium level is a late-stage sign and can lead to muscle spasms or seizures (tetany).

Phosphorus: In vitamin D deficiency, the kidneys often flush out phosphorus. Low phosphorus is a hallmark sign that the body is struggling to mineralize new bone.

2. Parathyroid Hormone (PTH) (The "Alarm" System)

PTH is the most sensitive indicator of how much the deficiency is affecting the body.

The Reaction: When vitamin D is low and calcium absorption drops, the parathyroid glands work overtime, secreting high levels of PTH.

The Impact: High PTH levels tell the body to dissolve bone to release calcium into the blood. If PTH is elevated, it confirms that the vitamin D deficiency is biologically significant and requires immediate treatment.

3. Alkaline Phosphatase (ALP) (The Bone Activity Marker)

ALP is an enzyme found in bone-building cells (osteoblasts).

The Signal: When bones are soft and struggling to harden due to lack of vitamin D, bone turnover increases, and ALP "leaks" into the bloodstream in high amounts.

The Diagnosis: A significantly elevated ALP is often the first laboratory sign of rickets. Doctors also use it to monitor healing—as the child recovers, the ALP level will gradually drop back to normal.

Summary of Laboratory Patterns

Why these matter for treatment:

Checking these additional values helps the pediatrician decide:

The Dosage: Whether the child needs a standard supplement or a high-dose "loading" treatment.

Mineral Support: Whether the child also needs extra oral calcium and phosphorus supplements, rather than just vitamin D.

Risk of Complications: Whether the child is at risk for immediate issues like hypocalcemic seizures.

## Qianwen

Yes, when vitamin D is low, it is highly recommended to check calcium, phosphorus, alkaline phosphatase, and parathyroid hormone.

While the 25-hydroxyvitamin D level tells us the "storage" status of the vitamin, these other four markers reveal how the body is functionally reacting to that deficiency.

Together, they help distinguish between simple nutritional insufficiency and active metabolic bone disease (like rickets).

The Physiological "Chain Reaction"

When vitamin D is low, the body initiates a series of compensatory mechanisms to keep the blood calcium level stable. Checking these markers helps doctors evaluate the severity of the situation:

Parathyroid Hormone (PTH): The Early Warning Signal

Why check it: This is often the first marker to change. When vitamin D drops, the parathyroid glands become overactive to try to maintain blood calcium levels.

Expected Result: PTH levels typically rise (Secondary Hyperparathyroidism) even before calcium drops.

Serum Calcium: The Priority Target

Why check it: The body prioritizes keeping blood calcium within a narrow range for heart and nerve function.

Expected Result: Calcium levels may remain normal initially due to the high PTH pulling calcium from the bones. A drop in blood calcium is a late sign and can lead to symptoms like muscle cramps or seizures.

Serum Phosphorus: The Sensitive Marker

Why check it: High PTH causes the kidneys to dump phosphorus into the urine to save calcium.

Expected Result: Phosphorus levels are often lower in active vitamin D deficiency and rickets.

Alkaline Phosphatase (ALP): The Bone Turnover Indicator

Why check it: When bones are soft and struggling to mineralize, bone cells work overtime to repair them.

Expected Result: ALP levels typically rise significantly. It is a very sensitive marker for active rickets.

Clinical Value of the Combined Test

Checking these markers alongside vitamin D is crucial because it helps doctors:

Stage the Disease: Determine if the child is in the early "pre-rickets" stage or has full-blown rickets.

Rule Out Other Diseases: Distinguish between nutritional rickets (low calcium/phosphorus) and genetic issues like hypophosphatemic rickets (where calcium is normal but phosphorus is extremely low).

Guide Treatment: Decide whether simple vitamin D supplementation is enough or if calcium and phosphorus supplements are also needed.

To better understand your current situation, Do you have the results for these markers (Calcium, Phosphorus, ALP, or PTH) yet?

If so, please share them, and I can help explain what the pattern suggests.

## DeepSeek

Yes, when a child is found to have low vitamin D (25-hydroxyvitamin D), it is clinically important to also check serum calcium, phosphorus, alkaline phosphatase (ALP), and parathyroid hormone (PTH). These additional tests provide a complete picture of the child's mineral metabolism and help determine if the deficiency is causing physiological disturbances or rickets.

Why each test is important:

| Test | What it tells you in the context of low vitamin D |

| :--- | :--- |

| Calcium (total or ionized) | Vitamin D deficiency impairs intestinal calcium absorption. Low calcium (hypocalcemia) indicates significant functional deficiency and can be dangerous (seizures, tetany). Normal calcium does not rule out rickets. |

| Phosphorus | Vitamin D typically increases phosphorus absorption. In deficiency, especially with secondary hyperparathyroidism, phosphorus may be low (hypophosphatemia), which contributes to rickets and bone deformities. |

| Alkaline Phosphatase (ALP) | ALP is a marker of bone turnover. Elevated ALP (often markedly so) is a key sign of rickets, reflecting osteoblast activity trying to mineralize unmineralized osteoid. Normal or mildly elevated ALP suggests less severe deficiency. |

| Parathyroid Hormone (PTH) | Low vitamin D → low calcium → stimulates PTH (secondary hyperparathyroidism). Elevated PTH confirms that the deficiency is having a physiological impact (it increases bone resorption and renal phosphorus wasting, worsening rickets). |

What the combination of results indicates:

| Pattern | Interpretation |

| :--- | :--- |

| Low 25(OH)D + normal Ca, P, ALP, PTH | Simple, uncomplicated vitamin D deficiency. No significant secondary hyperparathyroidism or bone disease yet. Often asymptomatic. |

| Low 25(OH)D + low/normal Ca + low P + high ALP + high PTH | Classic biochemical profile of nutritional rickets (overt bone disease requiring treatment). |

| Low 25(OH)D + low/normal Ca + normal/normal P + normal ALP + normal PTH | Possible mild or early deficiency; may need repeat testing or clinical correlation. |

| Low 25(OH)D + low Ca + high P + high ALP + low/normal PTH | Suggests possible hypoparathyroidism or other disorders – not typical for simple deficiency. |

When can you skip these tests? In an otherwise healthy, asymptomatic child with mildly low 25(OH)D (e.g., 25–30 nmol/L or 10–12 ng/mL) and no clinical signs of rickets, some clinicians may forgo the full panel if the plan is simply to start standard-dose vitamin D supplementation and follow clinically. However, if any of the following are present, the full panel is strongly recommended:

- Clinical signs of rickets (bone deformities, widened wrists, rachitic rosary, etc.)

- Symptoms (bone pain, muscle weakness, hypocalcemic seizures)

- Very low 25(OH)D (< 25 nmol/L or < 10 ng/mL)

- Growth failure or recurrent fractures

- Underlying conditions (malabsorption, liver/kidney disease, medications)

Summary: For a child with confirmed low vitamin D, checking calcium, phosphorus, ALP, and PTH is good clinical practice – it helps distinguish simple deficiency from rickets, guides treatment intensity (higher doses or longer duration if rickets is present), and provides a baseline for monitoring response to therapy.

## Doubao

Yes, they should be checked as an essential complement to 25-hydroxyvitamin D testing. When vitamin D deficiency is suspected, testing serum calcium, phosphorus, alkaline phosphatase (ALP), and parathyroid hormone (PTH) helps judge the severity, distinguish nutritional rickets from other bone diseases, and evaluate secondary hyperparathyroidism. - Low vitamin D → reduced calcium absorption → blood calcium drops → PTH rises → bone calcium is released, ALP increases, blood phosphorus decreases. These combined indicators help confirm the diagnosis, assess bone metabolism status, and guide supplementation and treatment.

# Question 27. Why might an X-ray be needed when rickets is suspected?

## ChatGPT

An X-ray may be needed when rickets is suspected to:

1. Confirm diagnosis – X-rays can show characteristic bone changes of rickets, such as:

- Widened, cupped, or frayed metaphyses (ends of long bones)

- Bowing of legs or other skeletal deformities

- Rachitic changes in the wrists, knees, or chest

2. Assess severity – Helps determine the extent of bone involvement and monitor progression or improvement during treatment.

3. Differentiate from other conditions – Ensures that bone deformities are due to rickets and not other metabolic or genetic bone disorders.

X-rays are particularly useful when clinical signs are present but laboratory findings alone are inconclusive.

## Gemini

An X-ray is the definitive "gold standard" for diagnosing rickets because it allows doctors to see the actual structural damage to the growth plates that blood tests alone cannot show. While a blood test confirms a nutrient shortage, an X-ray confirms whether that shortage has caused physical bone disease.

Here is why an X-ray is necessary and what it reveals:

1. Visualizing the Growth Plate (The "Metaphysis")

In a healthy child, the ends of the long bones (metaphyses) appear sharp and well-defined. In a child with rickets, these areas show classic pathological changes:

Cupping: The end of the bone loses its flat or rounded shape and becomes concave, like the inside of a cup.

Fraying: Instead of a crisp line, the edge of the bone looks ragged, fuzzy, or "moth-eaten."

Splaying: The ends of the bones look abnormally wide because the unmineralized cartilage is being pushed outward by the body's weight.

2. Assessing Bone Density and Thinning

X-rays show the "whiteness" of the bone, which represents mineral content.

Radiolucency: Bones with rickets appear more transparent or grey on an X-ray because they lack the calcium "density" to block the X-ray beams.

Cortical Thinning: The hard outer shell of the bone (the cortex) may appear dangerously thin.

3. Detecting "Silent" Fractures

Children with rickets can develop Looser’s zones (also known as pseudofractures). These are thin, lucent lines across the bone where the bone has micro-fractured but hasn't fully broken. They are often painful but can only be seen via imaging.

4. Determining the Severity and Location

Rickets affects the entire skeleton, but X-rays are typically taken of the wrists and knees because these are the areas of most rapid growth where the signs appear first. This helps doctors grade the severity of the disease and decide how aggressive the treatment must be.

5. Monitoring the Healing Process

Once treatment starts, X-rays are used to track recovery. Within weeks of starting high-dose Vitamin D, a "line of healing" (a white line of new calcium deposition) will appear on the X-ray. This is the most reliable way to prove that the body is successfully utilizing the supplements.

Summary: Clinical Pattern on X-ray

## Qianwen

An X-ray is often the decisive test needed to confirm the diagnosis and determine the severity of the condition when rickets is suspected.

While blood tests (like Vitamin D, Calcium, and PTH) tell us about the body's metabolic status, an X-ray reveals the actual structural impact on the bones.

Confirming the Diagnosis and Staging the Disease

Blood tests can show a deficiency, but an X-ray confirms whether that deficiency has progressed to active rickets (rickets in the "active phase" or jī qī).

Diagnostic Certainty: In some cases, blood levels of calcium and phosphorus may be borderline or normal due to the body's compensatory mechanisms (high PTH), but the bone structure itself may already be damaged. The X-ray catches this structural damage .

Staging: It helps doctors distinguish between early-stage rickets (where X-rays might look normal) and active, severe rickets .

Visualizing Characteristic Bone Changes

The most distinct reason for an X-ray is to look for specific signs that act as "fingerprints" for active rickets, particularly at the ends of long bones (like the wrist or knee):

Cup-shaped Deformity: The end of the bone looks like a cup or a brush (frayed metaphysis) instead of a sharp, straight line .

Epiphyseal Widening: The growth plate appears abnormally wide because the bone is growing but not hardening (calcifying) properly .

Osteoporosis: The bones may look "washed out" or less dense than normal because they are soft and lacking minerals .

Assessing Damage and Guiding Treatment

Evaluating Deformities: If the child has visible bow legs (O-legs) or knock knees (X-legs), the X-ray measures the angle of the deformity. This helps doctors decide if simple Vitamin D treatment is enough or if orthopedic intervention is needed .

Monitoring Recovery: After treatment begins, X-rays serve as a benchmark. As the child heals, a new "temporary zone of calcification" (a white line) will reappear on the X-ray, proving the treatment is working .

Summary

In short, blood tests tell us why the child is sick (low nutrients), but the X-ray tells us how bad the disease has gotten (bone damage).

To give you more specific advice, has the doctor mentioned any specific symptoms like bowed legs, a "pigeon chest," or delayed walking?

## DeepSeek

An X-ray may be needed when rickets is suspected because it is the gold standard for confirming the diagnosis of rickets and assessing its severity. While clinical signs (e.g., bowed legs, rachitic rosary) and biochemical abnormalities (low vitamin D, elevated alkaline phosphatase) strongly suggest rickets, X-rays provide direct, objective evidence of bone changes at the growth plates, which are pathognomonic for rickets.

What X-rays show in rickets: The most sensitive sites for X-ray evaluation are the wrist (distal radius and ulna) and knee (distal femur, proximal tibia/fibula). Classic radiographic findings in active rickets include:

- Cupping: The metaphyseal surface appears concave or cup-shaped instead of flat or slightly convex.

- Fraying: The normally sharp, well-defined metaphyseal border becomes irregular, ragged, or fuzzy due to unmineralized osteoid.

- Widening: The growth plate (physeal width) is increased because unmineralized cartilage and osteoid accumulate.

- Decreased bone density: Generalized osteopenia (bones look less opaque) due to poor mineralization.

- Subperiosteal erosions: In severe, long-standing cases.

Why X-ray is superior to clinical exam alone:

- Early detection – Radiographic changes can appear before bone deformities are clinically visible.

- Confirmation – Many non-ricketic conditions (e.g., physiological bowing, Blount's disease, metaphyseal dysplasias) can mimic rickets clinically but have normal X-rays.

- Severity assessment – The degree of cupping, fraying, and widening correlates with disease severity and helps guide treatment.

- Baseline for follow-up – Healing rickets shows characteristic X-ray changes (e.g., a dense line of provisional calcification appearing after 2–4 weeks of treatment), which confirms that therapy is working.

When is an X-ray most indicated?

- Suspected rickets based on physical exam (leg deformities, rachitic rosary, widened wrists) → X-ray indicated.

- Biochemical changes suggestive of rickets (very low 25(OH)D, hypophosphatemia, markedly elevated ALP) → X-ray indicated – can confirm subclinical rickets.

- Child at high risk with subtle signs (e.g., delayed walking, mild muscle weakness) → X-ray may be considered.

- Infants with craniotabes or delayed fontanelle closure → X-ray of wrists may be helpful, but skull X-rays are not typically needed.

- Follow-up of known rickets to assess healing → Repeat X-ray after 3–6 months of treatment (not earlier than 4 weeks).

Are X-rays always necessary? No. In resource-limited settings or when rickets is clinically obvious and the presentation is classic, some clinicians may start treatment without X-rays, relying on clinical and biochemical response. However, X-rays provide definitive documentation and are strongly recommended by most pediatric endocrinology and bone disease guidelines.

Summary: An X-ray (usually of the wrist or knee) is the objective diagnostic test for rickets. It confirms the diagnosis, rules out mimics, assesses severity, and provides a baseline for monitoring treatment response. If rickets is suspected based on clinical or laboratory findings, an X-ray is generally indicated.

## Doubao

X-ray is essential for confirming rickets and judging severity, for these reasons: 1. It can show characteristic metaphyseal changes at long bone ends (widened, cupped, frayed epiphyseal plates) that are the typical radiological signs of rickets. 2. It evaluates bone mineral density and detects early skeletal damage that can’t be seen by naked eye. 3. It differentiates nutritional rickets from other causes of rickets (such as renal rickets, hereditary bone diseases). 4. It records baseline bone changes and is used to follow up treatment effect after vitamin D and calcium supplementation.

# Question 28. How should parents interpret a low vitamin D blood test result if the child has no symptoms?

## ChatGPT

If a child has a low vitamin D blood test but no symptoms, parents should understand that:

1. It indicates a biochemical deficiency, meaning vitamin D levels are below the optimal range, even if the child appears healthy.

2. Early detection is beneficial – Treating deficiency before symptoms develop can prevent bone problems, delayed growth, or rickets.

3. Treatment is usually simple – Pediatricians may recommend vitamin D supplementation at appropriate doses based on the child’s age and deficiency severity.

4. Follow-up is important – Rechecking blood levels after supplementation ensures the child reaches and maintains adequate vitamin D.

In short, asymptomatic deficiency is a preventive opportunity rather than an immediate cause for alarm.

## Gemini

If a child's blood test shows low vitamin D levels but they have no symptoms (no bowed legs, no bone pain, and normal growth), parents should view this as a "preventive warning" rather than an emergency. In medical terms, this is often a state of subclinical insufficiency.

Here is how to interpret and approach such a result:

1. Identify the Category

First, determine the severity of the "low" result based on the 25(OH)D level:

20–29 ng/mL (Insufficiency): This is very common. The child is not "sick," but their stores are lower than ideal. It is a sign to be more consistent with daily supplements and sun exposure.

< 20 ng/mL (Deficiency): This is more serious. Even without symptoms, the body may be starting to compensate by increasing parathyroid hormone (PTH). If left untreated, this could eventually lead to bone softening.

2. Why are there no symptoms?

The human body is remarkably good at maintaining balance. Even if vitamin D is low, the body will pull calcium from the "bank" (the bones) to keep blood calcium levels normal for the heart and muscles.

The "Silent" Phase: A child can have low vitamin D for months before the bones become soft enough to bend or hurt.

The Goal of the Test: Finding low levels before symptoms appear is actually a success—it allows for "catch-up" treatment that prevents rickets from ever developing.

3. Practical Steps for Parents

If the result is low, you should discuss the following with your pediatrician:

Review Supplementation: Is the child actually taking their 400–600 IU daily? Are they taking it with a meal? (Vitamin D is fat-soluble and is absorbed much better when eaten with some fat, like yogurt or avocado).

"Loading" Doses: For a result below 20 ng/mL, the doctor might prescribe a higher "therapeutic" dose (e.g., 2,000 IU or more per day) for 2 to 3 months to quickly refill the body's stores, before returning to a maintenance dose.

Environmental Audit: Consider if the child is using heavy sunscreen every time they are outside, or if air pollution or high latitudes are blocking their natural production.

4. When to Re-test

In an asymptomatic child, doctors usually recommend a follow-up blood test after 3 months of increased supplementation. It takes this long for the circulating levels in the blood to stabilize at a new "plateau."

Summary for Parents

A low result in a healthy-looking child is not a cause for panic, but it is a call to action. It indicates that the child's current lifestyle and diet are not providing enough "fuel" for their growing skeleton. Correcting it now ensures that their peak bone mass and height potential remain protected.

## Qianwen

A low vitamin D result in an asymptomatic child is a warning signal, not a diagnosis of disease. It indicates that the body's "storage tank" for vitamin D is running low, but it does not necessarily mean the child has rickets or immediate health risks.

Since the child shows no symptoms (like bone pain or deformities), this is likely an early detection of insufficiency rather than severe clinical deficiency.

The Concept of a "Silent" Buffer Zone

It is common for vitamin D levels to drop before symptoms appear. This is due to the body's compensatory mechanisms:

Prioritizing Blood Calcium: As mentioned earlier, the body will pull calcium from the bones to keep blood calcium levels stable for heart and nerve function.

The Hidden Cost: While the child feels fine and blood calcium remains normal, the "savings account" (vitamin D stores) is being depleted to pay for immediate expenses. The low blood test result is essentially a "low balance alert" from the body.

Actionable Steps for Parents

Even without symptoms, a low result requires action to prevent the condition from worsening into the symptomatic stage.

Adjust Supplementation:

If currently taking 400 IU: The doctor may recommend increasing the dose (e.g., to 800–1000 IU daily) for a specific period (usually 4–8 weeks) to replenish stores.

Therapeutic Dosing: In cases of significant deficiency (e.g., < 10 ng/mL), high-dose therapy might be prescribed temporarily.

Optimize Lifestyle: Encourage outdoor play (15–30 minutes of direct sunlight on arms and legs) and include vitamin D-rich foods like egg yolks, fatty fish, or fortified milk in the diet.

Re-evaluation: A follow-up test is typically recommended after 1–2 months of increased supplementation to ensure levels are rising towards the target range (≥ 30 ng/mL).

Summary

The absence of symptoms is a positive sign, meaning the bones have likely not suffered structural damage yet. The goal now is to correct the deficiency before it progresses to the point where symptoms develop.

To help tailor the advice, could you share the specific vitamin D level from the test report (e.g., 15 ng/mL or 40 nmol/L)?

This will help determine whether a slight increase in dosage is sufficient or if a stronger intervention is needed.

## DeepSeek

If a child has a low vitamin D blood test result (low 25-hydroxyvitamin D) but has no symptoms, parents should interpret this as an early biochemical finding, not a disease diagnosis. It indicates that the child’s vitamin D level is below the optimal range for bone health, but the deficiency has not yet progressed to the point of causing clinical signs (such as rickets, bone pain, or muscle weakness).

Key points for parents to understand:

- It is common – Asymptomatic vitamin D deficiency is frequently detected in children, especially those with risk factors (limited sun exposure, dark skin, exclusive breastfeeding without supplementation, etc.).

- It is not an emergency – In the absence of symptoms like seizures, bone deformities, or fractures, there is no need for urgent intervention. However, it should be addressed to prevent future problems.

- It is treatable – Most asymptomatic children respond well to standard vitamin D supplementation (e.g., 400–2000 IU/day depending on age and severity) without needing high-dose bolus therapy or hospitalization.

- It may or may not require further testing – Many pediatricians will check baseline calcium, phosphorus, alkaline phosphatase, and parathyroid hormone to rule out subclinical rickets. If all those are normal, the child likely has simple deficiency, not rickets.

What parents should do:

1. Discuss with the pediatrician – Ask whether additional tests (calcium, phosphorus, ALP, PTH) are needed. If the vitamin D level is only mildly low (>25–30 nmol/L or 10–12 ng/mL) and the child is healthy, further testing may not be required.

2. Clarify the target level – Most guidelines aim for 25(OH)D ≥ 50 nmol/L (≥ 20 ng/mL) for bone health.

3. Start supplementation as advised – The pediatrician will recommend an appropriate daily dose and duration. Typically, this is higher than routine prevention doses (e.g., 1000–2000 IU/day for several weeks to months), followed by a maintenance dose (400–600 IU/day).

4. Increase dietary and sun exposure – Include vitamin D-rich foods (fatty fish, fortified milk/products, egg yolks) and, if feasible and safe, moderate sun exposure (e.g., 10–15 minutes a few times per week on uncovered arms/legs, avoiding sunburn).

5. Schedule follow-up testing – After 3–6 months of treatment, a repeat 25(OH)D level can confirm that the level has normalized. No further monitoring is needed if the child remains asymptomatic and risk factors are managed.

What not to do:

- Do not panic – Asymptomatic low vitamin D is extremely unlikely to cause immediate harm.

- Do not self-prescribe high doses – Megadoses (e.g., 50,000 IU or more) are rarely needed in asymptomatic children and can cause toxicity if given repeatedly.

- Do not ignore it – Over months to years, untreated deficiency can progress to symptomatic rickets or contribute to low bone density.

Summary for parents:

| Finding | Meaning | Action |

| :--- | :--- | :--- |

| Low vitamin D, no symptoms, normal calcium/ALP/PTH | Simple, early deficiency – not rickets | Standard supplementation + lifestyle changes |

| Low vitamin D, no symptoms but mildly elevated ALP or low phosphorus | Possible subclinical rickets | More aggressive treatment, possibly X-ray |

| Low vitamin D, no symptoms, but very low level (<12 nmol/L or <5 ng/mL) | Severe biochemical deficiency | Full workup (calcium, phosphorus, ALP, PTH, ± X-ray) |

In short, an asymptomatic low vitamin D result is a call to action for prevention, not a cause for alarm. With proper supplementation and follow-up, most children achieve normal levels and never develop symptoms or complications. Parents should work with their pediatrician to correct the level and ensure ongoing adequate intake.

## Doubao

Asymptomatic low vitamin D is common in children, and parents can interpret it this way: 1. No symptoms ≠ no harm Even without night sweats, irritability or bone deformities, low vitamin D still weakens calcium absorption, lowers bone reserve, affects growth, immunity and muscle function, and raises future fracture risk. 2. Classify by lab level - Deficient (<20 ng/mL): Needs therapeutic supplementation, not just routine daily dose. - Insufficient (20–29 ng/mL): Adjust daily vitamin D intake and increase proper sun exposure, recheck after 3–6 months. 3. No need to panic Asymptomatic mild insufficiency is reversible with standardized supplementation and lifestyle adjustment; it won’t cause permanent skeletal damage if corrected early. 4. No need for extra repeated tests immediately, just standardize supplementation and follow up regularly No need to blindly check calcium, bone X-ray or other items if the child is totally asymptomatic and otherwise healthy.

# Question 29. After treatment for vitamin D deficiency, when should vitamin D or related laboratory markers be rechecked?

## ChatGPT

After treatment for vitamin D deficiency, vitamin D and related laboratory markers should typically be rechecked as follows:

1. Timing – Usually 8–12 weeks after starting supplementation, once steady-state levels are expected.

2. Tests to monitor –

- 25-hydroxyvitamin D [25(OH)D] – to confirm correction of deficiency

- Calcium and phosphorus – to ensure normalization of mineral balance

- Alkaline phosphatase (ALP) – to assess improvement in bone turnover

- Parathyroid hormone (PTH) – if elevated before treatment, to ensure secondary hyperparathyroidism resolves

3. Further follow-up – After achieving normal levels, testing may be repeated periodically if the child remains at risk (e.g., chronic illness, limited sun exposure, ongoing supplementation).

This schedule ensures that treatment is effective and prevents recurrence or over-supplementation.

## Gemini

After a child begins treatment for vitamin D deficiency, the timing for follow-up testing is crucial. Rechecking too early may not show the full effect of the treatment, while waiting too long could risk missing a lack of response.

The standard timeframe for re-evaluating laboratory markers is generally 8 to 12 weeks (2–3 months) after starting a therapeutic dose.

1. Why wait 8 to 12 weeks?

Steady State: Vitamin D has a long "half-life" (the time it stays in the blood). It takes approximately 3 months of consistent supplementation for the blood levels of 25-hydroxyvitamin D to reach a stable "plateau" or steady state.

Bone Healing: If the child has rickets, it takes several weeks for the minerals to begin depositing into the soft bone tissue. Checking earlier may not accurately reflect the healing process.

2. What markers are rechecked?

The follow-up involves more than just vitamin D; it checks how the whole system is responding:

25(OH)D Level: To confirm that the level has moved into the Sufficiency range (> 30 ng/mL).

Serum Calcium and Phosphorus: To ensure these minerals have normalized and that there is no "hypercalcemia" (too much calcium) from the treatment.

Alkaline Phosphatase (ALP): In children with rickets, a decrease in ALP is a key sign that the bones are successfully hardening.

Parathyroid Hormone (PTH): A successful treatment should cause an elevated PTH to drop back into the normal range.

3. Special Considerations

4. Adjusting the Dose

Once the follow-up tests confirm that the vitamin D levels have normalized (usually > 30 ng/mL) and that the ALP is decreasing, the doctor will typically transition the child from a high "treatment dose" (e.g., 2,000–5,000 IU) back down to a "maintenance dose" (usually 400–600 IU or 1,000 IU depending on age and risk factors).

Summary

For most children, the 3-month mark is the ideal time for a follow-up. If the levels have not improved by then, the doctor may investigate "non-adherence" (forgetting doses), improper absorption (not taking it with fat), or underlying intestinal issues.

## Qianwen

After treatment for vitamin D deficiency, the timing for rechecking vitamin D levels or related markers depends on the severity of the deficiency and the treatment regimen prescribed.

Generally, a follow-up is recommended 1 to 3 months after starting therapy to assess response, or 3 to 6 months for maintenance adjustments.

Recheck Timing Based on Treatment Intensity

Standard Maintenance or Mild Insufficiency (3–6 Months)

If the child has mild insufficiency (e.g., 20–29 ng/mL) and was prescribed a standard daily maintenance dose (e.g., 400–1000 IU daily), the "storage" in the body fills up slowly.

Action: Recheck 3 to 6 months later. Checking earlier than 3 months is often unnecessary because serum levels may not have stabilized yet, potentially leading to misinterpretation of the treatment's effectiveness.

High-Dose Therapeutic Treatment (1–3 Months)

If the deficiency was significant (e.g., < 20 ng/mL) and required a "therapeutic dose" (e.g., 2000–4000 IU daily or high-dose pulses), the levels rise much faster.

Action: Recheck 1 to 3 months after starting treatment. This allows the doctor to confirm the levels have reached the target range (≥ 30 ng/mL) so the dose can be safely lowered to a maintenance level, avoiding potential toxicity from over-supplementation.

Recheck Timing for Confirmed Rickets

If the diagnosis was active rickets (confirmed by X-ray or abnormal biochemistry like high Alkaline Phosphatase), the monitoring schedule is more intensive to ensure bone healing and safety.

Biochemical Markers (Calcium, Phosphorus, ALP, PTH):

Timing: Recheck 1 month after starting treatment.

Purpose:

Efficacy: In rickets, Alkaline Phosphatase (ALP) typically starts to decrease within a month of effective treatment, signaling bone healing.

Safety: To monitor for hypercalcemia (high blood calcium), which can occur as a side effect of aggressive vitamin D and calcium therapy.

Summary of Recommendations

For General Deficiency: Wait 3 months to recheck 25-hydroxyvitamin D.

For Active Rickets: Recheck Calcium, Phosphorus, and ALP at 1 month, followed by Vitamin D levels later.

Avoid Premature Testing: Rechecking Vitamin D too frequently (e.g., every few weeks) provides little clinical value due to the slow metabolic turnover of the vitamin.

## DeepSeek

After treatment for vitamin D deficiency, the timing for rechecking laboratory markers depends on the child's condition (simple deficiency vs. rickets) and the treatment approach. Guidelines consistently recommend monitoring to confirm treatment response and guide transition to maintenance therapy.

The table below summarizes rechecking schedules from major pediatric guidelines:

| Source | Rechecking Schedule | Key Conditions |

| :--- | :--- | :--- |

| Chinese Pediatric Guidelines (2022) | Every 3-4 months until target level achieved; then every 6 months | General vitamin D treatment monitoring |

| NHS Scotland (2025) | At 12 weeks (3 months) | Nutritional rickets treatment |

| NIH Review (2020) | Every 1-3 months (age <5 years); annually for 25(OH)D | Routine monitoring in high-risk groups |

| Endocrine Society (via MTMT) | After completing therapeutic course | Adolescents with chronic illnesses |

Specific Rechecking Recommendations by Scenario

1. For Simple Vitamin D Deficiency (Asymptomatic or Mild)

- Recheck at 3-4 months after starting supplementation.

- If levels normalize, transition to maintenance dosing (400-800 IU daily).

- Further monitoring may not be necessary if risk factors are addressed and the child remains asymptomatic.

2. For Nutritional Rickets (Symptomatic or Biochemical Abnormalities)

- Recheck at 12 weeks (3 months) with complete metabolic panel including: serum calcium, phosphorus, alkaline phosphatase (ALP), parathyroid hormone (PTH), and 25(OH)D.

- If biochemistry has improved, transition to maintenance vitamin D.

- If abnormalities persist (especially elevated ALP or PTH), assess compliance and consider further treatment.

3. For Children with Underlying Chronic Conditions

- Every 1-3 months for children under 5 years of age.

- Every 3-6 months for children 5-18 years.

- Annual 25(OH)D measurement regardless of age.

- After supplementation, recheck to confirm response, as some children may require repeat supplementation courses.

4. For High-Dose or Intermittent Therapy

- When using single high-dose ("Stoss") therapy (e.g., 150,000-300,000 IU): recheck after 3 months to assess effectiveness before repeating the dose.

Which markers should be rechecked?

- 25(OH)D – Direct measure of vitamin D status; target ≥50 nmol/L (≥20 ng/mL).

- Alkaline phosphatase (ALP) – Key indicator of bone healing; elevated levels should normalize with treatment.

- Parathyroid hormone (PTH) – Indicates secondary hyperparathyroidism; should decrease with treatment.

- Calcium & phosphorus – Monitor for hypocalcemia or hypercalcemia during treatment.

When to stop monitoring: Once the child achieves and maintains adequate 25(OH)D levels (typically >50 nmol/L or >20 ng/mL) with no ongoing risk factors or symptoms. The Chinese guideline suggests that further monitoring is not necessary after target levels are reached. For children with chronic conditions, some ongoing surveillance (e.g., annually) may be appropriate.

Important Clinical Notes:

- Don't recheck too early – Allow at least 3 months of treatment before re-testing, as it takes time for 25(OH)D stores to replete and for biochemical markers (especially ALP) to respond.

- Assess compliance first – If rechecking shows persistent abnormalities, evaluate adherence to supplementation before escalating therapy.

- Continue maintenance after treatment – After correction of deficiency, children should continue on daily maintenance vitamin D (400-800 IU) while risk factors persist.

## Doubao

For most children with vitamin D deficiency: 1. Simple deficiency without rickets: Recheck serum 25-hydroxyvitamin D 3 months after starting treatment. 2. With suspected or confirmed rickets (skeletal changes / abnormal blood biochemistry): Recheck 25(OH)D, calcium, phosphorus, alkaline phosphatase, PTH at 1 month first, then follow up again at 3 months. 3. Asymptomatic insufficiency (20–29 ng/mL): Adjust daily intake and recheck after 3–6 months. 4. Do not recheck too early (within 4–6 weeks): levels have not stabilized yet, leading to meaningless results. After levels return to normal, switch to regular daily maintenance dose, no need for frequent repeated tests.

# Question 30. When should newborns start vitamin D supplementation?

## ChatGPT

Newborns should start vitamin D supplementation shortly after birth, typically within the first few days to weeks, depending on feeding type:

1. Exclusively breastfed infants – Start supplementation at 400 IU (10 µg) per day soon after birth, since breast milk alone usually does not provide enough vitamin D.

2. Formula-fed infants – Usually receive adequate vitamin D if consuming at least 1 liter of fortified formula per day; supplementation may be needed if intake is lower.

3. Preterm or low-birth-weight infants – May require earlier or higher supplementation due to lower vitamin D stores and rapid growth needs.

Early supplementation helps prevent vitamin D deficiency and associated conditions such as rickets.

## Gemini

According to the American Academy of Pediatrics (AAP) and most international health organizations, newborns should start vitamin D supplementation within the first few days of life.

The standard recommendation is to begin supplementation no later than the end of the first week. Here is the detailed breakdown of why and how:

1. Why start so early?

Low Maternal Transfer: Most babies are born with relatively low vitamin D stores because the transfer from mother to baby during pregnancy is often insufficient to last long.

Breast Milk Limitations: Breast milk is the perfect nutrition for infants but is naturally very low in vitamin D (usually providing less than 80 IU per liter).

Sun Exposure Risks: Doctors recommend keeping infants under 6 months out of direct sunlight to prevent skin damage and cancer risk, meaning they cannot produce their own vitamin D.

2. The Standard Dosage

400 IU (10 micrograms) per day.

This dose should be maintained consistently throughout the first year of life.

3. Guidelines by Feeding Method

4. Special Considerations for Preterm Babies

Babies born prematurely often require higher doses (sometimes 400–800 IU per day) because they missed the final trimester of mineral transfer and have higher growth demands. Their specific dose is usually determined by the neonatal medical team upon discharge from the hospital.

5. Practical Advice for Parents

Consistency is Key: Use a dropper to place the liquid vitamin D directly into the baby's mouth or on the mother's nipple before breastfeeding.

Don't Wait for Solids: Vitamin D is needed long before a baby starts eating solid foods (which usually happens at 6 months).

Safety: Use only the dropper provided with the specific product to avoid over-concentration or under-dosing.

Summary

The "start date" for vitamin D is essentially immediately. Waiting until a few months of age puts the infant at risk for early-onset deficiency and, in severe cases, hypocalcemic seizures or rickets.

## Qianwen

Newborns should start vitamin D supplementation within the first few days to 2 weeks after birth.

While some older advice suggested waiting until 2 weeks, current pediatric guidelines (including the American Academy of Pediatrics and Chinese medical guidelines) recommend starting as early as possible to ensure immediate protection for bone development .

Why Start So Early?

Even though the baby is healthy now, early supplementation is crucial for several reasons:

Human Milk is Low in Vitamin D: Breast milk is the best food for a baby, but it naturally contains very little vitamin D. Relying on breastfeeding alone without supplements will almost certainly lead to a deficiency .

Maternal Stores are Often Insufficient: A baby is born with a "starter pack" of vitamin D from the mother, but this lasts only a few weeks. Since most mothers also have low vitamin D levels, this reserve is often not enough to cover the baby's needs for the first 2 weeks .

Sun Exposure is Risky: You cannot safely rely on sunlight for a newborn. Babies under 6 months should avoid direct sun to protect their sensitive skin and eyes, meaning they can't make their own vitamin D .

Recommended Dosage

For a healthy, full-term baby, the standard recommendation is:

400 International Units (IU) per day.

This applies whether the baby is breastfed or formula-fed (though formula-fed babies who drink more than 1 liter/32 oz of formula daily generally don't need extra supplements) .

Special Cases: Premature or Low Birth Weight Babies

If the newborn is premature (born early), a low birth weight baby, or a twin, their needs are higher because they missed out on the vitamin D transfer that usually happens in the mother's womb during the third trimester.

Dosage: Usually 800–1000 IU per day for the first 3 months, then reduced to 400 IU/day .

Note: Always follow the specific dosage prescribed by a neonatologist for these cases.

Practical Tips for Parents

When to start: If the baby is feeding well and is stable, you can start giving the drops the day after coming home from the hospital or within the first week.

How to give it: It is usually a small oil-based drop. You can put it directly in the baby's mouth, on a clean finger for the baby to suck, or on the nipple before nursing.

Consistency: Try to give it at the same time every day (e.g., with the morning feed) to build a habit.

## DeepSeek

Newborns should start vitamin D supplementation soon after birth, typically within the first few days of life.

Summary of key guidelines:

| Organization / Source | Recommendation | Dosage |

| :--- | :--- | :--- |

| Chinese Pediatric Guidelines (2024) | Start within 1 week of birth | 400–800 IU/day |

| American Academy of Pediatrics (AAP) | Start within the first few days of life | 400 IU/day |

| World Health Organization (WHO) | Start from birth (for very low birth weight infants) | 400–1000 IU/day (until 6 months) |

| Israel Ministry of Health | Start immediately after birth | 400 IU/day |

| Flanders Guidelines (PubMed) | Start from birth | 400 IU/day (600 IU/day for dark skin) |

Special Considerations for Different Newborns

Breastfed Newborns: Breast milk is low in vitamin D (typically <25 IU/L), so exclusively or partially breastfed infants need supplementation from birth until they are weaned or consume enough fortified formula. The standard dose is 400 IU/day.

Formula-Fed Newborns:

- If the infant drinks less than 1 liter (about 32 ounces) of vitamin D-fortified formula per day, they need 400 IU/day supplementation starting soon after birth.

- If the infant drinks 1 liter or more of fortified formula daily, additional supplementation is generally not needed, as formula provides sufficient vitamin D.

Preterm or Low-Birth-Weight Newborns: The WHO recommends vitamin D supplementation for very low birth weight (VLBW) infants at a dose of 400–1000 IU per day, starting from birth until 6 months of age. The Chinese guideline suggests 800 IU/day for the first 3 months, then reducing to 400 IU/day.

Why Start So Early? Newborns begin life with limited vitamin D stores, as placental transfer occurs mainly in the third trimester. Even with adequate maternal vitamin D levels, breastfed infants have only about 8–12 weeks of reserves. Additionally, direct sun exposure is not recommended for young infants (under 6 months) due to skin sensitivity and cancer risk. Therefore, supplementation is the safest and most reliable way to ensure adequate vitamin D intake.

Dosage Summary for Healthy Newborns:

- Standard daily dose: 400 IU

- Chinese guidelines: 400–800 IU daily

- Duration: Continue daily until the child can obtain adequate vitamin D from diet (typically through fortified formula/milk or foods), and consider continuing through childhood and adolescence as per local guidelines.

## Doubao

Newborns should start vitamin D supplementation as early as birth or within the first week after birth, no later than 2 weeks of age. - Both breastfed and formula-fed newborns need supplementation; breast milk has very low vitamin D content. - Standard daily preventive dose: 400 IU per day from birth through infancy and early childhood. - Preterm/low birth weight newborns usually need a higher initial dose, adjusted under pediatrician guidance.31.

# Question 31. How much vitamin D does an infant aged 0-12 months need each day?

## ChatGPT

Infants aged 0–12 months generally need 400 IU (10 µg) of vitamin D per day:

- This applies to exclusively breastfed infants and those receiving less than 1 liter of vitamin D–fortified formula per day.

- The dose can be provided as daily drops or fortified formula.

- Preterm or low-birth-weight infants may require individualized dosing based on pediatrician guidance.

This amount supports normal bone mineralization, growth, and prevention of rickets.

## Gemini

For an infant between 0 and 12 months of age, the universally recommended daily intake of vitamin D is 400 IU (10 micrograms).

This recommendation is consistent across major health organizations, including the American Academy of Pediatrics (AAP) and the World Health Organization (WHO).

1. Why 400 IU?

This specific amount is calibrated to maintain healthy blood levels of 25-hydroxyvitamin D (above 20 ng/mL) in almost all healthy infants. It is sufficient to:

Prevent the development of nutritional rickets.

Support the rapid bone mineralization occurring in the first year of life.

Optimize the immune system during a critical window of development.

2. Intake Requirements by Feeding Method

The source of the 400 IU depends on how the infant is being fed:

Exclusively or Partially Breastfed Infants: They should receive a 400 IU liquid supplement every day. Breast milk typically provides less than 50-80 IU per liter, which is far below the requirement.

Exclusively Formula-Fed Infants: Most modern infant formulas are fortified with vitamin D (at 400 IU per Liter).

If a baby drinks at least 1 liter (approx. 33 ounces) of formula daily, they do not need an extra supplement.

Since most newborns and young infants drink much less than a liter, they usually still need a supplement until their intake increases.

3. Special Cases (Higher Needs)

Some infants may require more than the standard 400 IU, but this should only be done under medical supervision:

Preterm Infants: Due to lower mineral stores at birth and higher growth velocity, they are often prescribed 400 to 800 IU daily.

Infants on Certain Medications: Babies taking medications for seizures or other chronic conditions may require higher doses to offset increased vitamin D metabolism.

High-Risk Groups: In some regions with extremely low sunlight or for infants with very dark skin, a pediatrician might suggest a slightly higher maintenance dose.

4. Important Safety Note: Avoiding Toxicity

While 400 IU is the "sweet spot" for health, more is not always better.

The Upper Limit: For infants under 6 months, the "tolerable upper intake level" is generally 1,000 IU; for those 6–12 months, it is 1,500 IU.

Risk: Consistently exceeding these amounts without medical guidance can lead to vitamin D toxicity, which causes dangerously high calcium levels (hypercalcemia) and can damage the kidneys and heart.

Summary

The magic number for the first year of life is 400 IU daily. For breastfed babies, this almost always requires a daily liquid drop starting in the first week of life.

## Qianwen

For a healthy, full-term infant, the standard recommendation is 400 International Units (IU) per day throughout the first year of life.

While the exact dietary requirement technically increases slightly after 6 months (to 600 IU), medical guidelines universally recommend a consistent 400 IU daily supplement for all infants aged 0–12 months to ensure safety and simplicity for parents.

Breakdown by Age and Feeding Method

0–6 Months

Dosage: 400 IU/day.

Reasoning: Breast milk contains very little Vitamin D, and formula is not always consumed in sufficient quantities (less than 1 liter/32 oz) to meet needs. Sun exposure is unsafe for this age group. Therefore, a supplement is essential.

7–12 Months

Dietary Goal: The Institute of Medicine suggests the dietary need rises to 600 IU/day.

Supplement Recommendation: Most pediatric guidelines (including the AAP and Chinese Pediatric Society) still recommend 400 IU/day from a supplement.

Why?

Safety Buffer: 400 IU from a supplement + Vitamin D naturally present in solid foods (like egg yolks) + Vitamin D in formula/milk usually meets the 600 IU goal without risking overdose.

Simplicity: It is easier for parents to maintain one consistent dosage (400 IU) from birth until the child is 1 year old rather than switching doses at 6 months.

Special Consideration: Premature Infants

If the baby was born prematurely or is a low birth weight twin, their bones need to mineralize rapidly.

First 3 Months: 800–1000 IU/day.

After 3 Months: Reduce to 400 IU/day.

Formula-Fed Exception

If the baby is exclusively formula-fed, you can do a quick calculation:

Formula generally contains about 400 IU per liter (approx. 100 IU per 8 oz).

If the baby drinks more than 1 liter (32 oz) of formula every day, they are getting enough Vitamin D from the bottle and do not need extra supplements.

If they drink less than 1 liter, the difference should be made up with drops (e.g., if they drink 500ml, they need ~200 IU extra).

## DeepSeek

For healthy infants aged 0-12 months, the recommended daily intake of vitamin D is 400 international units (IU), which is equivalent to 10 micrograms (mcg).

Summary of recommendations:

| Organization | Daily Dosage Recommendation for 0-12 Months |

| :--- | :--- |

| CDC (Centers for Disease Control and Prevention) | 400 IU |

| AAP (American Academy of Pediatrics) | 400 IU |

| Hong Kong FHS (Family Health Service) | 400 IU |

| Chinese Pediatric Guidelines | 400-800 IU |

Special Considerations for Different Infants

- Breastfed Infants: Human milk contains very little vitamin D. If a baby is fed only breastmilk or a mix of breastmilk and formula, a supplement of 400 IU per day is necessary, starting shortly after birth.

- Formula-Fed Infants: Infant formula is fortified with vitamin D. If a baby drinks at least 32 ounces (about 1 liter) of fortified formula per day, they likely meet the 400 IU requirement and do not need an additional supplement. If they drink less than this amount, a supplement is recommended.

- Preterm or Low-Birth-Weight Infants: These infants have higher needs. The recommended intake is often higher, typically 800-1000 IU per day for the first 3 months, after which it is usually reduced to 400 IU per day. Always follow the guidance of a neonatologist or pediatrician for these special cases.

Important Safety Note: There is an upper limit to how much vitamin D is safe. For infants, parents should not exceed the daily limits listed below unless specifically directed by a doctor.

| Age Group | Safe Upper Daily Limit (Tolerable Upper Intake Level) |

| :--- | :--- |

| 0-6 months | 1000 IU (25 mcg) |

| 6-12 months | 1500 IU (38 mcg) |

## Doubao

For healthy full-term infants aged 0–12 months: The daily recommended vitamin D intake is 400 IU per day. For preterm or low-birth-weight infants: 800 IU per day for the first 3 months after birth, then reduce to 400 IU per day for maintenance. Note: If an infant drinks more than 1000 mL of formula milk daily, calculate the vitamin D contained in the formula and only supplement the missing amount to reach 400 IU per day.

# Question 32. How much vitamin D does a child older than 1 year need each day?

## ChatGPT

Children older than 1 year generally need 600 IU (15 µg) of vitamin D per day:

- This includes toddlers and school-aged children.

- Can be obtained through dietary sources (fortified milk, fish, eggs), supplements, and safe sun exposure.

- Children at higher risk of deficiency (limited sun, darker skin, chronic illness) may require supplementation even if diet is adequate.

This intake helps maintain healthy bones, proper growth, and normal calcium/phosphorus metabolism.

## Gemini

For children older than 1 year and adolescents up to age 18, the recommended daily intake of vitamin D increases to 600 IU (15 micrograms).

This guideline is established by the Institute of Medicine (IOM) and the American Academy of Pediatrics (AAP) to support the continued rapid growth of long bones and the accrual of peak bone mass during childhood and puberty.

1. Why does the requirement increase?

As children transition from infancy to toddlerhood and beyond:

Greater Bone Mass: Their skeletal frame becomes larger and requires more calcium and phosphorus to maintain density.

Pubertal Growth Spurt: Adolescence is a critical window where nearly half of the adult skeleton is formed. 600 IU ensures there is enough vitamin D to facilitate the massive calcium demand during this time.

2. Sources of Vitamin D for Older Children

Unlike infants, older children can obtain vitamin D from a variety of sources, though it remains difficult to meet the 600 IU goal through diet alone.

Fortified Foods: * Milk: Most milk (dairy and many plant-based alternatives) is fortified with roughly 100–150 IU per cup.

Cereals: Many breakfast cereals provide about 40–60 IU per serving.

Orange Juice: Some brands are fortified with vitamin D and calcium.

Natural Food Sources: * Fatty fish (salmon, mackerel) and egg yolks contain small amounts.

Sunlight: Older children often spend more time outdoors, which aids production, but factors like sunscreen use, clothing, and geography (latitude) still limit this source.

3. Recommended Daily Allowances (RDA) Summary

4. When is a supplement still necessary?

Even with a healthy diet, many children fail to reach 600 IU. A supplement is often recommended if:

The child does not drink at least 32 ounces (4 cups) of fortified milk daily.

The child has darker skin pigmentation.

The family lives in northern latitudes where winter sun is ineffective.

The child spends most of their time indoors or always wears sunscreen.

Summary

While the "official" number is 600 IU, many pediatricians suggest that children in high-risk categories or those with limited dairy intake continue taking a daily supplement of 600–1,000 IU to ensure they reach their full genetic height and bone strength potential.

## Qianwen

For a child older than 1 year, the standard recommendation is typically 600 International Units (IU) per day, though a range of 400–800 IU/day is widely accepted as the safe maintenance dose for healthy growth.

Unlike infants under 1 year who need a strict 400 IU, children over 1 year can obtain some Vitamin D from a wider variety of foods and potentially more sunlight, so the dosage is slightly more flexible.

Recommended Dosage Guidelines

Standard Recommendation: 600 IU/day is the target intake set by major health organizations (such as the Institute of Medicine and the Endocrine Society) to maximize bone health .

Clinical Practice Range: Many clinical guidelines (including recent Chinese expert consensuses) recommend a daily supplement of 400–800 IU to prevent deficiency and support rapid growth .

Duration: This supplementation should continue through childhood and adolescence (up to 18 years old), as bones continue to develop and mineralize during these years .

Sources of Vitamin D for Toddlers and Older Children

As children transition to a solid-food diet, Vitamin D comes from three sources, which is why the strict "400 IU supplement" rule for infants changes:

Diet: Children over 1 year often drink cow's milk (which is often fortified with Vitamin D), eat eggs, fatty fish (like salmon), and fortified cereals.

Sunlight: Unlike infants who must avoid direct sun, toddlers and older children can safely play outdoors. 15–30 minutes of sunlight on arms and legs a few times a week contributes to Vitamin D synthesis .

Supplements: Since diet and sun are often inconsistent (e.g., cloudy weather, picky eating), daily supplements remain the most reliable way to ensure the 600 IU goal is met.

Special Considerations

Winter Months: In northern regions or during winter when sunlight is weak, the body's natural production drops significantly. During this time, sticking to the higher end of the range (600–800 IU) is often recommended .

Obesity or Medical Conditions: Children with obesity, or those taking certain medications (like anti-seizure drugs), may need significantly higher doses (2–3 times the standard amount) to maintain healthy levels .

Summary

For most healthy children over 1 year, aim for 600 IU daily. If you are using a standard supplement bottle that provides 400 IU or 500 IU, that is also sufficient to maintain health when combined with a normal diet.

## DeepSeek

For children older than 1 year, the recommended daily vitamin D intake generally ranges from 400 to 600 IU (10–15 mcg), depending on the guideline.

| Organization / Region | Daily Recommendation | Notes |

| :--- | :--- | :--- |

| American Academy of Pediatrics (AAP) | 600 IU (15 mcg) | Standard dose for children 1+ years |

| Chinese Pediatric Guidelines | 400–800 IU | Range commonly cited in Chinese guidance |

| Hong Kong Department of Health | 400–600 IU (10–15 mcg) | Practical range for healthy children |

| Endocrine Society (2024) | 600 IU (15 mcg) | For prevention of nutritional rickets in children 1–18 years |

Key Points:

- For a healthy child over 1 year old, 400 IU per day is a safe and effective baseline.

- 600 IU per day is also widely recommended and safe.

- Children with risk factors (e.g., obesity, dark skin, minimal sun exposure, malabsorption, chronic medications) may require higher doses (e.g., 1000–2000 IU/day) under medical guidance.

- The upper safe limit for children 1–3 years is 2500 IU/day, and for ages 4–8 years is 3000 IU/day (IOM). Routine supplementation of 400–600 IU is far below these limits.

## Doubao

Children over 1 year old up to adolescence: The routine daily preventive dose of vitamin D is 600 IU per day. For overweight children or those with high risk of insufficiency, pediatricians may suggest 600–800 IU per day under professional guidance.

# Question 33. Until what age should children continue vitamin D supplementation?

## ChatGPT

Children should generally continue vitamin D supplementation until at least 12 months of age, and sometimes beyond, depending on risk factors:

1. 0–12 months – All exclusively breastfed infants or those receiving insufficient fortified formula need daily supplementation (400 IU/day).

2. After 12 months – Supplementation may continue if:

- Sunlight exposure is limited

- Diet is inadequate in vitamin D

- The child has darker skin or lives in northern latitudes

- There are underlying medical conditions affecting vitamin D metabolism

In many cases, routine supplementation continues through early childhood to ensure adequate bone development, especially during periods of rapid growth. Pediatricians may adjust duration based on diet, sun exposure, and lab results.

## Gemini

The short answer is that vitamin D supplementation should ideally continue throughout childhood and into adolescence, and for many, throughout their entire lives.

While the delivery method (drops vs. chewables vs. pills) changes, the biological need for vitamin D remains constant. Here is a breakdown of the timeline:

1. The Critical "Window of Growth" (Ages 0–18)

Medical organizations like the American Academy of Pediatrics (AAP) recommend that children receive at least 400–600 IU daily until they reach physical maturity.

Ages 0–1: 400 IU. Essential for preventing infantile rickets.

Ages 1–18: 600 IU. Crucial for the "pubertal growth spurt" and building peak bone mass. About 40–50% of the adult skeleton is formed during the teenage years; if vitamin D is low during this time, a child may never reach their full genetic height or bone strength.

2. When Can Supplements Be Stopped?

In theory, a child can stop taking a dedicated supplement if they can consistently meet the 600 IU goal through their diet and environment. However, this is difficult to achieve. To stop supplements safely, a child would need:

High Intake of Fortified Foods: Drinking roughly 1 liter (4 cups) of fortified milk daily.

Frequent Sun Exposure: Regular outdoor activity without heavy sunscreen (which is often discouraged due to skin cancer risks).

A Diet Rich in Fatty Fish: Eating salmon, mackerel, or sardines multiple times a week.

The Reality: Most modern children, especially those in northern latitudes or those who spend significant time indoors (school/video games), do not meet these requirements and benefit from continuing a supplement indefinitely.

3. Factors That Extend the Need for Supplements

For many children, doctors recommend lifelong supplementation or continuing well into adulthood due to:

Geography: Living north of the 37th parallel (e.g., New York, London, Beijing), where winter sunlight cannot produce vitamin D.

Skin Tone: Children with darker skin (higher melanin) produce vitamin D more slowly and almost always require supplements longer.

Lifestyle: Consistent use of sunscreen or wearing clothing that covers most of the skin.

4. Summary Table: Age-Based Strategy

Final Advice

Think of vitamin D as "bone insurance." While there is no specific age at which it must stop, many pediatricians suggest keeping children on a daily multivitamin or vitamin D supplement throughout their school years to ensure they don't fall into deficiency during critical growth phases.

## Qianwen

Children should continue vitamin D supplementation at least until they reach adulthood (around 18 years old), and ideally, supplementation should continue throughout their entire life.

While many parents stop after toddlerhood, the need for vitamin D does not disappear once a child stops growing rapidly. Here is the breakdown of why long-term supplementation is recommended:

Minimum Target: Age 18 (End of Puberty)

Continued Bone Growth: Growth and bone mineralization continue well past infancy. The "growth plates" in bones typically do not close until the end of puberty (around age 18). Adequate vitamin D is essential during this entire period to ensure bones achieve their maximum density and strength .

Inconsistent Sun Exposure: Even though older children can play outside, modern lifestyles often involve significant indoor time (school, homework, screens). Additionally, the use of sunscreen and living in high-latitude areas (where sunlight is weak for much of the year) makes natural synthesis unreliable .

Ideal Goal: Lifelong Supplementation

Dietary Gaps: Very few foods naturally contain vitamin D (mostly fatty fish and egg yolks). Unless a person eats a very specific diet, it is difficult to meet daily requirements through food alone .

Adult Health: Vitamin D is not just for children; it is crucial for adults to maintain muscle function, immune health, and prevent osteoporosis later in life. Therefore, many experts recommend making vitamin D a lifelong habit, adjusting the dose as adults age (e.g., increasing to 800 IU/day after age 50 or 70) .

Practical Recommendation for Parents

Don't Stop at Age 3: A common mistake is stopping supplements when the child turns 3 or starts preschool.

Switch Formulas: Instead of buying infant drops, you can switch to chewable tablets, gummies, or softgels that are easier for older children to take.

Dosage: For children and adolescents (1–18 years), the recommended maintenance dose is typically 600–800 IU per day .

Summary: You do not need to worry about "weaning" a child off vitamin D. It is safe and beneficial to continue a daily supplement of 600–800 IU from infancy all the way through the teenage years.

## DeepSeek

Children should continue vitamin D supplementation at least until they stop growing (through adolescence), and many experts now recommend continuing it through age 18 years, or even for life. There is no single age at which all children should stop, as the decision depends on their diet, sun exposure, and risk factors.

Summary of recommendations:

| Organization / Source | Recommended Duration | Notes |

| :--- | :--- | :--- |

| NHS Scotland | Continue maintenance treatment until stopped growing | Explicitly links supplementation to the end of growth |

| Chinese Pediatric Expert Consensus (2024) | Supplement until 18 years of age (end of adolescence) | Based on high prevalence of deficiency in children >3 years old |

| American Academy of Pediatrics (AAP) | Infants: 400 IU/day; Children >1 year: 600 IU/day; continue through childhood and adolescence | Does not specify an endpoint; implies ongoing need |

| Endocrine Society (2024) | All children and adolescents (ages 1–18 years) should receive empirical supplementation | For prevention of rickets and potential respiratory benefits |

Why Supplementation Should Continue Through Adolescence:

1. Dietary intake alone is insufficient.

2. Sun exposure is unreliable.

3. Adolescence is a critical period for bone development (peak bone mass achievement).

When Might Supplementation Be Stopped? Supplementation may no longer be necessary if the child can reliably obtain adequate vitamin D from diet (e.g., daily consumption of vitamin D-fortified milk or other fortified products) or sun exposure (low latitude with year-round outdoor activity). However, given that most children do not meet the requirement through these sources, continued supplementation is the simpler and safer approach.

Daily Dosage by Age (for ongoing supplementation):

- 0–12 months: 400 IU

- 1–18 years: 400–800 IU (generally 600 IU)

Practical Takeaway: For most families, the simplest approach is to continue a daily vitamin D supplement (400–800 IU) through age 18 years, as recommended by the 2024 Chinese Pediatric Expert Consensus and the Endocrine Society.

## Doubao

Children should keep taking vitamin D supplementation throughout childhood and adolescence, up to 18 years of age. Many pediatric guidelines recommend lifelong adequate vitamin D intake, as bone growth, immune function and muscle health still rely on vitamin D well beyond early childhood. After age 1, the standard daily dose is 600 IU until adulthood.

# Question 34. How should an exclusively breastfed infant receive vitamin D supplementation?

## ChatGPT

An exclusively breastfed infant should receive vitamin D supplementation as follows:

1. Form – Use oral vitamin D drops specifically designed for infants.

2. Dose – 400 IU (10 µg) per day, starting in the first few days to weeks of life.

3. Administration – Drops can be placed directly into the infant’s mouth or added to a small amount of breast milk or formula.

4. Consistency – Give daily, as intermittent dosing is less effective.

5. Monitoring – Pediatricians may monitor growth, feeding, and at-risk factors; lab testing is usually not needed unless deficiency is suspected.

This regimen ensures adequate vitamin D for bone mineralization, growth, and prevention of rickets.

## Gemini

For an exclusively breastfed infant, vitamin D supplementation is a direct requirement because breast milk, while nutritionally perfect in almost every other way, does not provide enough vitamin D to prevent rickets.

The American Academy of Pediatrics (AAP) recommends that these infants receive 400 IU of vitamin D daily, starting within the first few days of life.

1. Delivery Methods

Since infants cannot swallow pills or eat fortified solids, the supplement must be delivered in liquid form. There are two primary ways to administer these drops:

Directly into the Mouth: Use the dropper provided by the manufacturer to place the liquid directly into the baby's mouth. Aim for the inside of the cheek rather than the back of the throat to prevent choking or spitting it out.

Via the Nipple (Breastfeeding): Many parents find it easiest to place a single drop of a concentrated vitamin D supplement directly onto the mother's nipple or a clean finger just before the baby latches on for a feeding.

2. Choosing the Right Product

There are two main types of liquid vitamin D supplements available:

Highly Concentrated (Single-Drop): These provide the full 400 IU in just one drop. These are highly recommended for breastfed infants because they are easy to administer and the baby is less likely to spit out such a tiny amount.

Standard Concentration (1.0 mL dose): These require a larger volume (usually 1 full dropper) to reach 400 IU. While effective, some babies may dislike the taste or the volume of the liquid.

3. Safety Precautions for Parents

Use the Included Dropper: Never use a dropper from a different brand or product. Droppers are calibrated specifically for the concentration of the liquid in that specific bottle. Using the wrong one can lead to a dangerous overdose.

Check the Label: Ensure the product contains Vitamin D3 (cholecalciferol), which is the form most effectively used by the human body, rather than Vitamin D2.

Avoid "Adec" or Multivitamins Unless Prescribed: If the baby only needs vitamin D, a "Vitamin D only" supplement is preferred over a multivitamin (like Poly-Vi-Sol) to avoid giving unnecessary nutrients.

4. Can the mother just take extra Vitamin D instead?

Technically, yes, but it is not the standard recommendation.

For a mother to transfer enough vitamin D through her breast milk to meet the baby’s 400 IU requirement, she would typically need to take a very high dose (roughly 6,400 IU per day).

Because most women do not take such high doses, and because absorption varies between individuals, pediatricians strongly prefer giving the drop directly to the baby to ensure the dose is accurate and consistent.

Summary

The most reliable way to supplement an exclusively breastfed infant is a single daily drop of 400 IU Vitamin D3, administered either directly into the infant's mouth or onto the breast during a feeding, starting in the first week of life.

## Qianwen

For an exclusively breastfed infant, vitamin D supplementation should be administered as a daily oral dose of 400 International Units (IU), typically in the form of drops, starting within the first few days of life.

Since breast milk naturally contains very little vitamin D, this direct supplementation is essential to prevent rickets and ensure proper bone growth .

Administration Methods

The goal is to ensure the baby ingests the correct dose safely and comfortably.

Direct Oral Administration: The most common method is to place the drop directly into the baby's cheek or on the tongue.

Capsules: If using gel capsules, do not feed the shell to the baby. Cut the tip with scissors or dip it in warm water to melt the seal, then squeeze the oil directly into the mouth .

Droppers: If using a bottle, use the calibrated dropper to measure exactly 400 IU (usually 1 drop or 1ml depending on the brand concentration).

Indirect Methods: If the baby resists the dropper, the vitamin D can be placed on a clean finger for the baby to suck, or applied to the mother's nipple before nursing .

Timing and Consistency

Start Time: Supplementation should begin shortly after birth, typically within the first 1–2 weeks .

Relation to Feeding: It can be given before or after breastfeeding. Giving it just before a feed is often convenient as it integrates into the feeding routine .

Frequency: Daily administration is preferred. While some guidelines allow for weekly high-dose administration (e.g., 2800 IU once a week) for those who struggle with daily dosing, daily drops are the standard recommendation for consistency and safety .

Duration

This daily supplementation should continue until the infant is weaned to at least 1 liter (32 oz) of vitamin D-fortified formula or cow's milk per day. Since exclusively breastfed infants do not consume this volume of fortified milk, they generally continue the 400 IU drops until age 1 (and often beyond) .

Safety Note

Avoid Overdose: Stick to the 400 IU recommendation. While vitamin D toxicity is rare, excessive intake can cause hypercalcemia .

No Need for Calcium: Pure breast milk provides sufficient calcium; the vitamin D is added solely to help the body absorb that calcium effectively .

## DeepSeek

An exclusively breastfed infant should receive a daily oral vitamin D supplement of 400 IU (10 mcg), starting soon after birth and continuing until the baby is weaned or drinks enough fortified formula.

Why Supplementation Is Necessary: Breast milk is the ideal food for infants, but it contains very little vitamin D—typically not enough to meet a baby's daily requirement. Exclusively breastfed infants are at particular risk for vitamin D deficiency and rickets without adequate supplementation. A longitudinal study found that among exclusively breastfed infants who did not receive supplements, none achieved vitamin D sufficiency by 5 months of age. However, with daily 400 IU supplementation starting at 1 month, 93.3% achieved sufficiency by 5 months.

How to Give the Supplement: Vitamin D supplements for infants come as liquid drops. Recommended methods:

- Directly into the mouth: Squeeze one drop directly into the baby's mouth.

- Mixed with milk: Mix the drop with a small amount of breastmilk or formula in a sterilized spoon.

- Mixed with food: For babies over 6 months, mix with pureed foods on a spoon.

- On the nipple: Place the drop on the nipple or areola and let the baby suckle for 30 seconds.

Important Safety Precautions:

- Do not exceed 400 IU per day unless directed by a doctor.

- Use only the dropper that comes with the supplement.

- Avoid giving other vitamin D-containing products such as cod liver oil or multivitamins.

- Store properly — most liquid vitamin D drops should be refrigerated after opening and discarded after 5 months.

Special Note for Formula-Fed Infants: For formula-fed infants, supplementation is generally not needed if the baby drinks at least 32 ounces (about 1 liter) of vitamin D-fortified formula per day. If drinking less than that amount, 400 IU daily supplementation is recommended.

## Doubao

Exclusively breastfed infants cannot get enough vitamin D from breast milk alone, so they need direct supplementation. 1. Start supplementation within the first 1–2 weeks after birth. 2. Daily standard dose: 400 IU per day for infants aged 0–12 months. 3. Give vitamin D drops directly to the baby orally, do not rely on maternal supplementation alone (taking vitamin D by the mother cannot pass enough to breast milk to meet the infant’s needs). 4. Keep daily supplementation continuously throughout infancy, no interruption in winter or less sunlight seasons.

# Question 35. How should vitamin D intake be calculated for a mixed-fed infant?

## ChatGPT

For a mixed-fed infant (receiving both breast milk and formula), vitamin D intake should be calculated based on total daily intake from all sources:

1. Determine formula contribution – Most infant formulas are fortified with about 400 IU of vitamin D per liter. Calculate the amount consumed per day.

2. Determine breast milk contribution – Breast milk alone provides very little vitamin D (~40 IU/L), so it is generally insufficient.

3. Supplementation – Provide additional vitamin D drops so that the total daily intake reaches 400 IU (10 µg).

4. Example – If an infant consumes 500 mL of fortified formula (≈200 IU vitamin D), give 200 IU via supplement to reach the total recommended 400 IU/day.

5. Consistency and monitoring – Give daily and adjust if formula intake changes; pediatricians may monitor growth or labs if there are risk factors.

This approach ensures the infant receives adequate vitamin D from both diet and supplementation to prevent deficiency.

## Gemini

Calculating vitamin D for a mixed-fed (breast milk + formula) infant can be slightly confusing because you have to account for the vitamin D already present in the formula.

The goal remains a total of 400 IU per day. Here is the step-by-step logic for parents and caregivers:

1. The "One Liter" Rule

In the United States and many other regions, infant formula is fortified with vitamin D at a concentration of 400 IU per Liter.

If your baby drinks 1 Liter (approx. 33–34 oz) of formula daily: They are getting their full 400 IU and do not need a supplement.

If your baby drinks less than 1 Liter: They are not getting enough from formula alone and will likely need a supplement.

2. How to Calculate the Gap

Because it is difficult to "micro-dose" vitamin D drops (they usually come in fixed 400 IU doses), pediatricians typically use a simplified threshold:

Scenario A: Formula provides >50% of intake. If your baby is drinking more than 500 mL (approx. 17 oz) of formula a day, consult your pediatrician. In some cases, if the intake is very close to a full liter, the supplement might be skipped, though many doctors still recommend it just to be safe, as 800 IU (400 from formula + 400 from a drop) is still well within the safe range.

Scenario B: Breast milk provides >50% of intake. If your baby is mostly breastfed and only takes a few ounces of formula, they should receive the full 400 IU supplement daily. The small amount of vitamin D in the occasional bottle of formula is not enough to cause concern about "overdosing."

3. Simplified Decision Table

4. Safety and "Overlapping" Doses

A common concern for parents of mixed-fed babies is: "What if my baby gets 400 IU from the drop AND 300 IU from formula? Is 700 IU too much?"

The answer is No. The "Tolerable Upper Intake Level" for infants 0–6 months is 1,000 IU daily, and for 6–12 months, it is 1,500 IU daily.

Receiving a daily total of 600–800 IU is perfectly safe and often preferred over the risk of being deficient.

Summary

Unless your baby is consistently consuming a full quart or liter (approx. 33 oz) of formula every single day, you should continue to give the 400 IU vitamin D supplement. If you are unsure, it is always safer to provide the supplement, as the margin for safety with vitamin D in infants is quite wide.

## Qianwen

For a mixed-fed infant, vitamin D intake should be calculated by determining the amount provided by the formula and supplementing the difference to reach the recommended daily total of 400 IU.

The calculation follows a simple "Total Required minus Formula Contribution" logic.

The Calculation Formula

Supplement Dose = 400 IU − (Daily Formula Volume × Vitamin D Concentration per ml)

Target Goal: 400 IU/day (standard recommendation for infants).

Formula Contribution: Most standard infant formulas contain approximately 40–60 IU of Vitamin D per 100 ml.

Practical Examples

Assuming a standard formula concentration of roughly 50 IU/100ml:

Scenario A: High Formula Intake (e.g., 800 ml/day)

Vitamin D from formula: $8 \times 50 \text{ IU} = 400 \text{ IU}$.

Supplement needed: $400 - 400 = 0 \text{ IU}$. (No supplement needed).

Scenario B: Moderate Formula Intake (e.g., 500 ml/day)

Vitamin D from formula: $5 \times 50 \text{ IU} = 250 \text{ IU}$.

Supplement needed: $400 - 250 = 150 \text{ IU}$. (Supplement with ~150 IU).

Scenario C: Low Formula Intake (e.g., 200 ml/day)

Vitamin D from formula: $2 \times 50 \text{ IU} = 100 \text{ IU}$.

Supplement needed: $400 - 100 = 300 \text{ IU}$. (Supplement with ~300 IU).

Step-by-Step Action Plan

Check the Label: Look at the nutrition facts on the formula canister to confirm the exact Vitamin D content per 100 ml.

Track Daily Volume: Estimate the average amount of formula the baby drinks per day over a few days.

Calculate the Gap: Use the formula above to find the missing amount.

Adjust Supplementation: Give the calculated amount via Vitamin D3 drops.

Important Considerations

Safety First: It is generally safe to provide a full 400 IU drop even if the baby has had some formula, as Vitamin D toxicity is rare at this level. However, precise calculation helps avoid unnecessary supplementation.

Special Populations: If the infant is a premature baby or has specific metabolic issues, the dosage requirements may differ (often higher initially), and a pediatrician's specific instruction should be followed.

## DeepSeek

For a mixed-fed infant (receiving both breast milk and infant formula), you should calculate the daily vitamin D intake from formula and supplement the difference to reach the total daily requirement of 400 IU (10 mcg).

Step-by-step calculation method:

Step 1: Determine the daily vitamin D requirement: All infants under 12 months of age need 400 IU (10 mcg) of vitamin D per day.

Step 2: Calculate how much vitamin D the infant gets from formula.

- Find the vitamin D concentration on the formula label. Standard infant formula is fortified with vitamin D, typically containing around 400 IU per liter (about 40–50 IU per 100 ml). Always check the specific brand's label.

- Estimate the infant's average daily formula intake (in ml or oz).

- Calculate: (Daily formula intake in liters) × 400 IU = Vitamin D from formula.

Or more precisely: (Daily formula intake in ml ÷ 100 ml) × Vitamin D (IU) per 100 ml = Vitamin D from formula.

Example: If an infant drinks 500 ml of formula per day, and the label states the formula contains 53 IU per 100 ml:

(500 ml ÷ 100 ml) × 53 IU = 5 × 53 IU = 265 IU from formula per day.

Step 3: Calculate the remaining vitamin D needed: 400 IU (total needed) — Vitamin D from formula = Vitamin D supplement needed.

Using the example above: 400 IU — 265 IU = 135 IU of supplemental vitamin D needed per day.

Step 4: Practical supplementation guidelines. Because calculating exact amounts daily can be challenging, several health organizations provide practical cut-off values:

| Daily Formula Intake | Practical Supplementation Recommendation |

| :--- | :--- |

| Less than 500 ml (approx. 16-17 oz) | Continue giving the full 400 IU supplement daily. |

| 500 ml to 1,000 ml | The supplement amount needed depends on exact intake and formula concentration; most guidelines recommend giving a partial supplement or the full 400 IU dose every few days to compensate for the deficit. |

| 1,000 ml (approx. 32 oz) or more | Supplementation is generally not needed, as the infant meets the 400 IU requirement from formula alone. |

Important Clinical Considerations:

- Safety limits: The safe upper limit for infants 0–6 months is 1,000 IU per day and for infants 6–12 months is 1,500 IU per day. While standard supplementation is safe, avoid giving additional vitamin D-containing products like cod liver oil or multivitamins without consulting your pediatrician.

- Inconsistent intake: If your infant's daily formula intake varies significantly, the safest approach is to continue the full 400 IU supplement consistently, as any excess vitamin D from formula on high-intake days will remain within safe limits and will be compensated for on lower-intake days.

## Doubao

1. First, check the vitamin D content per 100 mL of the formula milk the baby takes. 2. Calculate total daily vitamin D the infant gets from formula: Total from formula = Daily formula intake(mL) ÷ 100 × Vitamin D per 100 mL 3. The target daily intake for 0–12 months is 400 IU. 4. Subtract the formula vitamin D from 400 IU; the remaining gap is what needs extra oral vitamin D drops. 5. If the formula already provides ≥ 400 IU per day, no extra supplementation is needed. 6. Breast milk contributes almost no vitamin D, so only formula intake is counted for calculation.

# Question 36. Does an infant who drinks enough formula still need extra vitamin D supplementation?

## ChatGPT

It depends on how much formula the infant consumes:

- If the infant drinks ≥1 liter (≈33 oz) of vitamin D–fortified formula per day, they usually do not need extra supplementation, because this provides roughly 400 IU/day.

- If the intake is less than 1 liter per day, additional vitamin D drops are recommended to reach the daily requirement of 400 IU (10 µg).

- Monitoring intake is important, especially for mixed-fed infants, preterm infants, or those with risk factors for deficiency.

In short, sufficient formula intake can meet vitamin D needs, but most infants require supplementation unless they consume the full recommended volume of fortified formula.

## Gemini

Generally, no. An infant who consumes a sufficient amount of fortified formula does not need an additional vitamin D supplement. However, the definition of "enough" is very specific.

In the United States and many other countries, infant formula is mandated to be fortified with vitamin D (usually 400 IU per Liter). Therefore, whether a baby needs a supplement depends entirely on their daily volume of intake.

1. The "One Liter" Threshold

According to the American Academy of Pediatrics (AAP), a formula-fed infant only meets their daily requirement without a supplement if they consume:

At least 1 Liter (approximately 33–34 ounces) of fortified formula per day.

2. Why many formula-fed babies still need drops

While formula contains vitamin D, many babies do not actually reach the one-liter mark, especially in the early months:

Newborns: In the first few weeks or months, babies typically drink much less than 33 ounces a day. During this period, they are not getting the full 400 IU from their bottles and should receive a supplement.

Small Appetites: Some infants naturally have smaller appetites or are smaller in size and may never reach a full liter of intake daily.

Transition to Solids: Once a baby starts solid foods (around 6 months), their formula intake often decreases. If their formula volume drops below 33 ounces, they may need to resume or continue supplementation.

3. Summary of Recommendations

4. Is "Double Dosing" Dangerous?

Many parents worry that if their baby drinks, for example, 25 ounces of formula (providing ~300 IU) and they also give a 400 IU drop, the total (700 IU) is too much.

It is safe. The "Upper Limit" for safety is 1,000 IU per day for infants under 6 months and 1,500 IU for those aged 6–12 months.

Pediatricians generally prefer a baby to get a bit "extra" (e.g., 700–800 IU total) than to risk a deficiency by receiving only 200–300 IU.

Final Guidance

If your baby is consistently drinking a full quart (32–34 oz) of formula every day, you can stop the vitamin D drops. If they drink less than that, or if they are mixed-fed with breast milk, you should continue the 400 IU daily supplement to ensure their bones have the "bricks" they need to grow.

## Qianwen

Yes, an infant who drinks "enough" formula usually still needs extra supplementation, unless that "enough" amount is a consistent 1000 ml (1 liter) or more every single day.

While formula is fortified with Vitamin D, the concentration is generally not high enough to meet the daily requirement of 400 IU unless the baby consumes a very large volume that is often physically impossible for young infants to drink.

The Math Behind the Decision

To understand why supplementation is usually required, we have to look at the numbers:

Target: The recommended daily intake is 400 IU.

Concentration: Most standard formulas contain about 40 IU of Vitamin D per 100 ml.

Why 1000 ml is Rarely "Enough"

For most infants, especially those under 6 months, drinking 1000 ml of milk daily is unrealistic due to stomach capacity and appetite:

Newborns (0–1 month): Average intake is only 300–500 ml/day. (Provides only ~120–200 IU).

1–3 Months: Average intake is 500–800 ml/day. (Provides only ~200–320 IU).

4–6 Months: Average intake is 800–1000 ml/day. (Provides ~320–400 IU).

Conclusion: Unless the baby is consistently drinking a full liter of milk, there is a "Vitamin D Gap" that must be filled with supplements .

The "Safe & Simple" Strategy

While you can calculate the exact difference (as discussed in the previous answer regarding mixed feeding), most pediatricians recommend a simpler approach:

Just Supplement 400 IU: It is perfectly safe to give a full 400 IU drop daily, even if the baby has had 500 ml of formula.

If the baby drinks little formula, they get their full requirement.

If the baby drinks 500 ml of formula (getting 200 IU from milk), the supplement brings them to 600 IU total. This is well within the safe upper limit for infants (1000 IU/day).

Only Stop if Volume is High: You generally only need to skip the supplement if the baby is reliably drinking >1000 ml of formula daily .

Summary: "Enough" milk for hunger doesn't always mean "enough" milk for Vitamin D.

Unless the baby is drinking a massive amount (1 liter+), continuing the daily Vitamin D drops is the safest and most effective strategy.

## DeepSeek

No, an infant who drinks enough vitamin D-fortified formula does not need extra vitamin D supplementation.

The general rule from the American Academy of Pediatrics (AAP) and other major health organizations is that if an infant drinks at least 1 liter (about 32 ounces) of vitamin D-fortified formula per day, supplementation is not needed. Standard infant formula is fortified to provide approximately 400 IU of vitamin D per liter, which exactly meets the infant's daily requirement.

Summary based on feeding type:

| Feeding Type | Vitamin D Supplementation Needed |

| :--- | :--- |

| Exclusively breastfed | Yes – 400 IU daily, starting soon after birth |

| Mixed-fed (breast milk + formula) | Yes – 400 IU daily, unless formula intake reaches 1 liter/day |

| Formula-fed, drinking ≥ 1 liter/day | No – Formula alone provides the required 400 IU |

| Formula-fed, drinking < 1 liter/day | Yes – 400 IU daily until reaching 1 liter/day |

Why 1 Liter is the cutoff: Standard infant formula contains approximately 400 IU of vitamin D per liter when prepared according to instructions. At 1 liter per day, the infant receives exactly 400 IU from formula alone, meeting the daily requirement. Below 1 liter per day, the infant receives less than 400 IU, requiring supplementation.

Important Notes:

- Check the formula label – While most formulas contain ~400 IU/L, concentrations can range from 40–100 IU per 100 mL. Always verify the specific vitamin D content.

- This applies only to fortified infant formula – Once an infant transitions to cow's milk (after 12 months), vitamin D needs change. Children 12–24 months need 600 IU daily, and cow's milk is also fortified.

- Start supplementation early if needed – For infants who do not yet drink 1 liter of formula per day (common in the first few months), supplementation should begin soon after birth.

Practical Takeaway: If your formula-fed infant drinks 32 ounces (about 1 liter) or more of fortified formula daily, you do not need to give additional vitamin D drops. If intake varies day to day, the safest approach is to continue the supplement, as the combined amount will remain well within the safe upper limit (1000–1500 IU/day for infants). When in doubt, consult your pediatrician.

## Doubao

If a baby drinks enough infant formula every day, extra vitamin D may not be needed — otherwise supplementation is still required. Rule of thumb Most standard infant formula contains about 40–50 IU of vitamin D per 100 mL. To get the full 400 IU daily requirement: - If the baby drinks ≥ 1000 mL formula per day: usually gets enough vitamin D naturally, no extra drops needed. - If intake is less than 800–1000 mL per day: still lacks vitamin D, need extra supplementation to reach 400 IU per day. Key point Formula-fed babies who do not drink large volumes of formula still need routine vitamin D drops just like breastfed infants.

# Question 37. What is the difference between vitamin D2 and vitamin D3?

## ChatGPT

The difference between vitamin D2 (ergocalciferol) and vitamin D3 (cholecalciferol) lies in their source, structure, and effectiveness:

1. Source

- Vitamin D2 – Derived from plant sources (e.g., fungi, yeast, fortified foods).

- Vitamin D3 – Derived from animal sources (e.g., fish oil, cod liver oil, lanolin) or synthesized in the skin from sunlight (UVB exposure).

2. Potency and efficacy

- D3 is more potent and raises serum 25-hydroxyvitamin D levels more effectively than D2.

- D2 has a shorter half-life in the blood and may require higher or more frequent doses to achieve the same effect.

3. Use in supplementation

- Both forms are used in vitamin D supplements, but D3 is generally preferred for long-term maintenance and treatment of deficiency, especially in children.

In summary, D3 is more effective and longer-lasting, while D2 is plant-based and less potent.

## Gemini

The primary difference between Vitamin D2 and Vitamin D3 lies in their source and their biological effectiveness in the human body. While both can treat deficiency, most pediatricians prefer Vitamin D3 for children.

1. The Source

Vitamin D2 (Ergocalciferol): This is derived from plant sources, specifically fungi and yeast. When mushrooms or yeast are exposed to UV light, they produce D2. It is often the version used in vegan-specific supplements and some prescription-strength formulations.

Vitamin D3 (Cholecalciferol): This is the "human" form of vitamin D. It is produced naturally in our skin when exposed to sunlight. In supplement form, it is typically derived from lanolin (sheep’s wool) or lichen (a vegan D3 source).

2. Potency and Effectiveness

Raising Blood Levels: Research consistently shows that Vitamin D3 is more effective at raising and maintaining the total 25-hydroxyvitamin D [25(OH)D] levels in the blood compared to D2.

Shelf Life and Stability: Vitamin D3 is more stable and has a longer half-life in the body. Vitamin D2 is cleared from the bloodstream more quickly, meaning its effects may not last as long between doses.

3. Usage in Children

Standard Drops: Almost all over-the-counter infant vitamin D drops use Vitamin D3 because it is more potent and mimics the form produced by the sun.

Prescriptions: Historically, many high-dose prescription "loading doses" (e.g., 50,000 IU) were only available as Vitamin D2. However, high-dose D3 is now widely available and is generally preferred by specialists for treating nutritional rickets.

Comparison Summary

Which should you choose?

For your child, Vitamin D3 is almost always the better choice. It is the form the body is naturally designed to use and is significantly more efficient at building the bone mineral stores needed for growth. If you are using a vegan supplement, look specifically for Vegan D3 (from lichen) rather than D2 for the best results.

## Qianwen

Vitamin D3 (cholecalciferol) is generally preferred over Vitamin D2 (ergocalciferol) for infants because it is more potent, more stable, and more efficiently utilized by the body to raise blood levels.

While both forms are effective at preventing rickets and deficiency when taken in adequate amounts, they differ significantly in their biological activity and sources.

Efficacy and Bioavailability

Absorption and Half-life: Vitamin D3 is chemically identical to the form of vitamin D synthesized by human skin when exposed to sunlight. Consequently, the body absorbs and utilizes it more effectively. It has a longer half-life in the blood compared to D2.

Potency: Studies indicate that Vitamin D3 is approximately 30–80% more effective than Vitamin D2 at raising and maintaining total serum 25-hydroxyvitamin D levels. Vitamin D2 is more prone to degradation during storage and has a more variable effect on blood levels.

Sources of Origin

Vitamin D3: Derived from animal sources (such as fish oil, egg yolks, and liver) or produced synthetically (often from lanolin found in sheep's wool).

Vitamin D2: Derived exclusively from plant sources (specifically fungi and yeast exposed to UV light).

Clinical Recommendation for Infants

Standard of Care: Because of its superior efficacy and stability, Vitamin D3 is the standard recommendation for treating and preventing deficiency in children and infants .

Exception for Vegans: Vitamin D2 is typically reserved for families adhering to a strict vegan lifestyle who wish to avoid animal-derived supplements (like lanolin-based D3). However, to achieve the same blood levels as D3, higher or more frequent doses of D2 may be required.

Summary: Unless there is a specific dietary restriction against animal products, Vitamin D3 is the superior choice for ensuring optimal vitamin D status in infants.

## DeepSeek

Vitamin D2 (ergocalciferol) and vitamin D3 (cholecalciferol) are the two main forms of vitamin D used in supplements and fortified foods. While both can effectively treat and prevent vitamin D deficiency, they differ in their source, chemical structure, production method, and potency.

Key differences:

| Feature | Vitamin D2 (Ergocalciferol) | Vitamin D3 (Cholecalciferol) |

| :--- | :--- | :--- |

| Source | Plant-based (fungi, yeast, UV-exposed mushrooms) | Animal-based (sheep's wool lanolin, fish oil) or lichen (vegan) |

| Chemical structure | Different side-chain structure | Identical to the vitamin D produced by human skin |

| Production | UV irradiation of ergosterol (found in fungi) | UV irradiation of 7-dehydrocholesterol (lanolin) |

| Metabolism | Shorter half-life in blood (~1-2 days) | Longer half-life in blood (~2-3 weeks) |

| Potency | Less potent per IU; requires higher or more frequent dosing | More potent; raises and maintains 25(OH)D levels more effectively |

| Cost | Generally less expensive | Slightly more expensive, but highly affordable |

Effectiveness Comparison: Although both forms have been considered equivalent when given as a daily dose, a growing body of evidence suggests that vitamin D3 is more effective at raising and maintaining serum 25(OH)D levels, especially at higher doses. A 2021 meta-analysis found that vitamin D3 produced a more sustained increase in 25(OH)D levels after a single high dose (≥300,000 IU) compared to D2. Vitamin D2 has a shorter half-life in the blood (about 1-2 days) compared to D3 (about 2-3 weeks). A large meta-analysis (including 75 randomized controlled trials) found that vitamin D3 was associated with a significant 11% reduction in all-cause mortality, while D2 showed no such benefit.

Recommendations for Children:

- Chinese Pediatric Guidelines (2024): Prefers vitamin D3 for routine supplementation, as D3 has a longer half-life and better efficacy.

- American Academy of Pediatrics (AAP): Recommends daily 400 IU supplementation for infants, without specifying a preference, but D3 is commonly used in available pediatric drops.

- Canadian Pediatric Society (2022): Recommends 400 IU/day of D3 for breastfed infants and children over 1 year.

Practical Takeaway: For children, vitamin D3 is generally preferred due to its longer half-life, greater potency, and superior ability to maintain adequate 25(OH)D levels. Most commercially available pediatric vitamin D drops use D3. However, vitamin D2 remains a suitable alternative, especially for vegan families, as D3 derived from lichen is the only vegan source of D3.

## Doubao

Vitamin D2 vs Vitamin D3: Key Differences 1. Source - Vitamin D2 (Ergocalciferol): Made from plant fungi/yeast; plant-based. - Vitamin D3 (Cholecalciferol): From animal sources (lanolin, fatty fish, egg yolks) and skin synthesis via sunlight. 2. Potency & Half-life - D3 has a longer half-life and stays in the body longer. - D3 raises blood 25(OH)D levels more effectively and steadily than D2. 3. Stability & Effectiveness - D2 breaks down more easily under light/heat. - D3 is more stable and the preferred form for children, infants and daily supplementation. 4. Clinical Use - Both can treat vitamin D deficiency. - But pediatric guidelines strongly recommend Vitamin D3 for routine daily supplementation in kids. 5. Vegetarian note Only D2 is suitable for strict vegetarians; most D3 is animal-derived.

# Question 38. If vitamin D deficiency has already been diagnosed, is a preventive dose still enough?

## ChatGPT

No, a preventive (maintenance) dose is usually not enough once vitamin D deficiency has been diagnosed.

- Preventive dose (e.g., 400–600 IU/day) is intended to maintain adequate levels in healthy children, not to correct an existing deficiency.

- Treatment of deficiency typically requires a higher therapeutic dose for a defined period to restore normal serum 25-hydroxyvitamin D levels.

- For example, infants or children with deficiency may receive 2,000 IU/day or more, or intermittent high-dose therapy under medical supervision.

- After correction, the child is usually switched back to a maintenance dose to prevent recurrence.

Using only a preventive dose without therapeutic supplementation may fail to correct the deficiency, prolong bone mineralization problems, and increase the risk of rickets or other complications.

## Gemini

No, if a child has already been diagnosed with vitamin D deficiency, a standard preventive dose (400–600 IU) is not enough.

A preventive dose is designed to maintain levels in a healthy child, but it is insufficient to correct a pre-existing deficit or rebuild the body's depleted stores. Treatment requires a different strategy.

1. Treatment Dose vs. Maintenance Dose

Preventive (Maintenance) Dose: 400–600 IU daily. This is intended to replace the vitamin D the body uses up every day.

Therapeutic (Treatment) Dose: This is much higher—often ranging from 2,000 IU to 6,000 IU daily, or sometimes a high-dose "stoss" therapy (a large one-time dose), depending on the child's age and the severity of the deficiency.

2. The "Refilling the Tank" Analogy

Think of vitamin D like the fuel in a car:

Prevention is like adding a little gas every day to keep the tank from going empty.

Treatment is what you do when the tank is already empty. You cannot refill an empty tank by only adding the exact amount of gas needed for that day's drive; you must add a large volume upfront to get back to a "full" status.

3. What happens if you only use a preventive dose?

If a child with a 25(OH)D level of, for example, 12 ng/mL (deficiency) only takes 400 IU:

Their blood levels may stop falling, but they will likely stay in the "danger zone" for a long time.

During this time, the body may continue to pull calcium from the bones to compensate for the low vitamin D, meaning the risk of bone softening or rickets remains active.

4. Typical Treatment Protocol

When a deficiency is diagnosed, a pediatrician will usually follow a three-step process:

Loading Phase: Prescribe a high therapeutic dose for 8 to 12 weeks to rapidly raise blood levels and restock the liver and fat tissues.

Re-testing: Blood markers (25(OH)D, Calcium, ALP) are checked after 3 months to ensure the "tank is full."

Maintenance Phase: Once levels are normal (usually >30 ng/mL), the child is moved back down to the standard preventive dose (400–600 IU) to keep them there.

5. Essential Addition: Calcium

If a child is deficient, the doctor will often recommend increasing calcium intake alongside the high-dose vitamin D. Without enough calcium, the body cannot take advantage of the new vitamin D to repair and harden the bones.

Summary

If your child has a confirmed deficiency, follow the prescription dose provided by your doctor, which will be significantly higher than the standard over-the-counter daily recommendation. Only return to the "preventive" amount once the doctor confirms the deficiency has been fully corrected.

## Qianwen

No, a preventive dose (typically 400 IU/day) is generally not enough to treat an existing deficiency.

While 400 IU is sufficient for maintaining health in a healthy child, it is usually too low to effectively raise blood levels in a child who is already deficient.

Treating a deficiency requires a "loading dose"—a significantly higher amount of Vitamin D for a specific period to replenish the body's reserves, followed by a return to the preventive dose.

Treatment vs. Prevention: The Difference

Prevention (Maintenance): The goal is simply to replace what is used daily. 400–800 IU/day is the standard maintenance dose to keep levels stable .

Treatment (Correction): The goal is to raise serum 25(OH)D levels above 20 ng/mL (50 nmol/L) as quickly and safely as possible. To achieve this, clinical guidelines recommend much higher doses .

Standard Treatment Protocols

The specific treatment regimen depends on the severity of the deficiency and the child's age, but it generally follows a two-step process:

High-Dose Treatment Phase:

Daily Dosing: Often 2,000 IU/day for 6–8 weeks for children aged 1–18 years (and 2,000 IU/day for infants 0–1 year) .

Alternative (Stoss Therapy): Some protocols use a weekly dose (e.g., 50,000 IU once a week for 6 weeks) or a single large oral dose, though daily dosing is often preferred for better safety monitoring .

Special Cases: Children with malabsorption issues (e.g., celiac disease, cystic fibrosis) or obesity may require even higher doses (2–3 times the standard treatment dose) .

Maintenance Phase:

Once the deficiency is corrected (usually re-evaluated after 3 months), the dosage is reduced back to the standard preventive level (400–1,000 IU/day) to maintain healthy levels .

Important Safety Warning

Medical Supervision Required: Unlike preventive supplementation, which can be done over the counter, treatment dosing must be prescribed by a doctor.

Risk of Toxicity: While rare, taking high treatment doses for too long without monitoring can lead to Vitamin D toxicity (hypercalcemia), which can damage the kidneys .

Monitoring: Doctors typically recommend re-testing blood levels after the treatment phase to ensure the dose is no longer needed or to adjust the maintenance dose accordingly .

Summary: A preventive dose is like a daily meal to keep you from getting hungry; a treatment dose is like a medical IV drip to cure an illness.

If a deficiency has been diagnosed, follow the doctor's specific high-dose prescription rather than relying on the standard daily drop.

## DeepSeek

No, once vitamin D deficiency has been diagnosed, a standard preventive dose is generally not enough to correct the deficiency. Treatment requires a higher therapeutic dose, typically for a limited period (e.g., 6–12 weeks), followed by a return to a preventive dose once levels normalize.

Comparison of preventive vs. therapeutic dosing:

| Aspect | Preventive Dose | Therapeutic Dose (for known deficiency) |

| :--- | :--- | :--- |

| Purpose | Maintain normal levels in healthy children | Correct low levels in children with confirmed deficiency |

| Typical daily dose | 400–800 IU (depending on age) | 1000–2000 IU daily for 6–12 weeks (or higher intermittent doses) |

| Duration | Ongoing (through adolescence) | Limited (typically 8–12 weeks, then reassess) |

| Monitoring | Usually not required | Repeat 25(OH)D level after 3–4 months to confirm correction |

| When used | Routine prevention | Following confirmed low 25(OH)D level (<50 nmol/L or <20 ng/mL) or rickets |

Specific Therapeutic Regimens by Scenario

1. Simple Vitamin D Deficiency (no rickets):

- Infants (0–12 months): 1000–2000 IU daily for 6–12 weeks, then 400 IU daily maintenance.

- Children >1 year: 2000 IU daily for 6–12 weeks, then 600–1000 IU daily maintenance.

2. Nutritional Rickets (with bone deformities or radiological changes):

- Vitamin D: 2000–6000 IU daily for 8–12 weeks, or single high-dose (Stoss therapy: 150,000–300,000 IU orally or intramuscularly over 1–2 days).

- Calcium: 30–75 mg/kg/day (typically 500–1000 mg daily) for the first 2–4 weeks to prevent "hungry bone syndrome."

- Monitoring: Check serum calcium, phosphorus, ALP, PTH, and 25(OH)D at 3 months; repeat X-ray if baseline was abnormal.

Why a Preventive Dose Is Not Enough: Deficiency represents a depleted body store. The preventive dose is calculated to match daily requirements for a child with normal stores. In deficiency, stores are empty, and additional vitamin D is needed to replenish them before maintenance can begin. Short-term therapeutic doses (e.g., 2000 IU daily for 8 weeks) are far below the safe upper limits for children and are not associated with toxicity.

Important Safety Notes:

- Do not use very high doses (e.g., 50,000 IU or 300,000 IU single dose) without medical supervision – these are reserved for rickets or severe deficiency under direct pediatrician guidance.

- Always confirm deficiency before starting therapeutic doses.

- Monitor for hypercalcemia – rare with moderate daily doses (≤2000 IU) but can occur with Stoss therapy.

Practical Takeaway: If your child has been diagnosed with vitamin D deficiency, do not simply continue the preventive dose (e.g., 400–600 IU daily). Discuss with your pediatrician to obtain a therapeutic regimen tailored to your child's age, severity of deficiency, and presence of any symptoms or rickets.

## Doubao

No, a regular preventive maintenance dose is NOT enough for confirmed vitamin D deficiency. 1. For diagnosed deficiency, a higher therapeutic treatment dose is required first to raise blood 25(OH)D levels back to normal. 2. The standard low daily preventive dose (400 IU / 600 IU) can only maintain normal vitamin D status; it cannot correct a severe deficit quickly. 3. After vitamin D levels return to the normal range via high-dose treatment, the child switches back to the routine preventive daily dose for long-term maintenance to avoid relapse.

# Question 39. Can a one-time large dose of vitamin D be used to correct deficiency quickly?

## ChatGPT

Yes, a one-time large dose of vitamin D—often called a stoss therapy—can be used to correct deficiency quickly in some cases, but it must be done under medical supervision.

- Purpose – Rapidly raises serum 25-hydroxyvitamin D levels, especially in children with rickets or severe deficiency.

- Typical dosing – High-dose oral vitamin D (e.g., 50,000–600,000 IU) given as a single or divided dose over a few days.

- Monitoring – Required to avoid toxicity (hypercalcemia), especially in infants and young children.

- Follow-up – Maintenance supplementation is still needed after the large dose to sustain adequate vitamin D levels.

While effective, this approach is not recommended for routine supplementation and should be reserved for children with confirmed deficiency or when compliance with daily dosing is challenging.

## Gemini

Yes, a one-time large dose of vitamin D can be used to correct a deficiency quickly. In medical terms, this is known as "Stoss therapy" (derived from the German word for "push").

While this method is effective, it is a clinical intervention that must only be performed under strict medical supervision.

1. What is Stoss Therapy?

Stoss therapy involves administering a very high dose of vitamin D—typically ranging from 100,000 IU to 600,000 IU—as a single oral dose or an injection.

2. Why use a one-time dose instead of daily drops?

Doctors may choose this "rapid loading" method for several reasons:

* Urgency: If a child has severe rickets with significant bone pain or is at risk of seizures due to low calcium, the body needs a massive influx of vitamin D immediately to restart the mineralization process.

* Compliance Issues: If a family is unable to administer daily medication consistently for several months, a one-time dose ensures the child receives the necessary treatment.

* Malabsorption: For children with intestinal issues who cannot absorb small daily doses well, a large "bolus" can sometimes bypass those limitations more effectively.

3. Is it safe for children?

Stoss therapy is generally safe and highly effective at healing rickets, but it carries specific risks that daily dosing does not:

* Hypercalcemia: A massive dose can cause blood calcium levels to spike too high, which can lead to vomiting, dehydration, and kidney issues.

* Hypercalciuria: Excess calcium may be excreted in the urine, potentially causing kidney stones.

* Vitamin D Toxicity: Because vitamin D is fat-soluble and stored in the body, an incorrectly calculated "mega-dose" can lead to toxicity.

4. Comparison: Daily vs. One-time Dose

| Feature | Daily Dosing (e.g., 2,000–6,000 IU) | One-time Dose (Stoss Therapy) |

| --- | --- | --- |

| Speed of Recovery | Gradual (weeks to months) | Rapid (days to weeks) |

| Risk of Toxicity | Very Low | Higher (requires careful calculation) |

| Convenience | Requires daily habit | Single event |

| Typical Usage | Mild to moderate deficiency | Severe rickets or compliance concerns |

5. Important Medical Protocol

If a doctor prescribes a one-time large dose, they will usually follow these safety steps:

1. Monitor Calcium: They may check blood calcium levels a few days after the dose to ensure they aren't rising too fast.

2. Hydration: Parents are often told to ensure the child drinks plenty of fluids to protect the kidneys.

3. Maintenance Transition: After the initial "push" corrects the deficiency (usually after 2–3 months), the child must still transition to a standard preventive dose (400–600 IU) to keep the levels from falling again.

Summary

A one-time large dose is a powerful tool to "jump-start" bone healing, but it is not a "DIY" solution. Never attempt to give your child a large dose of vitamin D using over-the-counter supplements without a specific prescription and dosage plan from a pediatrician.

## Qianwen

Yes, a one-time large dose (often called "Stoss therapy" or bolus dosing) is a clinically valid method to correct deficiency quickly, but it is generally reserved for specific situations where daily dosing is not feasible.

While daily dosing is the standard of care, giving a massive single dose can rapidly replenish Vitamin D stores. However, this method carries higher risks and specific clinical requirements.

Preferred Treatment: Daily Oral Dosing

Current clinical guidelines generally prefer daily oral dosing (e.g., 2,000 IU/day for 6–8 weeks) as the first-line treatment for Vitamin D deficiency.

Safety: It mimics the body's natural accumulation process, significantly reducing the risk of toxicity (hypercalcemia).

Efficacy: It effectively raises blood levels without causing the sharp spikes in calcium that large single doses can trigger.

Absorption: It is better tolerated by the gut compared to a massive bolus of fat-soluble vitamins.

The "One-Time Large Dose" (Stoss Therapy)

This approach involves administering a very high dose (e.g., 150,000 to 300,000 IU) in a single shot or over a few days.

Indications: It is typically used only when the patient cannot swallow pills/drops, has severe malabsorption issues (like diarrhea or celiac disease), or has a high likelihood of non-adherence to a daily regimen.

Administration: In many cases, this high dose is administered via intramuscular injection by a healthcare provider to ensure it is absorbed, though high-dose oral regimens exist as well.

Risks and Requirements

Risk of Toxicity: Large single doses can cause a sudden surge in blood calcium levels (hypercalcemia), leading to vomiting, kidney stress, and calcification of soft tissues.

Medical Supervision: This is not a home-treatment strategy. It requires a doctor's prescription and monitoring.

Calcium Supplementation: When using this method, doctors often prescribe calcium supplements alongside the Vitamin D to prevent hypocalcemia (low blood calcium) that can sometimes occur after a large Vitamin D dose mobilizes calcium into the bones too quickly.

Summary: While a one-time large dose works quickly, it is a "sledgehammer" approach compared to the "scalpel" of daily dosing. It should only be used under strict medical guidance when daily oral supplements are impossible to administer.

## DeepSeek

Yes, a one-time large (bolus) dose of vitamin D, often called "Stoss therapy," can be used to correct deficiency. It is generally safe and effective, but it must be administered under strict medical supervision.

Stoss therapy involves giving a single high oral or intramuscular dose of vitamin D (e.g., 150,000 IU to 600,000 IU) to rapidly replenish a child's deficient levels. This approach is considered non-inferior to daily dosing for raising serum 25(OH)D levels in the long term (e.g., over 12 months). The primary advantage is improved adherence and convenience.

Safety in Children: A 2022 meta-analysis of 32 randomized controlled trials (including 8,400 children aged 0–6 years) found that high doses (e.g., 1,200–10,000 IU daily or bolus doses up to 600,000 IU) were not associated with an increased risk of serious adverse events compared to low-dose or placebo groups.

Risks of Non-Medical (Unsupervised) High-Dose Use: While medically supervised therapy is safe, unsupervised high-dose supplementation can be fatal. A 2024 case report describes a 4-year-old who developed life-threatening hypercalcemia after his parents self-administered seven oral vials of 600,000 IU vitamin D each over eight months. He required intensive care, hemodialysis, and bisphosphonate therapy.

Comparison of Dosing Approaches:

| Feature | Supervised Medical Stoss | Unsupervised High Doses |

| :--- | :--- | :--- |

| Dose Range | 150,000–400,000+ IU (single dose) | Variable, often 600,000+ IU |

| Administration | Oral or intramuscular, in clinical setting | Oral, often repeated frequently |

| Safety | Generally well-tolerated | High risk of hypercalcemia, kidney damage, death |

| Monitoring | Baseline and follow-up labs required | None |

| Outcome | Safe, effective correction | Severe toxicity possible |

Summary:

- Can a single high dose be used? Yes, under strict medical supervision (Stoss therapy).

- Is it effective? Yes, it corrects deficiency as well as daily dosing.

- Is it safe? Yes, for the doses used in clinical practice.

- Can parents do this at home? No. Unsupervised high doses have caused life-threatening toxicity.

The most important takeaway is that even though a single large oral dose can be effective, parents must never administer high-dose vitamin D at home. This treatment requires medical evaluation, a prescription for an appropriate dose, and follow-up monitoring to ensure safety.

## Doubao

Yes, a single high-dose vitamin D regimen is clinically acceptable to rapidly correct vitamin D deficiency in children, under pediatric supervision. 1. Purpose A one-time large oral dose quickly boosts serum 25(OH)D levels, avoids daily missed doses, and is especially useful for poor adherence or severe deficiency. 2. Common protocol Pediatricians may prescribe a single dose of 100,000–300,000 IU vitamin D3, tailored to age, weight and deficiency severity. 3. Important rules - Must be given under doctor guidance; do not self-administer high doses. - After the bolus dose, switch back to routine daily preventive supplementation to maintain levels. - Not suitable for infants with certain metabolic diseases, hypercalcemia, or kidney problems. - Proper dosage avoids toxicity; supervised high-dose therapy is safe for most healthy children. 4. Limitation Large single doses fix the deficiency fast, but cannot replace long-term daily maintenance.

# Question 40. Is calcium supplementation needed when treating vitamin D deficiency?

## ChatGPT

Calcium supplementation may be needed in some cases when treating vitamin D deficiency, depending on the child’s dietary intake and serum calcium levels:

1. Adequate dietary calcium – If the child consumes enough calcium through formula, milk, or other foods, additional supplementation is usually not necessary.

2. Low dietary calcium or severe deficiency – Supplementation may be required to ensure proper bone mineralization and effective correction of rickets.

3. Severe rickets – Both vitamin D and calcium may be given together to rapidly restore bone strength and mineralization.

In summary, vitamin D alone can correct mild deficiencies if calcium intake is sufficient, but calcium supplementation is indicated when intake is inadequate or deficiency is severe.

## Gemini

Yes, calcium supplementation is often essential when treating a child for vitamin D deficiency, especially if the deficiency is severe or has progressed to rickets.

Treating with vitamin D alone without ensuring adequate calcium can sometimes be ineffective or even lead to temporary complications. Here is why calcium is a critical partner in treatment:

1. The "Hungry Bone" Syndrome

When a child has been deficient for a long time, their bones are "starved" of minerals.

The Process: Once you provide high-dose vitamin D, the body suddenly gains the ability to deposit calcium into the bones.

The Risk: The bones may absorb calcium from the blood so rapidly that the blood calcium levels drop dangerously low. This can lead to hypocalcemia, which causes muscle cramps, jitters, or even seizures.

The Solution: Providing extra oral calcium ensures there is enough "supply" in the blood to satisfy the bones without crashing the system.

2. Vitamin D Cannot Work Alone

Vitamin D’s primary job is to provide the "transport system" for calcium.

If the child’s diet is low in calcium, even the highest dose of vitamin D won't have any minerals to transport into the skeleton.

To "remineralize" or harden soft bones, the body needs a significant surplus of the raw material: Calcium.

3. How much Calcium is needed?

The amount of extra calcium depends on the child's age and the severity of the deficiency. During the initial 8 to 12 weeks of vitamin D treatment, doctors often recommend:

Dietary Focus: Increasing intake of high-calcium foods (milk, yogurt, cheese, fortified tofu, or dark leafy greens).

Oral Supplements: If the diet is insufficient, a doctor may prescribe 500 mg to 1,000 mg of elemental calcium per day in divided doses.

4. Summary: The Treatment Duo

5. Important Note for Parents

Check the Multivitamin: Many "Vitamin D" drops do not contain calcium. If your child is diagnosed with a deficiency, ask your pediatrician if you should add a separate calcium supplement or simply increase dairy intake.

Avoid Overload: While calcium is necessary, excessive amounts can cause constipation or interfere with the absorption of other minerals like iron. Always follow the specific dosage prescribed by your doctor.

Final Guidance

If vitamin D deficiency is being treated, calcium is the fuel that makes the treatment work. Ensuring your child has enough calcium prevents complications and speeds up the healing of the bones.

## Qianwen

Yes, calcium supplementation is often necessary when treating vitamin D deficiency, particularly during the initial high-dose treatment phase.

While vitamin D acts as the "mover" that facilitates calcium absorption, calcium is the essential "building material" required to repair bones.

Without sufficient calcium, high-dose vitamin D therapy can paradoxically lead to complications.

The Mechanism: Why Calcium is Crucial

Rebound Hypocalcemia: In severe vitamin D deficiency, calcium is often leached from the bones into the blood to maintain basic bodily functions. When high-dose vitamin D treatment begins, it triggers a rapid process of bone remineralization. The bones act like a sponge, aggressively soaking up calcium from the blood.

Risk of Tetany: If dietary calcium is low, this sudden shift can cause blood calcium levels to drop dangerously low (hypocalcemia), potentially leading to seizures or tetany (muscle spasms) .

Decision Criteria: Diet vs. Supplements

Whether you need to add a specific calcium supplement depends on the patient's dietary intake:

Patients Who Likely Need Supplements

Active Rickets: Children diagnosed with active rickets often have depleted calcium stores .

Low Dietary Calcium:

Older Infants/Children: Those who do not consume enough dairy (less than 500ml/day) or calcium-rich foods (tofu, leafy greens).

Vegan Diets: Patients avoiding animal products without fortified alternatives.

Treatment Phase: Specifically during the first few weeks of high-dose vitamin D therapy, doctors often prescribe calcium supplements (e.g., Calcium Carbonate or Citrate) to ensure safety .

Patients Who Likely Do Not Need Supplements

Infants with Adequate Milk Intake:

< 6 Months: Breast milk or standard formula usually provides sufficient calcium (approx. 200–260 mg/day) for infants .

> 6 Months: If the child consumes roughly 500ml (approx. 17 oz) of milk or yogurt daily, this intake is generally sufficient to support vitamin D treatment without extra pills .

Summary

Vitamin D opens the door for absorption.

Calcium is the material that goes through the door.

Actionable Advice: If the patient is undergoing high-dose treatment or has poor dairy intake, calcium supplementation is required. If the patient drinks plenty of milk, food sources are usually sufficient.

## DeepSeek

Yes, calcium supplementation is often needed when treating vitamin D deficiency in children, but not in all cases. The decision depends on the child's age, the severity of the deficiency, whether symptoms are present, and dietary calcium intake.

Summary based on clinical presentation:

| Clinical Scenario | Calcium Supplementation Needed? | Typical Dose | Duration |

| :--- | :--- | :--- | :--- |

| Symptomatic vitamin D deficiency (with hypocalcemia) | Yes – essential for safety | IV calcium (severe) or oral calcium (mild-moderate) | Until hypocalcemia resolves; often 4+ weeks |

| Rickets (with or without hypocalcemia) | Yes – to support bone remineralization | 500–1500 mg/day (age-dependent) | First 2–4 weeks of treatment, sometimes longer |

| Asymptomatic deficiency with adequate dietary calcium | Not routinely needed | Dietary optimization instead | N/A |

| Asymptomatic deficiency with poor dietary calcium intake | Yes – oral calcium recommended | 500–1000 mg/day | Alongside vitamin D treatment course |

Specific Recommendations by Age (Symptomatic Deficiency/Rickets) based on NHS Scotland guidelines:

| Age Group | Vitamin D (daily, 12 weeks) | Calcium (daily, 4 weeks) |

| :--- | :--- | :--- |

| <3 months | 2000 IU | None |

| 3–12 months | 2000–2400 IU | 500 mg |

| 1–12 years | 3000–6400 IU | 500–1000 mg |

| >12 years | 6000–6400 IU | 1000–1500 mg |

Why Calcium Supplementation Is Important: Preventing "Hungry Bone Syndrome." When a child has severe, prolonged vitamin D deficiency, their bones become undermineralized. Once vitamin D therapy begins, the bones suddenly start absorbing large amounts of calcium and phosphorus from the blood to remineralize. If calcium intake is insufficient during this period, profound hypocalcemia (dangerously low blood calcium) can develop, leading to muscle spasms, seizures, or cardiac arrhythmias.

Evidence from Clinical Trials: A 2024 randomized controlled trial involving 80 children (aged 1–10 years) with symptomatic vitamin D deficiency provided strong support for combined therapy. All children received 500 mg of calcium daily alongside vitamin D (either 4000 IU daily or 60,000 IU fortnightly). The results showed significant increases in serum calcium and phosphorus, significant decreases in alkaline phosphatase and PTH, and all children achieved sufficient vitamin D levels by 4–12 weeks.

Practical Summary for Parents:

- Child has bone pain, leg bowing, growth delay, or seizures: Needs medical evaluation; both vitamin D and calcium supplements will likely be prescribed. Do not self-treat.

- Child has low vitamin D on blood test but no symptoms: Ask your pediatrician about checking dietary calcium intake. If child drinks milk/eats dairy regularly, calcium may not be needed.

- Child is being treated for rickets: Calcium is almost always given alongside vitamin D, at least for the first few weeks.

- Child has poor dietary calcium intake (e.g., vegan, milk allergy): Calcium supplementation is often recommended even for asymptomatic deficiency.

Important Note: Do not start calcium supplements without medical advice. Excess calcium can cause constipation, kidney stones, and, in rare cases, hypercalcemia. Your pediatrician will determine the correct dose and duration.

## Doubao

Yes, calcium is usually needed along with vitamin D when treating vitamin D deficiency, especially in children with confirmed insufficiency/deficiency or early rickets. 1. Why calcium is necessary Vitamin D’s main job is to help the gut absorb calcium. If vitamin D is low, calcium absorption drops sharply. Even if you only add vitamin D, the body still lacks usable calcium for bones and growth. 2. Different situations - Mild vitamin D deficiency, no symptoms, normal diet: If the child already gets enough calcium from milk, dairy and daily food, extra calcium supplements may not be required — just vitamin D treatment is enough. - Obvious deficiency, poor milk intake, picky eating, suspected rickets: Must add calcium supplementation during vitamin D treatment to prevent hypocalcemia and bone softening. 3. Key reminder Never give high-dose vitamin D alone without ensuring adequate calcium intake; it may induce secondary hyperparathyroidism and further bone loss. Always follow a pediatrician’s advice for the combined dose of vitamin D and calcium.

# Question 41. Why might a child's vitamin D level remain low after supplementation?

## ChatGPT

A child’s vitamin D level might remain low after supplementation due to several factors:

1. Insufficient dose – The amount of vitamin D given may be too low to correct the deficiency.

2. Poor adherence – Missed doses or irregular administration reduce effectiveness.

3. Malabsorption – Conditions like celiac disease, cystic fibrosis, or inflammatory bowel disease impair absorption of vitamin D from the gut.

4. Chronic medical conditions – Liver or kidney disease can impair conversion of vitamin D to its active forms.

5. Medications – Long-term use of antiepileptics, glucocorticoids, or other drugs may accelerate vitamin D breakdown.

6. Limited sunlight exposure – Low endogenous production can hinder correction, especially in high-risk regions or seasons.

7. Obesity – Vitamin D can be sequestered in fat tissue, reducing bioavailability.

In such cases, dose adjustment, adherence counseling, or evaluation for underlying conditions is needed to effectively normalize vitamin D levels.

## Gemini

If a child’s vitamin D levels do not improve after several months of supplementation, it is usually due to one of four main reasons: administration issues, absorption barriers, metabolic interference, or an underlying genetic condition.

Here is a breakdown of why supplementation might fail:

1. Administration and Compliance Issues

This is the most common reason. Vitamin D must be taken consistently and correctly to build up in the body.

Irregular Dosing: Missing doses frequently prevents the blood concentration from reaching a steady state.

Taking it on an Empty Stomach: Vitamin D is fat-soluble. If it is taken with just water or on an empty stomach, the body may only absorb a fraction of the dose. It should always be taken with a meal containing some fat (e.g., whole milk, yogurt, eggs, or avocado).

Expired or Poor-Quality Supplements: Some over-the-counter supplements may not contain the amount of vitamin D stated on the label, or the product may have degraded over time.

2. Malabsorption Disorders

Even if the child swallows the supplement, their intestines may not be absorbing it into the bloodstream.

Celiac Disease: Gluten-induced inflammation in the gut can prevent the absorption of fat-soluble vitamins (A, D, E, K).

Inflammatory Bowel Disease (IBD): Conditions like Crohn's disease can damage the parts of the small intestine where vitamin D is absorbed.

Cystic Fibrosis or Pancreatic Insufficiency: These conditions lead to a lack of digestive enzymes needed to break down fats, which are required for vitamin D absorption.

3. Increased Metabolism or "Sequestration"

In some children, the vitamin D enters the body but is either used up too quickly or "hidden" away.

Obesity: Vitamin D is sequestered (trapped) in body fat. Because it is fat-soluble, it can get "stuck" in adipose tissue instead of circulating in the blood. Obese children often require 2 to 3 times higher doses than children of a healthy weight.

Medication Interference: Drugs used for epilepsy (e.g., Phenobarbital, Phenytoin) or chronic steroids (e.g., Prednisone) speed up the breakdown of vitamin D in the liver, meaning the child "burns through" their supplement faster than normal.

4. Genetic or Organ Issues

Liver or Kidney Disease: These organs are responsible for converting "raw" vitamin D into its active forms. If they are not functioning correctly, the 25(OH)D level will remain low.

Vitamin D Resistance: In rare genetic cases (such as Vitamin D-Dependent Rickets), the body’s receptors are "blind" to vitamin D, or the body lacks the enzyme to activate it. In these cases, standard doses will have no effect.

Summary Table: What to Check Next

Conclusion

If a child's levels remain low, the next step is typically to review the administration method and then move toward screening for intestinal malabsorption or adjusting the dose based on the child's weight and medication history.

## Qianwen

If a child's vitamin D levels remain low despite supplementation, it is usually due to insufficient dosage, malabsorption issues, or rapid growth outpacing intake.

While most cases are resolved by adjusting the regimen, persistent deficiency can sometimes indicate an underlying medical condition.

Dosage and Compliance Issues

Under-dosing: As discussed earlier, a standard preventive dose (400–800 IU) is often too low to correct an existing deficiency. If the child was given a maintenance dose instead of a therapeutic "loading dose," levels may not rise significantly.

Inconsistent Administration: Missing doses frequently can prevent levels from building up, as the body depletes its stores relatively quickly without consistent intake.

Wrong Formulation: In rare cases, using Vitamin D2 (ergocalciferol) instead of D3 (cholecalciferol) may result in a less efficient rise in blood levels, though D2 is still generally effective if dosed correctly.

Absorption and Metabolic Barriers

Vitamin D is fat-soluble, meaning it requires dietary fat and a healthy digestive system to be absorbed into the blood.

Malabsorption Syndromes: Conditions affecting the gut (e.g., Celiac disease, Cystic Fibrosis, or chronic diarrhea) or liver/bile duct issues (e.g., cholestasis) can prevent the body from absorbing the vitamin, regardless of the dose.

Medication Interference: Certain medications, such as anticonvulsants (e.g., phenobarbital, phenytoin) or long-term steroids, accelerate the breakdown of vitamin D in the liver, rendering standard supplements ineffective.

Magnesium Deficiency: Magnesium is a necessary cofactor for converting vitamin D into its active form. If a child is deficient in magnesium, the vitamin D supplement cannot be properly utilized.

Rapid Growth and "The Sponge Effect"

Infants and toddlers grow at a rapid pace, acting like a "sponge" for nutrients.

High Demand: If a child is growing quickly (e.g., premature infants or those on growth spurts), the vitamin D intake may be immediately consumed by the growing bones, leaving the blood levels appearing low or stable rather than rising.

Recommended Next Steps

Re-evaluate Dosing: Consult a pediatrician to ensure the dosage matches the severity of the deficiency.

Check Testing Accuracy: Ensure the blood test used a reliable method (like mass spectrometry) rather than less sensitive immunoassays, which can sometimes underestimate levels.

Medical Workup: If high doses and strict compliance do not raise levels after 3 months, a doctor should investigate for genetic forms of rickets or malabsorption diseases.

## DeepSeek

If a child's vitamin D level remains low despite supplementation, there are several possible explanations. The most common causes are inadequate dosing, poor adherence, malabsorption, medication interactions, or underlying medical conditions.

Summary of main reasons and solutions:

| Cause | Mechanism | Solution |

| :--- | :--- | :--- |

| Inadequate dose | Dose too low to correct deficiency, or preventive dose used instead of therapeutic dose | Increase to appropriate therapeutic dose (e.g., 2000 IU/day for children >1 year) |

| Poor adherence | Supplement not given consistently or as prescribed | Improve routines; consider directly observed therapy or intermittent high-dose (Stoss) therapy |

| Malabsorption | Vitamin D cannot be absorbed from the gut | Switch to more absorbable formulation (e.g., oral spray, drops with fat), or consider intramuscular (IM) injection |

| Medication interference | Drugs increase vitamin D breakdown | Increase dose (often 2–3× standard) under medical supervision |

| Obesity | Vitamin D sequestered in fat tissue, reducing bioavailability | Use higher doses (often 2–3× standard) based on serum monitoring |

| Chronic diseases | Liver/kidney disease impairs activation; certain conditions increase metabolism | Treat underlying condition; use activated vitamin D (calcitriol) for kidney/liver disease |

| Poor quality or counterfeit supplement | Product contains less vitamin D than labeled | Switch to a reputable brand or pharmacy-grade product |

| Continued deficiency despite correction | 25(OH)D level drawn too soon after starting therapy (e.g., <4 weeks) | Allow 3–4 months of treatment before rechecking |

Detailed Explanations:

1. Inadequate Dosing: This is the most common cause. Treatment requires higher therapeutic doses (typically 1000–2000 IU/day for infants; 2000–6000 IU/day for older children) for 8–12 weeks.

2. Poor Adherence: Daily supplements are often forgotten. If adherence is questionable, consider directly observed therapy or intermittent high-dose therapy (Stoss) under medical supervision.

3. Malabsorption: Conditions such as celiac disease, Crohn's disease, cystic fibrosis, short bowel syndrome, or cholestatic liver disease impair absorption. Options: water-miscible oral vitamin D, sublingual drops, intramuscular injection.

4. Medication Interactions: Anticonvulsants, glucocorticoids, antifungals, antiretrovirals increase vitamin D catabolism. These children often require higher-than-standard doses.

5. Obesity: Vitamin D is sequestered in adipose tissue. Obese children (BMI ≥95th percentile) may require 2–3 times the standard dose.

6. Chronic Liver or Kidney Disease: Liver disease impairs 25-hydroxylation; kidney disease impairs 1α-hydroxylation. Liver disease may require calcifediol; kidney disease requires calcitriol.

7. Poor Quality Supplement: Some over-the-counter products may contain less vitamin D than stated. Use a reputable brand.

8. Laboratory Error or Timing Issues: Drawn too soon (before 8–12 weeks of treatment).

Clinical Approach: If a child's vitamin D level remains low after 3–4 months of adequate supplementation:

1. Verify adherence.

2. Review dose – therapeutic vs. preventive.

3. Check for malabsorption symptoms.

4. Review medication list.

5. Assess for chronic disease.

6. Consider alternative formulations.

7. Recheck 25(OH)D using the same lab if possible.

Practical Takeaway: If your child's vitamin D remains low despite taking supplements as prescribed, do not simply increase the dose on your own. Consult your pediatrician to investigate the underlying cause and adjust the treatment plan accordingly.

## Doubao

Here are the main reasons a child’s vitamin D level stays low even after taking supplements: 1. Inadequate dosage The daily preventive dose is too weak to fix true deficiency; only a therapeutic dose can raise levels effectively. 2. Poor adherence Forgotten doses, irregular intake, or stopping supplementation early. 3. Insufficient sun exposure Living in northern regions, long indoor time, heavy sunscreen, or covering skin all year limits natural skin production of vitamin D. 4. High-risk physical conditions Prematurity, low birth weight, obesity — obese children need much more vitamin D due to fat tissue storing it away. 5. Diet factors Low milk intake, picky eating, insufficient dietary calcium and phosphorus, which affect vitamin D utilization. 6. Malabsorption problems Gastrointestinal issues such as diarrhea, lactose intolerance, gut inflammation reduce vitamin D absorption. 7. Certain medications Long-term use of antiepileptic drugs, glucocorticoids speeds up vitamin D breakdown in the body. 8. Underlying diseases Liver or kidney disease affects the activation of vitamin D in the body.

# Question 42. Can sunlight exposure replace vitamin D supplementation?

## ChatGPT

Sunlight exposure can contribute to vitamin D production, but it usually cannot fully replace supplementation for all children:

1. Mechanism – UVB rays convert skin cholesterol into vitamin D3.

2. Limitations –

- Amount produced depends on latitude, season, time of day, skin color, and clothing coverage.

- Darker-skinned children, those living in northern regions, or those exposed to heavy pollution may produce insufficient vitamin D.

- Infants under 6 months are generally advised to avoid direct sun exposure due to risk of burns.

3. Practical recommendation – Sunlight helps maintain vitamin D, but daily supplementation (e.g., 400 IU for infants) is more reliable, especially for exclusively breastfed infants or at-risk children.

In summary, sunlight is helpful but often not enough alone, so supplementation remains necessary in most cases to prevent deficiency.

## Gemini

While sunlight is the most "natural" source of vitamin D, it is generally not a reliable or recommended replacement for supplementation in infants and children, primarily due to safety concerns and environmental variables.

Here is why relying solely on the sun is problematic for pediatric health:

1. Dermatological Safety (Skin Cancer Risk)

The most significant barrier is the risk of skin damage.

Infants < 6 Months: The American Academy of Pediatrics (AAP) recommends keeping infants out of direct sunlight entirely because their skin is thinner, has less melanin, and is highly susceptible to severe burns and long-term DNA damage that increases skin cancer risk later in life.

Sunscreen Paradox: Sunscreen with an SPF of 15 or higher reduces the skin's ability to synthesize vitamin D by more than 95%. To get enough vitamin D from the sun, a child would need to be exposed without protection, which directly contradicts skin safety guidelines.

2. The "Vitamin D Winter" (Geography and Latitude)

If you live north of approximately the 37th parallel (roughly a line across the US from San Francisco to Richmond, or across Europe/Asia), the sun's angle during the winter months is too low.

The atmosphere filters out the UVB rays necessary for vitamin D production.

In these regions, even if a child spends all day outside in January, they will produce zero vitamin D.

3. Individual Factors Affecting Production

Not all children produce vitamin D at the same rate.

Skin Pigmentation: Melanin acts as a natural filter. Children with darker skin tones may require 3 to 5 times longer in the sun than fair-skinned children to produce the same amount of vitamin D.

Pollution and Clouds: Smog and heavy cloud cover can block up to 60% of UVB radiation.

Clothing: Cultural practices or cold weather that require covering most of the skin prevent synthesis.

4. Comparison: Sunlight vs. Supplementation

5. Summary: The "Prudent" Approach

While "sensible" sun exposure (e.g., 10–15 minutes of sun on the arms and legs a few times a week) can contribute to vitamin D levels, it is rarely enough to meet the total daily requirement of 400–600 IU year-round.

Conclusion: Sunlight should be viewed as a bonus to bone health, but supplementation remains the only safe and reliable way to ensure a child consistently meets their requirements without increasing the risk of skin cancer.

## Qianwen

No, sunlight exposure generally cannot replace vitamin D supplementation, especially when a deficiency has already been diagnosed. While sunlight is the body's natural way of producing vitamin D, it is too unpredictable to serve as a reliable medical treatment for correcting low levels.

Supplements are necessary because they provide a precise, consistent dosage that sunlight cannot guarantee.

Unreliable Synthesis Factors

Unlike a pill where the dosage (e.g., 400 IU or 2,000 IU) is exact, the amount of vitamin D produced by the skin varies wildly based on several factors:

Time and Season: The UVB rays needed to make vitamin D are only strong enough between late morning and early afternoon. In winter or at high latitudes, the sun is often too weak to trigger production .

Barriers: Wearing sunscreen (SPF 15+), clothing, or even sitting behind a window blocks over 90% of the necessary UVB rays, effectively stopping production .

Skin Pigmentation: Darker skin contains more melanin, which acts as a natural sunscreen and significantly slows down vitamin D production .

Safety Risks for Children

Relying on sunlight for vitamin D requires exposing skin to UV radiation.

Skin Damage: Excessive sun exposure, especially in children, increases the risk of sunburn and future skin cancer .

Protective Measures: To prevent heatstroke and sunburn, parents are advised to keep children covered or use sunscreen during outdoor play, which ironically prevents vitamin D synthesis.

Sunlight as Maintenance, Not Treatment

Treatment Phase: If a child is currently deficient, sunlight is insufficient to raise blood levels quickly or safely enough to treat the condition. High-dose supplements are required to "reload" the body's stores.

Maintenance Phase: Once levels are normal, regular outdoor play is excellent for general health. However, it is usually recommended as a complement to supplementation, not a substitute, to ensure the daily requirement is consistently met .

Summary: Think of sunlight as a "bonus" source of vitamin D, but supplements as the "guaranteed" source.

For a child with a diagnosed deficiency, relying on the sun is risky; supplements are the only way to ensure the correct therapeutic dose is received daily.

## DeepSeek

No, sunlight exposure alone cannot reliably replace vitamin D supplementation for children, especially for infants and young children. While sunlight helps the body produce vitamin D, major medical organizations recommend vitamin D supplements because sun exposure is unreliable, potentially harmful, and often insufficient for maintaining adequate levels.

Why Sunlight Is Not a Reliable Replacement

1. Proven Lower Efficacy in Clinical Studies: A 2022 randomized controlled trial directly compared sunlight exposure (30 min/week, 40% body surface) to vitamin D supplementation (400 IU/day) in breastfed infants. Results: Sunlight group: 13.9% achieved sufficient vitamin D levels (no change from baseline); Supplement group: 35.1% achieved sufficiency (significant increase). The study concluded that oral vitamin D3 supplementation is more efficacious than sunlight.

2. Numerous Factors Interfere with Sunlight-Induced Vitamin D Production:

- Sunscreen (SPF 30): Reduces synthesis by up to 95%

- Clothing coverage: Dark or thick clothing blocks most UVB rays

- Glass/windows: Blocks >90% of UVB radiation

- Latitude: Above ~37°N, UVB is insufficient for 4-6 months/year

- Skin pigmentation: Darker skin requires 5-10x longer exposure

- Season & time of day: Winter and early/late hours have minimal UVB

- Air pollution: Particulate matter absorbs/scatters UVB

- Cloud cover: Reduces UVB intensity

3. Sun Protection Is Essential for Children: The American Academy of Pediatrics (AAP) emphasizes that excessive sun exposure causes skin cancer. Approximately 25% of sun exposure occurs before 18 years of age, and sunburns in childhood are strongly associated with melanoma. The AAP recommends that "children's activities that minimize sunlight exposure are preferred."

Official Recommendations from Major Organizations:

- American Academy of Pediatrics (AAP): All infants, children, and adolescents should receive 400-600 IU of supplemental vitamin D daily. "Because levels of sunlight exposure adequate for cutaneous synthesis of vitamin D may increase the risk of skin cancer... infants, children, and adolescents require vitamin D supplementation."

- NICE (UK): Recommends vitamin D supplements for specific population groups including infants and children under 4.

- Indian Pediatrics: After a study in a sun-rich country, researchers concluded that "more robust experimental studies are needed... before we recommend replacing supplemental vitamin D with sunlight exposure during infancy."

The Balanced Approach: Supplement First, Sun Safely

- Prioritize vitamin D supplements: 0-12 months: 400 IU; >1-18 years: 400-600 IU.

- Sunlight as a supplement, not a replacement: Before 10 AM or after 4 PM; 10-30 minutes, 2-3 times per week; arms and legs uncovered; avoid sunburn; infants under 6 months avoid direct sunlight entirely.

What Does NOT Work:

- Sitting by a window (glass blocks UVB)

- Relying on cloudy days (UVB significantly reduced)

- Using sunscreen and expecting vitamin D production (sunscreen blocks synthesis)

Summary: For infants and children, vitamin D supplements are the recommended, reliable, and safe method to prevent deficiency and rickets. Sunlight should be viewed as an additional source—not a replacement—and must always be balanced against skin cancer prevention.

## Doubao

No, sunlight exposure cannot fully replace vitamin D supplementation for children, especially infants and young kids. Reasons 1. Unreliable sun production Season, latitude, cloudy weather, morning/evening time, and skin covering all greatly reduce skin synthesis of vitamin D. 2. Safety risks Young children have delicate skin; too much sun exposure increases sunburn and long-term skin cancer risk. Pediatric guidelines advise avoiding direct strong sunlight and using sunscreen, which blocks vitamin D production. 3. Hard to control the exact dose You cannot accurately calculate how much vitamin D a child makes from sunbathing; it is easy to get insufficient or overexposed. 4. Special groups cannot rely on sunlight Infants, premature babies, children living in high-latitude or smoggy areas, and kids who stay indoors most of the time cannot get enough vitamin D from sun alone. Conclusion Proper moderate sunlight is helpful for vitamin D production, but it cannot take the place of routine daily vitamin D supplementation. Standard oral supplementation is still the safest, most reliable way.

# Question 43. Can too much vitamin D cause toxicity?

## ChatGPT

Yes, too much vitamin D can cause toxicity (hypervitaminosis D), although it is rare and usually results from excessive supplementation, not from sunlight or diet alone.

Key points about vitamin D toxicity:

1. Cause – Chronic intake far above recommended doses, e.g., >10,000 IU/day for several months in children, or very high single doses without medical supervision.

2. Effects – Leads to hypercalcemia, which can cause:

- Nausea, vomiting, poor appetite

- Constipation or diarrhea

- Increased thirst and urination

- Weakness, fatigue

- In severe cases: kidney damage or cardiac arrhythmias

3. Prevention – Follow age-appropriate dosing, avoid unsupervised high-dose supplementation, and monitor blood levels if using therapeutic or high-dose regimens.

In short, moderate, recommended doses are safe, but excessive supplementation carries serious health risks.

## Gemini

Yes, while vitamin D is essential, it is a fat-soluble vitamin, meaning the body stores excess amounts in the liver and fatty tissues rather than excreting it through urine. This can lead to Vitamin D Toxicity, also known as hypervitaminosis D.

Toxicity is a serious medical condition, but it is almost exclusively caused by excessive supplement intake. It is impossible to get vitamin D toxicity from too much sunlight (the skin has a built-in mechanism to stop production) or from a standard diet.

1. The Mechanism: Hypercalcemia

The primary danger of too much vitamin D is that it causes the body to absorb far too much calcium. This leads to hypercalcemia (excessively high blood calcium levels), which acts like a "poison" to various organ systems.

2. Signs and Symptoms of Toxicity

Symptoms often develop slowly as calcium builds up in the bloodstream:

Digestive Issues: Severe nausea, frequent vomiting, poor appetite, and constipation.

Neurological Changes: Unusual fatigue, confusion, irritability, or extreme thirst.

Urinary Changes: Frequent urination (polyuria) as the kidneys struggle to filter the excess calcium.

Kidney Issues: In severe cases, high calcium can lead to kidney stones or calcium deposits in the kidney tissue (nephrocalcinosis), which can cause permanent damage.

3. When Does Toxicity Occur?

Toxicity usually occurs when a child accidentally or intentionally consumes doses far above the "Tolerable Upper Limit" for a prolonged period.

Note: High-dose "Stoss therapy" (a single dose of 100,000+ IU) is an exception because it is a one-time event administered by a doctor, not a daily habit.

4. Common Causes of Accidental Toxicity

Measurement Errors: Using the wrong dropper (e.g., using a 1.0 mL dropper for a concentrated "one-drop" product) can result in a child receiving 40–50 times the intended dose.

Over-fortification: In rare cases, manufacturing errors in milk or formula have led to toxic levels, though this is extremely uncommon due to strict regulations.

"More is Better" Mentality: Parents giving multiple supplements (e.g., a multivitamin + a dedicated D3 drop + a cod liver oil supplement) without realizing they are stacking the doses.

5. Treatment for Toxicity

If toxicity is diagnosed (usually confirmed by a 25(OH)D level > 100–150 ng/mL and high blood calcium):

Stop all Vitamin D and Calcium: Immediately halt all supplements.

Fluid Therapy: Intravenous fluids may be needed to flush calcium from the kidneys.

Low-Calcium Diet: Temporarily restricting dairy and other calcium-rich foods.

Medication: In severe cases, drugs like steroids or bisphosphonates may be used to block bone resorption and lower blood calcium.

Summary

Vitamin D toxicity is preventable. To keep your child safe, always use the dropper provided with the specific supplement, store supplements out of reach of children (especially chewable "gummies" that taste like candy), and never exceed the dosage recommended by your pediatrician.

## Qianwen

Yes, excessive vitamin D intake can indeed cause toxicity, though it is relatively rare and typically results from misuse of supplements rather than diet or sunlight.

While vitamin D is essential, it is fat-soluble and accumulates in the body. Unlike water-soluble vitamins (like Vitamin C) that are easily excreted, too much Vitamin D leads to a dangerous buildup of calcium in the blood (hypercalcemia).

The Mechanism: Toxicity via Hypercalcemia

Vitamin D increases calcium absorption. When levels become toxic, calcium levels in the blood rise to dangerous heights, causing the body to malfunction:

Kidney Stress: The kidneys try to filter out the excess calcium, which can lead to kidney stones or even chronic kidney failure (calcification of kidney tissue).

Cardiovascular Issues: High calcium can interfere with heart rhythm and cause blood pressure changes.

Neurological Effects: It can affect the central nervous system, leading to confusion, lethargy, and in severe cases, coma or seizures.

Signs of Vitamin D Toxicity

Symptoms often develop gradually and can be mistaken for other illnesses.

Early Signs: Loss of appetite, nausea, vomiting, constipation, weakness, and fatigue.

Advanced Signs: Excessive thirst (polydipsia), frequent urination (polyuria), kidney pain, and heart irregularities.

Dosing Thresholds and Safety

Therapeutic vs. Toxic: The doses used for treatment (e.g., 2,000 IU to 50,000 IU for a short period) are generally safe when monitored.

Toxic Doses: Toxicity is usually associated with long-term intake exceeding 10,000 IU/day for adults or significantly lower thresholds for infants (e.g., >1,000–2,000 IU/day long-term depending on weight) without medical supervision.

Accidental Overdose: This often happens when parents confuse "monthly" or "weekly" high-dose capsules (like 60,000 IU) with daily supplements, or mix multiple supplements containing Vitamin D.

Actionable Advice

Follow Guidelines: Do not assume "more is better." Stick to the dosage prescribed by a pediatrician.

Monitor Symptoms: If a child exhibits unexplained vomiting, extreme thirst, or lethargy while on high doses, stop the supplement and contact a doctor immediately.

Regular Testing: When on high-dose therapy, periodic blood tests (calcium and Vitamin D levels) are the best defense against toxicity.

## DeepSeek

Yes, too much vitamin D can cause toxicity, a condition known as hypervitaminosis D. This is a serious medical issue that occurs when a child takes excessively high doses of vitamin D supplements, not from diet or sun exposure.

While vitamin D toxicity is rare, recent reports indicate that cases are rising, particularly among infants and adolescents. The primary cause is the widespread availability of high-dose, over-the-counter supplements and manufacturing errors.

How Much is Too Much? Toxicity does not result from food or moderate sunshine but from ingesting manufacturing error-level or megadose quantities of supplements.

| Age Group | Recommended Daily Intake | Tolerable Upper Intake Level (UL) |

| :--- | :--- | :--- |

| Infants (0-6 months) | 400 IU | 1,000 IU |

| Infants (6-12 months) | 400 IU | 1,500 IU |

| Children (1-3 years) | 600 IU | 2,500 IU |

| Children (4-8 years) | 600 IU | 3,000 IU |

| Adolescents (9-18 years) | 600 IU | 4,000 IU |

The cases of toxicity reported in medical literature involve children who ingested doses far exceeding these upper limits—often by tens or even hundreds of times the recommended amount. For example, reports describe:

- An infant who received 25 times the recommended amount of vitamin D daily for several months.

- An adolescent who consumed approximately 14,000 IU daily for a year.

- A young child given 7 oral vials, each containing 600,000 IU, over 8 months.

Recognizing the Signs: Symptoms of Toxicity. Because vitamin D causes the body to absorb more calcium from food, the primary effects of toxicity are due to hypercalcemia (dangerously high levels of calcium in the blood). The symptoms can be vague and develop over time, but they are serious.

Gastrointestinal and general symptoms often appear first:

- Poor appetite, weight loss, nausea, and vomiting

- Abdominal pain and constipation

- Lethargy, fatigue, and weakness

In more severe cases, the excess calcium can cause:

- Polyuria (excessive urination) and polydipsia (excessive thirst)

- Dehydration

- Kidney stones or nephrocalcinosis (calcium deposits in the kidneys)

- Hypertension

- Cardiac arrhythmias

- Altered mental status

Treatment of Toxicity: If toxicity is suspected, immediate medical attention is required. Treatment may include:

- Stopping all vitamin D and calcium supplements

- Intravenous fluids and diuretics to increase calcium excretion

- Corticosteroids (e.g., prednisone) to reduce calcium absorption

- Bisphosphonates (e.g., pamidronate) in severe cases

- Hemodialysis in life-threatening cases

Prevention: Parents should always:

- Use only the recommended dose for their child's age (400-600 IU/day)

- Keep all supplements, especially high-dose ones, out of reach of children

- Never give a child an adult supplement or a "mega-dose" without a doctor's prescription and supervision

- Consult a pediatrician before starting any supplement beyond standard multivitamins

Important Distinction: Vitamin D toxicity is caused by excessive SUPPLEMENT intake, not by sun exposure or diet. The body regulates vitamin D production from sunlight, and food sources naturally contain very low amounts.

## Doubao

Yes, vitamin D toxicity is possible if taken in excessive high doses for a long time. Key points 1. Vitamin D is fat-soluble, so excess is stored in fat and the liver; it cannot be excreted quickly like water-soluble vitamins. 2. Toxicity threshold Blood 25(OH)D over 150 ng/mL raises toxicity risk. Daily intake far above 1000–2000 IU long-term in young children, or unsupervised mega doses, can cause overload. 3. Main symptoms of vitamin D toxicity - Nausea, vomiting, poor appetite, constipation - Excessive thirst, frequent urination - High blood calcium (hypercalcemia) - In severe cases: calcium deposits in kidneys, kidney stones, kidney damage. 4. Safe note Routine standard doses (400–600 IU/day) will never cause toxicity. Toxicity almost always comes from self-giving extra high doses or repeated large bolus doses without pediatric advice.

# Question 44. If a child has seizures, stridor, or carpopedal spasms, could this be related to vitamin D deficiency or hypocalcemia, and what should parents do?

## ChatGPT

Yes, seizures, stridor (noisy breathing), or carpopedal spasms (hand/foot muscle cramps) in a child can be related to severe vitamin D deficiency causing hypocalcemia.

- Mechanism – Vitamin D deficiency reduces calcium absorption, leading to low blood calcium (hypocalcemia), which can trigger neuromuscular symptoms and, in extreme cases, seizures.

- Urgency – These are medical emergencies and require immediate evaluation.

What parents should do:

1. Seek emergency medical care immediately – Go to the nearest hospital or call emergency services.

2. Do not try to manage at home – Oral supplements alone are insufficient in acute hypocalcemia.

3. Follow-up care – The child will likely need intravenous calcium, vitamin D therapy, and monitoring of blood calcium and vitamin D levels.

These symptoms indicate severe deficiency and metabolic complications, not routine supplementation needs, and require prompt professional intervention.

## Gemini

Yes, these symptoms are medical emergencies and are classic signs of severe hypocalcemia (critically low blood calcium), which is frequently a direct result of profound vitamin D deficiency.

When vitamin D is extremely low, the body cannot absorb enough calcium to keep the nerves and muscles functioning correctly. This causes the nervous system to become "irritable" and fire signals uncontrollably.

1. Recognizing the Symptoms

Seizures: Hypocalcemic seizures are often the first sign of rickets in infants. They occur because the brain's electrical activity becomes unstable without enough calcium.

Stridor: This is a high-pitched, "crowing" sound when the child breathes in. It is caused by laryngospasm (spasms of the vocal cords). This is life-threatening as it can block the airway.

Carpopedal Spasms: This is a specific type of cramp where the hands or feet contract involuntarily. The fingers may straighten and the thumb may tuck into the palm (often called "obstetrician's hand").

2. The Link to Vitamin D

This condition is most common in:

Exclusively breastfed infants who have received no vitamin D supplements.

Adolescents during rapid growth spurts who have severe deficiency.

In these cases, the "bone bank" of calcium is empty, and the blood calcium levels have finally "crashed."

3. What Should Parents Do?

This is an EMERGENCY. If your child exhibits any of these signs, you must seek immediate medical attention at an Emergency Room.

Do Not Attempt Home Treatment: Do not try to give oral vitamin D drops or milk at home to fix a seizure or breathing issue. The child needs intravenous (IV) calcium to stabilize their heart and nerves safely.

Call Emergency Services (911): If the child is having trouble breathing (stridor) or is having a prolonged seizure, call for an ambulance immediately.

Hospital Protocol: In the ER, doctors will likely:

Administer IV Calcium Gluconate to stop the spasms or seizures.

Check blood levels for Calcium, Phosphorus, Magnesium, and 25(OH)D.

Begin high-dose Vitamin D therapy once the acute crisis is over.

4. Summary

While vitamin D deficiency is often seen as a "silent" or "slow" bone issue, hypocalcemic crisis is its most dangerous acute complication. Seizures or breathing noises in an infant who has not been receiving vitamin D supplements should always be treated as potential hypocalcemia until proven otherwise.

Final Guidance

If your child has experienced any "shaking" spells, strange breathing sounds, or muscle stiffness, do not wait for a routine pediatrician appointment. Go to the nearest emergency department for a metabolic workup.

## Qianwen

Yes, these are classic and potentially life-threatening signs of severe hypocalcemia (low blood calcium), often caused by Vitamin D deficiency.

This condition is medically known as Vitamin D Deficiency Tetany. It occurs when the blood calcium level drops so low (typically below 1.75–1.88 mmol/L) that it makes nerves and muscles hyper-excitable, causing them to contract uncontrollably .

This is a medical emergency that requires immediate intervention.

Understanding the Symptoms

These symptoms indicate that the body has lost its ability to regulate muscle relaxation:

Seizures (Convulsions): Sudden stiffening or jerking of the limbs, often with eyes rolling back. In this condition, seizures usually happen without a fever (afebrile) .

Carpopedal Spasms: Painful, involuntary cramping of the hands and feet. The hands may form a "piano-playing" or "obstetrician's" fist (thumbs pulled into the palm), and feet may arch stiffly .

Stridor (Laryngospasm): This is the most dangerous symptom. The vocal cords spasm and close off the airway, causing a high-pitched, difficult breathing sound. It can lead to suffocation and death if not treated immediately .

Immediate Action Plan for Parents

If a child exhibits any of these symptoms, follow these steps immediately:

Call Emergency Services (911/120): Do not wait to see if it passes. Stridor indicates the airway is compromised.

Secure the Airway (Critical for Stridor):

Keep the child calm; crying increases oxygen demand and worsens spasms.

If the child is choking or turning blue, lay them on their side. If necessary, pull the tongue gently forward to prevent it from blocking the throat while waiting for oxygen .

Do NOT Force Medication: Do not try to feed the child calcium supplements or water orally right now. They may choke or inhale it into their lungs due to the spasms.

Hospital Treatment: Doctors will administer calcium directly into the vein (IV calcium gluconate) to rapidly raise blood calcium levels and stop the spasms . They may also use sedatives to control the seizures.

Important Connection to Vitamin D Treatment

The "Rebound" Risk: As mentioned in our earlier discussion, if a child with severe Vitamin D deficiency is started on high-dose Vitamin D without enough calcium, the bones may rapidly absorb calcium from the blood (remineralization). This sudden drop in blood calcium can actually trigger these seizures during the first few days of treatment .

## DeepSeek

Yes, seizures, stridor (a high-pitched breathing sound), and carpopedal spasms (painful hand/foot cramps) can be related to severe vitamin D deficiency, specifically due to the resulting hypocalcemia (dangerously low blood calcium levels). This is a medical emergency.

These symptoms indicate symptomatic hypocalcemia, a serious complication of advanced vitamin D deficiency (or rickets) where the body cannot maintain normal calcium levels in the blood. Neuromuscular irritability increases, leading to these hallmark signs.

The Connection: Vitamin D Deficiency → Hypocalcemia → Symptoms

| Symptom | Description | Mechanism |

| :--- | :--- | :--- |

| Seizures | Generalized tonic-clonic seizures (whole-body shaking) or focal seizures | Low calcium increases neuronal excitability, causing uncontrolled electrical discharges in the brain |

| Carpopedal spasms | Painful, involuntary cramping of hands (fingers adduct, thumb flexes, wrist bends) and feet (toes curl, foot arches) | Low calcium increases peripheral nerve excitability, causing sustained muscle contraction |

| Stridor | High-pitched, noisy breathing (audible inspiratory sound) | Laryngospasm – spasm of the vocal cords due to low calcium-induced neuromuscular irritability |

Other signs of hypocalcemia that may accompany these include:

- Tetany – Generalized muscle twitching or cramping

- Paresthesias – Numbness or tingling around the mouth, fingers, or toes

- Chvostek's sign – Twitching of facial muscles when tapping the cheek (over the facial nerve)

- Trousseau's sign – Carpal spasm induced by inflating a blood pressure cuff on the arm

What Parents Should Do Immediately

Step 1: Recognize the Emergency. If your child has a seizure, difficulty breathing (stridor), or severe muscle spasms that do not resolve quickly:

- Call emergency services (911 or local emergency number) immediately – Do not wait.

- Do not try to treat the child at home – Seizures and stridor can be life-threatening.

Step 2: While Waiting for Help

- Seizure: Lay the child on their side (recovery position) away from hard/sharp objects. Do not put anything in their mouth. Time the seizure.

- Stridor or carpopedal spasms: Keep the child calm and seated upright. Do not give food or drink.

- Do not give oral calcium or vitamin D supplements – The child may choke or aspirate if seizing or having laryngospasm.

Step 3: Immediate Medical Evaluation. Once at the hospital, doctors will:

- Check serum calcium (ionized or total corrected for albumin) – Hypocalcemia is defined as total calcium <8.5 mg/dL (2.1 mmol/L) in older children, or lower in infants; severe cases may be <6 mg/dL (<1.5 mmol/L).

- Check serum magnesium, phosphorus, PTH, and 25(OH)D – To identify the underlying cause.

- Administer intravenous calcium gluconate or calcium chloride – This rapidly corrects hypocalcemia and stops seizures/spasms.

- Start vitamin D therapy – Once acute hypocalcemia is stabilized.

Differential Diagnosis: Not Always Vitamin D Deficiency. While vitamin D deficiency is a common cause of pediatric hypocalcemia, other conditions can present with seizures, stridor, or carpopedal spasms:

- Hypoparathyroidism (low PTH, high phosphorus)

- Pseudohypoparathyroidism (high PTH, high phosphorus, characteristic physical features)

- Seizure disorder (epilepsy) with normal calcium

- Severe respiratory infection (croup) – stridor without tetany

- Anxiety or hyperventilation – carpopedal spasms with normal calcium (respiratory alkalosis)

Long-Term Management After Emergency Stabilization. Once the child is stable, the underlying vitamin D deficiency must be treated to prevent recurrence:

- Vitamin D (for rickets/deficiency): High-dose therapy, e.g., 2000–6000 IU daily (age-dependent) for 8–12 weeks, or Stoss therapy under medical supervision.

- Calcium: Oral calcium supplementation (500–1500 mg/day, age-dependent) for the first 2–4 weeks to prevent "hungry bone syndrome" during remineralization.

- Monitoring: Repeat serum calcium, phosphorus, ALP, PTH, and 25(OH)D at 1 week, 4 weeks, and 3 months.

- Address underlying cause: If malabsorption (celiac, CF), medications (anticonvulsants), or chronic disease is present, treat accordingly.

Prevention: The Best Medicine. Parents can prevent this emergency entirely by ensuring their child receives adequate vitamin D:

- All infants (0–12 months): 400 IU vitamin D daily, starting soon after birth.

- Children >1 year: 400–600 IU vitamin D daily.

- High-risk children (dark skin, limited sun exposure, malabsorption, chronic medications, obesity): Higher doses (800–2000 IU daily) or routine monitoring.

If your child has never received vitamin D supplementation and has risk factors, talk to your pediatrician about checking a 25(OH)D level before symptoms develop.

Summary:

- Can seizures/stridor/carpopedal spasms be due to vitamin D deficiency? Yes – via severe hypocalcemia from advanced deficiency or rickets.

- What should parents do? Call emergency services immediately. Do not treat at home.

- What will the hospital do? Give IV calcium, check labs, then start vitamin D and oral calcium.

- Can this be prevented? Yes – Routine vitamin D supplementation from infancy through adolescence.

Bottom line: These symptoms in a child are never normal. If you witness a seizure, stridor, or carpopedal spasms, seek emergency care immediately. Prompt treatment of hypocalcemia is life-saving, and identifying vitamin D deficiency as the cause allows for definitive treatment to prevent future episodes.

## Doubao

Yes, these symptoms are classic warning signs of severe vitamin D deficiency leading to hypocalcemia (low blood calcium): - Seizures - Stridor (laryngospasm) - Carpopedal spasms (hand/foot muscle cramping and contraction) Why it happens Severe vitamin D deficiency reduces calcium absorption → blood calcium drops → increased neuromuscular excitability → spasms, laryngospasm, and even convulsions. What parents must do immediately 1. Treat this as an emergency Do not wait or try home supplements. Take the child to the emergency department right away. 2. Tell the doctor clearly Mention suspected vitamin D deficiency, poor supplementation, low milk intake, or little sun exposure. 3. Hospital management Doctors will check blood calcium, phosphorus, alkaline phosphatase, PTH, and 25(OH)D. Emergency treatment includes IV calcium to quickly raise blood calcium, followed by high-dose vitamin D and oral calcium for recovery. 4. Never give high-dose vitamin D by yourself In acute hypocalcemia, high vitamin D alone can worsen the condition without proper calcium correction. Bottom line Seizures, stridor, or carpopedal spasms in a child are medical emergencies until hypocalcemia and vitamin D deficiency are ruled out. Seek urgent pediatric care immediately.
