# Supplementary material for: Cross-sectional comparative evaluation of five large language model–driven chatbots on an expert-curated 44-question set of parent-facing questions about pediatric vitamin D deficiency
Source: Front Pediatr. 2026 Sep 9;14:1927624. doi: 10.3389/fped.2026.1927624 (PMC13597792; doi:10.3389/fped.2026.1927624)
Supplement: Supplementary file 4 [file Supplementaryfile4.docx]

## Table S1. Inter-rater agreement

| **Metric** | **Agreement index** | **Estimate** | **95% CI** | ***P* value** | **Complete items** | **No. of raters** | **Note** |
| --- | --- | --- | --- | --- | --- | --- | --- |
| Safety | Fleiss' kappa | 0.842 | 0.746-0.913 | <0.001 | 220 | 5 | Primary agreement index for Safety; 95% CI based on 3000 nonparametric bootstrap resamples. |
| Accuracy | ICC(2,1) | 0.901 | 0.880-0.919 | <0.001 | 220 | 5 | Two-way random-effects, absolute-agreement, single-measure ICC. |
| Empathy | ICC(2,1) | 0.876 | 0.851-0.898 | <0.001 | 220 | 5 | Two-way random-effects, absolute-agreement, single-measure ICC. |
| DISCERN | ICC(2,1) | 0.893 | 0.871-0.912 | <0.001 | 220 | 5 | Two-way random-effects, absolute-agreement, single-measure ICC. |
| EQIP | ICC(2,1) | 0.846 | 0.816-0.873 | <0.001 | 220 | 5 | Two-way random-effects, absolute-agreement, single-measure ICC. |
| JAMA | ICC(2,1) | 0.899 | 0.879-0.918 | <0.001 | 220 | 5 | Two-way random-effects, absolute-agreement, single-measure ICC. |
| GQS | ICC(2,1) | 0.914 | 0.897-0.930 | <0.001 | 220 | 5 | Two-way random-effects, absolute-agreement, single-measure ICC. |

Note: Safety was assessed using Fleiss' kappa. Accuracy, Empathy, DISCERN, EQIP, JAMA and GQS were assessed using ICC(2,1).

## Table S2. Pairwise comparisons of safety

| **Comparison** | **N pairs** | **Safe rate difference, %** | **Safe to unsafe, n** | **Unsafe to safe, n** | **Adjusted *P*** | **Note** |
| --- | --- | --- | --- | --- | --- | --- |
| ChatGPT vs Gemini | 44 | 2.27 | 2 | 1 | 1.000 | Pairwise McNemar test |
| ChatGPT vs Qianwen | 44 | 4.55 | 4 | 2 | 1.000 | Pairwise McNemar test |
| ChatGPT vs DeepSeek | 44 | 6.82 | 5 | 2 | 1.000 | Pairwise McNemar test |
| ChatGPT vs Doubao | 44 | 9.09 | 6 | 2 | 1.000 | Pairwise McNemar test |
| Gemini vs Qianwen | 44 | 2.27 | 2 | 1 | 1.000 | Pairwise McNemar test |
| Gemini vs DeepSeek | 44 | 4.55 | 4 | 2 | 1.000 | Pairwise McNemar test |
| Gemini vs Doubao | 44 | 6.82 | 6 | 3 | 1.000 | Pairwise McNemar test |
| Qianwen vs DeepSeek | 44 | 2.27 | 2 | 1 | 1.000 | Pairwise McNemar test |
| Qianwen vs Doubao | 44 | 4.55 | 4 | 2 | 1.000 | Pairwise McNemar test |
| DeepSeek vs Doubao | 44 | 2.27 | 2 | 1 | 1.000 | Pairwise McNemar test |

Note: Pairwise comparisons were performed using McNemar tests with Benjamini-Hochberg correction.

## Table S3. Significant pairwise comparisons for non-binary outcomes

| **Metric** | **Comparison** | **N pairs** | **Mean difference** | **Median difference** | **Adjusted *P*** |
| --- | --- | --- | --- | --- | --- |
| Accuracy | ChatGPT vs Gemini | 44 | 0.45 | 0.20 | <0.001 |
| Accuracy | ChatGPT vs DeepSeek | 44 | 0.32 | 0.10 | 0.004 |
| Accuracy | Gemini vs Qianwen | 44 | -0.38 | -0.20 | 0.003 |
| Accuracy | Gemini vs Doubao | 44 | -0.39 | -0.20 | 0.001 |
| Accuracy | Qianwen vs DeepSeek | 44 | 0.25 | 0.00 | 0.025 |
| Accuracy | DeepSeek vs Doubao | 44 | -0.26 | 0.00 | 0.025 |
| Empathy | ChatGPT vs DeepSeek | 44 | -0.22 | 0.00 | 0.029 |
| Empathy | Gemini vs DeepSeek | 44 | -0.25 | 0.00 | 0.029 |
| Empathy | Qianwen vs DeepSeek | 44 | -0.39 | 0.00 | 0.011 |
| Empathy | Qianwen vs Doubao | 44 | -0.36 | 0.00 | 0.011 |
| DISCERN | ChatGPT vs Gemini | 44 | 5.20 | 6.10 | <0.001 |
| DISCERN | ChatGPT vs DeepSeek | 44 | 4.07 | 3.80 | <0.001 |
| DISCERN | Gemini vs Qianwen | 44 | -4.48 | -5.10 | <0.001 |
| DISCERN | Gemini vs Doubao | 44 | -5.29 | -5.00 | <0.001 |
| DISCERN | Qianwen vs DeepSeek | 44 | 3.36 | 2.70 | 0.004 |
| DISCERN | DeepSeek vs Doubao | 44 | -4.17 | -4.20 | <0.001 |
| EQIP | ChatGPT vs Gemini | 44 | 6.50 | 7.50 | <0.001 |
| EQIP | Gemini vs Qianwen | 44 | -5.11 | -5.00 | 0.001 |
| EQIP | Gemini vs DeepSeek | 44 | -3.89 | -5.00 | 0.016 |
| EQIP | Gemini vs Doubao | 44 | -7.36 | -7.50 | <0.001 |
| EQIP | Qianwen vs Doubao | 44 | -2.25 | -1.50 | 0.034 |
| EQIP | DeepSeek vs Doubao | 44 | -3.48 | -3.00 | 0.006 |
| JAMA | Gemini vs Qianwen | 44 | -0.55 | -0.80 | 0.019 |
| GQS | ChatGPT vs Gemini | 44 | 0.42 | 0.00 | <0.001 |
| GQS | Gemini vs Qianwen | 44 | -0.24 | 0.00 | 0.022 |
| GQS | Gemini vs DeepSeek | 44 | -0.30 | 0.00 | 0.004 |
| GQS | Gemini vs Doubao | 44 | -0.56 | -0.90 | <0.001 |
| GQS | Qianwen vs Doubao | 44 | -0.33 | 0.00 | 0.022 |
| GQS | DeepSeek vs Doubao | 44 | -0.26 | 0.00 | 0.033 |
| ARI | ChatGPT vs Gemini | 44 | -1.39 | -1.14 | <0.001 |
| ARI | ChatGPT vs Qianwen | 44 | -2.07 | -2.06 | <0.001 |
| ARI | ChatGPT vs Doubao | 44 | -5.60 | -5.51 | <0.001 |
| ARI | Gemini vs Qianwen | 44 | -0.67 | -0.74 | 0.019 |
| ARI | Gemini vs DeepSeek | 44 | 0.83 | 0.68 | 0.014 |
| ARI | Gemini vs Doubao | 44 | -4.21 | -3.85 | <0.001 |
| ARI | Qianwen vs DeepSeek | 44 | 1.50 | 1.55 | <0.001 |
| ARI | Qianwen vs Doubao | 44 | -3.54 | -2.92 | <0.001 |
| ARI | DeepSeek vs Doubao | 44 | -5.04 | -5.09 | <0.001 |
| CL | ChatGPT vs Gemini | 44 | 0.85 | 0.76 | 0.006 |
| CL | ChatGPT vs Qianwen | 44 | -2.46 | -2.34 | <0.001 |
| CL | ChatGPT vs DeepSeek | 44 | -0.89 | -0.26 | 0.047 |
| CL | ChatGPT vs Doubao | 44 | -1.36 | -1.32 | <0.001 |
| CL | Gemini vs Qianwen | 44 | -3.31 | -3.56 | <0.001 |
| CL | Gemini vs DeepSeek | 44 | -1.74 | -1.02 | <0.001 |
| CL | Gemini vs Doubao | 44 | -2.21 | -2.10 | <0.001 |
| CL | Qianwen vs DeepSeek | 44 | 1.57 | 1.87 | <0.001 |
| CL | Qianwen vs Doubao | 44 | 1.10 | 1.50 | <0.001 |
| FKGL | ChatGPT vs Gemini | 44 | -1.14 | -1.25 | <0.001 |
| FKGL | ChatGPT vs Qianwen | 44 | -2.12 | -2.02 | <0.001 |
| FKGL | ChatGPT vs Doubao | 44 | -4.47 | -4.38 | <0.001 |
| FKGL | Gemini vs Qianwen | 44 | -0.98 | -0.93 | <0.001 |
| FKGL | Gemini vs DeepSeek | 44 | 0.75 | 0.72 | 0.013 |
| FKGL | Gemini vs Doubao | 44 | -3.33 | -2.77 | <0.001 |
| FKGL | Qianwen vs DeepSeek | 44 | 1.73 | 1.83 | <0.001 |
| FKGL | Qianwen vs Doubao | 44 | -2.35 | -2.12 | <0.001 |
| FKGL | DeepSeek vs Doubao | 44 | -4.07 | -4.13 | <0.001 |
| GFI | ChatGPT vs Gemini | 44 | -1.26 | -1.32 | <0.001 |
| GFI | ChatGPT vs Qianwen | 44 | -2.62 | -2.38 | <0.001 |
| GFI | ChatGPT vs Doubao | 44 | -4.72 | -4.51 | <0.001 |
| GFI | Gemini vs Qianwen | 44 | -1.37 | -1.39 | <0.001 |
| GFI | Gemini vs Doubao | 44 | -3.47 | -2.74 | <0.001 |
| GFI | Qianwen vs DeepSeek | 44 | 1.99 | 1.90 | <0.001 |
| GFI | Qianwen vs Doubao | 44 | -2.10 | -1.97 | <0.001 |
| GFI | DeepSeek vs Doubao | 44 | -4.09 | -4.08 | <0.001 |
| SMOG | ChatGPT vs Gemini | 44 | -1.09 | -1.17 | <0.001 |
| SMOG | ChatGPT vs Qianwen | 44 | -1.38 | -1.19 | <0.001 |
| SMOG | ChatGPT vs Doubao | 44 | -3.40 | -3.34 | <0.001 |
| SMOG | Gemini vs DeepSeek | 44 | 0.84 | 0.80 | <0.001 |
| SMOG | Gemini vs Doubao | 44 | -2.31 | -1.82 | <0.001 |
| SMOG | Qianwen vs DeepSeek | 44 | 1.13 | 1.16 | <0.001 |
| SMOG | Qianwen vs Doubao | 44 | -2.02 | -1.90 | <0.001 |
| SMOG | DeepSeek vs Doubao | 44 | -3.15 | -3.48 | <0.001 |
| FRES | ChatGPT vs Qianwen | 44 | 15.20 | 13.88 | <0.001 |
| FRES | ChatGPT vs Doubao | 44 | 12.49 | 11.38 | <0.001 |
| FRES | Gemini vs Qianwen | 44 | 16.30 | 16.34 | <0.001 |
| FRES | Gemini vs Doubao | 44 | 13.60 | 13.88 | <0.001 |
| FRES | Qianwen vs DeepSeek | 44 | -11.94 | -14.21 | <0.001 |
| FRES | DeepSeek vs Doubao | 44 | 9.23 | 10.29 | <0.001 |

Note: Pairwise comparisons were performed using paired Wilcoxon signed-rank tests with Benjamini-Hochberg correction. Only significant comparisons are shown.

## **Table S4. All pairwise comparisons for non-binary outcomes**

| **Metric** | **Comparison** | **N pairs** | **Mean difference** | **Mean difference 95% CI** | **Median difference** | **Median difference 95% CI** | **Rank-biserial correlation** | **Rank-biserial 95% CI** | **Adjusted *P*** | **Note** |
| --- | --- | --- | --- | --- | --- | --- | --- | --- | --- | --- |
| Accuracy | ChatGPT vs Gemini | 44 | 0.45 | 0.29-0.61 | 0.20 | 0.00-1.00 | 0.858 | 0.636-1.000 | <0.001 | Paired Wilcoxon signed-rank test |
| Accuracy | ChatGPT vs Qianwen | 44 | 0.08 | -0.10-0.27 | 0.00 | 0.00-0.00 | 0.181 | -0.307-0.626 | 0.499 | Paired Wilcoxon signed-rank test |
| Accuracy | ChatGPT vs DeepSeek | 44 | 0.32 | 0.14-0.51 | 0.10 | 0.00-0.50 | 0.698 | 0.373-0.949 | 0.004 | Paired Wilcoxon signed-rank test |
| Accuracy | ChatGPT vs Doubao | 44 | 0.06 | -0.09-0.21 | 0.00 | 0.00-0.00 | 0.237 | -0.248-0.684 | 0.414 | Paired Wilcoxon signed-rank test |
| Accuracy | Gemini vs Qianwen | 44 | -0.38 | -0.58--0.16 | -0.20 | -1.00-0.00 | -0.673 | -0.961--0.333 | 0.003 | Paired Wilcoxon signed-rank test |
| Accuracy | Gemini vs DeepSeek | 44 | -0.13 | -0.30-0.06 | 0.00 | 0.00-0.00 | -0.433 | -0.843-0.076 | 0.127 | Paired Wilcoxon signed-rank test |
| Accuracy | Gemini vs Doubao | 44 | -0.39 | -0.55--0.23 | -0.20 | -0.80-0.00 | -0.779 | -0.972--0.486 | 0.001 | Paired Wilcoxon signed-rank test |
| Accuracy | Qianwen vs DeepSeek | 44 | 0.25 | 0.03-0.45 | 0.00 | 0.00-0.20 | 0.593 | 0.156-0.937 | 0.025 | Paired Wilcoxon signed-rank test |
| Accuracy | Qianwen vs Doubao | 44 | -0.01 | -0.20-0.18 | 0.00 | 0.00-0.00 | 0.027 | -0.411-0.496 | 0.919 | Paired Wilcoxon signed-rank test |
| Accuracy | DeepSeek vs Doubao | 44 | -0.26 | -0.45--0.08 | 0.00 | -0.20-0.00 | -0.563 | -0.894--0.156 | 0.025 | Paired Wilcoxon signed-rank test |
| Empathy | ChatGPT vs Gemini | 44 | 0.03 | -0.12-0.18 | 0.00 | 0.00-0.00 | 0.114 | -0.368-0.595 | 0.737 | Paired Wilcoxon signed-rank test |
| Empathy | ChatGPT vs Qianwen | 44 | 0.17 | 0.00-0.33 | 0.00 | 0.00-0.00 | 0.450 | 0.004-0.837 | 0.097 | Paired Wilcoxon signed-rank test |
| Empathy | ChatGPT vs DeepSeek | 44 | -0.22 | -0.36--0.08 | 0.00 | 0.00-0.00 | -0.575 | -0.850--0.188 | 0.029 | Paired Wilcoxon signed-rank test |
| Empathy | ChatGPT vs Doubao | 44 | -0.19 | -0.34--0.04 | 0.00 | 0.00-0.00 | -0.552 | -0.900--0.089 | 0.058 | Paired Wilcoxon signed-rank test |
| Empathy | Gemini vs Qianwen | 44 | 0.14 | -0.05-0.33 | 0.00 | 0.00-0.00 | 0.262 | -0.187-0.635 | 0.289 | Paired Wilcoxon signed-rank test |
| Empathy | Gemini vs DeepSeek | 44 | -0.25 | -0.41--0.07 | 0.00 | -0.50-0.00 | -0.542 | -0.852--0.155 | 0.029 | Paired Wilcoxon signed-rank test |
| Empathy | Gemini vs Doubao | 44 | -0.22 | -0.40--0.04 | 0.00 | -0.20-0.00 | -0.471 | -0.855--0.020 | 0.077 | Paired Wilcoxon signed-rank test |
| Empathy | Qianwen vs DeepSeek | 44 | -0.39 | -0.58--0.19 | 0.00 | -0.80-0.00 | -0.690 | -0.949--0.327 | 0.011 | Paired Wilcoxon signed-rank test |
| Empathy | Qianwen vs Doubao | 44 | -0.36 | -0.55--0.17 | 0.00 | -0.80-0.00 | -0.653 | -0.910--0.320 | 0.011 | Paired Wilcoxon signed-rank test |
| Empathy | DeepSeek vs Doubao | 44 | 0.03 | -0.09-0.16 | 0.00 | 0.00-0.00 | 0.014 | -0.511-0.495 | 0.969 | Paired Wilcoxon signed-rank test |
| DISCERN | ChatGPT vs Gemini | 44 | 5.20 | 3.79-6.64 | 6.10 | 3.50-7.60 | 0.853 | 0.685-0.964 | <0.001 | Paired Wilcoxon signed-rank test |
| DISCERN | ChatGPT vs Qianwen | 44 | 0.71 | -0.82-2.27 | 0.70 | -1.00-1.80 | 0.157 | -0.182-0.484 | 0.410 | Paired Wilcoxon signed-rank test |
| DISCERN | ChatGPT vs DeepSeek | 44 | 4.07 | 2.13-6.10 | 3.80 | 1.00-5.60 | 0.635 | 0.351-0.846 | <0.001 | Paired Wilcoxon signed-rank test |
| DISCERN | ChatGPT vs Doubao | 44 | -0.10 | -1.45-1.30 | 0.80 | -2.60-1.70 | -0.020 | -0.351-0.323 | 0.912 | Paired Wilcoxon signed-rank test |
| DISCERN | Gemini vs Qianwen | 44 | -4.48 | -6.20--2.87 | -5.10 | -6.60--2.90 | -0.733 | -0.906--0.498 | <0.001 | Paired Wilcoxon signed-rank test |
| DISCERN | Gemini vs DeepSeek | 44 | -1.12 | -2.55-0.49 | -1.80 | -2.80-0.20 | -0.350 | -0.638--0.016 | 0.066 | Paired Wilcoxon signed-rank test |
| DISCERN | Gemini vs Doubao | 44 | -5.29 | -6.60--3.93 | -5.00 | -6.60--3.80 | -0.900 | -0.984--0.768 | <0.001 | Paired Wilcoxon signed-rank test |
| DISCERN | Qianwen vs DeepSeek | 44 | 3.36 | 1.44-5.25 | 2.70 | 0.20-4.00 | 0.525 | 0.233-0.765 | 0.004 | Paired Wilcoxon signed-rank test |
| DISCERN | Qianwen vs Doubao | 44 | -0.81 | -2.03-0.37 | -1.50 | -2.80-0.40 | -0.246 | -0.573-0.094 | 0.202 | Paired Wilcoxon signed-rank test |
| DISCERN | DeepSeek vs Doubao | 44 | -4.17 | -5.98--2.44 | -4.20 | -5.30--1.50 | -0.662 | -0.861--0.410 | <0.001 | Paired Wilcoxon signed-rank test |
| EQIP | ChatGPT vs Gemini | 44 | 6.50 | 4.02-8.93 | 7.50 | 3.50-11.00 | 0.729 | 0.472-0.917 | <0.001 | Paired Wilcoxon signed-rank test |
| EQIP | ChatGPT vs Qianwen | 44 | 1.39 | -0.80-3.68 | 2.00 | -2.00-4.50 | 0.193 | -0.146-0.521 | 0.349 | Paired Wilcoxon signed-rank test |
| EQIP | ChatGPT vs DeepSeek | 44 | 2.61 | 0.07-5.32 | 2.50 | -1.00-7.00 | 0.289 | -0.050-0.608 | 0.143 | Paired Wilcoxon signed-rank test |
| EQIP | ChatGPT vs Doubao | 44 | -0.86 | -2.95-1.11 | -0.50 | -3.00-2.50 | -0.084 | -0.410-0.277 | 0.645 | Paired Wilcoxon signed-rank test |
| EQIP | Gemini vs Qianwen | 44 | -5.11 | -7.52--2.57 | -5.00 | -8.00--1.50 | -0.629 | -0.867--0.337 | 0.001 | Paired Wilcoxon signed-rank test |
| EQIP | Gemini vs DeepSeek | 44 | -3.89 | -6.59--0.93 | -5.00 | -8.00-0.00 | -0.466 | -0.743--0.144 | 0.016 | Paired Wilcoxon signed-rank test |
| EQIP | Gemini vs Doubao | 44 | -7.36 | -9.55--5.25 | -7.50 | -10.00--5.00 | -0.833 | -0.971--0.630 | <0.001 | Paired Wilcoxon signed-rank test |
| EQIP | Qianwen vs DeepSeek | 44 | 1.23 | -1.34-3.91 | 0.50 | -2.50-4.00 | 0.102 | -0.244-0.425 | 0.624 | Paired Wilcoxon signed-rank test |
| EQIP | Qianwen vs Doubao | 44 | -2.25 | -3.89--0.66 | -1.50 | -4.50-1.00 | -0.411 | -0.682--0.092 | 0.034 | Paired Wilcoxon signed-rank test |
| EQIP | DeepSeek vs Doubao | 44 | -3.48 | -6.00--1.30 | -3.00 | -4.50--1.00 | -0.546 | -0.812--0.244 | 0.006 | Paired Wilcoxon signed-rank test |
| JAMA | ChatGPT vs Gemini | 44 | 0.29 | 0.04-0.54 | 0.20 | 0.00-0.80 | 0.367 | 0.003-0.690 | 0.126 | Paired Wilcoxon signed-rank test |
| JAMA | ChatGPT vs Qianwen | 44 | -0.26 | -0.56-0.02 | 0.00 | 0.00-0.00 | -0.345 | -0.694-0.071 | 0.175 | Paired Wilcoxon signed-rank test |
| JAMA | ChatGPT vs DeepSeek | 44 | 0.10 | -0.16-0.34 | 0.00 | 0.00-0.20 | 0.134 | -0.230-0.531 | 0.521 | Paired Wilcoxon signed-rank test |
| JAMA | ChatGPT vs Doubao | 44 | -0.10 | -0.41-0.20 | 0.00 | -0.20-0.10 | -0.125 | -0.493-0.262 | 0.521 | Paired Wilcoxon signed-rank test |
| JAMA | Gemini vs Qianwen | 44 | -0.55 | -0.80--0.28 | -0.80 | -1.00--0.20 | -0.580 | -0.857--0.263 | 0.019 | Paired Wilcoxon signed-rank test |
| JAMA | Gemini vs DeepSeek | 44 | -0.19 | -0.41-0.04 | -0.20 | -0.70-0.00 | -0.318 | -0.676-0.052 | 0.175 | Paired Wilcoxon signed-rank test |
| JAMA | Gemini vs Doubao | 44 | -0.39 | -0.69--0.08 | -0.20 | -0.80-0.00 | -0.405 | -0.727--0.064 | 0.122 | Paired Wilcoxon signed-rank test |
| JAMA | Qianwen vs DeepSeek | 44 | 0.35 | 0.10-0.63 | 0.00 | 0.00-0.80 | 0.437 | 0.048-0.729 | 0.122 | Paired Wilcoxon signed-rank test |
| JAMA | Qianwen vs Doubao | 44 | 0.15 | -0.11-0.43 | 0.00 | 0.00-0.20 | 0.179 | -0.251-0.563 | 0.481 | Paired Wilcoxon signed-rank test |
| JAMA | DeepSeek vs Doubao | 44 | -0.20 | -0.51-0.10 | 0.00 | -0.20-0.00 | -0.215 | -0.566-0.175 | 0.394 | Paired Wilcoxon signed-rank test |
| GQS | ChatGPT vs Gemini | 44 | 0.42 | 0.28-0.57 | 0.00 | 0.00-0.90 | 0.926 | 0.762-0.993 | <0.001 | Paired Wilcoxon signed-rank test |
| GQS | ChatGPT vs Qianwen | 44 | 0.19 | -0.00-0.38 | 0.00 | 0.00-0.00 | 0.393 | -0.037-0.768 | 0.112 | Paired Wilcoxon signed-rank test |
| GQS | ChatGPT vs DeepSeek | 44 | 0.12 | -0.05-0.29 | 0.00 | 0.00-0.00 | 0.299 | -0.205-0.731 | 0.248 | Paired Wilcoxon signed-rank test |
| GQS | ChatGPT vs Doubao | 44 | -0.14 | -0.32-0.04 | 0.00 | 0.00-0.00 | -0.402 | -0.776-0.046 | 0.112 | Paired Wilcoxon signed-rank test |
| GQS | Gemini vs Qianwen | 44 | -0.24 | -0.39--0.08 | 0.00 | -0.20-0.00 | -0.587 | -0.886--0.200 | 0.022 | Paired Wilcoxon signed-rank test |
| GQS | Gemini vs DeepSeek | 44 | -0.30 | -0.45--0.15 | 0.00 | -0.20-0.00 | -0.792 | -0.986--0.457 | 0.004 | Paired Wilcoxon signed-rank test |
| GQS | Gemini vs Doubao | 44 | -0.56 | -0.70--0.41 | -0.90 | -1.00-0.00 | -0.995 | -1.000--0.963 | <0.001 | Paired Wilcoxon signed-rank test |
| GQS | Qianwen vs DeepSeek | 44 | -0.06 | -0.27-0.14 | 0.00 | 0.00-0.00 | -0.106 | -0.500-0.325 | 0.632 | Paired Wilcoxon signed-rank test |
| GQS | Qianwen vs Doubao | 44 | -0.33 | -0.54--0.11 | 0.00 | -1.00-0.00 | -0.527 | -0.826--0.159 | 0.022 | Paired Wilcoxon signed-rank test |
| GQS | DeepSeek vs Doubao | 44 | -0.26 | -0.45--0.08 | 0.00 | -0.20-0.00 | -0.543 | -0.873--0.130 | 0.033 | Paired Wilcoxon signed-rank test |
| ARI | ChatGPT vs Gemini | 44 | -1.39 | -1.98--0.82 | -1.14 | -2.42--0.96 | -0.687 | -0.875--0.430 | <0.001 | Paired Wilcoxon signed-rank test |
| ARI | ChatGPT vs Qianwen | 44 | -2.07 | -2.53--1.63 | -2.06 | -2.33--1.46 | -0.959 | -0.998--0.879 | <0.001 | Paired Wilcoxon signed-rank test |
| ARI | ChatGPT vs DeepSeek | 44 | -0.56 | -1.20-0.03 | -0.24 | -0.88-0.26 | -0.240 | -0.549-0.107 | 0.167 | Paired Wilcoxon signed-rank test |
| ARI | ChatGPT vs Doubao | 44 | -5.60 | -6.30--4.99 | -5.51 | -6.09--4.75 | -1.000 | -1.000--1.000 | <0.001 | Paired Wilcoxon signed-rank test |
| ARI | Gemini vs Qianwen | 44 | -0.67 | -1.17--0.14 | -0.74 | -1.24--0.21 | -0.417 | -0.714--0.079 | 0.019 | Paired Wilcoxon signed-rank test |
| ARI | Gemini vs DeepSeek | 44 | 0.83 | 0.25-1.43 | 0.68 | 0.10-1.96 | 0.441 | 0.137-0.725 | 0.014 | Paired Wilcoxon signed-rank test |
| ARI | Gemini vs Doubao | 44 | -4.21 | -4.87--3.59 | -3.85 | -4.17--3.15 | -1.000 | -1.000--1.000 | <0.001 | Paired Wilcoxon signed-rank test |
| ARI | Qianwen vs DeepSeek | 44 | 1.50 | 0.93-2.07 | 1.55 | 1.21-2.27 | 0.711 | 0.453-0.911 | <0.001 | Paired Wilcoxon signed-rank test |
| ARI | Qianwen vs Doubao | 44 | -3.54 | -4.21--2.88 | -2.92 | -4.09--2.40 | -1.000 | -1.000--1.000 | <0.001 | Paired Wilcoxon signed-rank test |
| ARI | DeepSeek vs Doubao | 44 | -5.04 | -5.85--4.24 | -5.09 | -5.79--4.05 | -0.988 | -1.000--0.952 | <0.001 | Paired Wilcoxon signed-rank test |
| CL | ChatGPT vs Gemini | 44 | 0.85 | 0.37-1.33 | 0.76 | 0.02-1.54 | 0.492 | 0.178-0.746 | 0.006 | Paired Wilcoxon signed-rank test |
| CL | ChatGPT vs Qianwen | 44 | -2.46 | -2.89--2.02 | -2.34 | -3.03--1.92 | -0.982 | -1.000--0.933 | <0.001 | Paired Wilcoxon signed-rank test |
| CL | ChatGPT vs DeepSeek | 44 | -0.89 | -1.55--0.27 | -0.26 | -1.48-0.27 | -0.352 | -0.642--0.008 | 0.047 | Paired Wilcoxon signed-rank test |
| CL | ChatGPT vs Doubao | 44 | -1.36 | -1.89--0.80 | -1.32 | -2.14--0.44 | -0.694 | -0.881--0.449 | <0.001 | Paired Wilcoxon signed-rank test |
| CL | Gemini vs Qianwen | 44 | -3.31 | -3.78--2.84 | -3.56 | -3.79--3.09 | -0.996 | -1.000--0.976 | <0.001 | Paired Wilcoxon signed-rank test |
| CL | Gemini vs DeepSeek | 44 | -1.74 | -2.36--1.11 | -1.02 | -1.84--0.72 | -0.777 | -0.933--0.568 | <0.001 | Paired Wilcoxon signed-rank test |
| CL | Gemini vs Doubao | 44 | -2.21 | -2.77--1.68 | -2.10 | -2.75--1.63 | -0.913 | -0.986--0.788 | <0.001 | Paired Wilcoxon signed-rank test |
| CL | Qianwen vs DeepSeek | 44 | 1.57 | 0.94-2.18 | 1.87 | 0.96-2.61 | 0.673 | 0.396-0.899 | <0.001 | Paired Wilcoxon signed-rank test |
| CL | Qianwen vs Doubao | 44 | 1.10 | 0.47-1.66 | 1.50 | 0.62-2.05 | 0.600 | 0.302-0.852 | <0.001 | Paired Wilcoxon signed-rank test |
| CL | DeepSeek vs Doubao | 44 | -0.47 | -1.23-0.34 | -1.08 | -1.60--0.02 | -0.297 | -0.617-0.036 | 0.087 | Paired Wilcoxon signed-rank test |
| FKGL | ChatGPT vs Gemini | 44 | -1.14 | -1.66--0.62 | -1.25 | -1.89--0.71 | -0.624 | -0.842--0.347 | <0.001 | Paired Wilcoxon signed-rank test |
| FKGL | ChatGPT vs Qianwen | 44 | -2.12 | -2.57--1.69 | -2.02 | -2.40--1.37 | -0.980 | -1.000--0.927 | <0.001 | Paired Wilcoxon signed-rank test |
| FKGL | ChatGPT vs DeepSeek | 44 | -0.39 | -0.93-0.10 | -0.15 | -0.81-0.46 | -0.202 | -0.505-0.139 | 0.246 | Paired Wilcoxon signed-rank test |
| FKGL | ChatGPT vs Doubao | 44 | -4.47 | -5.13--3.91 | -4.38 | -5.17--3.52 | -1.000 | -1.000--1.000 | <0.001 | Paired Wilcoxon signed-rank test |
| FKGL | Gemini vs Qianwen | 44 | -0.98 | -1.43--0.53 | -0.93 | -1.51--0.57 | -0.624 | -0.861--0.361 | <0.001 | Paired Wilcoxon signed-rank test |
| FKGL | Gemini vs DeepSeek | 44 | 0.75 | 0.23-1.26 | 0.72 | -0.01-1.73 | 0.439 | 0.106-0.713 | 0.013 | Paired Wilcoxon signed-rank test |
| FKGL | Gemini vs Doubao | 44 | -3.33 | -3.88--2.76 | -2.77 | -3.73--2.23 | -1.000 | -1.000--1.000 | <0.001 | Paired Wilcoxon signed-rank test |
| FKGL | Qianwen vs DeepSeek | 44 | 1.73 | 1.24-2.17 | 1.83 | 1.57-2.42 | 0.857 | 0.673-0.978 | <0.001 | Paired Wilcoxon signed-rank test |
| FKGL | Qianwen vs Doubao | 44 | -2.35 | -3.00--1.77 | -2.12 | -3.08--1.27 | -0.948 | -1.000--0.855 | <0.001 | Paired Wilcoxon signed-rank test |
| FKGL | DeepSeek vs Doubao | 44 | -4.07 | -4.70--3.36 | -4.13 | -4.65--3.11 | -0.966 | -1.000--0.885 | <0.001 | Paired Wilcoxon signed-rank test |
| GFI | ChatGPT vs Gemini | 44 | -1.26 | -1.81--0.66 | -1.32 | -2.10--0.86 | -0.632 | -0.853--0.355 | <0.001 | Paired Wilcoxon signed-rank test |
| GFI | ChatGPT vs Qianwen | 44 | -2.62 | -3.21--2.08 | -2.38 | -3.27--1.74 | -0.988 | -1.000--0.943 | <0.001 | Paired Wilcoxon signed-rank test |
| GFI | ChatGPT vs DeepSeek | 44 | -0.63 | -1.21--0.10 | -0.55 | -1.31-0.26 | -0.339 | -0.634--0.006 | 0.051 | Paired Wilcoxon signed-rank test |
| GFI | ChatGPT vs Doubao | 44 | -4.72 | -5.38--4.09 | -4.51 | -5.39--3.66 | -1.000 | -1.000--1.000 | <0.001 | Paired Wilcoxon signed-rank test |
| GFI | Gemini vs Qianwen | 44 | -1.37 | -1.91--0.81 | -1.39 | -1.78--0.89 | -0.705 | -0.899--0.451 | <0.001 | Paired Wilcoxon signed-rank test |
| GFI | Gemini vs DeepSeek | 44 | 0.62 | -0.01-1.23 | 0.76 | 0.33-1.46 | 0.345 | 0.008-0.669 | 0.051 | Paired Wilcoxon signed-rank test |
| GFI | Gemini vs Doubao | 44 | -3.47 | -4.06--2.89 | -2.74 | -3.85--2.39 | -1.000 | -1.000--1.000 | <0.001 | Paired Wilcoxon signed-rank test |
| GFI | Qianwen vs DeepSeek | 44 | 1.99 | 1.35-2.57 | 1.90 | 1.23-2.92 | 0.816 | 0.632-0.952 | <0.001 | Paired Wilcoxon signed-rank test |
| GFI | Qianwen vs Doubao | 44 | -2.10 | -2.81--1.42 | -1.97 | -2.88--1.36 | -0.778 | -0.919--0.562 | <0.001 | Paired Wilcoxon signed-rank test |
| GFI | DeepSeek vs Doubao | 44 | -4.09 | -4.84--3.36 | -4.08 | -4.86--3.37 | -0.958 | -1.000--0.861 | <0.001 | Paired Wilcoxon signed-rank test |
| SMOG | ChatGPT vs Gemini | 44 | -1.09 | -1.45--0.72 | -1.17 | -1.48--0.86 | -0.752 | -0.927--0.515 | <0.001 | Paired Wilcoxon signed-rank test |
| SMOG | ChatGPT vs Qianwen | 44 | -1.38 | -1.76--1.04 | -1.19 | -1.63--0.86 | -0.925 | -0.986--0.806 | <0.001 | Paired Wilcoxon signed-rank test |
| SMOG | ChatGPT vs DeepSeek | 44 | -0.25 | -0.63-0.15 | -0.14 | -0.76-0.43 | -0.202 | -0.523-0.139 | 0.245 | Paired Wilcoxon signed-rank test |
| SMOG | ChatGPT vs Doubao | 44 | -3.40 | -3.86--2.94 | -3.34 | -3.85--2.77 | -1.000 | -1.000--1.000 | <0.001 | Paired Wilcoxon signed-rank test |
| SMOG | Gemini vs Qianwen | 44 | -0.29 | -0.64-0.06 | -0.13 | -0.70--0.01 | -0.288 | -0.586-0.044 | 0.108 | Paired Wilcoxon signed-rank test |
| SMOG | Gemini vs DeepSeek | 44 | 0.84 | 0.44-1.22 | 0.80 | 0.44-1.42 | 0.656 | 0.376-0.893 | <0.001 | Paired Wilcoxon signed-rank test |
| SMOG | Gemini vs Doubao | 44 | -2.31 | -2.77--1.88 | -1.82 | -2.89--1.49 | -0.998 | -1.000--0.988 | <0.001 | Paired Wilcoxon signed-rank test |
| SMOG | Qianwen vs DeepSeek | 44 | 1.13 | 0.75-1.48 | 1.16 | 0.73-1.78 | 0.790 | 0.570-0.960 | <0.001 | Paired Wilcoxon signed-rank test |
| SMOG | Qianwen vs Doubao | 44 | -2.02 | -2.55--1.53 | -1.90 | -2.90--1.02 | -0.923 | -0.988--0.808 | <0.001 | Paired Wilcoxon signed-rank test |
| SMOG | DeepSeek vs Doubao | 44 | -3.15 | -3.70--2.56 | -3.48 | -3.85--2.35 | -0.966 | -1.000--0.893 | <0.001 | Paired Wilcoxon signed-rank test |
| FRES | ChatGPT vs Gemini | 44 | -1.11 | -4.01-1.73 | 1.05 | -3.95-2.66 | -0.079 | -0.402-0.271 | 0.653 | Paired Wilcoxon signed-rank test |
| FRES | ChatGPT vs Qianwen | 44 | 15.20 | 12.51-17.87 | 13.88 | 10.42-19.06 | 0.988 | 0.943-1.000 | <0.001 | Paired Wilcoxon signed-rank test |
| FRES | ChatGPT vs DeepSeek | 44 | 3.25 | -0.04-6.40 | 1.58 | -2.90-6.32 | 0.287 | -0.048-0.584 | 0.110 | Paired Wilcoxon signed-rank test |
| FRES | ChatGPT vs Doubao | 44 | 12.49 | 9.27-15.80 | 11.38 | 8.40-14.84 | 0.925 | 0.798-1.000 | <0.001 | Paired Wilcoxon signed-rank test |
| FRES | Gemini vs Qianwen | 44 | 16.30 | 13.67-19.06 | 16.34 | 13.87-18.34 | 0.994 | 0.970-1.000 | <0.001 | Paired Wilcoxon signed-rank test |
| FRES | Gemini vs DeepSeek | 44 | 4.36 | 1.30-7.65 | 1.37 | 0.01-6.16 | 0.362 | 0.026-0.651 | 0.053 | Paired Wilcoxon signed-rank test |
| FRES | Gemini vs Doubao | 44 | 13.60 | 10.54-16.91 | 13.88 | 7.99-18.32 | 0.941 | 0.840-0.998 | <0.001 | Paired Wilcoxon signed-rank test |
| FRES | Qianwen vs DeepSeek | 44 | -11.94 | -14.90--8.63 | -14.21 | -16.29--11.40 | -0.873 | -0.972--0.713 | <0.001 | Paired Wilcoxon signed-rank test |
| FRES | Qianwen vs Doubao | 44 | -2.71 | -6.24-1.07 | -2.90 | -7.51-1.46 | -0.287 | -0.590-0.051 | 0.110 | Paired Wilcoxon signed-rank test |
| FRES | DeepSeek vs Doubao | 44 | 9.23 | 5.22-13.25 | 10.29 | 5.87-15.53 | 0.687 | 0.422-0.911 | <0.001 | Paired Wilcoxon signed-rank test |

Note: Pairwise comparisons were performed using paired Wilcoxon signed-rank tests with Benjamini-Hochberg correction. Paired mean/median differences and rank-biserial correlations are reported with bootstrap 95% CIs.
