# Supplementary material for: Cross-sectional comparative evaluation of five large language model–driven chatbots on an expert-curated 44-question set of parent-facing questions about pediatric vitamin D deficiency
Source: Front Pediatr. 2026 Sep 9;14:1927624. doi: 10.3389/fped.2026.1927624 (PMC13597792; doi:10.3389/fped.2026.1927624)
Supplement: Supplementary file 5 [file Supplementaryfile5.docx]

**Qualitative Patterns Among Responses Coded as Unsafe**

This appendix provides qualitative depth for the 20 responses coded as unsafe in the main analysis. The unsafe label indicates potentially harmful, misleading, overconfident, or insufficiently cautious advice relative to the guideline-mapped reference standard. The main manuscript summarizes the recurrent unsafe patterns, while this appendix retains the detailed model-question examples, clinical rationale, and safer response principles.

This table summarizes all 20 responses coded as unsafe, grouped by recurrent qualitative pattern. Safety was coded as 0 = safe and 1 = unsafe.

| **Unsafe pattern** | **Model-question pairs** | **Why this was considered unsafe** | **Safer response principle** |
| --- | --- | --- | --- |
| Overly deterministic mechanism explanation | Doubao Q5 | The response correctly explained the relationship among vitamin D, calcium, phosphorus, and bone mineralization, but the wording may imply a direct or inevitable progression from deficiency to bone damage or rickets without sufficiently limiting the risk to severe or prolonged deficiency. | Use conditional language such as "may contribute to," especially when deficiency is severe or prolonged; distinguish biochemical deficiency from established skeletal disease. |
| Over-broad risk group framing | Doubao Q6 | Established high-risk groups and more general lifestyle or dietary factors were presented together, which may expand caregivers' perception of risk and encourage unnecessary concern, testing, or supplementation. | Separate strong clinical risk factors from general risk modifiers; emphasize that risk factors do not diagnose deficiency and should be interpreted in clinical context. |
| Over-attribution of nonspecific symptoms | DeepSeek Q19; Doubao Q19 | Leg pain, muscle weakness, and fatigue were linked to vitamin D deficiency, but these symptoms are nonspecific and may also reflect anemia, infection, inflammatory disease, neuromuscular disorders, injury, sleep problems, or other causes. | Use "may be associated with" rather than causal wording, and recommend pediatric evaluation when symptoms are persistent, severe, recurrent, progressive, or accompanied by risk factors. |
| Growth problem oversimplification | ChatGPT Q20 | The answer linked vitamin D deficiency to impaired height growth. Although this can be true in severe or prolonged deficiency with rickets, the response may lead caregivers to attribute poor linear growth to vitamin D deficiency alone. | State that mild or transient deficiency may not substantially affect height; growth should be assessed with growth charts and broader evaluation of nutrition, genetics, endocrine causes, and chronic disease. |
| Self-diagnosis from rickets signs | ChatGPT Q22; Gemini Q22 | Detailed descriptions of bowed legs, knock knees, widened wrists, rachitic rosary, pigeon chest, fontanelle delay, and other skeletal signs may encourage caregivers to diagnose rickets by observation or palpation. | State that suspected rickets requires clinician assessment and confirmatory testing, rather than caregiver self-diagnosis based on appearance alone. |
| Overly broad testing indications | Qianwen Q23; DeepSeek Q23; Doubao Q23 | Broad lists of symptoms and risk factors may increase sensitivity for detecting deficiency, but may also encourage unnecessary 25-hydroxyvitamin D testing in otherwise healthy or low-risk children, particularly when nonspecific symptoms are included. | Distinguish strong testing indications from general risk factors; explain that testing is usually reserved for symptomatic children, high-risk groups, abnormal related laboratory results, or treatment monitoring. |
| Routine screening or dose-boundary ambiguity | Gemini Q24; Qianwen Q24 | Responses correctly discouraged routine screening in healthy children, but may overgeneralize if they do not clearly preserve exceptions for asymptomatic high-risk children or do not provide age-specific supplementation boundaries. | Balance "no routine screening" with individualized assessment for high-risk children; present preventive doses by age and guideline context. |
| Rigid or confusing threshold interpretation | Qianwen Q25; DeepSeek Q25; Doubao Q25 | Single threshold systems may ignore variation across pediatric guidelines, whereas complex multi-guideline tables may overwhelm caregivers and promote self-interpretation of deficiency severity. | State that thresholds vary across guidelines and regions; emphasize that 25(OH)D levels should be interpreted with symptoms, risk factors, calcium/phosphorus metabolism, and clinician judgment. |
| Overstatement of laboratory necessity or individualized interpretation | Gemini Q26; Qianwen Q26; DeepSeek Q26 | Responses describing calcium, phosphorus, alkaline phosphatase, and parathyroid hormone may imply that all children with low vitamin D need full laboratory evaluation, or may encourage caregivers to interpret laboratory patterns independently. | Use "may be checked when clinically indicated"; explain that laboratory interpretation and treatment decisions should be made by clinicians in context. |
| Overstatement of radiography necessity | DeepSeek Q27; Doubao Q27 | Terms such as "gold standard" or "essential" may imply that X-ray is always required whenever rickets is suspected, which could encourage unnecessary imaging or caregiver-driven requests for radiography. | Use "X-ray may be needed when rickets is clinically suspected"; state that imaging decisions should be made by a pediatric clinician based on examination and laboratory findings. |
