# Supplementary material for: Cross-sectional comparative evaluation of five large language model–driven chatbots on an expert-curated 44-question set of parent-facing questions about pediatric vitamin D deficiency
Source: Front Pediatr. 2026 Sep 9;14:1927624. doi: 10.3389/fped.2026.1927624 (PMC13597792; doi:10.3389/fped.2026.1927624)
Supplement: Supplementary file 6 [file Supplementaryfile7.docx]

**Appendix 8. R code for statistical analysis and figure generation**

```
# ==========================================================
# 大语言模型健康问题回答评价：统计分析 + 作图 + 分布图整合版
# 新增指标：Accuracy（1-5分）、Safety（0=安全，1=不安全）、Empathy（1-5分）
# 主分析：按 question 配对比较不同模型
# 输出：
#   1) Excel：完整统计结果
#   2) TIFF：图1-图3柱状图，以及与图1-图3对应的三张分布图
# ==========================================================

# ---------- 0. 基础设置 ----------
getwd()
work_dir <- getwd()
input_file <- file.path(work_dir, "data.xlsx")
output_dir <- file.path(work_dir, "LLM_analysis_outputs")
plot_dir <- file.path(output_dir, "plots")

if (!dir.exists(output_dir)) dir.create(output_dir, recursive = TRUE)
if (!dir.exists(plot_dir)) dir.create(plot_dir, recursive = TRUE)

packages <- c(
  "readxl", "dplyr", "tidyr", "openxlsx", "ggplot2", "patchwork",
  "FSA", "stringr", "purrr", "scales",
  "officer", "flextable", "tibble", "irr", "psych"
)

for (pkg in packages) {
  if (!requireNamespace(pkg, quietly = TRUE)) install.packages(pkg)
  library(pkg, character.only = TRUE)
}

# ---------- 1. 指标分组 ----------
# 文章结果与图表顺序：
# 1) Safety 安全性
# 2) Accuracy 准确性
# 3) Empathy 同理心
# 4) Reliability/quality 可靠性/质量
# 5) Readability 可读性
safety_metrics <- c("Safety")
accuracy_metrics <- c("Accuracy")
empathy_metrics <- c("Empathy")
reliability_metrics <- c("DISCERN", "EQIP", "JAMA", "GQS")
readability_metrics <- c("ARI", "CL", "FKGL", "GFI", "SMOG", "FRES")
performance_metrics <- c(safety_metrics, accuracy_metrics, empathy_metrics)
all_metrics <- c(safety_metrics, accuracy_metrics, empathy_metrics, reliability_metrics, readability_metrics)

# 模型不预设固定名称；后续完全根据数据中的 model 列自动识别。

higher_better_metrics <- c("DISCERN", "EQIP", "JAMA", "GQS", "Accuracy", "Empathy", "FRES")
lower_better_metrics  <- c("Safety", "ARI", "CL", "FKGL", "GFI", "SMOG")

# Safety coding in the current dataset:
#   0 = Safe; 1 = Unsafe
# If your future dataset changes this coding, only modify the two values below.
safety_safe_value <- 0
safety_unsafe_value <- 1

ordinal_metrics <- c("GQS", "JAMA", "Accuracy", "Empathy")
binary_metrics <- c("Safety")
continuous_metrics <- setdiff(all_metrics, c(ordinal_metrics, binary_metrics))

# ---------- 2. 常用函数 ----------
normalize_model <- function(x) {
  # Do not hard-code, standardize, or correct any model names here.
  # Model names are read directly from the model column in the dataset.
  # Only leading/trailing/repeated spaces are cleaned to avoid accidental duplicate levels.
  y <- stringr::str_squish(as.character(x))
  y[y == ""] <- NA_character_
  y
}

sort_question_levels <- function(x) {
  x <- unique(as.character(x))
  xn <- suppressWarnings(as.numeric(x))
  x[order(is.na(xn), xn, x)]
}

safe_shapiro <- function(x) {
  x <- x[!is.na(x)]
  if (length(x) < 3 || length(unique(x)) < 3) return(NA_real_)
  tryCatch(shapiro.test(x)$p.value, error = function(e) NA_real_)
}

round4 <- function(x) {
  ifelse(is.na(x), NA, round(as.numeric(x), 4))
}

fmt_p <- function(x) {
  dplyr::case_when(
    is.na(x) ~ NA_character_,
    x < 0.001 ~ "<0.001",
    TRUE ~ sprintf("%.3f", x)
  )
}

metric_group_name <- function(x) {
  dplyr::case_when(
    x %in% safety_metrics ~ "1. Safety",
    x %in% accuracy_metrics ~ "2. Accuracy",
    x %in% empathy_metrics ~ "3. Empathy",
    x %in% reliability_metrics ~ "4. Reliability/quality",
    x %in% readability_metrics ~ "5. Readability",
    TRUE ~ "Other"
  )
}

metric_direction <- function(x) {
  dplyr::case_when(
    x %in% safety_metrics ~ "Safety: 0 = safe; 1 = unsafe",
    x %in% higher_better_metrics ~ "Higher score indicates better performance",
    x %in% c("ARI", "CL", "FKGL", "GFI", "SMOG") ~ "Lower score indicates easier readability",
    TRUE ~ ""
  )
}

empty_df <- function(note = "No available result") {
  data.frame(Note = note, stringsAsFactors = FALSE)
}

add_sheet <- function(wb, sheet_name, df) {
  sheet_name <- substr(sheet_name, 1, 31)
  if (is.null(df) || nrow(df) == 0) df <- empty_df()
  addWorksheet(wb, sheet_name)
  writeData(wb, sheet_name, df)
  freezePane(wb, sheet_name, firstRow = TRUE)
  header_style <- createStyle(
    textDecoration = "bold", fgFill = "#D9EAF7",
    halign = "center", valign = "center",
    border = "Bottom"
  )
  addStyle(wb, sheet_name, header_style, rows = 1, cols = 1:ncol(df), gridExpand = TRUE)
  setColWidths(wb, sheet_name, cols = 1:ncol(df), widths = "auto")
}

# 安全筛选函数：当某些指标缺失、模型不足或统计函数返回空表时，避免因为缺列而中断。
safe_filter_sig <- function(df, p_col = "P_adjusted_BH", alpha = 0.05) {
  if (is.null(df) || nrow(df) == 0 || !p_col %in% names(df)) return(empty_df("No significant result available"))
  out <- df %>% dplyr::filter(!is.na(.data[[p_col]]), .data[[p_col]] < alpha)
  if (nrow(out) == 0) return(empty_df("No significant result available"))
  out
}

has_cols <- function(df, cols) {
  !is.null(df) && nrow(df) > 0 && all(cols %in% names(df))
}

# ---------- 3. 读取与清洗数据 ----------

# 优先使用工作区中已读取的数据对象“数据分析”；如果不存在，则自动读取 input_file。
if (exists("数据分析")) {
  raw_data <- 数据分析
} else {
  if (!file.exists(input_file)) {
    stop(paste0("未找到输入文件：", input_file, "。请将 data.xlsx 放到 work_dir，或在环境中提供数据对象：数据分析。"))
  }
  raw_data <- readxl::read_excel(input_file, sheet = 1)
}

raw_data <- as.data.frame(raw_data)
names(raw_data) <- stringr::str_squish(names(raw_data))

required_cols <- c("question", "model", "metric", "score")
missing_cols <- setdiff(required_cols, names(raw_data))
if (length(missing_cols) > 0) {
  stop(paste0("数据缺少以下必要列：", paste(missing_cols, collapse = ", ")))
}

# 自动识别评分员列：rater1, rater2, ...；不再固定为5位评分员。
detect_rater_cols <- function(df) {
  original_names <- names(df)
  matched <- grepl("^rater\\s*_?\\s*[0-9]+$", original_names, ignore.case = TRUE)
  rater_original <- original_names[matched]
  if (length(rater_original) == 0) return(character(0))
  rater_number <- suppressWarnings(as.integer(stringr::str_extract(rater_original, "[0-9]+")))
  ord <- order(rater_number, rater_original)
  rater_original <- rater_original[ord]
  rater_number <- rater_number[ord]
  standardized <- paste0("rater", rater_number)
  names(df)[match(rater_original, names(df))] <- standardized
  assign("raw_data", df, envir = parent.frame())
  unique(standardized)
}

rater_cols <- detect_rater_cols(raw_data)
rater_count <- length(rater_cols)

select_cols <- c(required_cols, rater_cols)

data <- raw_data %>%
  dplyr::select(dplyr::all_of(select_cols)) %>%
  dplyr::filter(
    !is.na(question),
    !is.na(model),
    !is.na(metric),
    !is.na(score)
  ) %>%
  dplyr::mutate(
    question = as.character(question),
    model = normalize_model(model),
    metric = stringr::str_squish(as.character(metric)),
    score = suppressWarnings(as.numeric(score)),
    metric_group = metric_group_name(metric),
    direction = metric_direction(metric)
  ) %>%
  dplyr::mutate(
    dplyr::across(dplyr::all_of(rater_cols), ~ suppressWarnings(as.numeric(.x)))
  ) %>%
  dplyr::filter(metric %in% all_metrics)

question_levels <- sort_question_levels(data$question)

# 自动识别模型：以数据中 model 列首次出现的顺序为准。
# 不在代码中预设任何具体模型名称或模型版本；数据中写什么，结果与图例就显示什么。
# 不管是 2 个、5 个还是更多模型，后续统计、表格和作图都会自动使用这些模型。
detect_models <- function(x) {
  # Automatically read model names from the model column in their first-appearance order.
  # No model names or versions are predefined in the code.
  y <- unique(stringr::str_squish(as.character(x)))
  y <- y[!is.na(y) & y != ""]
  y
}

models_present <- detect_models(data$model)
if (length(models_present) < 1) {
  stop("未能从 model 列识别到任何模型，请检查数据中的 model 列。")
}

# 自动生成模型配色：模型数量改变时无需手动改颜色。
make_model_palette <- function(model_names) {
  # 冗余配色：预留足够颜色；模型数量超过预设时自动插值扩展。
  base_palette <- c(
    "#E64B35", "#4DBBD5", "#00A087", "#3C5488", "#F39B7F",
    "#8491B4", "#91D1C2", "#DC0000", "#7E6148", "#B09C85",
    "#6A51A3", "#E78AC3", "#A6D854", "#FFD92F", "#66C2A5",
    "#FC8D62", "#8DA0CB", "#E5C494", "#B3B3B3", "#1F78B4",
    "#B2DF8A", "#FB9A99", "#FDBF6F", "#CAB2D6", "#FFFF99",
    "#A6761D", "#1B9E77", "#D95F02", "#7570B3", "#E7298A",
    "#66A61E", "#E6AB02", "#A6CEE3", "#33A02C", "#B15928"
  )

  n_model <- length(model_names)
  if (n_model <= length(base_palette)) {
    pal <- base_palette[seq_len(n_model)]
  } else {
    pal <- grDevices::colorRampPalette(base_palette)(n_model)
  }

  names(pal) <- model_names
  pal
}

sci_colors <- make_model_palette(models_present)

metric_group_levels <- c(
  "1. Safety",
  "2. Accuracy",
  "3. Empathy",
  "4. Reliability/quality",
  "5. Readability"
)

data <- data %>%
  dplyr::mutate(
    question = factor(question, levels = question_levels),
    model = factor(model, levels = models_present),
    metric = factor(metric, levels = all_metrics),
    metric_group = factor(metric_group, levels = metric_group_levels)
  )

# ---------- 4. 数据完整性检查 ----------
duplicates <- data %>%
  dplyr::count(question, model, metric, name = "duplicate_n") %>%
  dplyr::filter(duplicate_n > 1)

coverage <- data %>%
  dplyr::count(metric_group, metric, model, name = "n") %>%
  dplyr::arrange(metric_group, metric, model)

expected_grid <- tidyr::expand_grid(
  question = factor(question_levels, levels = question_levels),
  model = factor(models_present, levels = models_present),
  metric = factor(all_metrics, levels = all_metrics)
)

missing_combinations <- expected_grid %>%
  dplyr::anti_join(
    data %>% dplyr::distinct(question, model, metric),
    by = c("question", "model", "metric")
  ) %>%
  dplyr::arrange(metric, question, model)

score_range_check <- data %>%
  dplyr::mutate(
    expected_range = dplyr::case_when(
      metric == "DISCERN" ~ "16-80",
      metric %in% c("EQIP", "FRES") ~ "0-100",
      metric %in% c("ARI", "CL", "FKGL", "GFI", "SMOG") ~ "usually 0-20",
      metric %in% c("GQS", "Accuracy", "Empathy") ~ "1-5",
      metric == "JAMA" ~ "0-4",
      metric == "Safety" ~ "0-1",
      TRUE ~ ""
    ),
    possible_problem = dplyr::case_when(
      metric == "Safety" & !(score %in% c(0, 1)) ~ "Safety should be 0 or 1",
      metric %in% c("Accuracy", "Empathy", "GQS") & (score < 1 | score > 5) ~ "Score should usually be 1-5",
      metric == "JAMA" & (score < 0 | score > 4) ~ "JAMA is outside 0-4 range",
      metric == "DISCERN" & (score < 16 | score > 80) ~ "DISCERN is outside 16-80 range",
      metric %in% c("EQIP", "FRES") & (score < 0 | score > 100) ~ "Score is outside 0-100 range",
      TRUE ~ ""
    )
  ) %>%
  dplyr::filter(possible_problem != "") %>%
  dplyr::select(question, model, metric, score, expected_range, possible_problem)

data_check_summary <- data.frame(
  Item = c(
    "Rows in raw data",
    "Rows after cleaning and metric filtering",
    "Number of questions",
    "Number of models",
    "Number of metrics",
    "Number of detected raters",
    "Detected rater columns",
    "Duplicate question-model-metric rows",
    "Missing question-model-metric combinations",
    "Out-of-range score records"
  ),
  Value = c(
    nrow(raw_data),
    nrow(data),
    length(question_levels),
    length(models_present),
    length(unique(data$metric)),
    rater_count,
    ifelse(rater_count > 0, paste(rater_cols, collapse = "; "), "No rater columns detected"),
    nrow(duplicates),
    nrow(missing_combinations),
    nrow(score_range_check)
  )
)

# ---------- 5. 描述性统计 ----------
descriptive_all <- data %>%
  dplyr::group_by(metric_group, metric, model, direction) %>%
  dplyr::summarise(
    n = sum(!is.na(score)),
    mean = round(mean(score, na.rm = TRUE), 4),
    sd = round(sd(score, na.rm = TRUE), 4),
    median = round(median(score, na.rm = TRUE), 4),
    q1 = round(quantile(score, 0.25, na.rm = TRUE), 4),
    q3 = round(quantile(score, 0.75, na.rm = TRUE), 4),
    iqr = round(IQR(score, na.rm = TRUE), 4),
    min = round(min(score, na.rm = TRUE), 4),
    max = round(max(score, na.rm = TRUE), 4),
    .groups = "drop"
  ) %>%
  dplyr::arrange(metric_group, metric, model)

safety_rate <- data %>%
  dplyr::filter(as.character(metric) == "Safety") %>%
  dplyr::mutate(score = as.numeric(score)) %>%
  dplyr::group_by(model) %>%
  dplyr::summarise(
    n = sum(!is.na(score)),
    safe_n = sum(score == safety_safe_value, na.rm = TRUE),
    unsafe_n = sum(score == safety_unsafe_value, na.rm = TRUE),
    safe_rate_percent = round(100 * safe_n / n, 2),
    unsafe_rate_percent = round(100 * unsafe_n / n, 2),
    .groups = "drop"
  )

normality_results <- data %>%
  dplyr::filter(metric != "Safety") %>%
  dplyr::group_by(metric_group, metric, model) %>%
  dplyr::summarise(
    n = sum(!is.na(score)),
    unique_scores = dplyr::n_distinct(score[!is.na(score)]),
    shapiro_p_value = round4(safe_shapiro(score)),
    normality_note = dplyr::case_when(
      is.na(shapiro_p_value) ~ "Not tested: sample too small or too few unique scores",
      shapiro_p_value >= 0.05 ~ "No evidence against normality",
      shapiro_p_value < 0.05 ~ "Non-normal distribution"
    ),
    .groups = "drop"
  )

# ---------- 5B. 评分者一致性：自动识别rater列，并计算95%CI ----------
# 输出指标：Safety = Fleiss' kappa；Accuracy/Empathy/DISCERN/EQIP/JAMA/GQS = ICC(2,1)。
# Fleiss' kappa 的95%CI采用按条目重抽样的百分位 bootstrap；ICC 的95%CI来自 psych::ICC。

agreement_metrics <- intersect(
  c("Safety", "Accuracy", "Empathy", "DISCERN", "EQIP", "JAMA", "GQS"),
  unique(as.character(data$metric))
)

agreement_boot_n <- 3000
agreement_seed <- 20260513

complete_rater_matrix <- function(df, metric_name) {
  if (length(rater_cols) < 2) return(NULL)
  mat <- df %>%
    dplyr::filter(as.character(metric) == metric_name) %>%
    dplyr::select(dplyr::all_of(rater_cols)) %>%
    dplyr::mutate(dplyr::across(dplyr::everything(), ~ suppressWarnings(as.numeric(.x))))
  mat <- mat[rowSums(!is.na(mat)) > 0, , drop = FALSE]
  mat
}

calc_fleiss_kappa_value <- function(mat) {
  mat <- as.data.frame(mat)
  mat <- mat[stats::complete.cases(mat), , drop = FALSE]
  if (nrow(mat) < 2 || ncol(mat) < 2) return(NA_real_)
  out <- tryCatch(irr::kappam.fleiss(mat), error = function(e) NULL)
  if (is.null(out)) return(NA_real_)
  as.numeric(out$value)
}

bootstrap_ci_percentile <- function(mat, fun, R = 1000, seed = 20260513) {
  mat <- as.data.frame(mat)
  mat <- mat[stats::complete.cases(mat), , drop = FALSE]
  if (nrow(mat) < 3 || ncol(mat) < 2) return(c(lower = NA_real_, upper = NA_real_))
  set.seed(seed)
  vals <- replicate(R, {
    idx <- sample(seq_len(nrow(mat)), size = nrow(mat), replace = TRUE)
    suppressWarnings(fun(mat[idx, , drop = FALSE]))
  })
  vals <- vals[is.finite(vals)]
  if (length(vals) < 20) return(c(lower = NA_real_, upper = NA_real_))
  stats::quantile(vals, probs = c(0.025, 0.975), na.rm = TRUE, names = FALSE) %>%
    setNames(c("lower", "upper"))
}

calc_icc2_1 <- function(mat) {
  mat <- as.data.frame(mat)
  mat <- mat[stats::complete.cases(mat), , drop = FALSE]
  if (nrow(mat) < 2 || ncol(mat) < 2) {
    return(c(estimate = NA_real_, lower = NA_real_, upper = NA_real_, p = NA_real_))
  }
  out <- tryCatch(psych::ICC(mat)$results, error = function(e) NULL)
  if (is.null(out) || !"type" %in% names(out)) {
    return(c(estimate = NA_real_, lower = NA_real_, upper = NA_real_, p = NA_real_))
  }
  row <- out[out$type == "ICC2", , drop = FALSE]
  if (nrow(row) == 0) {
    return(c(estimate = NA_real_, lower = NA_real_, upper = NA_real_, p = NA_real_))
  }
  c(
    estimate = as.numeric(row$ICC[1]),
    lower = as.numeric(row$lower[1]),
    upper = as.numeric(row$upper[1]),
    p = as.numeric(row$p[1])
  )
}

calc_agreement_one <- function(metric_name) {
  mat_all <- complete_rater_matrix(data, metric_name)
  if (is.null(mat_all)) {
    return(data.frame(
      Metric = metric_name, Agreement_index = NA_character_, Estimate = NA_real_,
      Lower_95_CI = NA_real_, Upper_95_CI = NA_real_, P_value = NA_real_,
      P_value_formatted = NA_character_, N_items = 0, N_complete_items = 0,
      N_raters = rater_count, Rater_columns = paste(rater_cols, collapse = "; "),
      Note = "Fewer than two rater columns detected",
      stringsAsFactors = FALSE
    ))
  }

  mat_complete <- mat_all[stats::complete.cases(mat_all), , drop = FALSE]
  n_items <- nrow(mat_all)
  n_complete <- nrow(mat_complete)

  if (n_complete < 2 || ncol(mat_complete) < 2) {
    return(data.frame(
      Metric = metric_name, Agreement_index = NA_character_, Estimate = NA_real_,
      Lower_95_CI = NA_real_, Upper_95_CI = NA_real_, P_value = NA_real_,
      P_value_formatted = NA_character_, N_items = n_items, N_complete_items = n_complete,
      N_raters = rater_count, Rater_columns = paste(rater_cols, collapse = "; "),
      Note = "Insufficient complete rater data",
      stringsAsFactors = FALSE
    ))
  }

  if (metric_name == "Safety") {
    if (!all(as.matrix(mat_complete) %in% c(safety_safe_value, safety_unsafe_value))) {
      return(data.frame(
        Metric = metric_name, Agreement_index = "Fleiss' kappa", Estimate = NA_real_,
        Lower_95_CI = NA_real_, Upper_95_CI = NA_real_, P_value = NA_real_,
        P_value_formatted = NA_character_, N_items = n_items, N_complete_items = n_complete,
        N_raters = rater_count, Rater_columns = paste(rater_cols, collapse = "; "),
        Note = "Safety rater values contain values outside the configured 0/1 coding",
        stringsAsFactors = FALSE
      ))
    }
    kp <- tryCatch(irr::kappam.fleiss(as.data.frame(mat_complete)), error = function(e) NULL)
    estimate <- ifelse(is.null(kp), NA_real_, as.numeric(kp$value))
    p_value <- ifelse(is.null(kp), NA_real_, as.numeric(kp$p.value))
    ci <- bootstrap_ci_percentile(mat_complete, calc_fleiss_kappa_value, agreement_boot_n, agreement_seed)

    return(data.frame(
      Metric = metric_name, Agreement_index = "Fleiss' kappa",
      Estimate = round4(estimate),
      Lower_95_CI = round4(ci["lower"]),
      Upper_95_CI = round4(ci["upper"]),
      P_value = round4(p_value),
      P_value_formatted = fmt_p(p_value),
      N_items = n_items,
      N_complete_items = n_complete,
      N_raters = rater_count,
      Rater_columns = paste(rater_cols, collapse = "; "),
      Note = paste0("Primary agreement index for Safety; 95% CI based on ", agreement_boot_n, " nonparametric bootstrap resamples."),
      stringsAsFactors = FALSE
    ))
  }

  icc <- calc_icc2_1(mat_complete)
  data.frame(
    Metric = metric_name,
    Agreement_index = "ICC(2,1)",
    Estimate = round4(icc["estimate"]),
    Lower_95_CI = round4(icc["lower"]),
    Upper_95_CI = round4(icc["upper"]),
    P_value = round4(icc["p"]),
    P_value_formatted = fmt_p(icc["p"]),
    N_items = n_items,
    N_complete_items = n_complete,
    N_raters = rater_count,
    Rater_columns = paste(rater_cols, collapse = "; "),
    Note = "Two-way random-effects, absolute-agreement, single-measure ICC.",
    stringsAsFactors = FALSE
  )
}

rater_agreement_results <- if (length(agreement_metrics) == 0 || rater_count < 2) {
  empty_df("No rater agreement result available: fewer than two rater columns or no eligible metrics.")
} else {
  purrr::map_dfr(agreement_metrics, calc_agreement_one) %>%
    dplyr::mutate(
      `95% CI` = dplyr::if_else(
        is.na(Lower_95_CI) | is.na(Upper_95_CI),
        NA_character_,
        paste0(sprintf("%.3f", Lower_95_CI), "-", sprintf("%.3f", Upper_95_CI))
      ),
      Estimate_text = dplyr::if_else(is.na(Estimate), NA_character_, sprintf("%.3f", Estimate))
    ) %>%
    dplyr::arrange(match(Metric, c("Safety", "Accuracy", "Empathy", "DISCERN", "EQIP", "JAMA", "GQS")))
}


# ---------- 6. 主分析：配对设计 ----------
run_friedman_one <- function(df, metric_name) {
  metric_df <- df %>%
    dplyr::filter(metric == metric_name) %>%
    dplyr::select(question, model, score) %>%
    dplyr::distinct(question, model, .keep_all = TRUE) %>%
    tidyr::pivot_wider(names_from = model, values_from = score)

  model_cols <- intersect(models_present, names(metric_df))
  if (length(model_cols) < 2) {
    return(data.frame(
      Metric = metric_name, Test = "Friedman test", N_questions = 0,
      N_models = length(model_cols), Statistic = NA, DF = NA,
      P_value = NA, Kendall_W = NA,
      Note = "Fewer than two models available"
    ))
  }

  metric_complete <- metric_df %>% tidyr::drop_na(dplyr::all_of(model_cols))
  n_q <- nrow(metric_complete)
  k <- length(model_cols)

  if (n_q < 2) {
    return(data.frame(
      Metric = metric_name, Test = "Friedman test", N_questions = n_q,
      N_models = k, Statistic = NA, DF = NA,
      P_value = NA, Kendall_W = NA,
      Note = "Fewer than two complete paired questions"
    ))
  }

  mat <- as.matrix(metric_complete[, model_cols])
  if (length(unique(as.vector(mat))) < 2) {
    return(data.frame(
      Metric = metric_name, Test = "Friedman test", N_questions = n_q,
      N_models = k, Statistic = NA, DF = k - 1,
      P_value = NA, Kendall_W = 0,
      Note = "All scores are identical; test not applicable"
    ))
  }

  out <- tryCatch(friedman.test(mat), error = function(e) NULL)
  if (is.null(out)) {
    return(data.frame(
      Metric = metric_name, Test = "Friedman test", N_questions = n_q,
      N_models = k, Statistic = NA, DF = k - 1,
      P_value = NA, Kendall_W = NA,
      Note = "Friedman test failed"
    ))
  }

  kendall_w <- as.numeric(out$statistic) / (n_q * (k - 1))

  data.frame(
    Metric = metric_name,
    Test = "Friedman test",
    N_questions = n_q,
    N_models = k,
    Statistic = round4(out$statistic),
    DF = as.numeric(out$parameter),
    P_value = round4(out$p.value),
    P_value_formatted = fmt_p(out$p.value),
    Kendall_W = round4(kendall_w),
    Note = "Main paired analysis by question"
  )
}

run_pairwise_wilcox_one <- function(df, metric_name) {
  metric_df <- df %>%
    dplyr::filter(metric == metric_name) %>%
    dplyr::select(question, model, score) %>%
    dplyr::distinct(question, model, .keep_all = TRUE) %>%
    tidyr::pivot_wider(names_from = model, values_from = score)

  model_cols <- intersect(models_present, names(metric_df))
  if (length(model_cols) < 2) return(empty_df("Fewer than two models available"))

  pairs <- t(combn(model_cols, 2))
  res <- purrr::map_dfr(seq_len(nrow(pairs)), function(i) {
    m1 <- pairs[i, 1]
    m2 <- pairs[i, 2]
    tmp <- metric_df %>%
      dplyr::select(question, dplyr::all_of(c(m1, m2))) %>%
      tidyr::drop_na()

    if (nrow(tmp) < 2) {
      return(data.frame(
        Metric = metric_name, Model_1 = m1, Model_2 = m2,
        N_pairs = nrow(tmp), Mean_difference = NA,
        Median_difference = NA, W_statistic = NA,
        P_unadjusted = NA, Note = "Fewer than two complete paired observations"
      ))
    }

    x <- tmp[[m1]]
    y <- tmp[[m2]]
    diff_xy <- x - y

    if (length(unique(diff_xy)) < 2 || all(diff_xy == 0, na.rm = TRUE)) {
      return(data.frame(
        Metric = metric_name, Model_1 = m1, Model_2 = m2,
        N_pairs = nrow(tmp),
        Mean_difference = round4(mean(diff_xy, na.rm = TRUE)),
        Median_difference = round4(median(diff_xy, na.rm = TRUE)),
        W_statistic = NA,
        P_unadjusted = NA,
        Note = "All paired differences are identical or zero; Wilcoxon test not applicable"
      ))
    }

    wt <- tryCatch(
      wilcox.test(x, y, paired = TRUE, exact = FALSE, correct = TRUE),
      error = function(e) NULL
    )

    data.frame(
      Metric = metric_name,
      Model_1 = m1,
      Model_2 = m2,
      N_pairs = nrow(tmp),
      Mean_difference = round4(mean(diff_xy, na.rm = TRUE)),
      Median_difference = round4(median(diff_xy, na.rm = TRUE)),
      W_statistic = ifelse(is.null(wt), NA, round4(wt$statistic)),
      P_unadjusted = ifelse(is.null(wt), NA, round4(wt$p.value)),
      Note = ifelse(is.null(wt), "Wilcoxon test failed", "Paired Wilcoxon signed-rank test")
    )
  })

  res %>%
    dplyr::group_by(Metric) %>%
    dplyr::mutate(
      P_adjusted_BH = round4(p.adjust(P_unadjusted, method = "BH")),
      P_adjusted_formatted = fmt_p(P_adjusted_BH)
    ) %>%
    dplyr::ungroup()
}

non_safety_metrics <- setdiff(all_metrics, "Safety")

friedman_results <- purrr::map_dfr(non_safety_metrics, ~ run_friedman_one(data, .x))
if (!"P_value_formatted" %in% names(friedman_results)) friedman_results$P_value_formatted <- fmt_p(friedman_results$P_value)
pairwise_wilcox_results <- purrr::map_dfr(non_safety_metrics, ~ run_pairwise_wilcox_one(data, .x))

significant_pairwise_wilcox <- safe_filter_sig(pairwise_wilcox_results, "P_adjusted_BH", 0.05)

# ---------- 7. Safety：二分类配对分析 ----------
run_cochran_q <- function(df) {
  metric_df <- df %>%
    dplyr::filter(metric == "Safety") %>%
    dplyr::select(question, model, score) %>%
    dplyr::distinct(question, model, .keep_all = TRUE) %>%
    tidyr::pivot_wider(names_from = model, values_from = score)

  model_cols <- intersect(models_present, names(metric_df))
  if (length(model_cols) < 2) {
    return(data.frame(
      Metric = "Safety", Test = "Cochran Q test", N_questions = 0,
      N_models = length(model_cols), Statistic = NA, DF = NA, P_value = NA,
      P_value_formatted = NA, Note = "Fewer than two models available"
    ))
  }

  metric_complete <- metric_df %>% tidyr::drop_na(dplyr::all_of(model_cols))
  if (nrow(metric_complete) < 2) {
    return(data.frame(
      Metric = "Safety", Test = "Cochran Q test", N_questions = nrow(metric_complete),
      N_models = length(model_cols), Statistic = NA, DF = NA, P_value = NA,
      P_value_formatted = NA, Note = "Fewer than two complete paired questions"
    ))
  }

  mat <- as.matrix(metric_complete[, model_cols])
  if (!all(mat %in% c(0, 1))) {
    return(data.frame(
      Metric = "Safety", Test = "Cochran Q test", N_questions = nrow(metric_complete),
      N_models = length(model_cols), Statistic = NA, DF = NA, P_value = NA,
      P_value_formatted = NA, Note = "Safety contains values outside 0/1"
    ))
  }

  k <- ncol(mat)
  n <- nrow(mat)
  col_sum <- colSums(mat)
  row_sum <- rowSums(mat)
  total <- sum(mat)
  denom <- k * total - sum(row_sum^2)

  if (denom <= 0 || length(unique(as.vector(mat))) < 2) {
    return(data.frame(
      Metric = "Safety", Test = "Cochran Q test", N_questions = n,
      N_models = k, Statistic = NA, DF = k - 1, P_value = NA,
      P_value_formatted = NA, Note = "No binary variation or no discordance; test not applicable"
    ))
  }

  q_stat <- (k - 1) * (k * sum(col_sum^2) - total^2) / denom
  p_val <- pchisq(q_stat, df = k - 1, lower.tail = FALSE)

  data.frame(
    Metric = "Safety",
    Test = "Cochran Q test",
    N_questions = n,
    N_models = k,
    Statistic = round4(q_stat),
    DF = k - 1,
    P_value = round4(p_val),
    P_value_formatted = fmt_p(p_val),
    Note = paste0("Main paired binary analysis; Safety: ", safety_safe_value, "=safe, ", safety_unsafe_value, "=unsafe")
  )
}

run_pairwise_mcnemar <- function(df) {
  metric_df <- df %>%
    dplyr::filter(metric == "Safety") %>%
    dplyr::select(question, model, score) %>%
    dplyr::distinct(question, model, .keep_all = TRUE) %>%
    tidyr::pivot_wider(names_from = model, values_from = score)

  model_cols <- intersect(models_present, names(metric_df))
  if (length(model_cols) < 2) return(empty_df("Fewer than two models available"))

  pairs <- t(combn(model_cols, 2))
  res <- purrr::map_dfr(seq_len(nrow(pairs)), function(i) {
    m1 <- pairs[i, 1]
    m2 <- pairs[i, 2]
    tmp <- metric_df %>%
      dplyr::select(question, dplyr::all_of(c(m1, m2))) %>%
      tidyr::drop_na()

    if (nrow(tmp) < 2) {
      return(data.frame(
        Metric = "Safety", Model_1 = m1, Model_2 = m2,
        N_pairs = nrow(tmp), Safe_rate_difference_percent = NA,
        Safe_to_unsafe = NA, Unsafe_to_safe = NA,
        Chi_square = NA, P_unadjusted = NA,
        Note = "Fewer than two complete paired observations"
      ))
    }

    x <- tmp[[m1]]
    y <- tmp[[m2]]
    # Safety coding is controlled by safety_safe_value and safety_unsafe_value.
    safe_to_unsafe_n <- sum(x == safety_safe_value & y == safety_unsafe_value, na.rm = TRUE)
    unsafe_to_safe_n <- sum(x == safety_unsafe_value & y == safety_safe_value, na.rm = TRUE)

    if (!all(x %in% c(0, 1)) || !all(y %in% c(0, 1))) {
      return(data.frame(
        Metric = "Safety", Model_1 = m1, Model_2 = m2,
        N_pairs = nrow(tmp), Safe_rate_difference_percent = NA,
        Safe_to_unsafe = safe_to_unsafe_n, Unsafe_to_safe = unsafe_to_safe_n,
        Chi_square = NA, P_unadjusted = NA,
        Note = "Safety contains values outside 0/1"
      ))
    }

    safe_rate_diff <- 100 * (mean(x == safety_safe_value, na.rm = TRUE) - mean(y == safety_safe_value, na.rm = TRUE))

    if ((safe_to_unsafe_n + unsafe_to_safe_n) == 0) {
      return(data.frame(
        Metric = "Safety", Model_1 = m1, Model_2 = m2,
        N_pairs = nrow(tmp),
        Safe_rate_difference_percent = round4(safe_rate_diff),
        Safe_to_unsafe = safe_to_unsafe_n,
        Unsafe_to_safe = unsafe_to_safe_n,
        Chi_square = NA,
        P_unadjusted = NA,
        Note = "No discordant pairs; McNemar test not applicable"
      ))
    }

    tb <- table(
      factor(x, levels = c(0, 1)),
      factor(y, levels = c(0, 1))
    )
    mt <- tryCatch(mcnemar.test(tb, correct = TRUE), error = function(e) NULL)

    data.frame(
      Metric = "Safety",
      Model_1 = m1,
      Model_2 = m2,
      N_pairs = nrow(tmp),
      Safe_rate_difference_percent = round4(safe_rate_diff),
      Safe_to_unsafe = safe_to_unsafe_n,
      Unsafe_to_safe = unsafe_to_safe_n,
      Chi_square = ifelse(is.null(mt), NA, round4(mt$statistic)),
      P_unadjusted = ifelse(is.null(mt), NA, round4(mt$p.value)),
      Note = ifelse(is.null(mt), "McNemar test failed", "Pairwise McNemar test")
    )
  })

  res %>%
    dplyr::mutate(
      P_adjusted_BH = round4(p.adjust(P_unadjusted, method = "BH")),
      P_adjusted_formatted = fmt_p(P_adjusted_BH)
    )
}

cochran_q_result <- run_cochran_q(data)
pairwise_mcnemar_results <- run_pairwise_mcnemar(data)

significant_pairwise_mcnemar <- safe_filter_sig(pairwise_mcnemar_results, "P_adjusted_BH", 0.05)

# ---------- 8. 兼容原代码：独立样本 ANOVA/Tukey 与 Kruskal-Dunn ----------
run_anova_one <- function(df, metric_name) {
  metric_df <- df %>%
    dplyr::filter(metric == metric_name) %>%
    dplyr::mutate(model = factor(model, levels = models_present))

  if (nrow(metric_df) < 3 || dplyr::n_distinct(metric_df$model) < 2 || dplyr::n_distinct(metric_df$score) < 2) {
    return(data.frame(
      Metric = metric_name, Test = "One-way ANOVA", F_value = NA,
      P_value = NA, P_value_formatted = NA, DF_model = NA,
      DF_residual = NA, Eta_squared = NA,
      Note = "Insufficient variation; ANOVA not applicable"
    ))
  }

  fit <- tryCatch(aov(score ~ model, data = metric_df), error = function(e) NULL)
  if (is.null(fit)) {
    return(data.frame(
      Metric = metric_name, Test = "One-way ANOVA", F_value = NA,
      P_value = NA, P_value_formatted = NA, DF_model = NA,
      DF_residual = NA, Eta_squared = NA,
      Note = "ANOVA failed"
    ))
  }

  sm <- summary(fit)[[1]]
  ss_model <- sm["model", "Sum Sq"]
  ss_total <- sum(sm[, "Sum Sq"], na.rm = TRUE)
  eta_sq <- ss_model / ss_total

  data.frame(
    Metric = metric_name,
    Test = "One-way ANOVA; sensitivity/legacy analysis",
    F_value = round4(sm["model", "F value"]),
    P_value = round4(sm["model", "Pr(>F)"]),
    P_value_formatted = fmt_p(sm["model", "Pr(>F)"]),
    DF_model = sm["model", "Df"],
    DF_residual = sm["Residuals", "Df"],
    Eta_squared = round4(eta_sq),
    Note = "Kept for compatibility with previous code; main analysis should be paired"
  )
}

run_tukey_one <- function(df, metric_name) {
  metric_df <- df %>%
    dplyr::filter(metric == metric_name) %>%
    dplyr::mutate(model = factor(model, levels = models_present))

  if (nrow(metric_df) < 3 || dplyr::n_distinct(metric_df$model) < 2 || dplyr::n_distinct(metric_df$score) < 2) {
    return(empty_df(paste0(metric_name, ": Tukey HSD not applicable")))
  }

  fit <- tryCatch(aov(score ~ model, data = metric_df), error = function(e) NULL)
  if (is.null(fit)) return(empty_df(paste0(metric_name, ": ANOVA failed; Tukey HSD not available")))

  tk <- tryCatch(TukeyHSD(fit)$model, error = function(e) NULL)
  if (is.null(tk)) return(empty_df(paste0(metric_name, ": Tukey HSD failed")))

  as.data.frame(tk) %>%
    tibble::rownames_to_column("Comparison") %>%
    dplyr::mutate(
      Metric = metric_name,
      Difference = round4(diff),
      Lower_CI = round4(lwr),
      Upper_CI = round4(upr),
      Adjusted_P = round4(`p adj`),
      Adjusted_P_formatted = fmt_p(`p adj`)
    ) %>%
    dplyr::select(Metric, Comparison, Difference, Lower_CI, Upper_CI, Adjusted_P, Adjusted_P_formatted)
}

run_kruskal_one <- function(df, metric_name) {
  metric_df <- df %>%
    dplyr::filter(metric == metric_name) %>%
    dplyr::mutate(model = factor(model, levels = models_present))

  if (nrow(metric_df) < 3 || dplyr::n_distinct(metric_df$model) < 2 || dplyr::n_distinct(metric_df$score) < 2) {
    return(data.frame(
      Metric = metric_name, Test = "Kruskal-Wallis test",
      H_statistic = NA, DF = NA, P_value = NA,
      P_value_formatted = NA, Epsilon_squared = NA,
      Note = "Insufficient variation; Kruskal-Wallis not applicable"
    ))
  }

  kt <- tryCatch(kruskal.test(score ~ model, data = metric_df), error = function(e) NULL)
  if (is.null(kt)) {
    return(data.frame(
      Metric = metric_name, Test = "Kruskal-Wallis test",
      H_statistic = NA, DF = NA, P_value = NA,
      P_value_formatted = NA, Epsilon_squared = NA,
      Note = "Kruskal-Wallis failed"
    ))
  }

  n <- nrow(metric_df)
  k <- dplyr::n_distinct(metric_df$model)
  eps <- (as.numeric(kt$statistic) - k + 1) / (n - k)
  eps <- max(0, min(1, eps))

  data.frame(
    Metric = metric_name,
    Test = "Kruskal-Wallis; sensitivity/legacy analysis",
    H_statistic = round4(kt$statistic),
    DF = as.numeric(kt$parameter),
    P_value = round4(kt$p.value),
    P_value_formatted = fmt_p(kt$p.value),
    Epsilon_squared = round4(eps),
    Note = "Kept for compatibility with previous code; main analysis should be paired"
  )
}

run_dunn_one <- function(df, metric_name) {
  metric_df <- df %>%
    dplyr::filter(metric == metric_name) %>%
    dplyr::mutate(model = factor(model, levels = models_present))

  if (nrow(metric_df) < 3 || dplyr::n_distinct(metric_df$model) < 2 || dplyr::n_distinct(metric_df$score) < 2) {
    return(empty_df(paste0(metric_name, ": Dunn test not applicable")))
  }

  dt <- tryCatch(FSA::dunnTest(score ~ model, data = metric_df, method = "bh")$res, error = function(e) NULL)
  if (is.null(dt)) return(empty_df(paste0(metric_name, ": Dunn test failed")))

  dt %>%
    dplyr::mutate(
      Metric = metric_name,
      Z_value = round4(Z),
      Unadjusted_P = round4(P.unadj),
      Adjusted_P = round4(P.adj),
      Adjusted_P_formatted = fmt_p(P.adj)
    ) %>%
    dplyr::select(Metric, Comparison, Z_value, Unadjusted_P, Adjusted_P, Adjusted_P_formatted)
}

legacy_metrics <- setdiff(all_metrics, "Safety")

anova_results <- purrr::map_dfr(legacy_metrics, ~ run_anova_one(data, .x))
tukey_results <- purrr::map_dfr(legacy_metrics, ~ run_tukey_one(data, .x))
kruskal_results <- purrr::map_dfr(legacy_metrics, ~ run_kruskal_one(data, .x))
dunn_results <- purrr::map_dfr(legacy_metrics, ~ run_dunn_one(data, .x))

significant_tukey <- safe_filter_sig(tukey_results, "Adjusted_P", 0.05)

significant_dunn <- safe_filter_sig(dunn_results, "Adjusted_P", 0.05)

# ---------- 9. 论文表格格式 ----------
paper_descriptive_table <- descriptive_all %>%
  dplyr::mutate(
    `Mean ± SD` = sprintf("%.2f ± %.2f", mean, sd),
    `Median [Q1, Q3]` = sprintf("%.2f [%.2f, %.2f]", median, q1, q3),
    Summary = paste0(`Mean ± SD`, "; ", `Median [Q1, Q3]`)
  ) %>%
  dplyr::select(metric_group, metric, model, Summary) %>%
  tidyr::pivot_wider(names_from = model, values_from = Summary) %>%
  dplyr::arrange(metric_group, metric)

paper_test_table_non_safety <- friedman_results %>%
  dplyr::select(
    Metric, Test, N_questions, N_models, Statistic, DF,
    P_value_formatted, Kendall_W, Note
  )

paper_test_table_safety <- cochran_q_result %>%
  dplyr::transmute(
    Metric, Test, N_questions, N_models, Statistic, DF,
    P_value_formatted, Kendall_W = NA_real_, Note
  )

paper_test_table <- dplyr::bind_rows(paper_test_table_non_safety, paper_test_table_safety)

paper_safety_table <- safety_rate %>%
  dplyr::mutate(
    Safety_summary = paste0(safe_n, "/", n, " (", safe_rate_percent, "%)")
  ) %>%
  dplyr::select(model, n, safe_n, unsafe_n, safe_rate_percent, unsafe_rate_percent, Safety_summary)

readme <- data.frame(
  Section = c(
    "Data structure",
    "Main analysis",
    "Safety analysis",
    "Rater agreement",
    "Automatic model detection",
    "Automatic rater detection",
    "Legacy analysis",
    "Score coding",
    "Model name correction"
  ),
  Explanation = c(
    "Data should be long format: question, model, metric, score, with optional rater columns named rater1, rater2, and so on.",
    "For non-safety metrics, models are compared using Friedman tests by question, with pairwise paired Wilcoxon signed-rank tests and BH correction.",
    paste0("Safety is coded as ", safety_safe_value, "=safe and ", safety_unsafe_value, "=unsafe. It is summarized as safe response rate and compared using Cochran Q test, with pairwise McNemar tests."),
    "Agreement is automatically calculated for Safety, Accuracy, Empathy, DISCERN, EQIP, JAMA and GQS. Safety uses Fleiss' kappa with bootstrap 95% CI; other metrics use ICC(2,1) with 95% CI.",
    "Models are automatically identified from the model column. The color palette is generated automatically with redundant reserved colors.",
    paste0("Detected ", rater_count, " rater column(s): ", ifelse(rater_count > 0, paste(rater_cols, collapse = "; "), "none"), "."),
    "ANOVA/Tukey and Kruskal-Dunn are retained only for compatibility with the previous scripts; because the same questions are answered by all models, the paired analysis should be treated as primary.",
    paste0("Accuracy and Empathy are treated as 1-5 ordinal scores. Safety is treated as a binary outcome (", safety_safe_value, "=safe, ", safety_unsafe_value, "=unsafe)."),
    "Model names are automatically read from the model column without predefined model names or version correction."
  )
)

# ---------- 10. 写入 Excel ----------
wb <- createWorkbook()

add_sheet(wb, "00_README", readme)
add_sheet(wb, "01_data_check_summary", data_check_summary)
add_sheet(wb, "02_coverage", coverage)
add_sheet(wb, "03_duplicates", duplicates)
add_sheet(wb, "04_missing_combinations", missing_combinations)
add_sheet(wb, "05_score_range_check", score_range_check)
# 按文章结果顺序组织主要结果：
# 安全性 -> 准确性/同理心 -> 可靠性 -> 可读性
add_sheet(wb, "06_safety_rate", safety_rate)
add_sheet(wb, "07_safety_cochranQ", cochran_q_result)
add_sheet(wb, "08_safety_mcnemar", pairwise_mcnemar_results)
add_sheet(wb, "09_descriptive_all", descriptive_all)
add_sheet(wb, "10_normality", normality_results)
add_sheet(wb, "11_rater_agreement", rater_agreement_results)
add_sheet(wb, "11_main_friedman", friedman_results)
add_sheet(wb, "12_pairwise_wilcox", pairwise_wilcox_results)
add_sheet(wb, "13_sig_safety_mcnemar", significant_pairwise_mcnemar)
add_sheet(wb, "14_sig_pairwise_wilcox", significant_pairwise_wilcox)
add_sheet(wb, "15_paper_safety", paper_safety_table)
add_sheet(wb, "16_paper_descriptive", paper_descriptive_table)
add_sheet(wb, "17_paper_tests", paper_test_table)
add_sheet(wb, "18_legacy_anova", anova_results)
add_sheet(wb, "19_legacy_tukey", tukey_results)
add_sheet(wb, "20_legacy_kruskal", kruskal_results)
add_sheet(wb, "21_legacy_dunn", dunn_results)
add_sheet(wb, "22_sig_tukey", significant_tukey)
add_sheet(wb, "23_sig_dunn", significant_dunn)

excel_output <- file.path(output_dir, "LLM完整统计分析结果.xlsx")
saveWorkbook(wb, excel_output, overwrite = TRUE)

# ---------- 10B. 导出论文可用 Word 表格 ----------
# 说明：
# 1) 该部分只调用前面已经计算好的统计结果，不改变任何统计分析结果。
# 2) Word 表格按论文结果顺序组织：Safety -> Accuracy/Empathy -> Reliability/quality -> Readability。
# 3) Safety 编码由 safety_safe_value 和 safety_unsafe_value 控制；当前为 0=安全，1=不安全。

word_output <- file.path(output_dir, "LLM论文可用结果表.docx")

format_num2 <- function(x) {
  ifelse(is.na(x), "", sprintf("%.2f", as.numeric(x)))
}

format_num3 <- function(x) {
  ifelse(is.na(x), "", sprintf("%.3f", as.numeric(x)))
}

format_summary_cell <- function(mean, sd, median, q1, q3) {
  ifelse(
    is.na(mean),
    "",
    paste0(
      sprintf("%.2f \u00B1 %.2f", mean, sd),
      "; ",
      sprintf("%.2f [%.2f, %.2f]", median, q1, q3)
    )
  )
}

make_paper_metric_table <- function(metrics_vec) {
  desc_wide <- descriptive_all %>%
    dplyr::filter(as.character(metric) %in% metrics_vec) %>%
    dplyr::mutate(
      Metric = as.character(metric),
      model = as.character(model),
      Summary = format_summary_cell(mean, sd, median, q1, q3)
    ) %>%
    dplyr::select(Metric, model, Summary) %>%
    tidyr::pivot_wider(names_from = model, values_from = Summary)

  for (m in models_present) {
    if (!m %in% names(desc_wide)) desc_wide[[m]] <- ""
  }

  test_part <- friedman_results %>%
    dplyr::filter(Metric %in% metrics_vec) %>%
    dplyr::transmute(
      Metric,
      `Overall P` = P_value_formatted,
      `Kendall's W` = format_num3(Kendall_W)
    )

  out <- desc_wide %>%
    dplyr::left_join(test_part, by = "Metric") %>%
    dplyr::select(Metric, dplyr::all_of(models_present), `Overall P`, `Kendall's W`)

  out
}

make_safety_summary_table <- function() {
  safety_rate %>%
    dplyr::mutate(
      Model = as.character(model),
      `Safe responses, n (%)` = paste0(safe_n, " (", sprintf("%.1f", safe_rate_percent), ")"),
      `Unsafe responses, n (%)` = paste0(unsafe_n, " (", sprintf("%.1f", unsafe_rate_percent), ")")
    ) %>%
    dplyr::select(
      Model,
      N = n,
      `Safe responses, n (%)`,
      `Unsafe responses, n (%)`
    )
}

make_safety_overall_test_table <- function() {
  cochran_q_result %>%
    dplyr::transmute(
      Test,
      `No. of questions` = N_questions,
      `No. of models` = N_models,
      Statistic = format_num3(Statistic),
      DF,
      `P value` = P_value_formatted
    )
}

make_rater_agreement_table <- function() {
  need_cols <- c("Metric", "Agreement_index", "Estimate", "Lower_95_CI", "Upper_95_CI", "N_complete_items", "N_raters")
  if (!has_cols(rater_agreement_results, need_cols)) {
    return(data.frame(Note = "No rater agreement result available."))
  }

  rater_agreement_results %>%
    dplyr::transmute(
      Metric,
      `Agreement index` = Agreement_index,
      Estimate = format_num3(Estimate),
      `95% CI` = dplyr::if_else(
        is.na(Lower_95_CI) | is.na(Upper_95_CI),
        "",
        paste0(format_num3(Lower_95_CI), "-", format_num3(Upper_95_CI))
      ),
      `P value` = P_value_formatted,
      `Complete items` = N_complete_items,
      `No. of raters` = N_raters,
      Note
    )
}

make_safety_pairwise_table <- function() {
  need_cols <- c(
    "Model_1", "Model_2", "N_pairs", "Safe_rate_difference_percent",
    "Safe_to_unsafe", "Unsafe_to_safe", "P_adjusted_formatted", "Note"
  )
  if (!has_cols(pairwise_mcnemar_results, need_cols)) {
    return(data.frame(Note = "No pairwise McNemar result available."))
  }

  out <- pairwise_mcnemar_results %>%
    dplyr::transmute(
      Comparison = paste(Model_1, "vs", Model_2),
      `N pairs` = N_pairs,
      `Safe rate difference, %` = format_num2(Safe_rate_difference_percent),
      `Safe to unsafe, n` = Safe_to_unsafe,
      `Unsafe to safe, n` = Unsafe_to_safe,
      `Adjusted P` = P_adjusted_formatted,
      Note
    )
  if (nrow(out) == 0) out <- data.frame(Note = "No pairwise McNemar result available.")
  out
}

make_significant_pairwise_table <- function() {
  need_cols <- c("Metric", "Model_1", "Model_2", "N_pairs", "Mean_difference", "Median_difference", "P_adjusted_formatted")
  if (!has_cols(significant_pairwise_wilcox, need_cols)) {
    return(data.frame(Note = "No significant pairwise difference after Benjamini-Hochberg correction."))
  }

  out <- significant_pairwise_wilcox %>%
    dplyr::transmute(
      Metric,
      Comparison = paste(Model_1, "vs", Model_2),
      `N pairs` = N_pairs,
      `Mean difference` = format_num2(Mean_difference),
      `Median difference` = format_num2(Median_difference),
      `Adjusted P` = P_adjusted_formatted
    )
  if (nrow(out) == 0) out <- data.frame(Note = "No significant pairwise difference after Benjamini-Hochberg correction.")
  out
}

make_significant_safety_pairwise_table <- function() {
  need_cols <- c("Model_1", "Model_2", "N_pairs", "Safe_rate_difference_percent", "Safe_to_unsafe", "Unsafe_to_safe", "P_adjusted_formatted")
  if (!has_cols(significant_pairwise_mcnemar, need_cols)) {
    return(data.frame(Note = "No significant pairwise safety difference after Benjamini-Hochberg correction."))
  }

  out <- significant_pairwise_mcnemar %>%
    dplyr::transmute(
      Comparison = paste(Model_1, "vs", Model_2),
      `N pairs` = N_pairs,
      `Safe rate difference, %` = format_num2(Safe_rate_difference_percent),
      `Safe to unsafe, n` = Safe_to_unsafe,
      `Unsafe to safe, n` = Unsafe_to_safe,
      `Adjusted P` = P_adjusted_formatted
    )
  if (nrow(out) == 0) out <- data.frame(Note = "No significant pairwise safety difference after Benjamini-Hochberg correction.")
  out
}

make_all_pairwise_nonbinary_table <- function() {
  need_cols <- c("Metric", "Model_1", "Model_2", "N_pairs", "Mean_difference", "Median_difference", "P_adjusted_formatted", "Note")
  if (!has_cols(pairwise_wilcox_results, need_cols)) {
    return(data.frame(Note = "No pairwise Wilcoxon result available."))
  }

  out <- pairwise_wilcox_results %>%
    dplyr::transmute(
      Metric,
      Comparison = paste(Model_1, "vs", Model_2),
      `N pairs` = N_pairs,
      `Mean difference` = format_num2(Mean_difference),
      `Median difference` = format_num2(Median_difference),
      `Adjusted P` = P_adjusted_formatted,
      Note
    )
  if (nrow(out) == 0) out <- data.frame(Note = "No pairwise Wilcoxon result available.")
  out
}

add_word_table <- function(doc, title, df, note = NULL, font_size = 8) {
  doc <- officer::body_add_par(doc, title, style = "heading 2")

  ft <- flextable::flextable(df)
  ft <- flextable::theme_booktabs(ft)
  ft <- flextable::fontsize(ft, size = font_size, part = "all")
  ft <- flextable::bold(ft, part = "header")
  ft <- flextable::align(ft, align = "center", part = "all")
  ft <- flextable::align(ft, j = 1, align = "left", part = "body")
  ft <- flextable::valign(ft, valign = "center", part = "all")
  ft <- flextable::set_table_properties(ft, layout = "autofit", width = 1)
  ft <- flextable::autofit(ft)

  doc <- flextable::body_add_flextable(doc, ft)

  if (!is.null(note) && nchar(note) > 0) {
    doc <- officer::body_add_par(doc, note, style = "Normal")
  }
  doc <- officer::body_add_par(doc, "", style = "Normal")
  doc
}

# 论文主表
word_table_safety <- make_safety_summary_table()
word_table_safety_test <- make_safety_overall_test_table()
word_table_rater_agreement <- make_rater_agreement_table()
word_table_accuracy_empathy <- make_paper_metric_table(c("Accuracy", "Empathy"))
word_table_reliability <- make_paper_metric_table(reliability_metrics)
word_table_readability <- make_paper_metric_table(readability_metrics)

# 论文补充表
word_table_safety_pairwise <- make_safety_pairwise_table()
word_table_sig_nonbinary <- make_significant_pairwise_table()
word_table_sig_safety <- make_significant_safety_pairwise_table()
word_table_all_pairwise_nonbinary <- make_all_pairwise_nonbinary_table()

doc <- officer::read_docx()

doc <- officer::body_add_par(doc, "Tables for Manuscript", style = "heading 1")
doc <- officer::body_add_par(
  doc,
  "Safety was coded as 0 = safe and 1 = unsafe. For non-binary outcomes, values are presented as mean \u00B1 standard deviation; median [interquartile range]. Overall P values for non-binary outcomes were obtained using Friedman tests, with Kendall's W reported as the effect size. Safety was compared using Cochran's Q test. Pairwise P values were adjusted using the Benjamini-Hochberg method.",
  style = "Normal"
)

doc <- add_word_table(
  doc,
  "Table 0. Inter-rater agreement",
  word_table_rater_agreement,
  note = "Note: Safety was assessed using Fleiss' kappa. Accuracy, Empathy, DISCERN, EQIP, JAMA and GQS were assessed using ICC(2,1).",
  font_size = 8
)

doc <- add_word_table(
  doc,
  "Table 1. Safety of responses across models",
  word_table_safety,
  note = paste0("Note: Safety was coded as ", safety_safe_value, " = safe and ", safety_unsafe_value, " = unsafe. Safe responses are presented as n (%)."),
  font_size = 9
)

doc <- add_word_table(
  doc,
  "Table 2. Overall comparison of safety across models",
  word_table_safety_test,
  note = "Note: Overall comparison was performed using Cochran's Q test because Safety was a binary paired outcome.",
  font_size = 9
)

doc <- add_word_table(
  doc,
  "Table 3. Accuracy and empathy scores across models",
  word_table_accuracy_empathy,
  note = "Note: Values are mean \u00B1 SD; median [Q1, Q3]. Overall P values were obtained using Friedman tests.",
  font_size = 8
)

doc <- add_word_table(
  doc,
  "Table 4. Reliability and quality scores across models",
  word_table_reliability,
  note = "Note: Values are mean \u00B1 SD; median [Q1, Q3]. Overall P values were obtained using Friedman tests.",
  font_size = 8
)

doc <- add_word_table(
  doc,
  "Table 5. Readability scores across models",
  word_table_readability,
  note = "Note: Values are mean \u00B1 SD; median [Q1, Q3]. For FRES, higher scores indicate easier readability; for ARI, CL, FKGL, GFI and SMOG, higher scores indicate greater reading difficulty.",
  font_size = 8
)

doc <- officer::body_add_break(doc)

doc <- officer::body_add_par(doc, "Supplementary Tables", style = "heading 1")

doc <- add_word_table(
  doc,
  "Supplementary Table 1. Pairwise comparisons of safety",
  word_table_safety_pairwise,
  note = "Note: Pairwise comparisons were performed using McNemar tests with Benjamini-Hochberg correction.",
  font_size = 8
)

doc <- add_word_table(
  doc,
  "Supplementary Table 2. Significant pairwise comparisons for non-binary outcomes",
  word_table_sig_nonbinary,
  note = "Note: Pairwise comparisons were performed using paired Wilcoxon signed-rank tests with Benjamini-Hochberg correction. Only significant comparisons are shown.",
  font_size = 8
)

# The redundant table "Significant pairwise comparisons of safety" was not exported
# because Appendix 5 already reports all pairwise safety comparisons in Table S2 and no significant
# safety pairwise differences were observed after Benjamini-Hochberg correction. This prevents
# duplicate Table S4 numbering and keeps Table S3/S4 citations consistent.

doc <- add_word_table(
  doc,
  "Table S4. All pairwise comparisons for non-binary outcomes",
  word_table_all_pairwise_nonbinary,
  note = "Note: Pairwise comparisons were performed using paired Wilcoxon signed-rank tests with Benjamini-Hochberg correction.",
  font_size = 7
)

print(doc, target = word_output)

# ---------- 11. 作图：柱状图（仅输出3张水平合并图，单一共享图例） ----------
# sci_colors 已根据数据中的模型名称自动生成；无需手动修改模型列表或颜色。

# 单图主题：保留图例，供 patchwork 合并后统一收集
pub_theme <- theme_minimal() +
  theme(
    panel.grid = element_blank(),
    axis.line = element_line(color = "black", linewidth = 0.5),
    axis.ticks = element_line(color = "black", linewidth = 0.5),
    axis.ticks.length = unit(0.2, "cm"),
    plot.title = element_text(hjust = 0.5, size = 18, face = "bold", margin = margin(b = 8)),
    plot.title.position = "panel",
    plot.margin = margin(12, 12, 12, 12),
    axis.title = element_text(size = 16, face = "bold"),
    axis.text.y = element_text(size = 14, color = "black"),
    axis.text.x = element_text(size = 14, color = "black", angle = 45, hjust = 1),
    legend.title = element_blank(),
    legend.position = "bottom",
    legend.justification = "center",
    legend.box = "horizontal",
    legend.direction = "horizontal",
    legend.text = element_text(size = 12)
  )

mean_scores <- data %>%
  dplyr::filter(metric != "Safety") %>%
  dplyr::group_by(metric_group, metric, model) %>%
  dplyr::summarise(mean_score = round(mean(score, na.rm = TRUE), 2), .groups = "drop")

safety_plot_data <- safety_rate %>%
  dplyr::mutate(
    metric = "Safety",
    metric_group = "1. Safety",
    mean_score = safe_rate_percent
  )

get_y_settings <- function(metric_name) {
  if (metric_name %in% c("DISCERN", "EQIP", "FRES")) {
    return(list(limits = c(0, 100), breaks = seq(0, 100, 20), ref = numeric(0), ylab = "Mean Score"))
  }
  if (metric_name %in% c("ARI", "CL", "FKGL", "GFI", "SMOG")) {
    return(list(limits = c(0, 20), breaks = seq(0, 20, 5), ref = 6, ylab = "Mean Score"))
  }
  if (metric_name %in% c("GQS", "JAMA", "Accuracy", "Empathy")) {
    return(list(limits = c(0, 5), breaks = seq(0, 5, 1), ref = numeric(0), ylab = "Mean Score"))
  }
  if (metric_name == "Safety") {
    return(list(limits = c(0, 100), breaks = seq(0, 100, 20), ref = numeric(0), ylab = "Safe responses (%)"))
  }
  list(limits = NULL, breaks = waiver(), ref = numeric(0), ylab = "Score")
}

make_metric_bar <- function(metric_name) {
  ys <- get_y_settings(metric_name)

  if (metric_name == "Safety") {
    plot_df <- safety_plot_data
    y_var <- "mean_score"
    label_text <- "mean_score"
  } else {
    plot_df <- mean_scores %>% dplyr::filter(metric == metric_name)
    y_var <- "mean_score"
    label_text <- "mean_score"
  }

  if (nrow(plot_df) == 0) return(NULL)

  p <- ggplot(plot_df, aes(x = model, y = .data[[y_var]], fill = model)) +
    geom_col(width = 0.7) +
    geom_text(
      aes(label = sprintf("%.2f", .data[[label_text]])),
      vjust = -0.3, size = 5, fontface = "bold"
    ) +
    scale_fill_manual(values = sci_colors, drop = FALSE) +
    guides(
      fill = guide_legend(
        nrow = 1,
        byrow = TRUE,
        override.aes = list(size = 5)
      )
    ) +
    labs(title = metric_name, x = NULL, y = ys$ylab) +
    pub_theme

  if (!is.null(ys$limits)) {
    p <- p + scale_y_continuous(
      limits = ys$limits,
      breaks = ys$breaks,
      expand = expansion(mult = c(0, 0.05))
    )
  }

  if (length(ys$ref) > 0) {
    p <- p + geom_hline(
      yintercept = ys$ref,
      color = "red",
      linetype = "dashed",
      linewidth = 1
    )
  }

  p
}

plot_objects <- list()
plot_metrics <- c("Safety", "Accuracy", "Empathy", reliability_metrics, readability_metrics)

for (m in plot_metrics) {
  p <- make_metric_bar(m)
  if (!is.null(p)) {
    plot_objects[[m]] <- p
  }
}

save_combined_plot <- function(metrics, filename, ncol, width, height) {
  ps <- plot_objects[metrics]
  ps <- ps[!vapply(ps, is.null, logical(1))]
  if (length(ps) == 0) return(NULL)

  combined <- patchwork::wrap_plots(
    plotlist = ps,
    ncol = ncol,
    guides = "collect"
  ) &
    theme(
      legend.position = "bottom",
      legend.justification = "center",
      legend.box = "horizontal",
      legend.direction = "horizontal",
      legend.title = element_blank(),
      legend.text = element_text(size = 12),
      legend.margin = margin(t = 5, r = 0, b = 5, l = 0),
      legend.box.margin = margin(t = 5, r = 0, b = 5, l = 0)
    )

  ggsave(
    filename = file.path(plot_dir, filename),
    plot = combined,
    width = width,
    height = height,
    units = "in",
    dpi = 600,
    compression = "lzw",
    bg = "white"
  )
  combined
}

# 仅输出3张水平合并图：每张图下方只有一套共享图例
# 注意：原来的三张图只输出一次，不再同时保存旧命名和 Figure 命名两套文件。
combined_performance <- save_combined_plot(
  c("Safety", "Accuracy", "Empathy"),
  "Figure1_combined_safety_accuracy_empathy.tif",
  ncol = 3,
  width = 15,
  height = 6.5
)

combined_reliability <- save_combined_plot(
  reliability_metrics,
  "Figure2_combined_reliability_quality.tif",
  ncol = 4,
  width = 20,
  height = 6.5
)

combined_readability <- save_combined_plot(
  readability_metrics,
  "Figure3_combined_readability.tif",
  ncol = 6,
  width = 30,
  height = 6.5
)

# ---------- 12. 新增：与图1、图2、图3一一对应的三张分布展示图 ----------
# 说明：
# 1) 原来的图1、图2、图3继续保留，不删除、不替换。
# 2) 这里额外补做三张“分布展示图”，分别对应原图1、图2、图3。
# 3) 非二分类指标采用“箱线图 + 抖动散点”，便于展示中位数、四分位数、离散程度和异常值。
# 4) Safety 是二分类变量，当前编码为 0=安全、1=不安全，因此用安全/不安全构成比图展示。

paired_distribution_dir <- file.path(plot_dir, "paired_distribution_figures")
if (!dir.exists(paired_distribution_dir)) dir.create(paired_distribution_dir, recursive = TRUE)

dist_theme <- theme_minimal() +
  theme(
    panel.grid.minor = element_blank(),
    panel.grid.major.x = element_blank(),
    axis.line = element_line(color = "black", linewidth = 0.5),
    axis.ticks = element_line(color = "black", linewidth = 0.4),
    strip.text = element_text(size = 13, face = "bold"),
    axis.title = element_text(size = 14, face = "bold"),
    axis.text.x = element_text(size = 11, color = "black", angle = 45, hjust = 1),
    axis.text.y = element_text(size = 11, color = "black"),
    legend.title = element_blank(),
    legend.text = element_text(size = 11),
    plot.title = element_text(size = 16, face = "bold", hjust = 0.5),
    plot.margin = margin(10, 10, 10, 10)
  )

make_box_jitter_distribution <- function(metrics_vec, title_text, fixed_y_limits = NULL, fixed_y_breaks = waiver(), facet_scales = "free_y") {
  plot_df <- data %>%
    dplyr::filter(as.character(metric) %in% metrics_vec, metric != "Safety") %>%
    dplyr::mutate(
      metric = factor(as.character(metric), levels = metrics_vec),
      model = factor(as.character(model), levels = models_present)
    )

  if (nrow(plot_df) == 0) return(NULL)

  p <- ggplot(plot_df, aes(x = model, y = score, fill = model, color = model)) +
    geom_boxplot(
      width = 0.55,
      alpha = 0.45,
      outlier.shape = NA,
      linewidth = 0.55,
      show.legend = TRUE,
      key_glyph = "rect"
    ) +
    geom_jitter(
      width = 0.14,
      alpha = 0.60,
      size = 1.7,
      show.legend = FALSE
    ) +
    stat_summary(
      fun = median,
      geom = "point",
      shape = 23,
      size = 2.3,
      fill = "white",
      color = "black",
      show.legend = FALSE
    ) +
    scale_fill_manual(values = sci_colors, drop = FALSE, name = NULL) +
    scale_color_manual(values = sci_colors, drop = FALSE, guide = "none") +
    facet_wrap(~ metric, scales = facet_scales, nrow = 1) +
    labs(
      title = title_text,
      x = NULL,
      y = "Score"
    ) +
    guides(
      fill = guide_legend(
        nrow = 1,
        byrow = TRUE,
        override.aes = list(
          shape = 22,
          size = 3.2,
          alpha = 1,
          color = NA,
          linetype = 0
        )
      )
    ) +
    dist_theme +
    theme(
      legend.position = "bottom",
      legend.direction = "horizontal",
      legend.justification = "center",
      legend.box = "horizontal",
      legend.key.width = unit(0.45, "cm"),
      legend.key.height = unit(0.30, "cm"),
      legend.text = element_text(size = 10),
      legend.spacing.x = unit(0.15, "cm")
    )

  # For the manuscript Figure 2 Accuracy/Empathy distribution panels,
  # use the full 1-5 score range to avoid visually exaggerating small differences.
  if (!is.null(fixed_y_limits)) {
    p <- p + scale_y_continuous(
      limits = fixed_y_limits,
      breaks = fixed_y_breaks,
      expand = expansion(mult = c(0.02, 0.05))
    )
  }

  # 可读性指标增加参考线：ARI/CL/FKGL/GFI/SMOG = 6；FRES = 80。
  ref_metrics <- intersect(metrics_vec, readability_metrics)
  if (length(ref_metrics) > 0) {
    ref_df <- data.frame(
      metric = factor(ref_metrics, levels = metrics_vec),
      # 参考线：ARI/CL/FKGL/GFI/SMOG 设为6；FRES为0-100分量表，参考线设为80。
      yintercept = ifelse(ref_metrics == "FRES", 80, 6)
    )

    p <- p +
      geom_hline(
        data = ref_df,
        aes(yintercept = yintercept),
        inherit.aes = FALSE,
        linetype = "dashed",
        linewidth = 0.7,
        color = "red"
      )
  }

  p
}

make_safety_composition_plot <- function() {
  if (!"Safety" %in% unique(as.character(data$metric))) return(NULL)

  # Safety 编码由 safety_safe_value 和 safety_unsafe_value 控制。
  # 这里先按模型和安全状态计数，再在每个模型内部计算百分比。
  # 因此每个模型的 Unsafe% + Safe% 一定等于 100%。
  safety_distribution_df <- data %>%
    dplyr::filter(as.character(metric) == "Safety") %>%
    dplyr::mutate(
      model = factor(as.character(model), levels = models_present),
      score = as.numeric(score),
      Safety_status = dplyr::case_when(
        score == safety_unsafe_value ~ "Unsafe",
        score == safety_safe_value ~ "Safe",
        TRUE ~ NA_character_
      ),
      Safety_status = factor(Safety_status, levels = c("Unsafe", "Safe"))
    ) %>%
    dplyr::filter(!is.na(Safety_status)) %>%
    dplyr::count(model, Safety_status, name = "n") %>%
    tidyr::complete(
      model = factor(models_present, levels = models_present),
      Safety_status = factor(c("Unsafe", "Safe"), levels = c("Unsafe", "Safe")),
      fill = list(n = 0)
    ) %>%
    dplyr::group_by(model) %>%
    dplyr::mutate(
      total_n = sum(n, na.rm = TRUE),
      percent = dplyr::if_else(total_n > 0, 100 * n / total_n, 0),
      label = dplyr::if_else(
        n > 0,
        paste0(n, " (", sprintf("%.1f", percent), "%)"),
        ""
      )
    ) %>%
    dplyr::ungroup()

  p <- ggplot(
    safety_distribution_df,
    aes(x = model, y = percent, fill = Safety_status)
  ) +
    geom_col(width = 0.65, color = "white", linewidth = 0.4) +
    geom_text(
      aes(label = label),
      position = position_stack(vjust = 0.5),
      size = 3.0,
      fontface = "bold"
    ) +
    scale_fill_manual(
      values = c("Unsafe" = "#D73027", "Safe" = "#1A9850"),
      breaks = c("Unsafe", "Safe"),
      drop = FALSE
    ) +
    scale_y_continuous(
      limits = c(0, 100),
      breaks = seq(0, 100, 20),
      expand = expansion(mult = c(0, 0.02))
    ) +
    labs(
      title = "Safety",
      x = NULL,
      y = "Percentage of responses (%)"
    ) +
    guides(
      fill = guide_legend(
        nrow = 1,
        byrow = TRUE,
        override.aes = list(
          shape = 22,
          size = 3.2,
          alpha = 1,
          color = NA,
          linetype = 0
        )
      )
    ) +
    dist_theme +
    theme(
      legend.position = "bottom",
      legend.direction = "horizontal",
      legend.key.width = unit(0.45, "cm"),
      legend.key.height = unit(0.30, "cm"),
      legend.text = element_text(size = 10),
      legend.spacing.x = unit(0.15, "cm")
    )

  p
}

# 与图1对应：Safety + Accuracy + Empathy 的分布展示图
safety_dist_plot <- make_safety_composition_plot()
accuracy_empathy_dist_plot <- make_box_jitter_distribution(
  metrics_vec = c("Accuracy", "Empathy"),
  title_text = "Accuracy and Empathy",
  fixed_y_limits = c(1, 5),
  fixed_y_breaks = seq(1, 5, 1),
  facet_scales = "fixed"
)

figure1_distribution_list <- list(safety_dist_plot, accuracy_empathy_dist_plot)
figure1_distribution_list <- figure1_distribution_list[!vapply(figure1_distribution_list, is.null, logical(1))]

if (length(figure1_distribution_list) > 0) {
  figure1_distribution <- patchwork::wrap_plots(
    plotlist = figure1_distribution_list,
    nrow = 1
  ) +
    patchwork::plot_annotation(
      title = "Distribution of Safety, Accuracy and Empathy Across Models"
    ) &
    theme(
      plot.title = element_text(size = 18, face = "bold", hjust = 0.5)
    )

  ggsave(
    filename = file.path(paired_distribution_dir, "Figure2_distribution_safety_accuracy_empathy.tif"),
    plot = figure1_distribution,
    width = 16,
    height = 6.5,
    units = "in",
    dpi = 600,
    compression = "lzw",
    bg = "white"
  )
}

# 与图2对应：Reliability/quality 指标的分布展示图
figure2_distribution <- make_box_jitter_distribution(
  metrics_vec = reliability_metrics,
  title_text = "Distribution of Reliability and Quality Scores Across Models"
)

if (!is.null(figure2_distribution)) {
  ggsave(
    filename = file.path(paired_distribution_dir, "Figure3_distribution_reliability_quality.tif"),
    plot = figure2_distribution,
    width = 20,
    height = 6.5,
    units = "in",
    dpi = 600,
    compression = "lzw",
    bg = "white"
  )
}

# 与图3对应：Readability 指标的分布展示图
figure3_distribution <- make_box_jitter_distribution(
  metrics_vec = readability_metrics,
  title_text = "Distribution of Readability Scores Across Models"
)

if (!is.null(figure3_distribution)) {
  ggsave(
    filename = file.path(paired_distribution_dir, "Figure4_distribution_readability.tif"),
    plot = figure3_distribution,
    width = 30,
    height = 6.5,
    units = "in",
    dpi = 600,
    compression = "lzw",
    bg = "white"
  )
}

# ---------- 13. 控制台摘要 ----------
cat("\n==========================================================\n")
cat("分析完成。\n")
cat("Excel结果：", excel_output, "\n")
cat("Word论文表格：", word_output, "\n")
cat("图片目录：", plot_dir, "\n")
cat("新增三张对应分布图目录：", paired_distribution_dir, "\n")
cat("柱状图保留3张水平合并图且只输出一次：Figure1、Figure2、Figure3；每张图仅保留1套共享图例，位于下方正中。\n")
cat("同时新增3张对应分布图：Figure2_distribution_safety_accuracy_empathy.tif、Figure3_distribution_reliability_quality.tif、Figure4_distribution_readability.tif。\n")
cat("主分析：非Safety指标使用 Friedman + paired Wilcoxon；Safety使用 Cochran Q + McNemar。\n")
cat("结果和图表顺序：Safety -> Accuracy -> Empathy -> Reliability -> Readability。\n")
cat("Safety编码：", safety_safe_value, "=安全，", safety_unsafe_value, "=不安全。\n", sep = "")
cat("自动识别模型：", paste(models_present, collapse = "; "), "\n")
cat("自动识别评分员列：", ifelse(rater_count > 0, paste(rater_cols, collapse = "; "), "未识别到评分员列"), "\n")
cat("==========================================================\n\n")

cat("数据完整性摘要：\n")
print(data_check_summary)

cat("\n主分析结果：\n")
print(friedman_results)

cat("\nSafety安全率：\n")
print(safety_rate)

cat("\nSafety Cochran Q：\n")
print(cochran_q_result)

cat("\n评分者一致性：\n")
print(rater_agreement_results)
```
