# Supplementary material for: Cross-sectional comparative evaluation of five large language model–driven chatbots on an expert-curated 44-question set of parent-facing questions about pediatric vitamin D deficiency
Source: Front Pediatr. 2026 Sep 9;14:1927624. doi: 10.3389/fped.2026.1927624 (PMC13597792; doi:10.3389/fped.2026.1927624)
Supplement: Supplementary file 7 [file Supplementaryfile8.docx]

**CHART Checklist**

| Heading | No | Chart checklist item | Locator / reported on |
| --- | --- | --- | --- |
| Title and abstract |  |  |  |
| Title | 1a | State that the study is assessing one or more generative AI-driven chatbots for clinical evidence or health advice. | Title |
| Abstract/summary | 1b | Apply a structured format, if applicable. | Abstract |
| Introduction |  |  |  |
| Background | 2a | State the scientific background, rationale, and healthcare context for evaluating the generative AI-driven chatbot(s), referencing relevant literature when applicable. | Introduction |
|  | 2b | State the aims and research questions including the target audience, intervention, comparator(s), and outcome(s). | Introduction |
| Methods |  |  |  |
| Model identifiers | 3a | State the name and version identifier(s) of the generative AI model(s) and chatbot(s) under evaluation, as well as their date of release or last update. | Models under evaluation; Appendix 2, Tables S3/S6 |
|  | 3b | State whether the generative AI model(s) and chatbot(s) are open-source or closed-source/proprietary. | Appendix 2, Table S6 |
| Model details | 4a | State whether the generative AI model was a base model or a novel base model, tuned model, or fine-tuned model. | Models under evaluation |
|  | 4b | If a base model is used, cite its development in sufficient detail to identify the model. | Models under evaluation; Appendix 2, Tables S3/S6 |
|  | 4c | If a novel base model, tuned model, or fine-tuned model is used, describe the pre- and/or post-implementation/deployment data and parameters. | N/A; Models under evaluation |
| Prompt engineering | 5a | Describe the evolution of study prompt development. | Question bank development; Appendix 2 |
|  | 5ai | Describe the sources of prompts. | Question bank development; Appendix 2 |
|  | 5aii | State the number and characteristics of the individual(s) involved in prompt engineering. | Author contributions; Appendix 2 |
|  | 5aiii | Provide details of any patient and public involvement during prompt engineering. | Patient and public involvement |
|  | 5b | Provide study prompts. | Prompting and query strategy; Appendix 2, Table S2 |
| Query strategy | 6a | State route of access to generative AI model. | Models under evaluation; Appendix 2, Table S6 |
|  | 6b | State the date(s) and location(s) of queries for the generative AI-driven chatbot(s) including the day, month, and year as well as city and country. | Models under evaluation; Prompting and query strategy; Appendix 2, Table S6 |
|  | 6c | Describe whether prompts were input into separate chat session(s). | Prompting and query strategy; Appendix 2, Table S6 |
|  | 6d | Provide all generative AI-driven chatbot output/responses | Prompting and query strategy; Appendix 4 |
| Performance evaluation | 7a | Define the ground truth or reference standard used to define successful generative AI-driven chatbot performance. | Reference standard; Appendix 3 |
|  | 7b | Describe the process undertaken for generative AI-driven chatbot performance evaluation. | Performance measures; Raters and scoring process; Appendix 5 |
|  | 7bi | State the number and characteristics of team members involved in performance evaluation. | Raters and scoring process; Appendix 5, Table S1 |
|  | 7bii | Provide details of any patients and public involvement during the evaluation process. | Patient and public involvement |
|  | 7biii | State whether evaluators were blinded to the identity of the generative AI-driven chatbot(s) under assessment. | Raters and scoring process; Strengths and limitations |
| Sample size | 8 | Report how the sample size was determined. | Sample size |
| Data analysis | 9a | Describe statistical analysis methods, including any evaluation of reproducibility of generative AI-driven chatbot responses. | Prompting and query strategy; Statistical Analysis; Appendices 5/8 |
|  | 9ai | Report the measures used for performance evaluation. | Performance measures |
| Results |  |  |  |
|  | 10a | Report the performance evaluation undertaken including the alignment between generative AI-driven chatbot output and ground truth or reference standard using quantitative or mixed methods approaches as applicable. | Results; Appendices 5/7 |
|  | 10b | For responses deviating from the ground truth or reference standard, state the nature of the difference(s). | Results – safety, accuracy, and empathy; Appendix 6 |
|  | 10c | Report the evaluation for potentially harmful, biased, or misleading responses. | Results – safety, accuracy, and empathy; Appendix 6 |
| Discussion |  |  |  |
|  | 11a | Interpret study findings in the context of relevant evidence. | Principal findings; Comparison with prior work and methodological implications |
|  | 11b | Describe the strengths and limitations of the study. | Strengths and limitations |
|  | 11c | Describe the potential implications for practice, education, policy, regulation, and research. | Clinical and public health implications |
| Open science |  |  |  |
| Disclosures | 12a | Report any relevant conflicts of interest for all authors. | Competing interests |
| Funding | 12b | Report sources of funding and their role in the conduct and reporting of the study. | Funding |
| Ethics | 12c | Describe the process undertaken for ethical approval. | Ethics approval and consent to participate |
|  | 12ci | Describe the measures taken to safeguard data privacy of patient health information, as applicable. | Data privacy and copyright |
|  | 12cii | State whether permission/licensing was obtained for the use of original, copyrighted data. | Data privacy and copyright |
| Protocol | 12d | Provide a study protocol. | N/A (no separate study protocol) |
| Data availability | 12e | State where study data, code repository, and model parameters can be accessed. | Data availability; Appendices 2–8 |

Footer Note: AI, artificial intelligence. Locations are reported by manuscript section heading and supplementary appendix/table identifier rather than page number.
